# Complete biosynthesis of the potent vaccine adjuvant QS-21

**DOI:** 10.1038/s41589-023-01538-5

**Published:** 2024-01-26

**Authors:** Laetitia B. B. Martin, Shingo Kikuchi, Martin Rejzek, Charlotte Owen, James Reed, Anastasia Orme, Rajesh C. Misra, Amr El-Demerdash, Lionel Hill, Hannah Hodgson, Yuzhong Liu, Jay D. Keasling, Robert A. Field, Andrew W. Truman, Anne Osbourn

**Affiliations:** 1 John Innes Centre, Norwich Research Park, Norwich, UK; 2 California Institute of Quantitative Biosciences (QB3), University of California, Berkeley, Berkeley, CA USA; 3 Joint BioEnergy Institute, Emeryville, CA USA; 4 Department of Chemical & Biomolecular Engineering, University of California, Berkeley, Berkeley, CA USA; 5 Department of Bioengineering, University of California, Berkeley, Berkeley, CA USA; 6 Center for Biosustainability, Danish Technical University, Lyngby, Denmark; 7 Center for Synthetic Biochemistry, Shenzhen Institutes for Advanced Technologies, Shenzhen, China; 8 Department of Chemistry and Manchester Institute of Biotechnology, University of Manchester, Manchester, UK; 9 Department of Chemistry, Faculty of Sciences, Mansoura University, Mansoura, Egypt

**Keywords:** Plant sciences, Biochemistry, Biosynthesis, Natural products

## Abstract

QS-21 is a potent vaccine adjuvant currently sourced by extraction from the Chilean soapbark tree. It is a key component of human vaccines for shingles, malaria, coronavirus disease 2019 and others under development. The structure of QS-21 consists of a glycosylated triterpene scaffold coupled to a complex glycosylated 18-carbon acyl chain that is critical for immunostimulant activity. We previously identified the early pathway steps needed to make the triterpene glycoside scaffold; however, the biosynthetic route to the acyl chain, which is needed for stimulation of T cell proliferation, was unknown. Here, we report the biogenic origin of the acyl chain, characterize the series of enzymes required for its synthesis and addition and reconstitute the entire 20-step pathway in tobacco, thereby demonstrating the production of QS-21 in a heterologous expression system. This advance opens up unprecedented opportunities for bioengineering of vaccine adjuvants, investigating structure–activity relationships and understanding the mechanisms by which these compounds promote the human immune response.

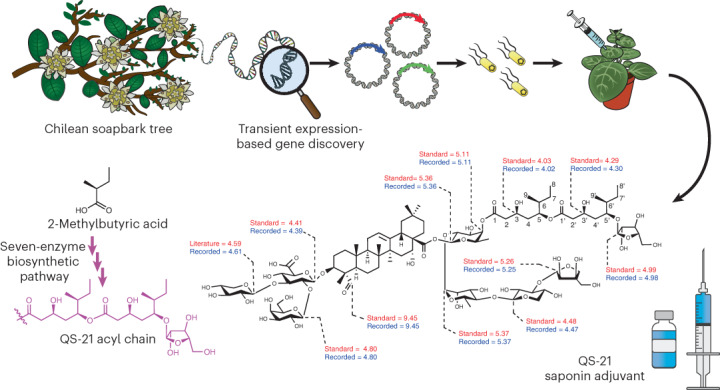

## Abstract (web summary)

Vaccine immunoadjuvants are central to vaccine efficiency. Now, the complete characterization of the biosynthetic pathway of QS-21, a potent immunoadjuvant produced by the Chilean soapbark tree, has been reported. These findings open the door to heterologous production of QS-21 and new-to-nature adjuvants.

## Main

Adjuvants are added to vaccines to boost the immune response. Until recently, the only adjuvants available for clinical use were aluminum salts either alone or in proprietary mixtures and oil-in-water emulsions containing squalene; these adjuvents have relatively low potency and notable side effects, respectively^1^. Natural surfactants (saponins) have been used as adjuvants in veterinary medicine for almost a century. In 2017, a breakthrough came when the first saponin adjuvant AS01 was approved for use in a human vaccine, the highly effective shingles vaccine Shingrix (produced by GSK^2^). AS01 is a liposome-based formula that contains monophosphoryl lipid A and a saponin known as QS-21, which act synergistically to induce strong antibody and helper T cell responses.

QS-21 is produced by the Chilean soapbark tree *Quillaja saponaria*. A crude aqueous extract from the bark of *Q. saponaria* has been used extensively for animal vaccines under the name QuilA. However, QuilA is unsuitable for human use due to its toxicity. Kensil et al.^3^ fractionated *Q. saponaria* bark extract by reverse-phase chromatography and identified four fractions with adjuvant properties (fractions QS-7, QS-17, QS-18 and QS-21). The most abundant saponin component QS-18 showed high toxicity in animal models, whereas QS-7 and QS-21 were less toxic. QS-21 is far more abundant in bark extract than QS-7 and was therefore identified as a promising saponin adjuvant. After 20 years of development, QS-21 is now a key component of several saponin-adjuvanted human vaccines, including Shingrix, the malaria vaccine Mosquirix (also produced by GSK) and ‘Matrix-M’, a combination adjuvant containing a mixture of QS saponins, including QS-21, QS-17 and QS-7, used in the NVX-CoV2373 coronavirus disease 2019 vaccine produced by Novavax. The approval of these vaccines will inevitably lead to increased demand on the existing QS-21 supply chain.

**Fig. 1:**
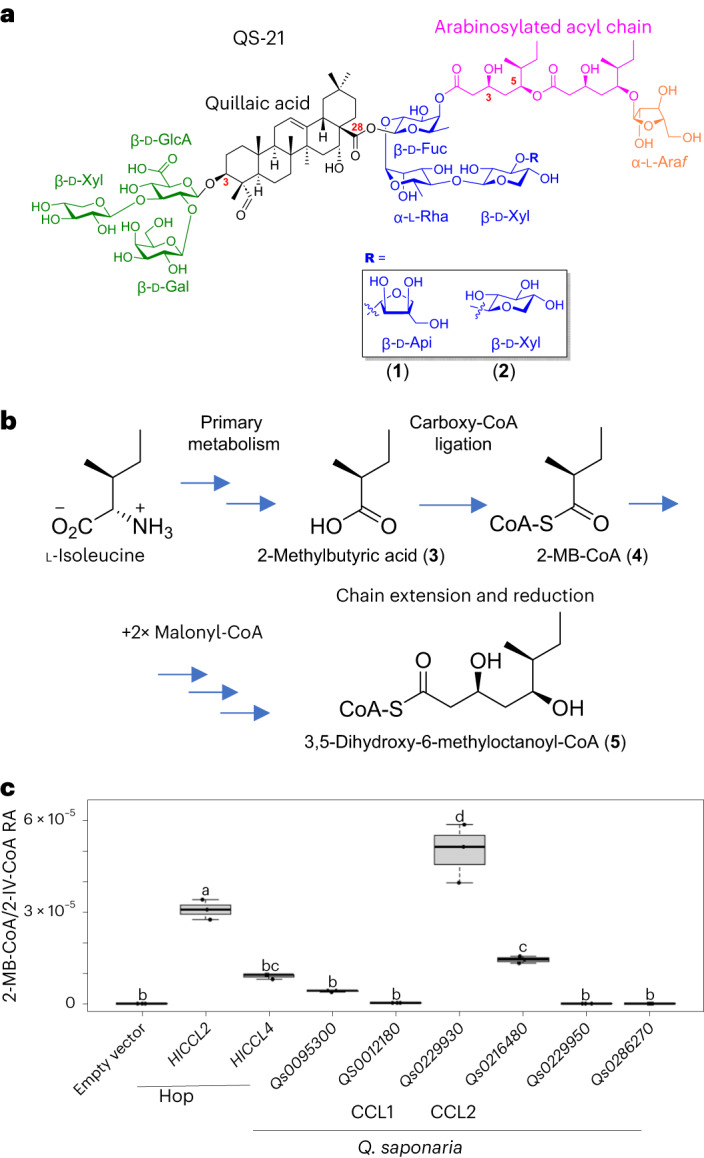
Structure of QS-21 and functional analysis of *Q. saponaria* CCL enzymes. **a**, Structure of QS-21. The quillaic acid core, the C3 sugar chain and the C28 sugar chain are shown in black, green and blue, respectively. The major components of fraction QS-21 are the congeners **1** and **2**, having either d-apiofuranose (d-Api*f*) or d-xylopyranose (d-Xyl*p*) at **R**. We previously characterized the enzymes for the biosynthesis of the quillaic acid core with the C3 and C28 sugar chains attached^7^. The steps required for the biosynthesis and addition of the 18-carbon acyl chain (shown in pink) attached to the C28 β-d-fucopyranose (d-Fuc*p*) and the terminal l-Arabinofuranose (l-Ara*f;* shown in orange) are the targets of this study. d-GlcA, d-glucuronic acid; d-Gal, d-Galactose, l-Rha, l-Rhamnose. **b**, Proposed origin of the C_9_ acyl unit of QS-21. **c**, Functional analysis of candidate *Q. saponaria* CCL genes in yeast. Yeast extracts were analyzed by LC–MS, and the ion transition 852.205 → 345.183 was used for detection and quantification of the branched short-chain acyl-CoA molecules 2-MB-CoA/IV-CoA. These compounds coelute and were therefore analyzed as a pool; RA, relative amounts. The hop CCLs HlCCL2 and HlCCL4, which produce IV-CoA and 2-MB-CoA, respectively^10^, were included as controls. The box plots show the distributions of the values, represented by the dots, for three biologically independent yeast cultures per treatment. The center line represents the median, the box shows the lower and upper quartile values, and the whiskers represent the minimum and maximum data values. Letters represent significantly different data as determined by a two-sided post hoc Tukey’s honestly significant difference test (*P* = 0.05) after analysis of variance (d.f. = 8, *P* = 2.04 × 10^−12^) using the R multcompView package. Source data

The major components of the QS-21 fraction are the saponin isomers **1** and **2** (Fig. 1a). Both have a central triterpene core (quillaic acid) with a branched trisaccharide chain at the C3 position and a linear tetrasaccharide chain at the C28 position. In addition, they have a glycosylated C_18_ acyl chain linked to the saponin core via an ester link to the initial sugar of the C28 sugar chain (d-fucose). The two saponins differ in the nature of the terminal sugar residue of the tetrasaccharide chain, the major and minor compounds having d-apiofuranose (d-Api*f*; **1**; 65%) or d-xylopyranose (d-Xyl*p*; **2**; 35%), respectively^3,4^. QS-21 is one of the most potent adjuvants known^1^. The ability of QS-21 to stimulate cytotoxic T cell proliferation is dependent on the lipophilic acyl side chain^1,3,5^. This acyl chain is highly unusual and particular to saponins from *Quillaja* species^6^. We previously identified the genes required for the biosynthesis of the triterpene glycoside scaffold (Fig. 1)^7^. However, the origin of the acyl group remained unknown. Here, we elucidate the biosynthetic steps and underlying mechanisms required for the addition of the C_18_ acyl chain and reconstitute the entire QS-21 pathway in tobacco.

## Results

### Speculative biosynthetic route

Although triterpenes and other natural products are often acylated, the length and complexity of the QS-21 glycosylated acyl group are highly unusual (Fig. 1a). Analysis of the chemical databases confirmed that the QS-21 dimeric C_18_ acyl chain is unique and restricted to saponins produced by *Q. saponaria* and the closely related species *Q. brasiliensis* (Supplementary Fig. 1 and Methods). The biosynthetic route leading to the formation of this glycosylated acyl chain is unknown. However, based on the dimeric structure of the C_18_ acyl chain of QS-21, we hypothesized that it is constructed by the ligation of two C_9_ acyl units. The branched nature of the acyl chain suggests that these acyl units may be derived from the branched-chain amino acid l-isoleucine, especially as the stereochemistry is consistent with this hypothesis. We speculated that l-isoleucine may be converted into a short-chain acyl-coenzyme A (CoA), most likely (*S*)-2-methylbutyryl-CoA (2-MB-CoA; **4**; Fig. 1b), because this occurs naturally^8^ and is a plausible precursor for the biosynthesis of a C_9_ acyl unit. From this point, two different routes could potentially lead to the C_9_ monomer. In the first route, 2-MB-CoA (**4**) is used as a starter unit by a polyketide synthase (PKS). Two rounds of chain extension would then take place, each with reduction of the resulting keto group. Type III PKSs (PKSIIIs; chalcone synthases and chalcone synthase-like enzymes) are commonly used by plants to catalyze chain extension reactions, although these pathways usually result in non-reduced products^9^.

### Pathway initiation

We first investigated the biosynthetic route to the predicted CoA-activated substrate 2-MB-CoA (**4**) because this is the start of both potential proposed pathways. Degradation of branched short-chain amino acids and their conversion into the corresponding acyl-CoAs occurs in the mitochondrion in plant cells^8^. By analogy with the biosynthesis of bitter acids in hop (*Humulus lupulus*)^10^, it is likely that the CoA group is removed by a thioesterase to allow export of 2-methylbutyric acid (**3**) to the cytosol, where CoA is then reattached by carboxyl-CoA ligases (CCLs). The CoA-activated molecule is then available as a substrate for further modification by PKSIIIs and other cytosolic enzymes. We therefore initiated a search for CCLs from *Q. saponaria* with the aim of finding an enzyme that catalyzes CoA activation of 2-methylbutyric acid (**3**) in the cytosol.

**Extended Data Fig. 1:**
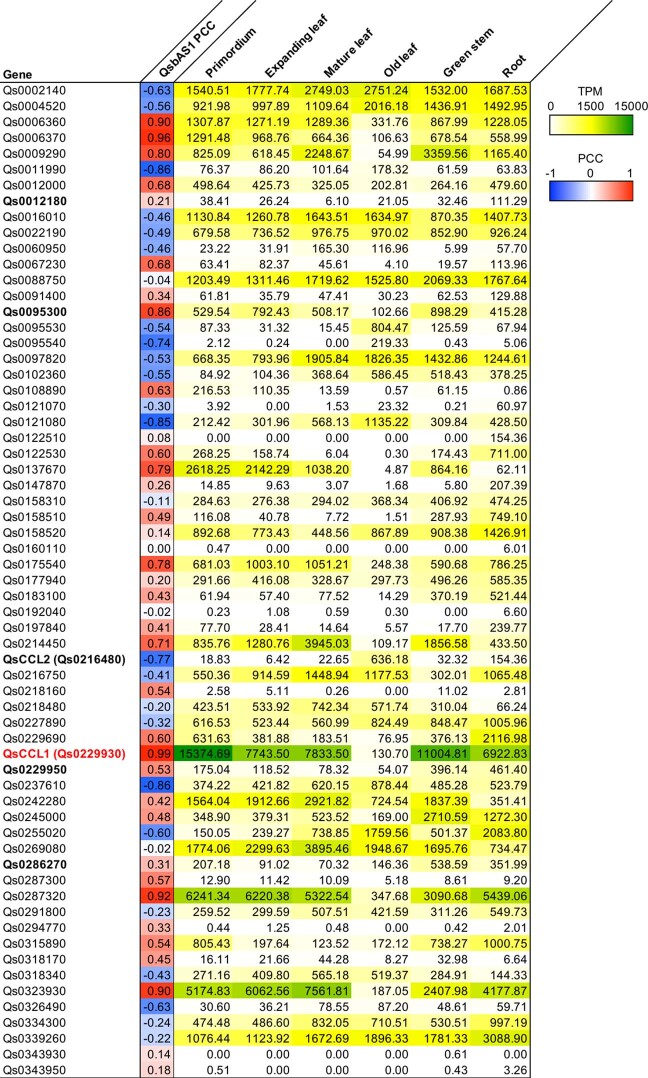
Expression data for predicted *Q. saponaria* CCL genes. Co-expression PCC values with *QsbAS1* and DeSeq normalised read counts (TPM) for six tissues are shown. *QsCCL1* (*Qs0229930*) is indicated in red. This gene and the five additional closely related genes indicated in bold (Fig. S2) were also cloned and tested for function.

We previously elucidated the early pathway steps for the biosynthesis of quillaic acid-based saponins bearing the C3 trisaccharide and C28 tetrasaccharide chains, as found in QS-21 (Fig. 1a)^7^. In the current study, we mined the *Q. saponaria* genome for all predicted acyl-activating enzymes and recovered a total of 63 genes. Phylogenetic analysis revealed seven clades of acyl-activating enzymes, consistent with a prior investigation of this enzyme superfamily in *Arabidopsis thaliana*, moss and poplar^11^ (Supplementary Fig. 2). The predicted *Q. saponaria* CCL that was most highly coexpressed with *QsbAS1*, the gene encoding the enzyme for the first committed step in the QS-21 pathway^7^, was *Qs0229930* (Pearson correlation coefficient (PCC) ≥ 0.99; Extended Data Fig. 1). *CCL1*, like *QsbAS1*, is also expressed at high levels in the primordia (Extended Data Fig. 1) and is strongly coexpressed with the other previously characterized *Q. saponaria* saponin biosynthetic pathway genes^7^ (Supplementary Fig. 3). Phylogenetic analysis revealed that CCL1, which was selected de novo using the unbiased approach outlined above, was located in subgroup VI of the acyl-activating enzyme superfamily, clustered with two previously characterized enzymes that are involved in the biosynthesis of bitter acids in hop trichomes (HlCCL2 and HlCCL4; Supplementary Fig. 2). HlCCL4 ligates a CoA group onto 2-methylbutyric acid (**3**; derived from isoleucine), whereas the preferred substrate of HlCCL2 is isovaleric acid (derived from leucine). *Qs0229930*, five other closely related subgroup VI *Q. saponaria* CCLs and another gene with a PCC of >0.95 (*Qs0006370*; Supplementary Fig. 2 and Extended Data Fig. 1) were cloned into an expression vector for functional analysis in yeast, the expression system previously used for characterization of the hop enzymes HlCCL2 and HlCCL4 (ref. ^10^). Preliminary results showed no detectable activity for Qs0006370, and it was therefore not considered further.

Direct measurement of acyl-CoAs in vivo is notoriously difficult, and so CCL enzyme activity has previously been performed by proxy (by detecting the products of modification of acyl-CoAs by secondary enzymes)^10,12^. To measure short-chain CoA thioesters directly, we adapted a liquid chromatography–electrospray ionization–tandem mass spectrometry (LC–ESI–MS/MS)-based method developed by Gläser et al.^12^. To confirm the identity of 2-MB-CoA (**4**), we made a synthetic standard (Supplementary Fig. 4 and Methods). However, 2-MB-CoA (**4**) and the isobaric species isovaleryl-CoA (IV-CoA) coeluted under our C_18_ reversed-phase chromatography conditions, and both species were therefore regarded as one pool. Functional analysis of *Q. saponaria* CCL candidates was performed in yeast (Fig. 1c), with the hop HlCCL2 and HlCCL4 enzymes included as controls. HlCCL2 yielded significantly higher levels of 2-MB-CoA/IV-CoA than HlCCL4. Of the six *Q. saponaria* CCLs tested, Qs0229930 (hereafter named CCL1) yielded the highest levels of 2-MB-CoA/IV-CoA, Qs0216480 (hereafter named CCL2) had a lower level of activity, and the other CCL candidates had little or no activity (Fig. 1c). Conversely, CCL2 yielded high levels of isobutyryl-CoA and CCL1 yielded moderate levels, whereas the other four *Q. saponaria* CCL enzymes and the hop enzymes HlCCL2 and HlCCL4 generated little or no detectable levels of this short-chain acyl-CoA (Supplementary Fig. 5). Collectively these findings implicate CCL1 in the generation of 2-MB-CoA (**4**), the likely starting substrate for the biosynthesis of the acyl chain of QS-21 in *Q. saponaria*.

### Role of the PKSIII enzymes

**Fig. 2:**
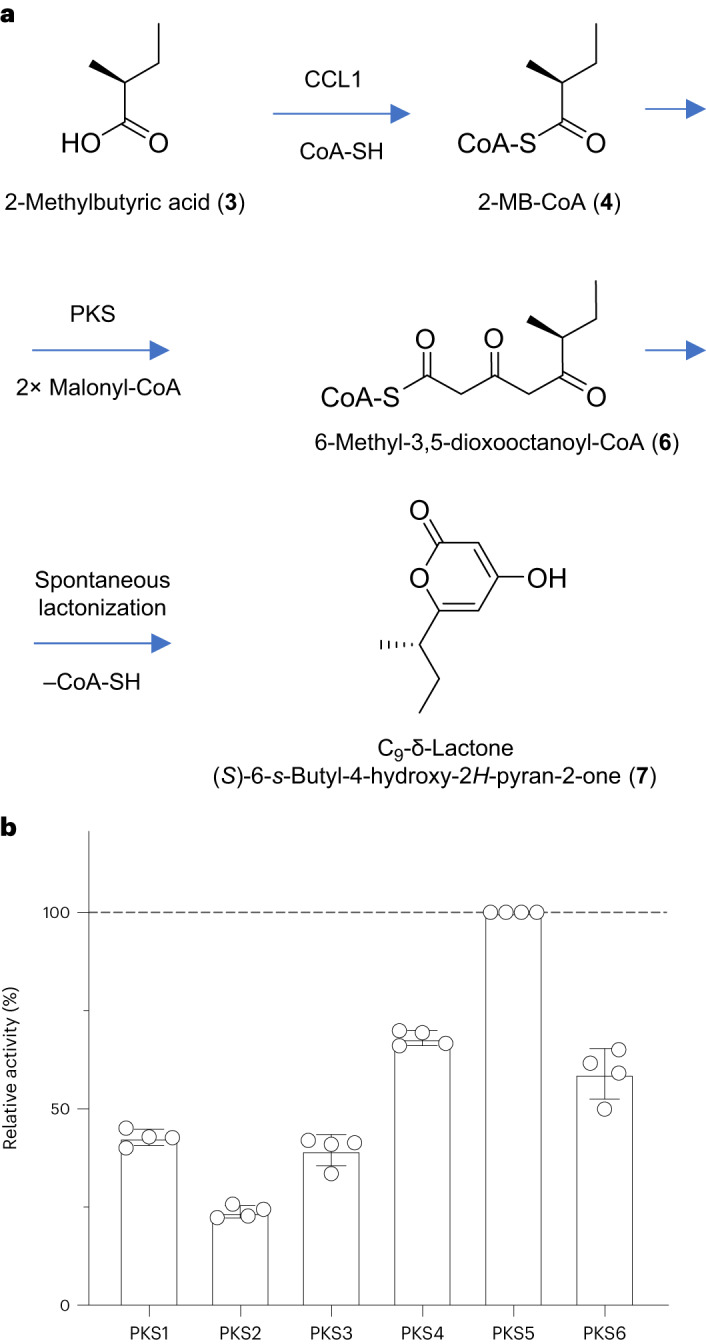
Biosynthesis of the C_9_ acyl chain monomer. **a**, Proposed pathway for the biosynthesis of 6-methyl-3,5-dioxooctanoyl-CoA (**6**), the presumed C_9_ acyl chain monomer used in the biosynthesis of the QS-21 acyl chain. In solution, **6** is unstable and is predicted to undergo spontaneous formation to a C_9_-δ-lactone (**7**) with concomitant release of CoA-SH. Structural elucidation of **7** revealed it to be an uncharacterized compound, (*S*)-6-*s*-butyl-4-hydroxy-2*H*-pyran-2-one (Supplementary Figs. 8 and 9). **b**, In vitro activity of the six selected *Q. saponaria* PKSIII enzymes, PKS1–PKS6. The peak area corresponding to the C_9_-δ-lactone (**7**) was measured after separation by HPLC. The amount of product generated by PKS5 is set at 100%. Data are shown as mean ± s.d. (four biological replicates). Source data

**Extended Data Fig. 2:**
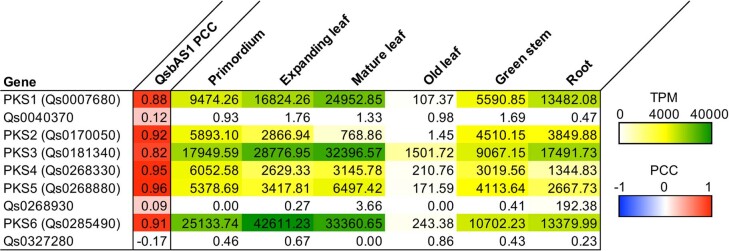
Expression data for predicted *Q. saponaria* PKSIII genes. Co-expression PCC values with *QsbAS1* and DeSeq normalised read counts (TPM) for six tissues are shown. The six PKSIII genes selected for functional analysis are labelled PKS1-6.

**Extended Data Fig. 3:**
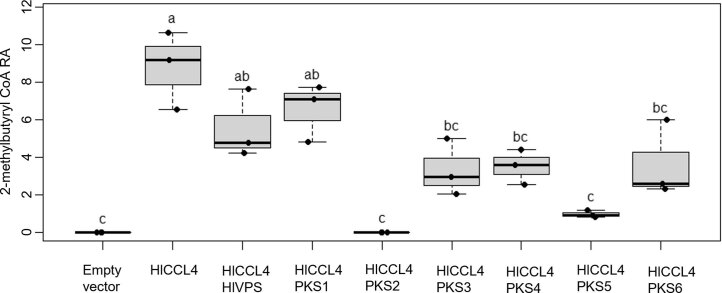
Utilisation of HlCCL4-generated 2-methylbutyryl Co-A (4) by *Q. saponaria* PKS enzymes in yeast. The *Q. saponaria* candidate PKS enzymes PKS1-6 were co-expressed in yeast with the hop HlCCL4 enzyme, which produces 2-MB-CoA (**4**)^10^. RA, relative amounts. The hop PKS enzyme HlVPS was also co-expressed with HlCCL4 as a positive control, since this enzyme is known to utilize (**4**)^10^. Letters represent significantly different data as determined by the two-sided post-hoc Tukey’s HSD (p = 0.05) after ANOVA (Df = 8, p Value = 2.69E^−06^) using the multcompView package in R. The boxplots show the distributions of the values for three biologically independent yeast cultures per treatment (represented by the dots), the centre line representing the median, the box showing the lower and upper quartile values and the whiskers representing the minimum and maximum data values. A Waters Xevo TQ-S Tandem LC-MS system was used to detect 2-methylbutyryl-CoA, using the 852.205 - > 345.183 ion transition (Methods). Source data

**Extended Data Fig. 4:**
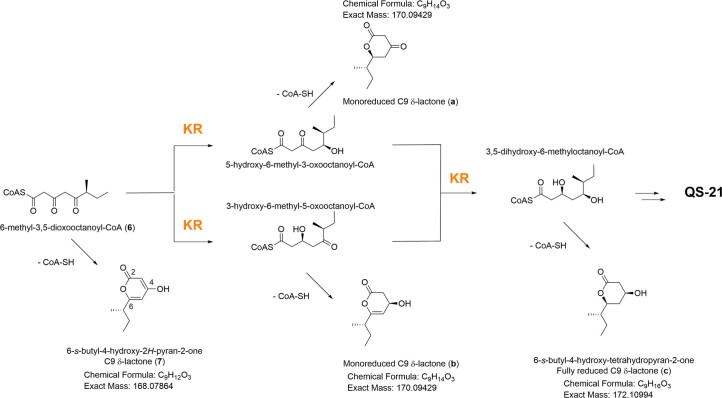
A summary of hypothetical intermediates and degradation products of the C9 acyl unit. 6-Methyl-3,5-dioxooctanoyl-CoA (**6**) is reduced by a keto-reductase to produce either 5-hydroxy-6-methyl-3-oxooctanoyl-CoA or 3-hydroxy-6-methyl-5-oxooctanoyl-CoA. These intermediates are then further reduced to give 3,5-dihydroxy-6-methyloctanoyl-CoA. These CoA-derivatives are unstable and prone to spontaneous chemical degradation to give C9 δ-lactone (**7**), monoreduced C9-δ-lactone (**a**), monoreduced C9-δ-lactone (**b**), or fully reduced C9-δ-lactone (**c**), respectively. KR, keto-reductase.

Having identified the source of 2-MB-CoA (**4**), we next investigated the possibility that the acyl chain may originate via the polyketide route by searching for predicted PKSIII enzymes that could potentially use 2-MB-CoA (**4**) as a starter unit for chain extension^13–16^. Mining of the *Q. saponaria* genome identified a total of nine predicted PKSIII genes (Supplementary Fig. 6). Based on the degree of coexpression with *QsbAS1* and overall expression levels in primordial tissue, six of these genes were selected for functional analysis (*PKS1*–*PKS6*; Extended Data Fig. 2). In preliminary experiments involving coexpression with the hop enzyme HlCCL4 (which generates 2-MB-CoA (**4**)) in yeast, expression of each of the six *Q. saponaria* PKSIII enzymes led to a reduction in the abundance of **4**, indicating that they are all able to use this compound as a substrate, with PKS2 and PKS5 being the most effective (Extended Data Fig. 3). To further investigate the properties of these enzymes, we expressed His-tagged PKS constructs in *Nicotiana benthamiana* by transient expression (Supplementary Fig. 7) and, following one-step metal affinity purification, performed in vitro assays for chain extension of 2-MB-CoA (**4**) in the presence of malonyl-CoA. In preliminary experiments with PKS4, for which we recovered the highest yield of purified protein, high-performance LC (HPLC) analysis revealed that this enzyme was able to perform complete conversion of 2-MB-CoA (**4**) to a hydrophobic product (Supplementary Fig. 8). However, the observed mass of this compound (*m*/*z* 169.09, ESI^+^ [M + H]^+^) did not correspond to the mass (*m*/*z* 936.20, ESI^+^ [M + H]^+^) of the expected product 6-methyl-3,5-dioxooctanoyl-CoA (**6**; Fig. 2a and Supplementary Fig. 9). We therefore performed a large-scale reaction and purified 0.4 mg of this product. Extensive NMR (Supplementary Figs. 10 and 11) revealed this to be an uncharacterized compound, a C_9_-δ-lactone, (*S*)-6-*s*-butyl-4-hydroxy-2*H*-pyran-2-one (**7**; Fig. 2a). This C_9_-δ-lactone is the product of spontaneous chemical degradation of 6-methyl-3,5-dioxooctanoyl-CoA (**6**) after C5 enol formation and subsequent lactonization with concomitant release of CoA-SH (Extended Data Fig. 4). We next used the C_9_-δ-lactone as an indirect read-out for 6-methyl-3,5-dioxooctanoyl-CoA (**6**) formation. Analysis of the six *Q. saponaria* PKS enzymes revealed that they all produce the C_9_-δ-lactone (**7**), PKS5 being the most active, followed by PKS4 and PKS6 (Fig. 2b and Supplementary Figs. 12 and 13). The C_9_-δ-lactone (**7**) was also detected when CCL1 was coexpressed with *Q. saponaria* PKSs in *N. benthamiana* by *Agrobacterium tumefaciens*-mediated transient expression^17^ (Supplementary Fig. 14). However, it was not detected in extracts from *Q. saponaria* primordia and old leaves, suggesting that it is an artifact of the PKS-mediated enzymatic reaction in vitro and in the heterologous expression host *N. benthamiana* (Supplementary Fig. 15a). We speculate that in *Q. saponaria*, 6-methyl-3,5-dioxooctanoyl-CoA (**6**) may be stabilized by substrate channeling or possibly a metabolon, thereby preventing spontaneous chemical degradation to the biochemically inactive C_9_-δ-lactone (**7**) and enabling efficient processing of the authentic intermediate (**6**) by downstream pathway enzymes.

Collectively, these findings are consistent with our hypothesis that the biosynthesis of the C_9_ acyl unit occurs by PKS-mediated extension of 2-MB-CoA (**4**; Fig. 2a). 6-Methyl-3,5-dioxooctanoyl-CoA (**6**) is therefore the likely C_9_ acyl chain monomer used in the biosynthesis of the QS-21 acyl chain. The generation of the full C_18_ acyl chain would then require reduction of the keto groups, condensation of two C_9_ acyl monomers and the addition of the terminal α-l-arabinofuranose (l-Ara*f*) in a currently undefined order.

### Identification of the remaining QS-21 biosynthetic genes

The *Q. saponaria* saponin biosynthetic pathway genes that we previously characterized^7^ all show high expression in the primordia and are coexpressed with *QsbAS1* (the gene encoding the enzyme for the first committed pathway step). Some are also located in biosynthetic gene clusters (BGCs)^7^. To identify additional candidate pathway genes, we used a scoring system that takes into account these parameters (Methods). Our prioritized list of 68 candidate genes included 55 genes predicted to encode classes of enzymes likely to be involved in the missing downstream steps required for biosynthesis and the addition of the acyl chain (reductases, acyl transferases and glycosyl transferases; Supplementary Data 1).

**Fig. 3:**
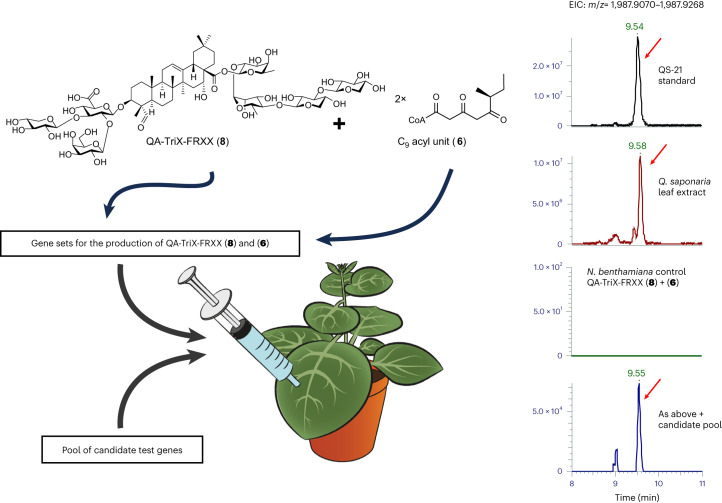
Expression of candidate genes for downstream pathway steps in *N. benthamiana* with a shotgun approach. Detection of a product with the same retention time and mass spectrum as a QS-21 standard in extracts of *N. benthamiana* leaves following shotgun expression of a pool of 68 candidate genes together with the genes required to make the glycosylated triterpene QA-TriX-FRXX (**8**) and the acyl chain monomer 6-methyl-3,5-dioxooctanoyl-CoA (**6**; QsCCL1 together with PKS1–PKS6). LC–MS extracted ion chromatograms (EICs) in negative mode for a QS-21 standard, *Q. saponaria* leaf extract and *N. benthamiana* leaf extracts following expression of the gene sets for **8** and **6** without and with the pooled candidate gene tester set are shown. A product with a retention time, *m*/z (1,987.9) and mass spectrum consistent with that of QS-21 (**2**) was only detected in the *N. benthamiana* leaf extracts when the candidate pool was introduced. Further information about the gene expression constructs and methods used for transient expression can be found in the Methods.

Evaluation of the functions of these candidate genes in vitro is confounded by the instability of 6-methyl-3,5-dioxooctanoyl-CoA (**6**) and by the lack of knowledge of the order in which these events occur. We therefore elected to take a ‘shotgun’ approach to identify the missing pathway steps using *A. tumefaciens*-mediated transient gene expression in *N. benthamiana*. Combinatorial expression of biosynthetic enzymes in *N. benthamiana* is a powerful strategy for characterizing gene function, reconstituting biosynthetic pathways and producing metabolites on a larger scale^17–19^. We previously used this approach to elucidate and reconstitute the pathway for advanced glycosylated triterpene scaffolds from *Q. saponaria*, including 3-*O*-{β-d-xylopyranosyl-(1 → 3)-[β-d-galactopyranosyl-(1 → 2)]-β-d-glucopyranosiduronic acid}-28-*O*-{β-d-xylopyranosyl-(1 → 3)-β-d-xylopyranosyl-(1 → 4)-α-l-rhamnopyranosyl-(1 → 2)-β-d-fucopyranosyl ester}-quillaic acid (hereafter referred to as QA-TriX-FRXX; **8**; Fig. 3), the precursor of the d-Xyl variant of QS-21 (**2**), and determined the full structures of these by extensive one-dimensional (1D) and two-dimensional (2D) NMR^7^. We reasoned that if the enzymes needed for the acyl chain were contained within a larger test gene pool, coexpression of this pool of genes in *N. benthamiana* along with the genes needed to make QA-TriX-FRXX (**8**) and the acyl chain monomer 6-methyl-3,5-dioxooctanoyl-CoA (**6**) would likely result in QS-21 production. Coexpression of a total of around 30 candidate genes by transient expression in *N. benthamiana* has been used successfully to identify cytochrome P450 (CYP) enzymes that catalyze the conversion of cholesterol to diosgenin^20^. We cloned full-length coding sequences for each of our candidates (68 in total; Supplementary Data 1) into the pEAQ-HT expression vector^21^ and individually transformed the resultant constructs into *A. tumefaciens*. These were coinfiltrated as a pool into the leaves of *N. benthamiana* in combination with *A. tumefaciens* strains harboring the genes needed for the biosynthesis of compounds **8** (ref. ^7^) and **6** (*CCL1* and *PKS1*–*PKS6*; a total of 12 strains; see Methods for further information). LC–MS metabolite analysis of leaf extracts 5 d after agroinfiltration revealed that coexpression of the candidate gene pool resulted in formation of a new peak with the same retention time, mass and MS^2^ fragmentation spectrum as the d-Xyl chemotype of QS-21 (**2**; Fig. 3 and Supplementary Fig. 16).

**Extended Data Fig. 5:**
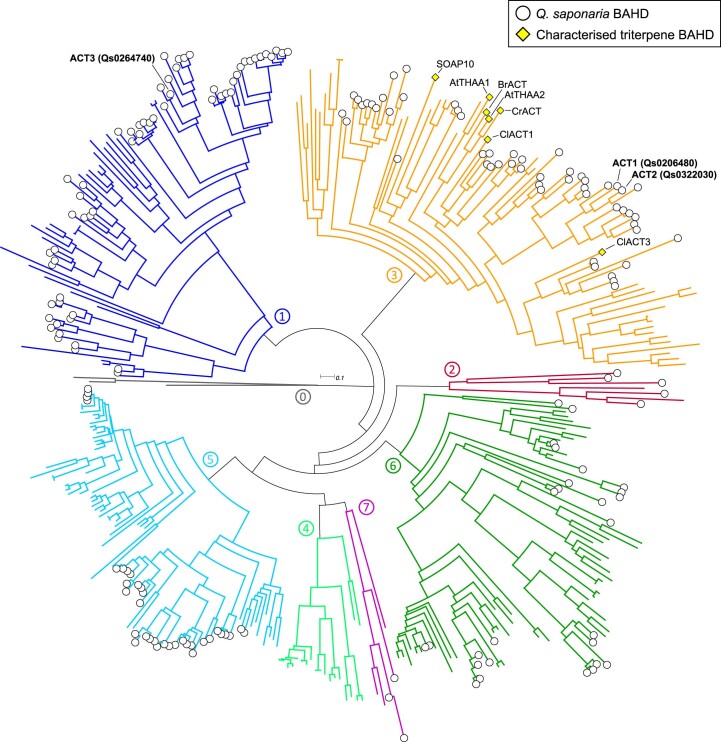
Phylogeny of *Q. saponaria* BAHD enzymes. Sequences were mined from the *Q. saponaria* genome using the PF02458 and IPR003480 domains, producing 182 candidate BAHDs. Sequences from^45^ were used as reference for clade assignment. The following additional characterised BAHDs were included and labelled: AtTHAA1 (At5g47980, *Arabidopsis thaliana*), AtTHAA2 (AT5G47950, *A. thaliana*), BrACT (XP_009117330.1, *Brassica rapa*), CrACT (Carubv10017289m, *Capsella rubella*), ClACT1 (Cla007081, *Citrullus lanatus*), ClACT3 (Cla022713, *C. lanatus*) and SOAP10 (KNA12459.1, *Spinacia oleracea*). Labelled clade numbers corresponding to those described in^45^. Sequences were aligned and phylogenies generated as described above. The scale bar indicates the number of amino acid substitutions per site. The *Q. saponaria* BAHD enzymes involved in biosynthesis of the QS-21 acyl chain, ACT2 (group 3) and ACT3 (group 1), are indicated. The *Q. saponaria* BAHD ACT1 was previously shown to transfer an acetyl group onto the triterpene glycoside scaffold during the biosynthesis of the simpler *Q. saponaria* saponin QS-7^7^.

**Extended Data Fig. 6:**
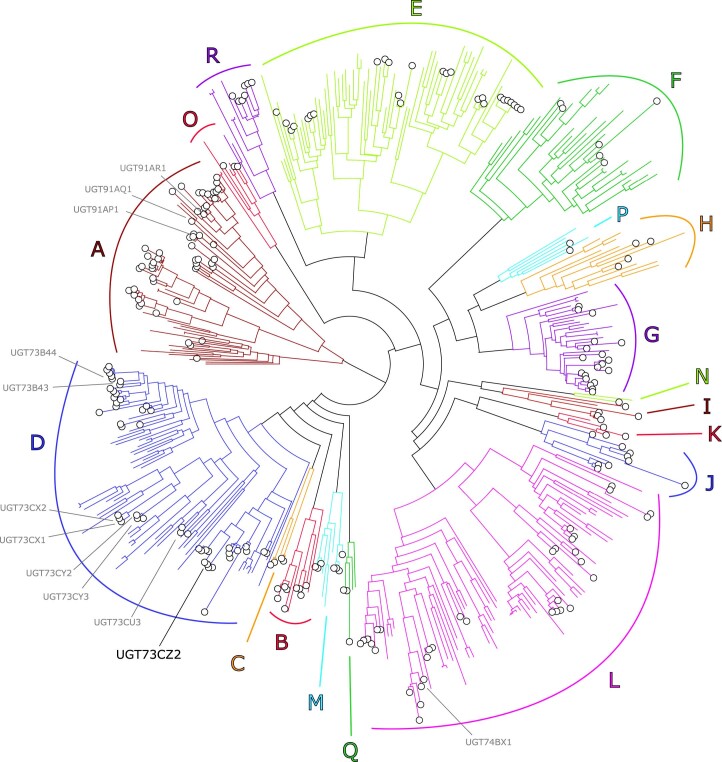
Phylogeny of *Q. saponaria* UGT enzymes. The tree was generated as described in^7^. UGTs from *Q. saponaria* are indicated with white circles. Characterized UGTs from other species included in the alignment and phylogeny in order to classify the UGTs into the labelled groups are from^46^. The UGT names shown in grey are *Q. saponaria* UGTs previously shown to be required for saponin biosynthesis^7^. The UGT reported in the current study, UGT73CZ2, is labelled in black.

To identify the specific enzymes responsible for the biosynthesis and addition of the C_18_ acyl chain, we performed further experiments in which we removed a subset of candidates from the test pool each time. Through successive rounds of testing, we established that, in addition to CCL1 and the PKSs, a further five enzymes are required to build the QS-21 acyl chain, specifically two ketoreductases (KR1 and KR2, encoded by *Qs0326850* and *Qs0235370*, respectively), two BAHD acyl transferases (ACT2 and ACT3, encoded by *Qs0322030* and *Qs0264740*, respectively) and one sugar transferase (UGT73CZ2, encoded by *Qs0131010*; Supplementary Fig. 17 and Extended Data Figs. 5 and 6). The genes encoding these enzymes all grouped within the top 40 prioritized genes shown in Table 1, which also included the other previously characterized *Q. saponaria* saponin pathway genes (shown in bold)^7^.

**Table 1:** Identification of candidate genes for downstream pathway steps

Gene ID	Enzyme class	Annotation	Score
***Qs0315350***	**Characterized gene**	**Terpene cyclase/mutase family member (QsbAS1)**	**3.00**
***Qs0322000***	**Characterized gene**	**CYP (CYP716A297)**	**3.00**
***Qs0321930***	**Characterized gene**	**Glycosyltransferase (UGT74BX1)**	**3.00**
***Qs0259300***	**Characterized gene**	**CYP (CYP716A224)**	**3.00**
***Qs0321920***	**Characterized gene**	**Glycosyltransferase (UGT91AR1)**	**3.00**
***Qs0123860***	**Characterized gene**	**Glycosyltransferase (UGT73CU3)**	**3.00**
*Qs0268880*	PKSIII	Chalcone synthase (PKS5)	3.00
***Qs0283870***	**Characterized gene**	**Glycosyltransferase (UGT73CX1)**	**3.00**
***Qs0321940***	**Characterized gene**	**Glycosyltransferase (UGT91AP1)**	**3.00**
*Qs0322030*	Acyltransferase	Vinorine synthase-like (ACT2)	3.00
***Qs0234120***	**Characterized gene**	**Glycosyltransferase (UGT91AQ1)**	**3.00**
***Qs0283850***	**Characterized gene**	**Glycosyltransferase (UGT73CX2)**	**3.00**
*Qs0234150*	Glycosyltransferase	Glycosyltransferase	2.91
*Qs0234050*	Other	CYP	2.80
*Qs0326850*	Reductase	Cinnamoyl-CoA reductase (KR1)	2.50
*Qs0175050*	Other	ATP-citrate synthase β-chain protein	2.50
*Qs0162400*	Other	Squalene monooxygenase-like	2.50
*Qs0081220*	Other	CYP	2.50
*Qs0264740*	Acyltransferase	Anthocyanin 5-aromatic acyltransferase (ACT3)	2.50
*Qs0149710*	Other	ATP-citrate synthase α-chain protein	2.50
*Qs0131010*	Glycosyltransferase	Glycosyltransferase (UGT73CZ2)	2.50
*Qs0233700*	Glycosyltransferase	Glycosyltransferase	2.50
*Qs0082410*	Glycosyltransferase	Glycosyltransferase	2.50
*Qs0152180*	Glycosyltransferase	Glycosyltransferase	2.50
*Qs0098630*	Acyltransferase	Vinorine synthase-like	2.50
*Qs0298840*	Other	Trifunctional RHM1-like	2.50
*Qs0007520*	Other	2-Oxoglutarate and Fe(II)-dependent oxygenase superfamily	2.50
*Qs0264720*	Acyltransferase	Malonyl-coenzyme:anthocyanin 5-*O*-glucoside-6′′′-*O*-malonyltransferase	2.50
*Qs0124580*	Other	ATP-citrate synthase α-chain protein	2.50
*Qs0020840*	Other	Patellin-3-like	2.50
*Qs0287320*	Other	4-Coumarate-CoA ligase-like	2.50
*Qs0004900*	Other	ATP-citrate synthase β-chain protein	2.50
*Qs0264710*	Acyltransferase	Malonyl-coenzyme:anthocyanin 5-*O*-glucoside-6′′′-*O*-malonyltransferase	2.50
*Qs0098610*	Glycosyltransferase	Glycosyltransferase	2.50
*Qs0285490*	PKSIII	Chalcone synthase (PKS6)	2.50
*Qs0235370*	Reductase	Very-long-chain 3-oxoacyl-CoA reductase 1 (KR2)	2.50
*Qs0060960*	Other	Tubulin β-chain	2.45
*Qs0091410*	Other	C2 domain-containing protein	2.43
***Qs0234130***	**Characterized gene**	**Glycosyltransferase (UGT73CY3)**	**2.38**
*Qs0283860*	Glycosyltransferase (partial)	Glycosyltransferase (partial)	2.30

The top 40 *Q. saponaria* candidate genes for QS-21 biosynthesis. The overall scores were determined by the strength of gene coexpression with *QsbAS1*, absolute transcript abundance in primordial tissue and presence in putative BGCs determined by a modified version of plantiSMASH^28^ (see Methods for details). Previously characterized QS-21 pathway genes^7^ are indicated in bold. PKS5 and PKS6 (annotated as PKSIIIs) are also in the top 40. Candidates for the missing steps for addition of the acyl chain are listed with their corresponding enzyme class, whereas genes irrelevant to this work are labeled as ‘Other’.

### Five additional enzymes needed to complete the QS-21 pathway

**Fig. 4:**
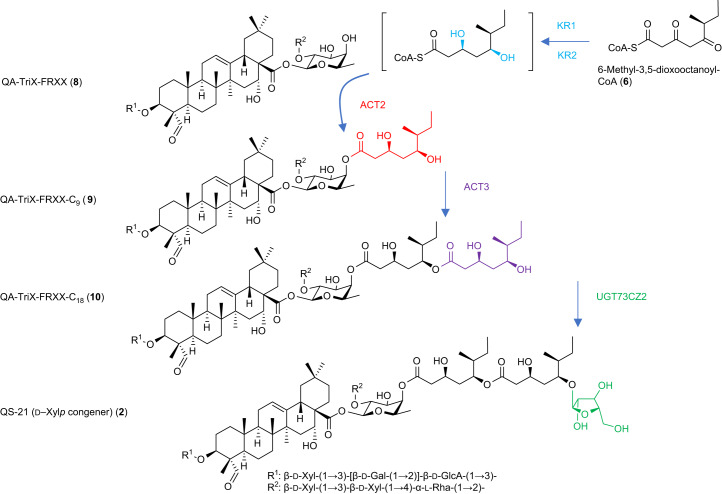
Identification of the five additional genes necessary for biosynthesis and addition of the arabinofuranosylated C_18_ acyl chain of QS-21 (2). Proposed steps for addition of the acyl chain to the triterpene scaffold. Note that the ketoreductions may occur before or after ligation of the C_9_ acyl unit to the triterpene scaffold. Supplementary Table 2 summarizes the LC–MS/MS data of these compounds.

**Extended Data Fig. 7:**
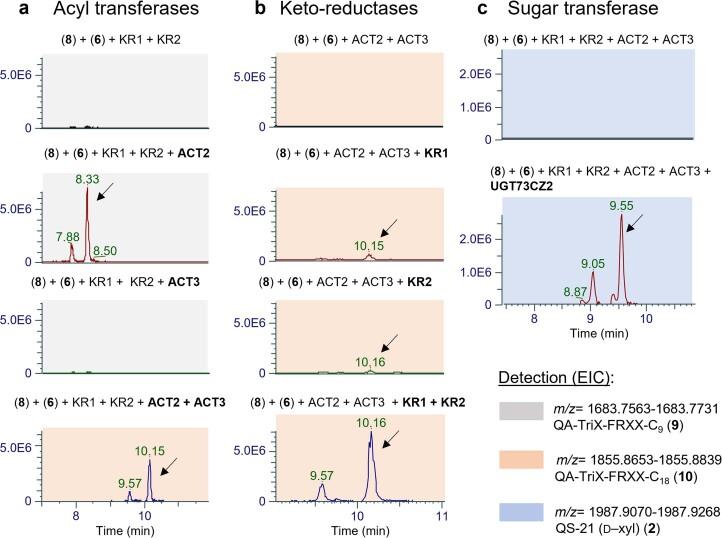
Identification of the five additional genes necessary for biosynthesis and addition of the arabinofuranosylated C18 acyl chain of QS-21 (2). **a**–**c**, Investigation of the contributions of the five candidate enzymes to addition of the acyl chain. Mass spectrometry traces (EICs) are shown. Full details can be found in Supplementary Figs. 22–28. **a**,Co-expression of the acyl transferases ACT2 and ACT3 separately and together in combination with the enzymes needed for the biosynthesis of QA-TriX-FRXX (**8**) and the C9 acyl unit (**6**) and the keto-reductases KR1 and KR2. A putative QA-TriX-FRXX-C9 product (**9**) was synthesised in the presence of ACT2 but not ACT3, while co-expression of ACT2 and ACT3 together yielded a putative QA-TriX-FRXX-C18 product (**10**). Thus ACT2 is likely to ligate the first C9 acyl unit to the triterpene scaffold, and ACT3 the second one. **b**, Co-expression of the keto-reductases KR1 and KR2 separately and together in combination with the enzymes needed for the biosynthesis of QA-TriX-FRXX (**8**) and the C9 acyl unit (**6**) and the acyl transferases ACT2 and ACT3. KR1 and KR2 individually both yield detectable but low levels of the putative QA-TriX-FRXX-C18 (**10**), while co-expression of the two reductases together yields substantially more of the presumed (**10**). **c**, Co-expression of UGT73CZ2 with KR1, KR2, ACT2 and ACT3 yields a product with a mass and fragmentation pattern consistent with QS-21 (**2**). LC-MS extracted chromatograms (EIC) in negative mode are shown. The screened masses of the [M-H]^-^ pseudomolecular ions are: QA-TriX-FRXX-C9 (**9**), *m/z* = 1683.7563–1683.7731; QA-TriX-FRXX-C18 (**10**), *m/z* = 1855.8653–1855.8839; QS-21 (d-xyl) (**2**), *m/z* = 1987.9070–1987.9268.

Further tests of the functions of these five enzymes individually and in combination enabled us to propose a pathway for the addition of the acyl chain (Fig. 4), in which ACT2 transfers the first C_9_ acyl unit to the triterpene scaffold, and ACT3 transfers the second C_9_ acyl unit to yield QA-TriX-FRXX-C_9_ (**9**) and QA-TriX-FRXX-C_18_ (**10**), respectively (Extended Data Fig. 7 and Supplementary Figs. 18 and 19). When the ketoreductases KR1 and KR2 were expressed in combination with the acyl transferases ACT2 and ACT3 and the enzymes needed for the biosynthesis of QA-TriX-FRXX (**8**) and the C_9_ acyl unit (**6**), a peak of the presumed product QA-TriX-FRXX-C_18_ (**10**) was readily detected by LC–MS (Extended Data Fig. 7). Lower levels of this product were observed when the ketoreductases were expressed individually. The acyl chain attached to the triterpene scaffold but retaining the C3 and C5 ketones was not detected in *N. benthamiana* infiltrated with ACT2 with or without KR1 and/or KR2, suggesting that at least one reduction is required for the C_9_ acyl unit to be transferred to the triterpene scaffold. However, small amounts of QA-TriX-FRXX-C_9_ (**9**) retaining one of the acyl donor ketones were detected in the presence of either KR1 or KR2, suggesting that ACT2 has some ability to transfer the partially reduced C_9_ acyl unit to the scaffold (Supplementary Figs. 20–23). The smaller peak typically eluting at a slightly earlier retention time (Extended Data Fig. 7a–c) may be a product of d-fucose-3-OH acylation. This could be the result of limited ACT specificity, but it is more likely that the minor isomer is an artifact of isolation and that it may result from a chemical 4 to 3 acyl migration, as previously reported^22^. The activities of the ketoreductases were further investigated by measuring the presence of the C_9_-δ-lactone and its monoreduced forms (Supplementary Fig. 15). Although the presence of KR1 did not lead to the formation of monoreduced C_9_-δ-lactone, the associated decrease of the C_9_-δ-lactone suggests that KR1 may play a role in stabilizing 6-methyl-3,5-dioxooctanoyl-CoA (**6**), thereby preventing excessive lactonization. The addition of KR2 led to a decrease in C_9_-δ-lactone and a concomitant increase in the levels of monoreduced C_9_-δ-lactone, indicating that KR2 reduces the relevant ketones of **6**. The addition of the remaining QS-21 pathway genes led to marked reduction in the levels of the C_9_-δ-lactone and its monoreduced forms (Supplementary Fig. 15), indicating further stabilization of the substrate.

Last, the addition of the glycosyltransferase UGT73CZ2 yielded a product with a retention time and mass spectrum consistent with the addition of the terminal l-Ara*f* to the C_18_ acyl chain, thereby completing the pathway to the presumed product QS-21 (**2**; Extended Data Fig. 7c and Supplementary Fig. 24). In vitro investigation of the sugar nucleotide donor specificity of UGT73CZ2 using des-l-Ara*f*-QS-21 as the acceptor (QA-TriX-FRXA-C_18_; **11**; purified from *Q. saponaria* bark extract; Methods) revealed that this enzyme prefers using UDP-l-Ara*f* over the majority of other UDP sugar donors (Supplementary Fig. 25). Interestingly, however, it is also able to use UDP-d-Xyl. Of note, an isomer of QS-21 (**Qb1**) that has β-d-Xyl instead of α-l-Ara*f* at the end of the C_18_ acyl chain has recently been reported from the related species *Q. brasiliensis*^23^. Collectively these data are consistent with a role for UGT73CZ2 in catalyzing the final step in the QS-21 pathway.

### Approaches to increase QS-21 yield in a heterologous host

**Extended Data Fig. 8:**
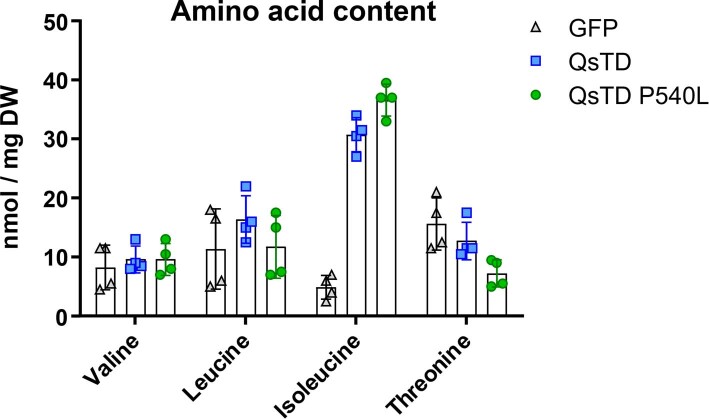
Transient expression of wild type and mutant threonine deaminases increases free isoleucine content in *N. benthamiana*. The *Q. saponaria* QsTD enzyme was transiently expressed in *N. benthamiana* either as the native wild-type protein, or as a predicted feedback-insensitive P540L mutant form. Plants transiently expressing green fluorescent protein (GFP) were used as a negative control. Three days later, leaves were harvested and content of free branched-chain amino acids (BCAAs) (valine, leucine and isoleucine) and threonine were determined by LC-MS. A large increase in the pool of isoleucine could be detected in the plants expressing both wild-type and mutant QsTD compared to the controls, while little variation was detected for the other BCAAs. Data represent the average content from four independent leaves, with error bars showing standard deviation. Source data

**Extended Data Fig. 9:**
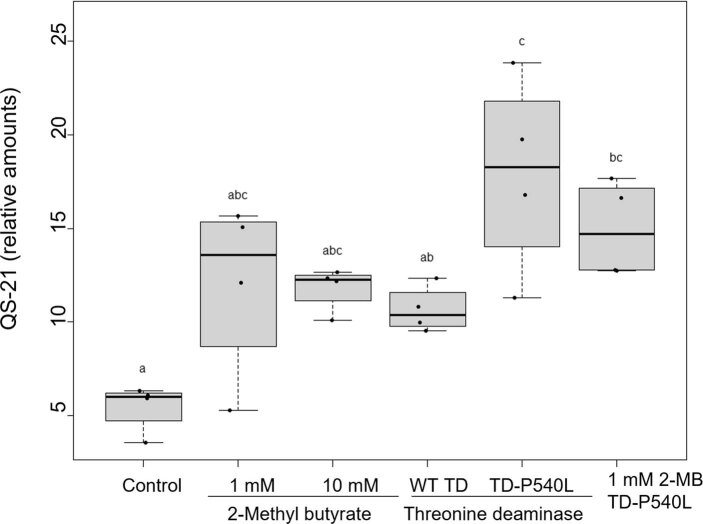
Production of QS-21 (1) in *N. benthamiana*. Evaluation of methods to boost product yield. The gene set for QS-21 was expressed in *N. benthamiana* as follows: agro-infiltration of the QS-21 gene set alone (control); by co-infiltration with 1 or 10 mM 2-MB (**3**); and/or co-expressed with the wild type *Quillaja* threonine deaminase (TD) or a feedback-insensitive QsTD P540L mutant. The average peak areas of QS-21 were normalised to an internal standard (digitoxin, 10 µg/g dry leaf weight). The boxplots show the distributions of the values for four biologically independent infiltrated leaf per treatment (represented by the dots), the centre line representing the median, the box showing the lower and upper quartile values and the whiskers representing the minimum and maximum data values. Letters represent significantly different data as determined by the two-sided post-hoc Tukey’s HSD (p = 0.05) after ANOVA (Df = 5, p Value = 0.000963) using the multcompView package in R. Source data

We previously identified the enzymes needed to generate both the d-Xyl*p* and d-Api*f* variants of the QS-21 tetrasaccharide chain, which furnish the QS-21 variants **2** and **1**, respectively^7^. Our subsequent experiments focused on the d-Api*f* chemotype (**1**) because this was the most abundant component of the two saponins in the QS-21 fraction. Further analysis revealed that the majority of the triterpene glycoside acyl chain acceptor remained unconverted to QS-21, indicating that acylation was inefficient, possibly due to limiting the availability of acyl chain precursors such as 2-methylbutyric acid (**3**; Supplementary Fig. 26). This inefficiency is unlikely to be due to differential compartmentalization of the pathway because, apart from KR2, which is predicted to be targeted to the endoplasmic reticulum, all the glycosyl transferases involved in decorating the triterpene scaffold and the other enzymes required for acylation are predicted to be cytosolic (Supplementary Fig. 27). When the gene set for QS-21 (**1**) was transiently expressed in *N. benthamiana* with 1 mM 2-methylbutyric acid included in the infiltration buffer, the levels of product increased by two- to fourfold (Supplementary Fig. 28), suggesting that this metabolite is limiting in *N. benthamiana* for QS-21 biosynthesis. Given that 2-MB-CoA (**4**) is derived from the breakdown of l-isoleucine (Fig. 1b), we considered that increasing the free l-isoleucine content in leaves might also increase the abundance of 2-MB-CoA through the effect of endogenous homeostatic control mechanisms. l-Isoleucine can be derived from threonine through the action of the plastidic threonine deaminase (TD). Several feedback-insensitive mutants of TD have been characterized from *A. thaliana* (AT3G10050)^24,25^. Of these, a proline-to-leucine substitution at position 519 has been reported to result in greater than 140-fold increases in free isoleucine^25^. We therefore identified and cloned a *Q. saponaria* homolog (*QsTD*), which shows good expression in leaf primordia (*Qs0222940*), and introduced the relevant proline-to-leucine mutation (corresponding to position 540 (QsTD-P540L)). Transient expression of either the wild-type or mutant forms of QsTD resulted in a seven- to eightfold increase in l-isoleucine content in *N. benthamiana* leaves, while little effect was observed for two other branched-chain amino acids, leucine and valine (Extended Data Fig. 8). Coexpression of the wild-type and mutant forms of QsTD together with the gene set for QS-21 (**1**) in both cases resulted in enhanced product levels, with the mutant form yielding levels around threefold higher than for the wild type (Extended Data Fig. 9). Quantification of QS-21 levels in *N. benthamiana* leaf extracts (with QsTD-P540L) revealed this to be 8.6 μg per g dry leaf weight, around three to five times lower than the levels found in *Q. saponaria* leaves^7^. However, supplementing the *N. benthamiana* leaf expressing QsTD-P540L with 2-methylbutyric acid (**3**) did not result in a further increase in QS-21 levels, suggesting that either strategy may be sufficient to resolve substrate limitation. Our demonstration that the feedback-insensitive version of TD QsTD-P540L boosts l-isoleucine levels but not the levels of other branched-chain amino acids in *N. benthamiana* (Extended Data Fig. 8) and leads to increased QS-21 yield (Extended Data Fig. 9) is consistent with our proposal that QS-21 acyl chain biosynthesis is initiated from l-isoleucine, as shown in Fig. 1b.

### Heterologous expression and purification of QS-21

**Fig. 5:**
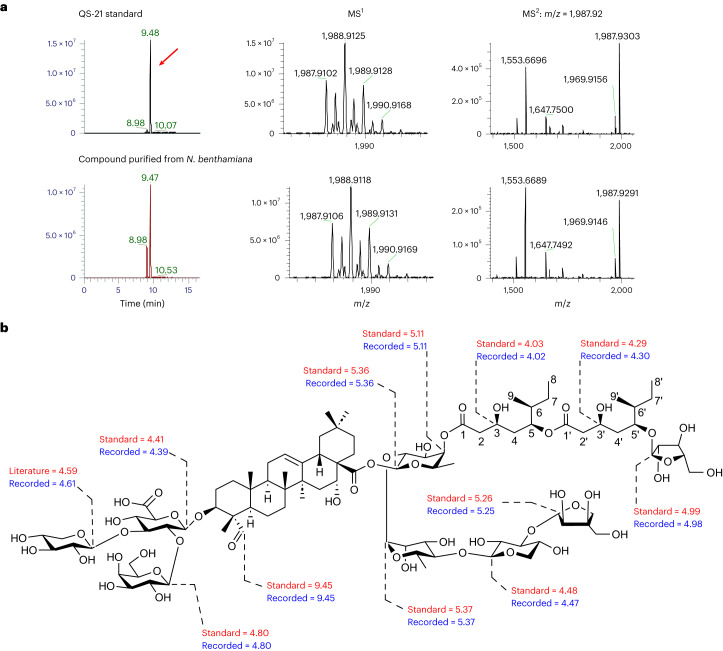
Production of QS-21 (1) in *N. benthamiana*. **a**, Comparison of the retention times, HRMS and MS^2^ data for the product purified from *N. benthamiana* and a commercial standard of QS-21. **b**, ^1^H NMR spectral data for key resonances for the QS-21 standard and the product purified from *N. benthamiana*, recorded in methanol-*d*_4_ at 600 MHz. Full 1D and 2D NMR data can be found in Supplementary Figs. 29 and 30, Extended Data Fig. 10, Supplementary Table 1 and Supplementary Data 6.

**Extended Data Fig. 10:**
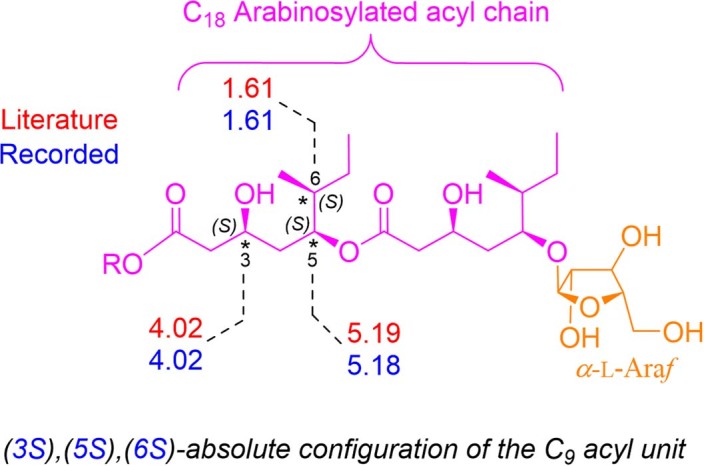
Comparison of ^1^H-NMR chemical shifts (recorded, in blue and literature, in red) of the three stereocentres (3*/5*/6*) of the C9-monomeric acyl unit. The identical proton environments confirm the same relative and absolute stereochemistry of the dimeric C18 acyl chain.

We next scaled up our transient plant expression experiments to provide further evidence that the molecule that we had produced was indeed QS-21 (**1**). We included the feedback-insensitive TD variant TD-P540L to boost product yield. Following vacuum infiltration of ~300 *N. benthamiana* plants, the leaves were freeze-dried, and extracts were subjected to multistep chromatography to yield a semipure non-separable preparation of the presumed QS-21 product. LC–high-resolution MS (LC–HRMS) analysis revealed a compound with a retention time and observed mass matching an authentic standard (Desert King) of QS-21 from *Q. saponaria*, which predominantly contains the d-Api variant (**1**; 94% from ^1^H NMR integration). The retention times of the purified compound and standard were *R*_f_ = 9.47 and 9.48 min, respectively, the observed mass *m*/*z* was 1,987.9106 for [M – H]^–^, and the calculated *m*/*z* was 1,987.9169 with an error of 3.2 ppm (Fig. 5a). ^1^H NMR of the purified QS-21 (**1**) preparation revealed the presence of a contaminant that turned out to be des-d-apiosyl-QS-21 (QS-21 lacking the d-apiosyl group of the C28 sugar chain) in a 1:1 ratio (based on ^1^H NMR integration). The full structure of the semipurified QS-21 (**1**) was resolved based on extensive high-resolution ESI–MS and 1D and 2D NMR data analysis, together with comparisons to data from the literature^26,27^ and a commercial QS-21 standard. The entire structure of the QS-21-apiose version was previously verified by the Gin group, including an enantioselective total chemical synthesis of the C_18_ arabinosylated acyl chain, which confirmed the proposed stereochemistry of the naturally isolated compound^27^. Preliminary comparison of the ^1^H NMR spectra recorded for purified QS-21 and the commercial standard (under identical conditions, methanol-*d*_4_, 600 MHz) showed complete superimposition, indicating the same connectivities and relative stereochemistry. Moreover, a careful inspection of the ^1^H NMR spectrum of the partially purified compound (1,000 scans recorded in methanol-*d*_4_ at 600 MHz; Supplementary Figs. 29 and 30, Supplementary Table 1 and Supplementary Data 6) revealed several diagnostic resonances, including the Fuc-H4 (recorded 5.11 ppm, m, *–*; standard 5.11 ppm, d, *J*_1,2_ = 3.2 Hz; literature^26^ 5.11 ppm, br d), which confirms the linkage of the C1 of the C_18_ acyl chain to the C4 position of the fucosyl moiety. An additional key resonance of the anomeric proton of l-arabinose at recorded 4.98 ppm (d, *J*_1,2_ = 2 Hz), standard 4.99 ppm (d, *J*_1,2_ = 1.8 Hz) and literature^26^ 4.98 ppm unambiguously confirmed the attachment of the l-arabinosyl moiety to the C5′ of the C_18_ acyl chain. These two key structural features show that we have achieved the complete biosynthesis of QS-21 in a heterologous host (Fig. 5b). Furthermore, the anomeric proton H1 of the C28 d-apiose was assigned at recorded 5.25 ppm (d, *J*_1,2_ = 2.7 Hz), standard 5.26 ppm (d, *J*_1,2_ = 2.9 Hz) and literature^26^ 5.25 ppm, which confirms that the generated molecule is QS-21 (d-Api*f* version; **1**; Supplementary Table 1). In summary, the recorded chemical shifts for the compound that we have produced and the Desert King standard (under identical conditions, methanol-*d*_4_, 600 MHz) are consistent with those reported in the literature for the naturally isolated compound^26^ (Supplementary Table 1 and Supplementary Data 6). The agreement of the spectra suggests the same relative and absolute configuration of the acyl chain. In particular, the resonances at the stereocenters (*δ*_H_ 4.02/acyl-3, 5.18/acyl-5, 1.61/acyl-6) were in full agreement with published data (*δ*_H_ 4.02, 5.19 and 1.61, respectively), supporting the expected (3*S*), (5*S*), (6*S*) absolute configuration of the C_9_ acyl unit (Extended Data Fig. 10). Collectively, these results unambiguously confirm the same connectivities and the same stereochemistry of the C_18_ arabinosylated acyl chain for the molecule that we have produced, the previously reported QS-21-apiose molecule generated by enantioselective total chemical synthesis and the naturally isolated compound from *Q. saponaria*.

## Discussion

QS-21 is a crucially important vaccine adjuvant that is currently sourced by extraction from the bark of the Chilean soapbark tree *Q. saponaria*. Its highly unusual and structurally complex glycosylated 18-carbon acyl chain is critical for immunostimulant activity. In this work, we have elucidated the biosynthetic route of this acyl chain and reconstituted the entire pathway for QS-21 synthesis in a heterologous expression system.

Our investigation shows that acyl chain biosynthesis originates from iterative decarboxylative condensations of malonyl-CoA with 2-MB-CoA (**4**), a catabolite of l-isoleucine. This reaction is catalyzed by members of the plant PKSIII family, which typically catalyze the formation of polyketides that undergo cyclization, releasing the CoA inherited from the substrate^13^. In the case of QS-21 acyl chain biosynthesis, this cyclization is a dead end, as the acyl unit needs to remain linear and be activated by a CoA to undergo transfer either directly to the glycosylated scaffold or to the C_9_ acyl unit already attached to the scaffold. *Q. saponaria* appears to have evolved a strategy to prevent this cyclization. It is possible that the biosynthetic enzymes are organized in a metabolon that stabilizes the extending C_9_ acyl unit, thereby preventing spontaneous lactonization. It is conceivable that the key role of KR1 may be one of stabilization rather than ketoreduction, a possibility that will be addressed in future work.

To summarize, we have successfully identified the enzymes necessary to reconstitute the entire QS-21 biosynthetic pathway in a heterologous host. This advance now opens up the opportunity to produce ‘free-from-tree’ QS-21 that does not depend on extraction from the bark of the soapbark tree. Our findings further open up unprecedented opportunities to engineer designer saponins with optimal immunostimulatory activity and low toxicity using metabolic engineering approaches, that is, a whole new phase of adjuvant discovery and development. Although QS-21 is a potent immunostimulant, it has a level of toxicity toward human cells. Our advances will enable the investigation of the poorly understood relationship between saponin structure and adjuvant activity, an area that is of keen interest for the development of vaccines of the future. To our knowledge, a transformation system is not currently available for *Q. saponaria*. However, in the future, the development of functional genomics tools for soapbark coupled with the understanding of the QS-21 biosynthetic pathway reported here and in Reed et al.^7^ could also enable optimization of the quantitative/qualitative saponin content of *Q. saponaria* plants and cell lines through manipulation of pathway regulation and flux using gene editing and other genetic modification strategies.

## Methods

### Natural product database mining

The occurrence of the QS-21 dimeric C_18_ acyl chain was investigated using two chemical databases, Reaxys and SciFinder (accessed on 4 July 2022). The dimeric C_18_ acyl chain termini were substituted with generic groups (R_1_ and R_2_ = H, carbon chain, carbocycle or cycle), and the search query was performed as drawn. In total, 80 and 77 compounds were recovered from Reaxys and SciFinder, respectively, following exclusion of all synthetic derivatives (Supplementary Data 2 and 3). A summary of the search outputs is shown in Supplementary Fig. 1.

### Genome mining, phylogenetic analysis, gene expression analysis and strategy used for candidate gene prioritization for shotgun transient plant expression

The genome assembly for *Q. saponaria* accession S10 and associated RNA-sequencing data are reported in Reed et al.^7^ (fully assembled and annotated *Q. saponaria* genome sequence, NCBI BioProject ID PRJNA914519; RNA-sequencing reads, NCBI BioProject ID PRJNA914309, SRA accession numbers SRR22829626–SRR22829649). Protein sequences for target genes were extracted from the *Q. saponaria* genome via InterPro or Pfam annotation generated by InterProScan output^29^ (CCL: IPR000873; PKSIII: IPR011141; short-chain dehydrogenase/reductase (SDR): PF00106, PF01073 and PF01370; BAHD: PF02458 and IPR003480). Unless otherwise stated, protein alignments were performed with MAFFT^30^ using the FFT-NS-I method with a maximum of 1,000 iterations. Phylogenetic trees were generated with RaxML^31^ using the PROTGAMMAAUTO model with 100 rapid bootstraps. SDRs were classified according to sdr-enzymes.org ref. ^32^.

For the discovery of candidate genes for the shotgun transient plant expression analysis, a scoring system for gene identification and prioritization was developed as follows. Transcript quantification and coexpression analysis were performed as described in Reed et al.^7^. Candidate gene scores were generated based on the strength of coexpression with *QsbAS1* (PCC value), absolute levels of transcript abundance in primordial tissue (transcripts per million (TPM) value) and the absence/presence of each gene in a putative BGC, as defined by a modified version of plantiSMASH^28^ (modifications detailed below), up to a maximum potential score of 3.0. Specifically, genes were given a score of 1 for a *QsbAS1* PCC value of 0.9 or greater or else were given a score of between 0 and 1 for a *QsbAS1* PCC value of between 0.8 and 0.9 on a linear scale. Genes were then given a score of 1 for a primordium TPM value of 3,000 or greater or else were given a score of between 0 and 1 for a primordium TPM value of between 1,000 and 3,000 on a linear scale. According to plantiSMASH output, genes were finally given a score of 1 if they formed part of a putative BGC with an already characterized saponin gene and otherwise were given a score of 0.5 if they formed part of any other putative BGC. These parameters were ascertained using those of the saponin genes characterized thus far. The sum of these scores was then used to give a final score, up to a maximum of 3.0. A list of the 68 top scoring genes is provided in Supplementary Data 1.

The modifications to plantiSMASH involved the inclusion of 50 additional pHMMs used to classify signature genes. These were based on hypothesized potentially relevant gene families for QS-21 biosynthesis that were not already included in plantiSMASH 1.0 or were gene families otherwise observed to be present in manual inspection of putative triterpene BGCs. The additional pHMMs were obtained from Pfam and are detailed in Supplementary Table 3. Output of this plantiSMASH analysis across the full genome with gene annotation data is provided in Supplementary Data 4.

### Cloning of *Q. saponaria* genes for functional analysis

Oligonucleotide primers were designed based on predicted gene sequences (Supplementary Table 4) and flanked with *attB* sites for Gateway cloning. RNA extracted from primordia was used for cDNA synthesis. The collected tissues were flash-frozen in liquid nitrogen and ground to a fine powder using a pestle and mortar. RNA extraction was performed using a Qiagen RNeasy Plant Mini kit with a modified protocol according to MacKenzie et al.^33^. Following cleanup of the purified RNAs, as per the protocol of the RNeasy Mini Handbook (Qiagen), RNA quality was assessed using Nanodrop ratios and gel electrophoresis. cDNA synthesis was performed using Superscript III (Thermo Fisher) with oligo(dT) primers according to the manufacturer’s instructions. Candidate genes were amplified from cDNA of primordia using iProof polymerase (Bio-Rad), cloned into pDONR207 using BP clonase (Thermo Fisher) and sequenced (Genewiz, Azenta Life Sciences) before being introduced into vectors for expression in *Escherichia coli*, yeast or *N. benthamiana* (see below).

### Analysis of carboxyl-CoA ligase activity

#### Preparation of cell extracts

*Q. saponaria* CCL candidate genes were cloned into the pAG423GAL-ccdB (*his3*Δ*1*/*his3*Δ*1*) vector using Gateway technology^34^ and introduced into the *Saccharomyces cerevisiae* strain Y21900 following a standard transformation protocol (YeastMaker, yeast transformation system 2, Clontech Laboratories). The hop genes *HlCCL2* and *HlCCL4* (ref. ^10^), with flanking Gateway sequences, were synthesized by Twist Bioscience according to their GenBank sequences (JQ740204.1 for *HlCCL2* and JQ740206.1 for *HlCCL4*). Yeast strains from fresh plates were cultured for 24 h at 30 °C in 20 ml of synthetic drop-out medium with d-galactose as the carbon source. The absorbances of the cultures were recorded for normalization of the data, and, after full-speed centrifugation, the resulting pellets were resuspended in 600 ml of quenching/extraction buffer (95% acetonitrile and 25 mM formic acid at −20 °C)^12^ and 300 ml of cold water. The cells were ground at full speed in a Spex 2010 Geno/Grinder for 15 min, and complete lysis was confirmed by light microscopy. After full-speed centrifugation, the supernatants were collected and freeze-dried overnight. The pellets were resuspended in 130 μl of resuspension buffer (25 mM ammonium formate (pH 3.0) and 2% methanol at 4 °C), filtered (0.2 μm, Spin-X, Costar) and transferred into glass vials with conical inserts for LC–MS analysis.

#### Acyl-CoA analysis

LC–ESI–MS/MS was performed on a Xevo TQ-S tandem quadrupole mass spectrometer (Waters) coupled to a UPLC system (Acquity). Multiple reaction monitoring transitions for standards of target acyl-CoAs were generated using IntelliStart software in both negative and positive ESI modes (Supplementary Tables 5 and 6). Positive mode turned out to be slightly more sensitive with a limit of detection of 50 fmol on column for IV-CoA. Separation of the target analytes was achieved on a reversed-phase C_18_ column (Kinetex XB-C_18_, 2.6 μm, 100 Å, 50 × 2.1 mm, Phenomenex) at 40 °C using a gradient of formic acid (50 mM, adjusted to pH 8.1 with 25% ammonium hydroxide in water; eluent A) and methanol (eluent B) at a flow rate of 300 μl min^–1^, according to Gläser et al.^12^. The following program was used: from 0 to 7 min using a linear gradient from 0 to 10% B, from 10 to 100% B over 3 min, hold at 100% B for 2 min, back to 0% B over 1 min and hold at 0% for 5 min. IV-CoA, isobutyryl-CoA, malonyl-CoA and CoA-SH were purchased from Sigma-Aldrich. 2-MB-CoA (**4**) was synthesized as described below.

#### Chemical synthesis of (*S*)-2-methylbutyryl-CoA (4)

(*S*)-2-MB-CoA (**4**) was prepared and purified using a procedure adapted from the literature^35,36^. CoA trilithium salt dihydrate (10 mg, 12.2 µmol) was added to a solution of saturated sodium bicarbonate in water (1 ml), and the solution was cooled to 0 °C. (*S*)-2-Methylbutyric anhydride (Sigma-Aldrich, 348562; 113.6 mg, 121.6 µl, 610 µmol) was then added, and the reaction mixture was stirred for 6 h at 0 °C. Reaction progress was followed by Ellman’s test (mix 80 µl of Ellman’s reagent 5,5′-dithiobis(2-nitrobenzoic acid), aqueous solution 1.8 mg ml^–1^, with 20 µl of the reaction mixture). The presence of thiols changes the color to yellow. Once all free thiol was reacted, HCl (1 M, ~920 µl) was added to adjust the pH to ~2. The solution was extracted with ethyl acetate (2 × 1 ml). The remaining aqueous solution was filtered through a 0.22-µm disc filter and kept frozen until further purification. The title product was purified by reversed-phase chromatography on a C_18_ column (Phenomenex, Gemini NX-C18 110 Å, axial compression, particle size of 5 μm, dimensions of 150 × 21.2 mm) using an HPLC (Dionex Ultimate 3000) with in-line UV detection. Elution was achieved using a gradient of methanol (solvent B) against 10 mM ammonium formate (pH 4.5; solvent A) at a flow rate of 20 ml min^–1^ 5% B for 2 min, a gradient to 50% B over 20 min, then to 95% B over 1 min, hold for 2 min, back to 5% B over 1 min and a final equilibration for 4 min with UV detection at 260 nm. Under these conditions, the title compound eluted at *R*_f_ = 19.0 min (CoA-SH elutes at *R*_f_ = 8.5 min). Pooled fractions containing (*S*)-2-MB-CoA were concentrated in vacuo to remove methanol, and the aqueous residue was freeze-dried to yield formate salt of (*S*)-2-MB-CoA (**4**; 7.2 mg, 66% isolated yield), specific rotation [*α*]_D_^20 °C^ = +0.6 (*c* = 1, water).

### Functional analysis of type III polyketide synthase candidates

#### Consumption of 2-methylbutyryl-CoA (4) following expression in yeast

Candidate *Q. saponaria* PKSIII enzymes and HlVPS^10^ (AB015430.1, synthesized by Twist Bioscience) were cloned into the vector pAG425GAL-ccdB (*leu2*Δ*0*/*leu2*Δ*0*), as described above. Yeast strain Y21900 was cotransformed with the hop *HlCCL4* (ref. ^10^) and each of the six PKSIII candidates individually. Preparation of cell extracts and LC–ESI–MS/MS were performed as described above.

#### Expression and purification of the *Q. saponaria* candidate type III polyketide synthase enzymes for in vitro assays

PKS1, PKS3, PKS4, PKS5 and PKS6 were expressed with a carboxy-terminal hexahistidine tag in *N. benthamiana* using *Agrobacterium*-infiltrated transient expression^17^. The His tag was added to the five PKSs by PCR using oligonucleotides encoding six histidine residues (Supplementary Table 7), and the amplified fragment was inserted into a unique NruI site of linearized pEAQ-HT vector^21^ by In-Fusion cloning (TaKaRa Bio/Clontech). PKS2 was cloned into the Gateway destination vector pEAQ-HT-DEST2 for expression with an amino-terminal hexahistidine tag^21^. The expression constructs were transformed into *A. tumefaciens* strain GV3101 and infiltrated into leaves of 3-week-old *N. benthamiana* plants^17^. After 6 d of incubation, 2 g of leaf material was ground in 10 ml of buffer (50 mM HEPES-KOH (pH 7.8), 330 mM sorbitol, 1% polyvinylpolypyrrolidone, 7 mM 2-mercaptoethanol and cOmplete EDTA-free protease inhibitor cocktail (Roche, 11 873 580 001)) on ice using a mortar and pestle. The homogenate was filtered through two layers of Miracloth (Calbiochem), centrifuged at 3,220*g* for 10 min to remove debris and centrifuged at 30,000*g* for 20 min to obtain cleared lysate without microsomes. The lysate (1.5 ml) was incubated with 50 μl of TALON metal affinity resin slurry (TaKaRa Bio/Clontech) in the presence of 5 mM imidazole and 0.1% (wt/vol) Triton X-100 for 2 h in a cold room with end-over-end mixing. The resin was washed four times with TBS-TX-Imi buffer (50 mM Tris-HCl (pH 7.5), 150 mM NaCl, 0.1% Triton X-100 and 5 mM imidazole) and once with buffer A4 (20 mM HEPES (pH 7.5) and 150 mM NaCl). His-tagged PKS protein was eluted twice with 250 μl of elution buffer (20 mM HEPES (pH 7.5), 150 mM NaCl and 150 mM imidazole). The eluant was subjected to two cycles of dilution in buffer A4 and concentration with Vivaspin 20 concentrators (50,000 molecular weight cutoff PES; Sartorius, VS2031) to minimize imidazole content. The concentration of PKS was adjusted to 0.5 mg of protein per ml. The cleared lysate and the purified PKS proteins were monitored by SDS–PAGE and Coomassie Brilliant Blue staining (Supplementary Fig. 7).

#### In vitro polyketide synthase assays

The substrates 2-MB-CoA (1 mM) and malonyl-CoA (2 mM) were mixed in phosphate buffer (100 mM, pH 7.0) in the presence of 1 mM tris(2-carboxyethyl)phosphine (TCEP) in a volume of 30 μl. Purified PKS enzyme was added at a final concentration of 0.1 mg of protein per ml, and the mixture was incubated at 25 °C for 150 min. After quenching with methanol (final concentration of 50%), the filtered reaction mixture (10 μl) was subjected to analytical HPLC (Dionex Ultimate 3000) equipped with an RP-C_18_ column (Kinetex XB-C_18_, 100 Å, particle size 5 μm, 100 × 4.6 mm, Phenomenex). Chromatography was performed using a gradient of solvent B (acetonitrile) against solvent A (10 mM ammonium formate, pH 4.5) as follows: 5% B for 2 min, gradient to 50% B over 20 min, 95% B over 1 min, hold for 2 min, back to 5% B over 1 min and equilibrate for 4 min at a flow rate of 1 ml min^–1^. Products were visualized by UV absorbance (260 nm).

#### Purification of (*S*)-6-*s*-butyl-4-hydroxy-2*H*-pyran-2-one (C_9_-δ-lactone)

Enzymatic transformation was performed in phosphate buffer (100 mM, pH 7.0) in a total volume of 6 ml. A mixture of PKS4–His and PKS5–His, coexpressed in *N. benthamiana* and freshly purified by metal affinity chromatography (0.7 mg of total protein, 900 µl), was added to a mixture containing malonyl-CoA (10 mg, final concentration of 2.0 mM), 2-MB-CoA (5.8 mg, final concentration of 1.0 mM) and TCEP (1.7 mg, final concentration of 1 mM). To enhance enzymatic transformation, the mixture was divided into 20 small tubes and incubated overnight at 25 °C. Progress of the enzymatic transformation was monitored by analytical HPLC as described above. Conversion after 14 h reached around 50%. The aliquots were combined and freeze-dried. The residue was dissolved in MilliQ water (1 ml) and methanol (1 ml), and the mixture was filtered through a PTFE disc filter (0.22 µm). The title compound was purified by preparative HPLC (Dionex Ultimate 3000) using an RP-C_18_ column (Gemini NX-C18 110 Å, Axial compression, particle size 5 μm, 150 × 21.2 mm, Phenomenex) with UV detection at 260 nm. Elution was achieved using a gradient of solvent B (acetonitrile) against solvent A (10 mM ammonium formate, pH 4.5). Elution started with 5% B for 2 min, gradient to 50% B over 20 min then to 95% B over 1 min, hold for 2 min, then back to 5% B over 1 min and finally equilibrate for 4 min at a flow rate of 20 ml min^–1^. Under these conditions, the title compound eluted at *R*_f_ = 15.1 min (CoA-SH eluted at *R*_f_ = 8.5 min). Pooled fractions were freeze-dried to yield the title compound (0.4 mg, 37% yield).

#### High-resolution mass spectrometry and tandem mass spectrometry analysis of the polyketide synthase product

For HRMS, the samples were diluted into 50% methanol/0.1% formic acid and infused into a Synapt G2-Si mass spectrometer (Waters) at 5–10 µl min^−1^ using a Harvard Apparatus syringe pump. The mass spectrometer was controlled by Masslynx 4.1 software (Waters), operated in resolution and positive ion mode and calibrated using sodium formate. The sample was analyzed for 1 min with 1 s of MS scan time over the range of 50–1,200 *m*/*z* with 3-kV capillary voltage, 40-V cone voltage and 120 °C cone temperature. Leu-enkephalin peptide (1 ng µl^−1^, Waters) was infused at 10 µl min^−1^ as a lock mass (*m*/*z* 556.2766) and was measured every 10 s. Spectra were generated in Masslynx 4.1 by combining several scans, and peaks were centered using automatic peak detection with lock mass correction. MS^2^ spectra of selected precursors were acquired directly via the tune page acquisition tab. The collision energy was ramped in steps of 5 between 25 and 40. Spectra were processed in Masslynx 4.1 by selecting the appropriate collision energy.

#### Nuclear magnetic resonance analysis of the polyketide synthase product

NMR spectra were recorded on a Bruker Avance III 400 MHz or Bruker Avance NEO 600 MHz with CryoProbe spectrometers. Chemical shifts of ^1^H NMR signals recorded in deuterium oxide were reported with respect to the residual solvent peak at *δ*_H_ 4.79 ppm or to the methyl resonance of internal acetone at *δ*_H_ 2.22 ppm. Chemical shifts of ^1^H NMR signals recorded in DMSO-*d*_6_ were reported with respect to the residual solvent peak at *δ*_H_ 2.50 ppm. Chemical shifts of ^13^C NMR signals recorded in deuterium oxide were reported with respect to the methyl carbon resonance of internal acetone at *δ*_C_ 30.89 ppm. Chemical shifts of ^13^C NMR signals recorded in DMSO-*d*_6_ were reported with respect to the residual solvent peak at *δ*_C_ 39.52 ppm. For samples recorded in methanol-*d*_4_, the chemical shifts are relative to the residual signal solvent (methanol-*d*_4_: *δ*_H_ 3.31 ppm; *δ*_C_ 49.15 ppm). ^31^P NMR spectra were recorded in deuterium oxide with ^1^H decoupling. Assignment of NMR spectra was aided by 2D experiments, including COSY, NOESY, HSQCed and HMBC. In ^13^C NMR spectra, the information on the multiplicity of carbon atom substitution with hydrogens (s = C, d = CH, t = CH_2_, q = CH_3_) was derived from HSQCed experiments. High-resolution accurate mass spectra were obtained using a Synapt G2-Si Q-TOF mass spectrometer using negative electrospray ionization. HPLC purification was performed on a Dionex Ultimate 3000 instrument equipped with a UV/Vis detector. Freeze drying was performed on a Labconco FreeZone Benchtop Freeze Dryer with a PTFE Coil. An Eppendorf 5810R benchtop centrifuge was used for centrifugation.

### QS-21 pathway reconstitution in *N. benthamiana*

Transient expression of candidate genes in *N. benthamiana* was performed as follows. A total of 68 candidate genes prioritized based on the criteria (Supplementary Data 1) were cloned into the binary expression vector pEAQ-HT*-*DEST1 (ref. ^21^). The expression constructs were transformed into *A. tumefaciens* strains LBA4404 or GV3101. For ease of performing infiltrations, in some cases, multiple genes incorporated into a single binary vector using Golden Gate cloning were used^37,38^. The coding sequence of each gene was domesticated by removal of BpiI and/or BsaI restriction sites as needed and assembled into the Golden Gate entry vector pL0-pICH41308. Genes were further assembled into level 1 expression cassettes consisting of the flanking modified 5′ and 3′ untranslated regions from cowpea mosaic virus^21^ under control of the CaMV35S promoter and Nos terminator. To enhance the expression of recombinant proteins in *N. benthamiana*, the P19 viral suppressor of gene silencing was also assembled under the control of the CaMV35S promoter and CaMV35S terminator. Finally, multiple genes were incorporated into level 2 and/or a set of level M binary expression vectors and transformed into *A. tumefaciens* strain LBA4404 or GV3101. The Golden Gate constructs were used interchangeably with the pEAQ constructs. The constructs used for the production of QA-TriX-FRXX (**8**) are reported in Reed et al.^7^. Additional constructs generated for the pathway genes reported in this study are shown in Supplementary Figs. 31 and 32.

*N. benthamiana* plants were maintained under greenhouse conditions, as described previously^7^. For screening of candidate genes, agroinfiltrations were performed using a needleless syringe^7,39^. For pooled *Agrobacterium* infiltrations, appropriate volumes of each strain suspension were mixed together to reach the same final concentration for each strain. Leaf material was collected 5 d after infiltration and frozen at –70 °C before lyophilization for 24–72 h. In addition to the *Q. saponaria* QS-21 pathway genes characterized in Reed et al.^7^ and in this study, all experiments included coexpression of the truncated feedback-insensitive mevalonate pathway enzyme 3-hydroxy-3-methylglutaryl-CoA reductase to boost triterpene yield^40^.

### Preparation of *Q. saponaria* and *N. benthamiana* leaf extracts for liquid chromatography–mass spectrometry analysis

Freeze-dried plant material (10–15 mg per sample) was disrupted with 3-mm tungsten carbide beads (Qiagen) at 1,000 r.p.m. for 1 min (Geno/Grinder 2010, Spex SamplePrep). Metabolites were extracted in 600 µl of 80% methanol, and 4 μg of internal standard was added (digitoxin, Sigma-Aldrich). The samples were incubated for 1 h at 70 °C with shaking at 1,000 r.p.m. (Thermomixer Comfort, Eppendorf). Each sample supernatant was defatted by partitioning once with 400 µl of hexane. The lower aqueous phase was dried under vacuum at 45 °C for 1.5 h (EZ-2 Series Evaporator, Genevac). Dried material was resuspended in 130 µl of 80% methanol, filtered at 12,500*g* for 30 s (0.2 μm; Spin-X, Costar) and used for LC–MS analysis.

### High-performance liquid chromatography–electrospray ionization–mass spectrometry analysis of leaf extracts

Analysis was performed using a Thermo Scientific QExactive Hybrid Quadrupole-Orbitrap mass spectrometer HPLC system calibrated using Pierce positive/negative calibration standards according to the manufacturer’s instructions. Detection was performed using the following parameters: MS (ESI ionization), scan range of 400–2,500 *m*/*z* in negative mode, 70,000 resolution, data-dependent MS^2^, isolation window of 4.0 *m*/*z*, collision energy of 30, resolution of 17,500 and dynamic exclusion of 5.0 s. Solvent A consisted of water + 0.1% formic acid, and solvent B consisted of acetonitrile. The injection volume was 10 µl, and the following gradient was used: 15% B from 0 to 0.75 min, 15 to 60% B from 0.75 to 13 min, 60 to 100% B from 13 to 13.25 min, 100 to 15% B from 13.25 to 14.5 min and 15% B from 14.5 to 16.5 min. The method was performed using a flow rate of 0.6 ml min^−1^ and a Kinetex column (2.6 μm XB-C_18_, 100 Å, 50 × 2.1 mm (Phenomenex)) maintained at 40 °C. The analysis was performed using Xcalibur and FreeStyle software (Thermo Scientific). A QS-21 standard obtained from Desert King was used as a control.

The measurement of δ-lactone and monoreduced δ-lactone was performed as described above with the following changes: a scan range of 75 to 1,125 *m*/*z* in positive mode, collision energy of 55 and 60 and dynamic exclusion of 3.0 s. The LC gradient with the same solvents as described above was 5% B from 0 to 1.5 min, 5 to 50% B from 1.5 to 11.5 min, 50 to 95% B from 11.5 to 12.2 min, 95% B from 12.2 to 13.6 min, 95 to 5% B from 13.6 to 14.3 min and 5% B from 14.3 to 16.5 min.

### Investigation of the activity of the UGT73CZ2 sugar transferase in vitro

#### Generation of purified UGT73CZ2

UGT73CZ2 was expressed with a carboxy-terminal hexahistidine tag in *N. benthamiana* by agroinfiltration as described for the PKSs (see above). Oligonucleotide sequences are listed in Supplementary Table 4. The purity of UGT73CZ2 was monitored by SDS–PAGE and Coomassie Brilliant Blue staining.

#### Purification of the des-arabinosyl-QS-21 acceptor (11)

One gram of commercially available *Q. saponaria* (Sigma-Aldrich) bark was solubilized in methanol/water (80/20 (vol/vol)) and directly subjected to Biotage C18–60 g reversed-phase flash column chromatography using a long gradient of water/acetonitrile + 0.1% formic acid (90/10 → 30/70) for 60 min at 50 ml min^–1^. Fractions were monitored by LC–MS. A fraction containing QS-17, QS-18 and QS-21 along with des-arabinosyl QS-21 was subjected to further repetitive fractionation using an Agilent semipreparative HPLC (in isocratic mode, water/acetonitrile + 0.1% formic acid (55/45) for 30 min at 4 ml min^–1^; Luna 5 m C_18_(2), 250 × 10 mm). A peak corresponding to the des-arabinosyl form of QS-21 was collected and dried to yield 3.5 mg of purified product. This was confirmed to be the des-arabinosylated form of QS-21 (d-apiose form; **1**) by HRMS and extensive 1D and 2D NMR analysis (Supplementary Data 5). This compound (**11**) was used as the acceptor in assays of UGT73CZ2 activity (see below).

#### UGT73CZ2 enzyme assays

The reaction mixture was composed of 50 mM HEPES-KOH (pH 7.5), 2 mM MgCl_2_, 0.3% 2-mercaptoethanol, 0.1 mM des-arabinosyl-QS-21 (QA-TriX-FRXA-C_18_) and 0.5 mM of each UDP sugar in a final volume of 50 μl. Reactions were initiated by the addition of 0.8 μg of purified UGT73CZ2 to the reaction mixture and incubation at 25 °C for 14 h. After quenching with methanol (final concentration of 50%), the filtered reaction mixture (10 μl) was analyzed with a QExactive Hybrid Quadrupole-Orbitrap mass spectrometer (Thermo Scientific) equipped with a Charged Aerosol Detector (Thermo Scientific) and an RP-C_18_ column (Kinetex XB-C_18_, 100 Å, particle size 2.6 μm, 50 × 2.1 mm, Phenomenex). UDP-β-l-arabinofuranose was obtained from Peptide Institute (Japan), UDP-α-d-glucose and UDP-α-d-galactose were from Sigma-Aldrich, and UDP-α-d-xylose and UDP-β-l-rhamnose were from Carbosynth (Switzerland). UDP-β-l-arabinopyranose and UDP-α-d-fucose were prepared following published procedures^41^.

### Cloning and mutagenesis of QsTD

The *Q. saponaria QsTD* gene (*Qs0222940*) was cloned by PCR from leaf cDNA using primers as detailed in Supplementary Table 4 and was inserted into pDONR207. Once the clone had been verified by Sanger sequencing (Eurofins Genomics), the relevant P540L mutant was generated by using a Q5 site-directed mutagenesis kit (New England Biolabs) using the primers as detailed in Supplementary Table 4 and according to the manufacturer’s instructions. This mutant was again verified by sequencing. Both wild-type and mutant *QsTD* were inserted into the binary expression vector pEAQ-HT*-*DEST1 and transformed into *Agrobacterium* LBA4404.

### Free amino acid extraction and profiling

*A. tumefaciens* cells carrying QsTD, QsTD-P540L mutant or green fluorescent protein in pEAQ-HT*-*DEST1 were infiltrated into the leaves of 5-week-old *N. benthamiana* plants. Four leaves were used for each condition as biological replicates. After 3 d, leaves were collected, flash-frozen in liquid nitrogen and lyophilized. For the extraction of free amino acids, 20 mg of dry leaf material was ground with 3-mm tungsten beads (Qiagen) using a Spex Geno/Grinder at 1,000 r.p.m. for 30 s. To the dry leaf powder, 120 µl of a buffer containing 20 mM HEPES (pH 7.0), 5 mM EDTA and 10 mM NaF was added, followed by 500 µl of chloroform:methanol (3:7 (vol/vol)). The sample was vortexed and kept on ice for 30 min before the addition of 600 µl of water. The samples were centrifuged for 10 min at 14,000*g* to separate the mixture into two phases. The upper aqueous methanol phase was transferred to a fresh tube, and the majority of the methanol was removed from the sample by centrifugation under vacuum at 40 °C for 1 h (Genevac). The remaining extract was frozen in a bath of ethanol on dry ice and finally lyophilized to dryness. Each sample was resuspended in 100 µl of water, filtered with Spin-X filter columns (0.22 μm, nylon; Costar) and further diluted 100-fold. Ten microliters of this dilution was derivatized using 20 µl of reconstituted AccQ-Fluor reagent (Waters) and 70 µl of borate buffer vortexed and heated at 55 °C for 10 min. Standards of amino acids were prepared using an AccQ-Fluor Reagent kit (Waters) according to manufacturer’s instructions.

Amino acid profiling was performed using a Xevo TQ-S tandem quadrupole mass spectrometer (Waters) coupled to a UPLC system (Acquity). The source temperature was set to 151 °C, and the desolvation temperature was set to 345 °C. Cone gas flow was 50 ml min^–1^, the desolvation gas flow was 33.3 ml min^–1^, and the collision gas flow was 0.14 ml min^–1^. Multiple reaction monitoring transitions for standards of target amino acids were generated using IntelliStart software in positive ESI mode (Supplementary Table 8). The collision energy was 30 V. Two microliters of each sample was injected for analysis. Separation of target analytes was achieved using a Kinetics XB-C_18_ column (100 × 2.1 mm, 2.6 μm, 100 Å; Phenomenex) with a solvent system of 0.1% formic acid in water (solvent A) and acetonitrile (solvent B). The LC program was set to 1% B for 1 min before increasing to 20% B until 15.5 min, then from 20 to 90% B until 17.5 min before returning to 1% B at 18 min. The column was held at 1% B until 20 min.

### Purification and structural determination of QS-21 produced in *N. benthamiana*

Three hundred *N. benthamiana* plants were vacuum infiltrated as described in Stephenson et al.^42^ with equal amounts of the *A. tumefaciens* strains containing the genes required to make QA-TriX-FRXA^7^ (the C28 d-apiose variant of the QS-21 pathway intermediate) and 3-hydroxy-3-methylglutaryl-CoA reductase, TD-P540L, CCL1, PKS1–PKS6, KR1, KR2, ACT2, ACT3 and UGT73CZ2. After 5 d, leaves were collected, freeze-dried and prepared as described in Stephenson et al.^42^ for pressurized solvent extraction. The leaves were first defatted with hexane in the pressurized solvent extraction instrument (0 min hold time), and the extracts resulting from two cycles of 100% methanol (0 min hold time and then 5 min hold time at 100 °C) were pooled. The extract was dried on celite, and flash chromatography (5 to 100% acetonitrile, flow rate of 50 ml min^–1^, 1,312 ml) was used as a first fractionation step. The fraction containing QS-21 was further purified using an Agilent 1260 prep LC–MS with water + 0.1% formic acid (solvent A) and acetonitrile (solvent B) using the following method: from 0 to 2 min, 15 to 40% B; from 2 to 34 min, 40 to 60% B (QS-21 elutes around 50% B); from 34 to 34.5 min, 60 to 100% B; hold for 3.5 min and return to 15% B in 30 s (flow rate of 25 ml min^–1^ on a Luna 5-mm C_18_(2) 100-Å LC column 250 × 21.2 mm). The fractions containing QS-21 were further purified using an Agilent 1290 UHPLC with the same chromatography method used for the Agilent 1260 prep LC–MS but with a shallower gradient from 44 to 50% B from 2 to 34 min and with a Luna 5-mm C_18_(2) 100-Å column (250 × 10 mm at 4 ml min^–1^).

### Nuclear magnetic resonance analysis of QS-21

One-dimensional and 2D NMR spectra were recorded on a Bruker Avance 600 MHz spectrometer equipped with a BBFO Plus Smart probe and a triple resonance TCI cryoprobe, respectively (John Innes Centre (JIC)). The chemical shifts are relative to the residual signal solvent (methanol-*d*_4_: *δ*_H_ 3.31 ppm; *δ*_C_ 49.15 ppm). Spectra for QS-21 produced in *N. benthamiana* and a QS-21 commercial standard (Desert King) were compared to the data reported for QS-21 in the literature^22,27,43,44^.

### Reporting summary

Further information on research design is available in the Nature Portfolio Reporting Summary linked to this article.

## Online content

Any methods, additional references, Nature Portfolio reporting summaries, source data, extended data, supplementary information, acknowledgements, peer review information; details of author contributions and competing interests; and statements of data and code availability are available at 10.1038/s41589-023-01538-5.

### Supplementary information

Supplementary Information Supplementary Figs. 1–32, Tables 1–8 and source data for Supplementary Fig. 7.

Reporting Summary

Supplementary Data 1, 2 and 4 Supplementary Data 1. New candidate genes prioritized for functional analysis in the shotgun experiment. Supplementary Data 2. Reaxys search output. Supplementary Data 4. plantiSMASH output.

Supplementary Data 3, 5 and 6 Supplementary Data 3. CAS SciFinder search output. Supplementary Data 5. Confirmation of the structure of des-arabinosyl QS-21 (d-apiose; **11**; used in UGT73CZ2 assays). Supplementary Data 6. Spectral confirmation of QS-21/des-apiosyl-QS-21 produced and purified from *N. benthamiana*.

Supplementary Data 7 Statistical source data for Supplementary Figs. 5, 14, 15 and 28.

### Source data

Source Data Table 1, Figs. 1c and 2b and Extended Figs. 3, 8 and 9 Statistical source data.

## Data Availability

Transcriptome and genome sequence data for *Q. saponaria* were previously reported in Reed et al.^7^ submitted under NCBI BioProject IDs PRJNA914309 (SRA accession numbers SRR22829626–SRR22829649) and PRJNA914519. The sequences of the following genes characterized in the current study have been deposited in GenBank: *CCL1* (*Qs0229930*), OQ241430; *CCL2* (*Qs0216480*), OQ241421; *PKS1* (*Qs0007680*), OQ241431; PKS2 (*Qs0170050*), OQ241424; *PKS3* (*Qs0181340*), OQ241419; PKS4 (*Qs0268330*), OQ241427; *PKS5* (*Qs0268880*), OQ241428; *PKS6* (*Qs0285490*), OQ241422; *KR1* (*Qs0326850*), OQ241429; *KR2* (*Qs0235370*), OQ241432; *ACT2* (*Qs0322030*), OQ241420; *ACT3* (*Qs0264740*), OQ241426; *UGT73CZ2* (*Qs0131010*), OQ241425; *TD* (*Qs0222940*), OQ241423; feedback-insensitive TD (TD-P540L; *Qs0222940_P540L*), OQ241433. The databases used in this study were SciFinder (scifinder.cas.org), Reaxys.com, sdr-enzymes.org, InterPro-85.0 (https://www.ebi.ac.uk/interpro/) and Pfam-33.1 (http://pfam.xfam.org/). Source data are provided with this paper.
