# Supplementary material for: Complete biosynthesis of the potent vaccine adjuvant QS-21
Source: Nat Chem Biol. 2024 Jan 26;20(4):493–502. doi: 10.1038/s41589-023-01538-5 (PMC10972754; doi:10.1038/s41589-023-01538-5)
Supplement: Supplementary file 1 — Supplementary Figs. 1–32, Tables 1–8 and source data for Supplementary Fig. 7. [file 41589_2023_1538_MOESM1_ESM.pdf]

nature chemical biology

Article

https://doi.org/10.1038/s41589-023-01538-5

Complete biosynthesis of the potent vaccine adjuvant QS-21

In the format provided by the authors and unedited

Table of Contents

Supplementary Fig. 1. Mining the Reaxys and SciFinder chemical databases for structures containing the dimeric C_{18} acyl chain recovered saponins from Quillaja species only 3
Supplementary Fig 2. Predicted CCLs from *Q. saponaria*. 4
Supplementary Fig. 3. Co-expression of *Q. saponaria* CCL candidates with saponin biosynthetic genes. DeSeq normalised read counts (TPM) for primordium tissue and co-expression PCC values for each of the candidate CCL genes (Supplementary Fig. 2) with the biosynthetic genes elucidated in[7] and this publication are shown. 5
Supplementary Fig. 4. Chemical synthesis of (*S*)-2-methylbutyryl-CoA (4)..................................6
Supplementary Fig. 5. Production of the short-chain acyl CoA isobutyryl-CoA by Q. saponaria CCLs expressed in yeast...7
Supplementary Fig 6. Predicted PKSIII genes from *Q. saponaria* 8
Supplementary Fig. 7. Heterologous expression in *N. benthamiana* and purification of His-tagged *Q. saponaria* PKS enzymes...9
Supplementary Fig. 8. *Q. saponaria* PKS4 converts 2-methylbutyryl-CoA (4) to a new product, X 10
Supplementary Fig. 9. Characterization of the PKS product X by mass spectrometry 11
Supplementary Fig. 10. (S)-6-sec-butyl-4-hydroxy-2H-pyran-2-one (C_9 δ-lactone) with carbon atom numbering. Selected key interactions in 2D NMR spectra (HMBC and NOESY)................12
Supplementary Fig. 11. NMR spectra for the C_9-δ-lactone 13
Supplementary Fig. 12. *In vitro* activity assay comparing the six purified *Q. saponaria* PKS enzymes 15
Supplementary Fig. 13. MS/MS analysis of *in vitro* products of the six PKS enzymes................16
Supplementary Fig. 14. Detection and quantification of (*S*)-6-sec-butyl-4-hydroxy-2H-pyran-2-one (C_9-δ-lactone) (7) following *Agrobacterium*-mediated co-expression of CCL1 with each of the six *Q. saponaria* PKSs in leaves of *N. benthamiana* 17
Supplementary Fig. 15. Formation of the C_9-δ-lactone and the monoreduced C_9-δ-lactone in *N. benthamiana* and *Q. saponaria* 18
Supplementary Fig. 16. Co-expression of the genes for biosynthesis of QA-TriX-FRXX (8) and the acyl donor (6) with the pool of candidate genes for addition of the acyl moiety results in production of a compound identical to a standard of QS-21...19
Supplementary Fig. 17. Phylogeny of *Q. saponaria* SDR enzymes 20
Supplementary Fig. 18. Functional analysis of the ACT candidates 21
Supplementary Fig. 19. Further analysis of the ACT candidates – biosynthesis of QA-TriX-FRXX-C_{18}...22
Supplementary Fig. 20. Screening for the reductase modifications on QA-TriX-FRXX-C_9..........23
Supplementary Fig. 21. Screening for the reductase modifications on QA-TriX-FRXX-C_{18} 24
Supplementary Fig. 22. Functional analysis of the KR candidates (QA-TriX-FRXX-C_9)............25
Supplementary Fig. 23. Functional analysis of the KR candidates (QA-TriX-FRXX-C_{18})...........26
Supplementary Fig. 24. Functional analysis of the arabinofuranosyl transferase candidate UGT73CZ2..27
Supplementary Fig. 25. UDP sugar donor specificity of UGT73CZ2 28
Supplementary Fig. 26. Detection of QS-21 and selected pathway intermediates in extracts from *N. benthamiana* leaves expressing QS-21 biosynthetic genes 29
Supplementary Fig. 27. Predicted subcellular localization of saponin biosynthetic genes from *Q. saponaria* 30
Supplementary Fig. 28. Effect of 2-methylbutyric acid supplementation on QS-21 yield 31

Supplementary Fig. 29. Full 1H NMR spectral comparison between the QS-21 preparation generated by large-scale agro-infiltration of N. benthamiana (blue) and a QS-21 standard (red) ..32
Supplementary Fig. 30. Expanded 1H-NMR spectral comparisons between a commercial QS-21 standard and the partially purified QS-21 produced in *N. benthamiana* 33
Supplementary Fig. 31. Plasmid map of Golden Gate vector EC90023_pLM-1-FAC1 34
Supplementary Fig. 32. Plasmid map of Golden Gate vector EC90024_pLM-5-FAC2 35
Supplementary tables 36
Supplementary Table 1. 1H, 13C-NMR spectroscopic data comparison (key resonances, retrieved based on HSQC) for the QS-21 standard and the product produced in *N. benthamiana* with the data reported for QS-21 in the literature 36
Supplementary Table 2. LC/MS-MS information for the three saponins described in this work...37
Supplementary Table 3. Additional pHHMs used in plantiSMASH analysis of *Q. saponaria* genome 38
Supplementary Table 4. Primer sequences used to clone the candidate genes for yeast and *N. benthamiana* expression 39
Supplementary Table 5. MRM transitions of acyl-CoA standards in ESI positive mode...............40
Supplementary Table 6. MRM transitions of acyl-CoA standards in ESI negative mode.............41
Supplementary Table 7. Primer sequences used to make His-tagged proteins for *in vitro* analysis 42
Supplementary Table 8. MRM transitions of amino acid standards in positive ESI mode 43
Source Data Supplementary Fig 7 44
Supplementary Information References 45

a

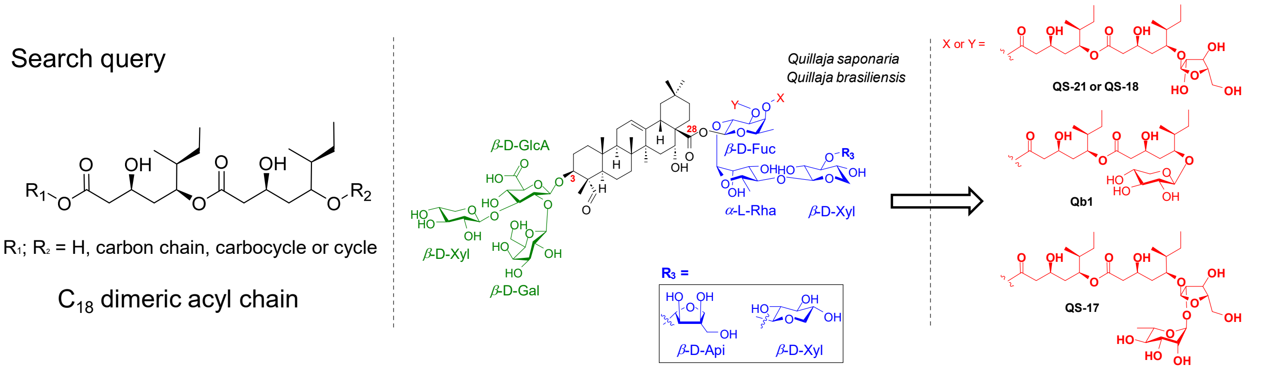

b

Saponin	Reaxys		SciFinder	
	Number of hits	Position of acyl chain	Number of hits	Position of acyl chain
QS-21	22<br>7	X<br>Y	8<br>7	X<br>Y
QS-17	1	Y	3	X
QS-18	3<br>1	X<br>Y	2<br>1	X<br>Y
QB1	1	X	1	X
Other *Quillaja* saponins	16<br>10	X<br>Y	36<br>8	X<br>Y

Supplementary Fig. 1. Mining the Reaxys and SciFinder chemical databases for structures containing the dimeric C_{18} acyl chain recovered saponins from Quillaja species only. (a) Search query and predominant found structures. R1 and R2 were specified as being a H, a carbon chain, a carbocycle or a cycle in the SciFinder search. (**b**) Summary of the search outputs. The majority of the retrieved compounds had a C_{18} dimeric acyl chain attached to the saponin scaffold via the C-4 (X) position of D-fucose, although examples in which linkage occurred via the C-3 (Y) position of this sugar were also found. Other *Quillaja* saponins harbor different glycosylation and/or oxidation patterns of the triterpene backbone than those of QS-21, QS-17, QS-18 and QB1. Regiomigration of the acyl chain of QS-21 between the C3 and C4 positions is known to occur in basic solution[27,43,44,47]. See Supplementary Datasets 2 and 3 for further information.

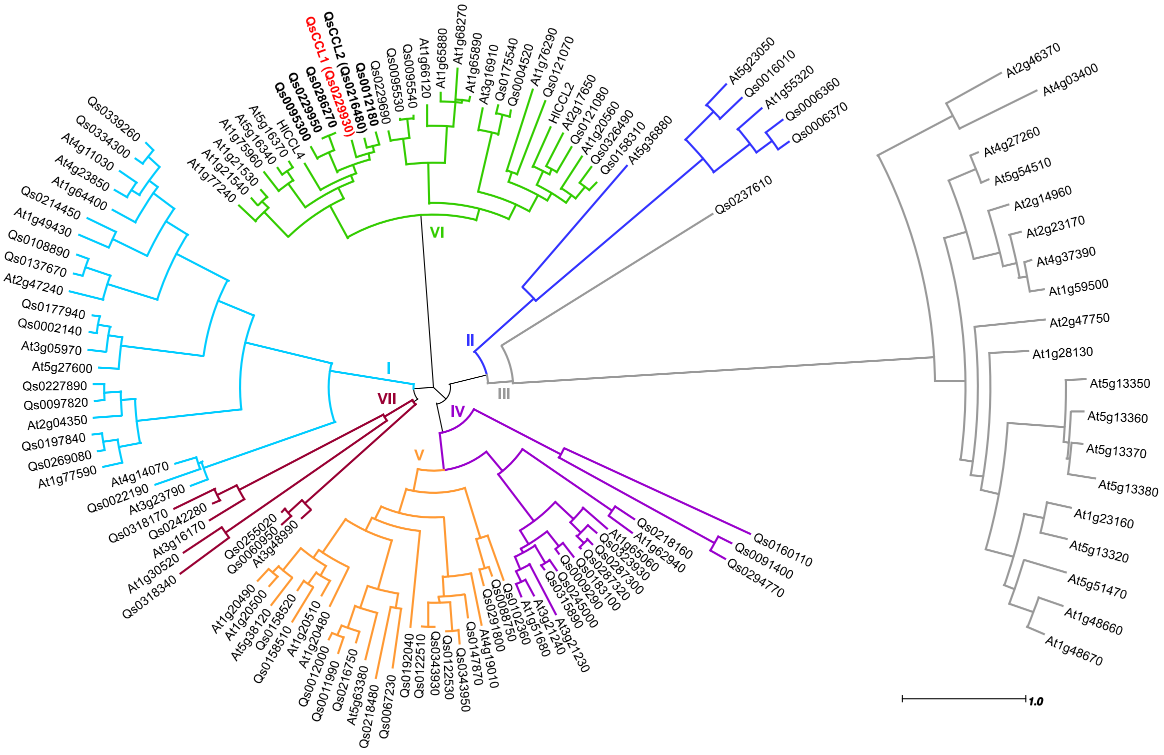

Supplementary Fig 2. Predicted CCLs from *Q. saponaria*. The presence of Interpro domain IPR000873 (AMP-dependent synthetase/ligase) was used to mine 63 genes from the *Q. saponaria* genome. These were aligned with 63 *A. thaliana* genes from[47] and the two CCL genes HlCCL2 (M4IRL4.1) and HlCCL4 (M4IQQ5.1) isolated from *Humulus lupus*[10]. Seven clades are labelled according to the classification of[47]. The gene highlighted in red was most highly co-expressed with *QsbAS1* (*QsCCL1; Qs0229930*; PCC ≥ 0.99) and was also the most highly expressed CCL gene in the primordium (Supplementary Fig. 3). This gene and five additional closely related genes (indicated in bold) were also cloned and tested for function. See Materials and Methods for methods used for phylogenetic analysis.

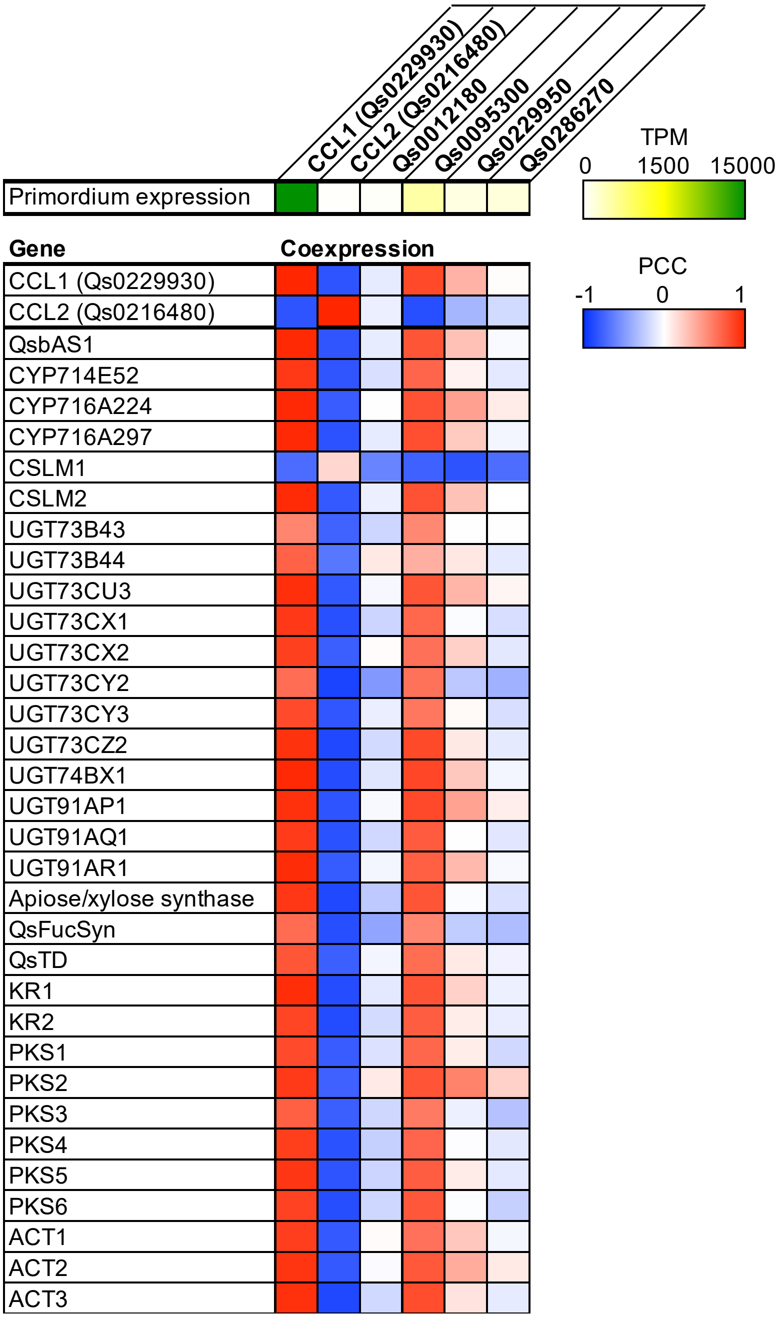

Supplementary Fig. 3. Co-expression of *Q. saponaria* CCL candidates with saponin biosynthetic genes. DeSeq normalised read counts (TPM) for primordium tissue and co-expression PCC values for each of the candidate CCL genes (Supplementary Fig. 2) with the biosynthetic genes elucidated in[7] and this publication are shown.

a Chemical synthesis of (*S*)-2-methylbutyryl-CoA **(4)**

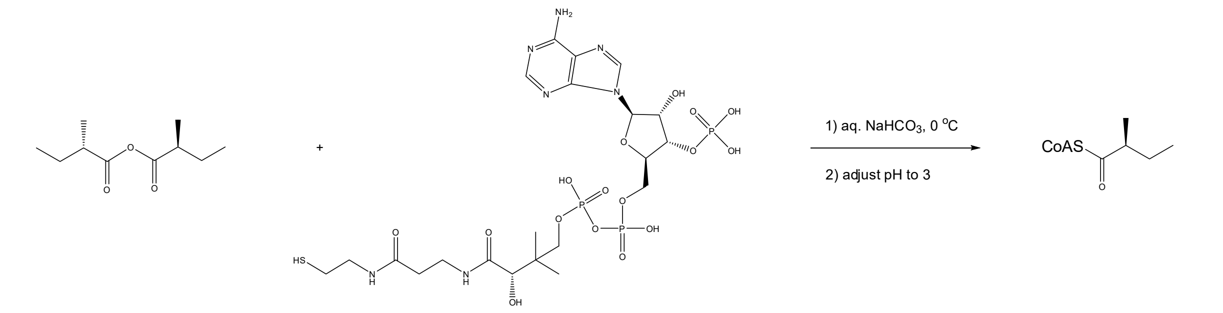

(*S*)-2-methylbutyric anhydride **CoASH** **(*S*)-2-methylbutyryl-CoA**

b Confirmation of structure by NMR

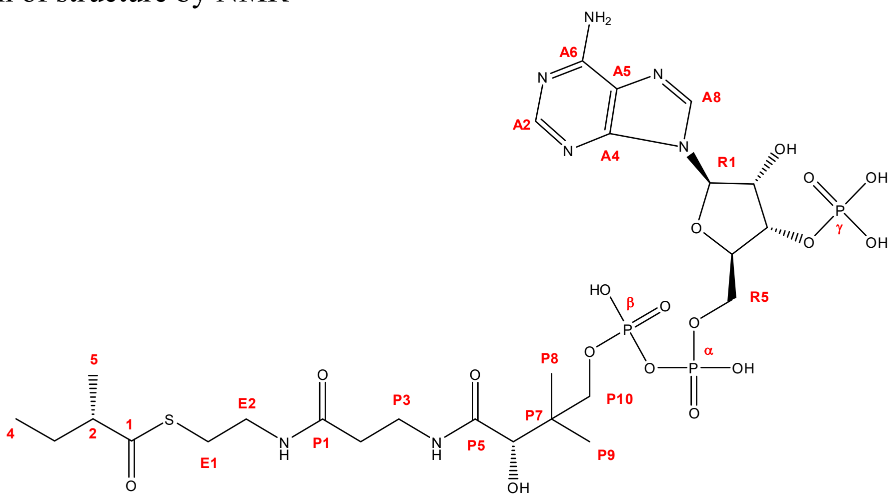

Supplementary Fig. 4. Chemical synthesis of (*S*)-2-methylbutyryl-CoA (4). (**a**) Scheme for chemical synthesis. (**b**) Atom numbering of (**4**) for NMR. ^{1}H NMR (400 MHz, D_2O) δ_H 8.55 (s, 1H, A8), 8.46 (s, 1H, A1 NH^+), 8.27 (s, 1H, A2), 6.18 (d, J = 6.3 Hz, 1H, R1), 4.88-4.79 (m, 2H, R3 and R4 overlapped with HDO peak), 4.59 (bs, 1H, R4), 4.25 (bs, 2H, R5), 4.02 (s, 1H, P6), 3.84 (bd, J = 6.5 Hz, 1H, P10a), 3.57 (bd, J = 6.5 Hz, 1H, P10b), 3.51-3.40 (m, 2H, P3), 3.33 (t, J = 6.3 Hz, 2H, E2), 2.99 (t, J = 6.3 Hz, 2H, E1), 2.68-2.59 (m, 1H, 2), 2.43 (t, J = 6.6 Hz, 2H, P2), 1.66-1.55 (m, 1H, 3a), 1.50-1.40 (m, 1H, 3b), 1.10 (d, J = 6.9 Hz, 3H, 5), 0.90 (s, 3H, P8 or P9), 0.84 (t, J = 7.4 Hz, 3H, 4), 0.77 (s, 3H, P8 or P9). ^{13}C NMR (100 MHz, D_2O) δ_C 209.7 (s, 1C, 1), 175.3 and 174.5 (2s, 2 C, P1 and P5), 171.7 (d, 1C, HCOO), 156.2 (s, 1C, A6), 153.5 (d, 1C, A2), 150.0 (s, 1C, A4), 140.4 (d, 1C, A8), 119.2 (s, 1C, A5), 87.1 (d, 1C, R1), 84.4 (ddd, $^3J_{R4,P\alpha}$ = 8.79 Hz, $^3J_{R4,P\gamma}$ = 4.45 Hz, 1C, R4), 74.8 (dd, $^3J_{R2,P\gamma}$ = 3.16 Hz, 1C, R2), 74.7 (d, 1C, P6), 74.4 (dd, $^2J_{R3,P\gamma}$ = 4.72 Hz, 1C, R3), 72.5 (dt, $^2J_{P10,P\beta}$ = 6.21 Hz, 1C, P10), 66.2 (dt, $^2J_{R5,P\alpha}$ = 4.95 Hz, 1C, R5), 50.5 (d, 1C, 2), 39.3 (t, 1C, E2), 38.9 (sd, $^3J_{P7,P\beta}$ = 8.04 Hz, 1C, P7), 36.0 (2t, 2C, P2 and P3), 28.4 (t, 1C, E1), 27.5 (t, 1C, 3), 21.5 and 18.8 (2q, 2C, P8 and P9), 17.2 (q, 1C, 5), 11.4 (q, 1C, 4). ^{31}P{^{1}H} NMR (162 MHz, D_2O) δ_P 1.7 (bs, Pγ), -10.8 and -11.3 (2bd, Pα and Pβ). HR-MS (ESI$^-$) for $C_{26}H_{43}N_7O_{17}P_3S^-$ m/z calcd.: 850.1654 found: 850.1652 [M-H]$^-$.

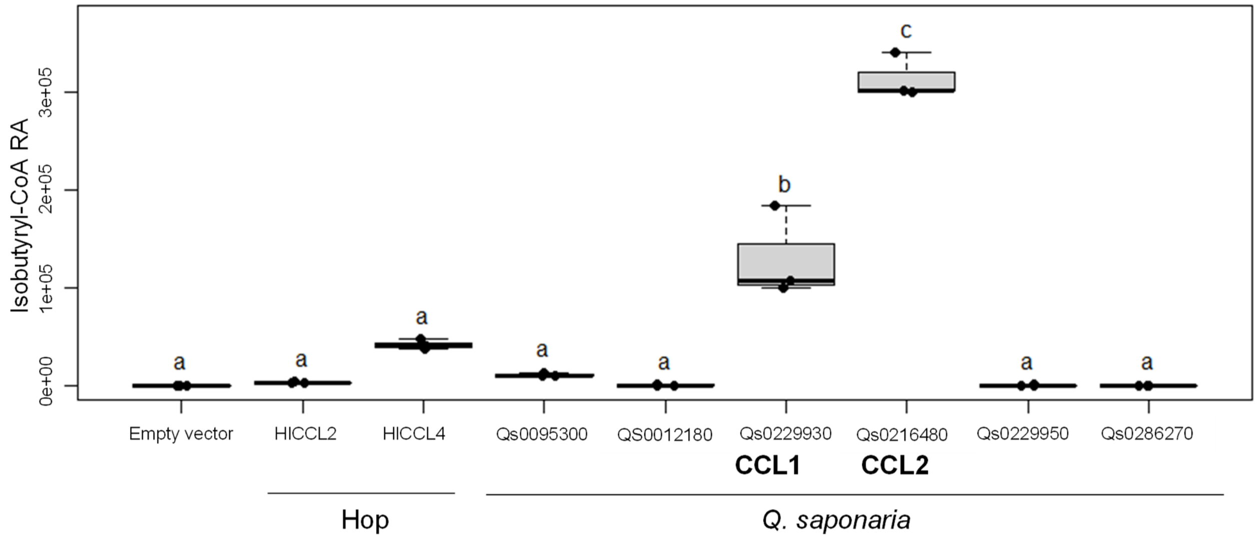

Supplementary Fig. 5. Production of the short-chain acyl CoA isobutyryl-CoA by Q. saponaria CCLs expressed in yeast. HlCCL2 and HlCCL4, hop CCLs that produce isovaleryl-CoA and 2-methylbutyryl-CoA (**4**), respectively[10]; clade VI *Q. saponaria* CCLs (Supplementary Fig. 2). RA, relative amounts. Letters represent significantly different data as determined by the two-sided post-hoc Tukey's HSD (p = 0.05) after ANOVA (Df = 8, p Value = $9.87E^{-14}$) using the multcompView package in R. The boxplots show the distributions of the values for three independent yeast cultures per treatment (represented by the dots), the centre line representing the median, the box showing the lower and upper quartile values and the whiskers representing the minimum and maximum data values. A Waters Xevo TQ-S Tandem LC-MS system was used to detect isobutyryl-CoA, using the 838.209 -> 331.147 ion transition (see Methods).

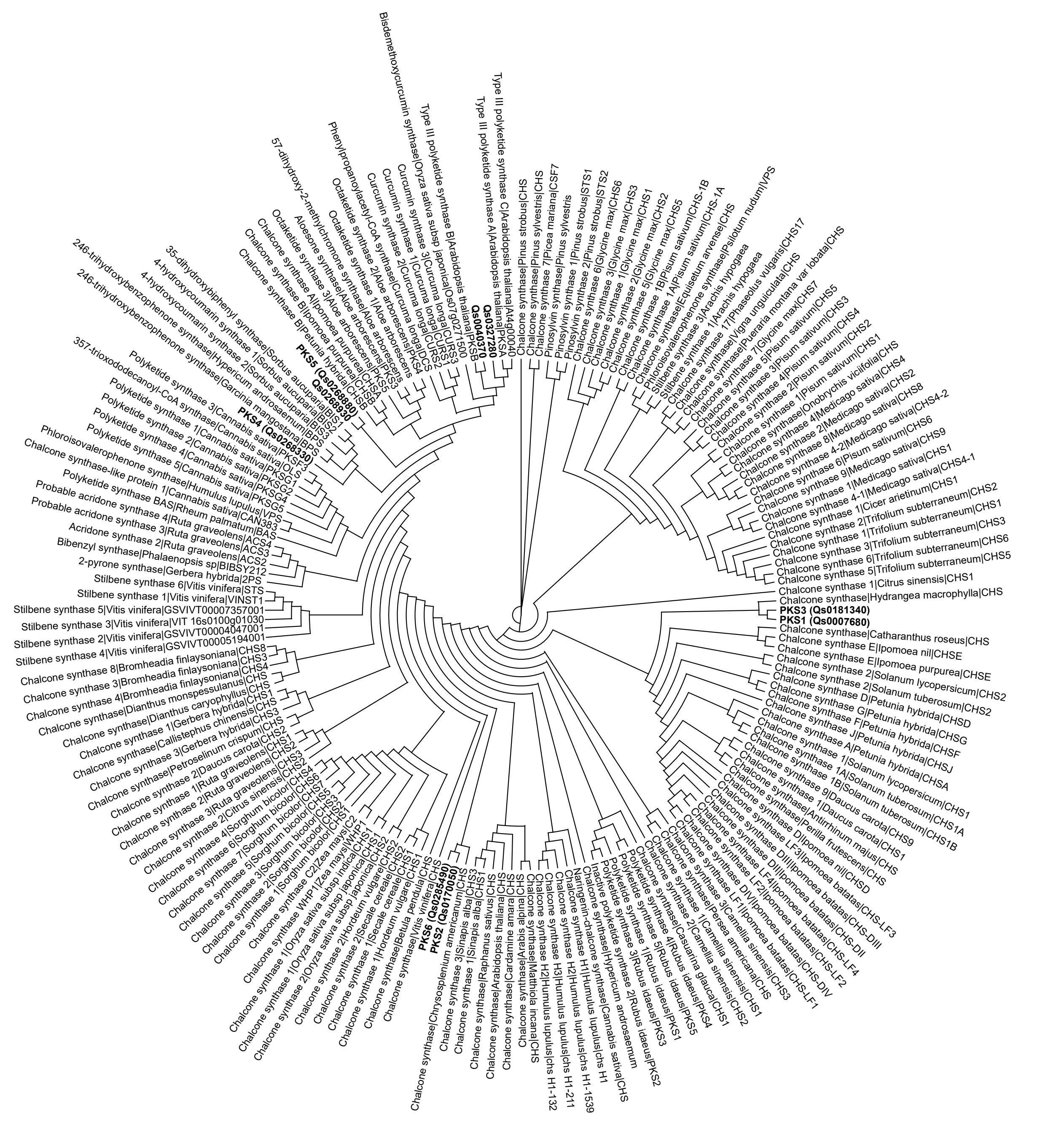

Supplementary Fig 6. Predicted PKSIII genes from *Q. saponaria*. The presence of Interpro domain IPR011141 (Polyketide synthase, type III) was used to mine 9 genes from the *Q. saponaria* genome. SwissProt (https://www.uniprot.org/uniprot/?query=reviewed:yes) was mined for protein sequences using the same domain, resulting in 165 full length PKSIII genes from Streptophyta. Sequences were aligned and a phylogeny generated as described in the Materials and Methods. Sequences from *Q. saponaria* are labelled in bold.

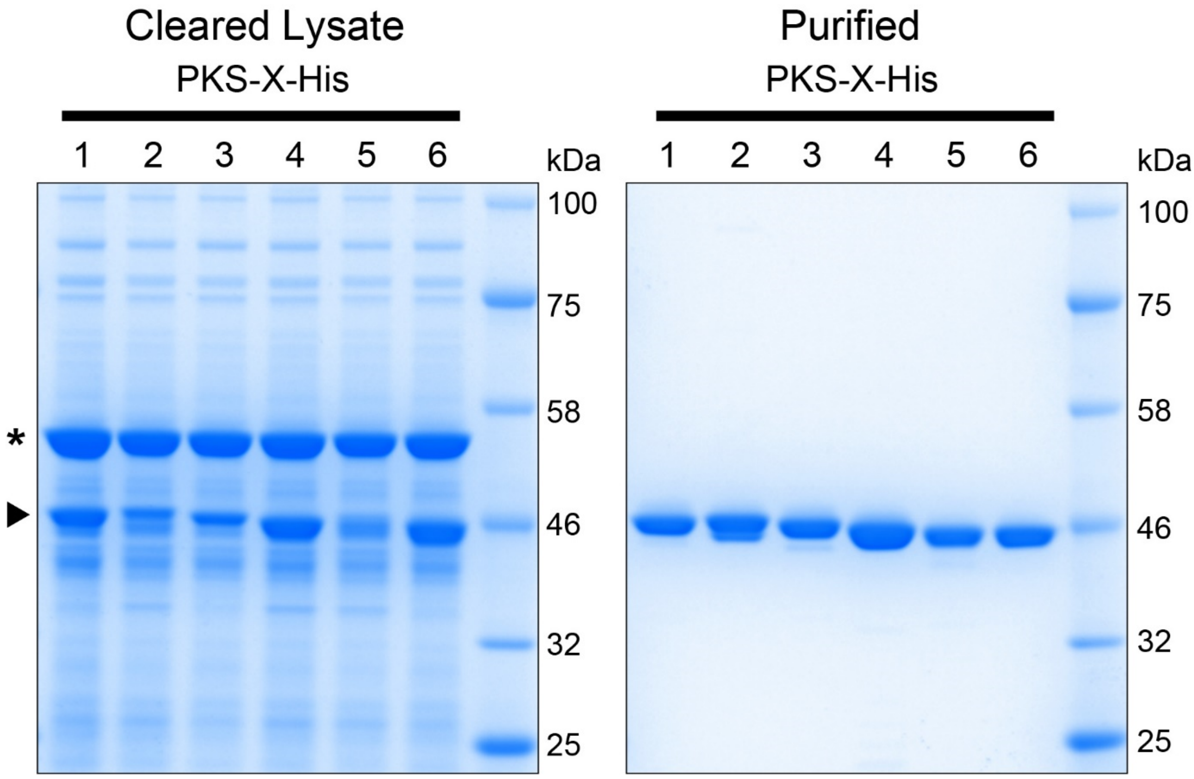

Supplementary Fig. 7. Heterologous expression in *N. benthamiana* and purification of His-tagged *Q. saponaria* PKS enzymes. The six *Q. saponaria* PKSs were expressed with C-terminal His-tags in *N. benthamiana* using *Agrobacterium*-mediated transient expression[17]. Six days after infiltration, the leaves were ground on ice using a mortar and pestle. The homogenate was filtered, centrifuged at low speed to remove debris, and then centrifuged at 30,000g for 20 min to obtain a cleared lysate without microsomes (Left). Arrowhead, transiently expressed PKSs; asterisk, Rubisco large subunit (which is highly abundant in plant leaf soluble extracts). His-tagged PKSs were purified by metal affinity chromatography. The concentration was adjusted to 0.5 mg protein/mL to ensure equal loading (Right). The purities of the PKSs were monitored by SDS-PAGE followed by Coommassie Brilliant Blue (CBB) staining. The gel is representative of at least three independent experiments. The two panels are from the same gel and were cropped and arranged for clarity. An uncropped gel is provided as Source Data.

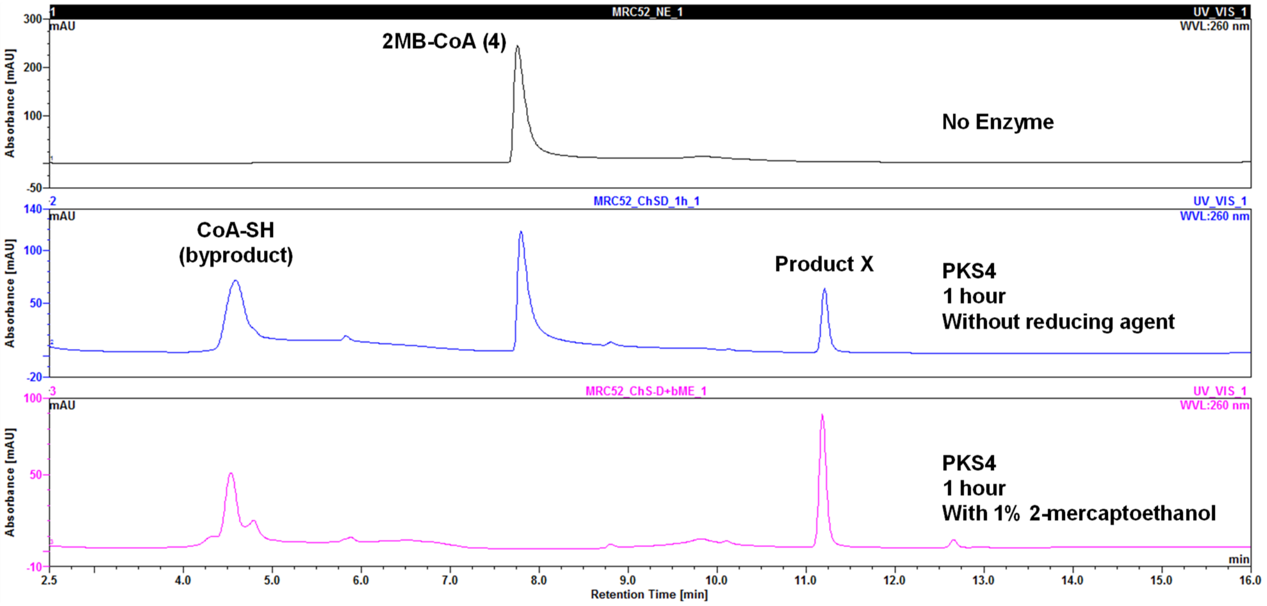

Supplementary Fig. 8. ***Q. saponaria*** **PKS4 converts 2-methylbutyryl-CoA (4) to a new product, X.** Purified PKS4 enzyme converted 2-methylbutyryl-CoA (2-MB-CoA, **4**) to a new product (X) *in vitro* in the presence of malonyl-CoA. The addition of 1% 2-mercaptoethanol (reducing agent) enabled complete conversion within 1 hour. The traces shown are Reverse Phase (RP)-C_{18} HPLC chromatograms with UV detection at 260 nm.

a

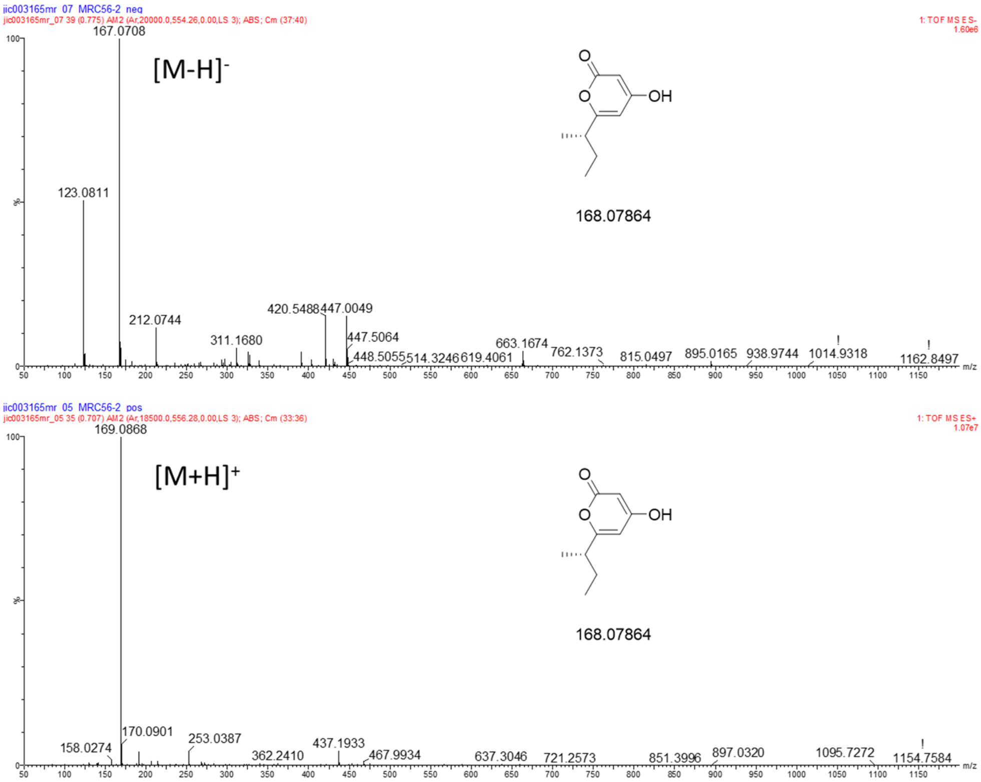

b

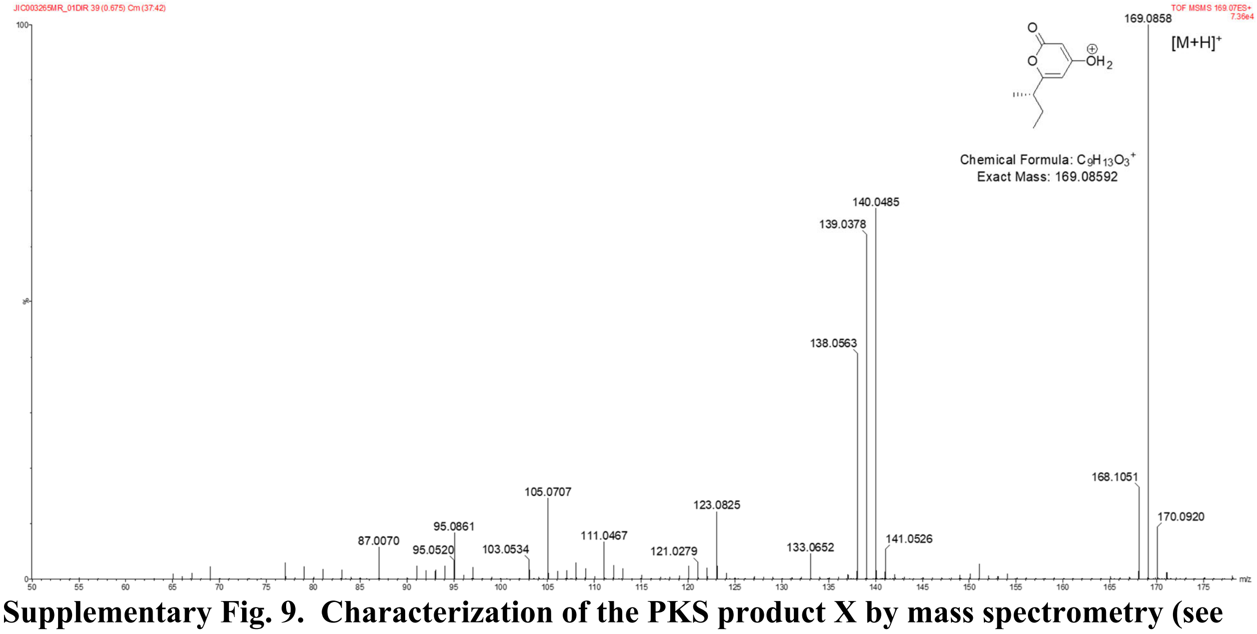

Supplementary Fig. 9. Characterization of the PKS product X by mass spectrometry (see Supplementary Fig. 8). (**a**) Mass spectra of X in negative and positive modes on a Synapt G2-Si mass spectrometer (Waters, Manchester, UK). The observed mass of product X (Supplementary Fig 11) (*m/z* 169.09, ESI$^+$ [M+H]$^+$) did not correspond to the mass (*m/z* 936.20, ESI$^+$ [M+H]$^+$) of the expected product, 6-methyl-3,5-dioxooctanoyl-CoA (**6**). (**b**) High Resolution (**HR**)-MS MS2 spectrum of compound X on a Synapt G2-Si mass spectrometer (Waters, Manchester, UK) in ESI positive mode. The MS2 spectrum of the selected precursor (*m/z* 169.09) was acquired directly via the tune page acquisition tab. The collision energy (CE) was ramped in steps of 5 between 25 and 40. The spectrum was processed in Masslynx 4.1 by selecting the appropriate CE.

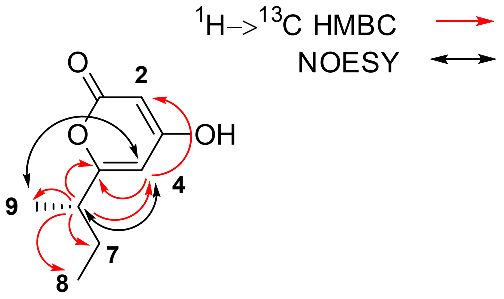

Supplementary Fig. 10. (S)-6-sec-butyl-4-hydroxy-2H-pyran-2-one (C_9 δ-lactone) with carbon atom numbering. Selected key interactions in 2D NMR spectra (HMBC and NOESY).

^{1}H-NMR (600 MHz, DMSO-d6) δ_H 5.48 (s, 1H, H-4), 4.56 (s, 1H, H-2), 2.31-2.28 (m, 1H, H-6), 1.57-1.50 (m, 1H, H_a-7), 1.44-1.37 (m, 1H, H_b-7), 1.07 (d, J = 6.9 Hz, 3H, CH_3-9), 0.81 (t, J = 7.4 Hz, CH_3-8). ^{13}C-NMR (150 MHz, DMSO-d6) δ_C 166.3 (s, 1C, C3), 165.5 (2s, 2 C, C1 and C5), 103.8 (d, 1C, C4), 86.2 (d, 1C, C2), 38.9 (d, 1C, C6), 26.9 (t, 1C, C7), 17.9 (q, 1C, C9), 11.5 (q, 1C, C8). HR-MS (ESI$^-$) for $C_9H_{12}O_3$ m/z calculated.: 167.0714 found: 167.0711 [M-H]$^-$.

a. ^{1}H NMR spectrum of (*S*)-6-*sec*-butyl-4-hydroxy-2*H*-pyran-2-one (C_9-δ-lactone)

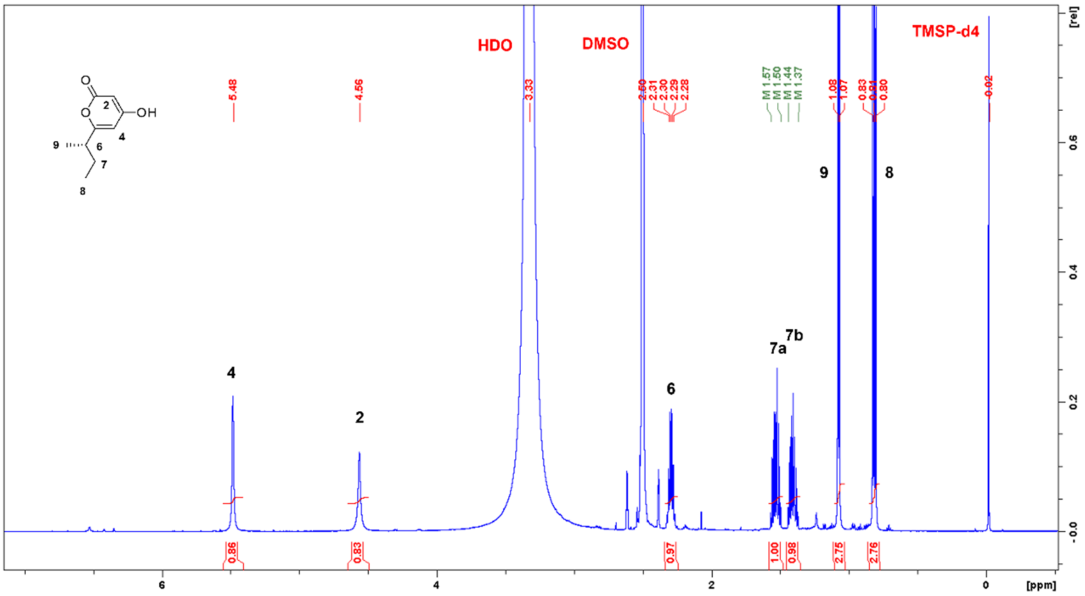

b. ^{13}C NMR spectrum of (*S*)-6-*sec*-butyl-4-hydroxy-2*H*-pyran-2-one (C_9-δ-lactone)

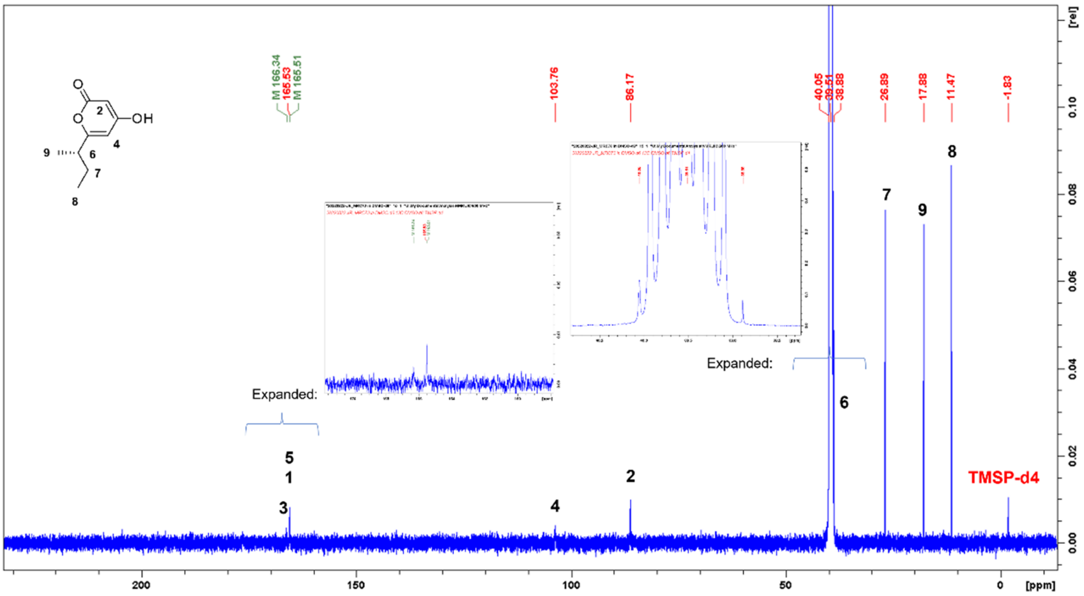

Supplementary Fig. 11. NMR spectra for the C_9-δ-lactone (continued on next page)

c. 2D NMR spectra of (*S*)-6-*sec*-butyl-4-hydroxy-2*H*-pyran-2-one (C_9-δ-lactone)

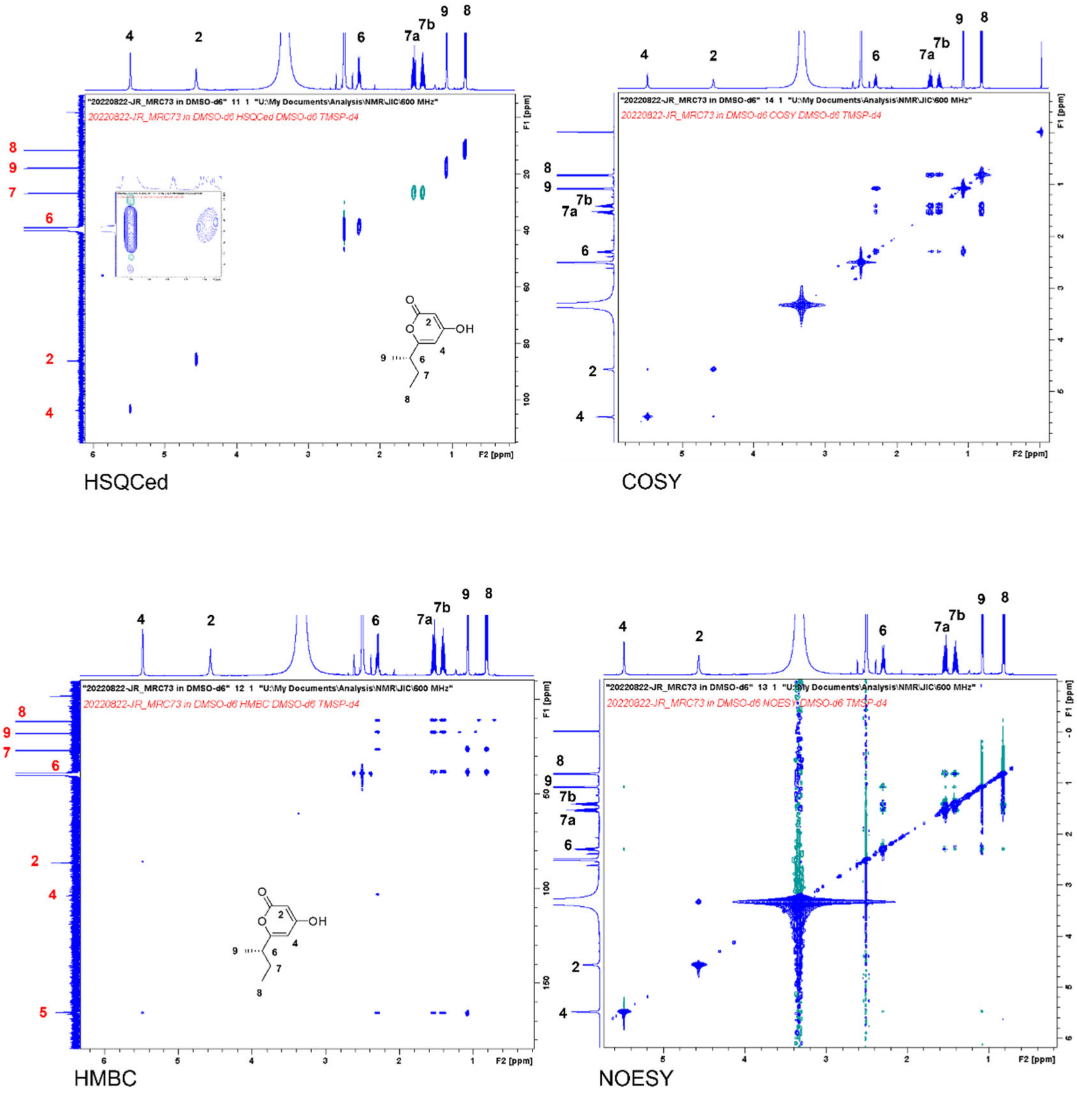

Supplementary Fig. 11. NMR spectra for the C_9-δ-lactone (continued)

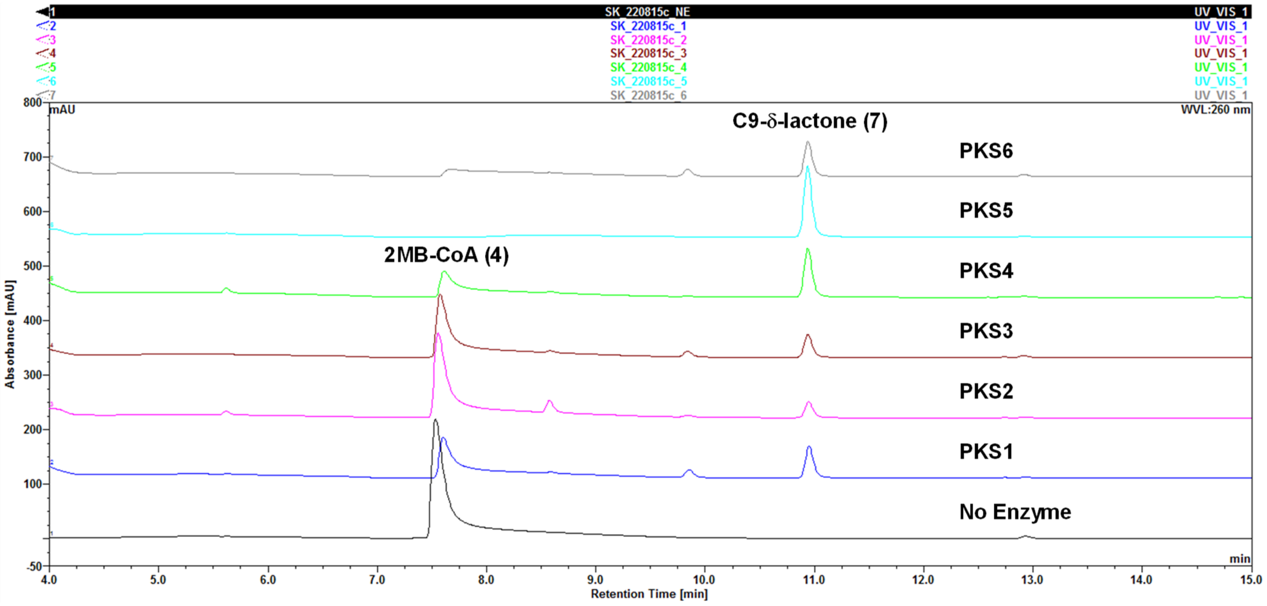

Supplementary Fig. 12. ***In vitro* activity assay comparing the six purified *Q. saponaria* PKS enzymes.**
The chemically synthesized starter molecule, 2-methylbutyryl-CoA (**4**) (1 mM) was mixed with malonyl-CoA (2 mM) in phosphate buffer (100 mM, pH 7.0) in the presence of 1 mM TCEP (reducing agent). Purified PKS enzymes were added at a final concentration of 0.1 mg protein/mL, and the mixture was incubated at 25°C for 150 min. After quenching with methanol, the mixture was subjected to analytical HPLC using a RP-C_{18} column, with monitoring by UV detection at 260 nm. The product peak area corresponding to the C_9-δ-lactone (**7**) at R_f = 11 min was measured to obtain relative activities for the six PKSs (Fig. 2b).

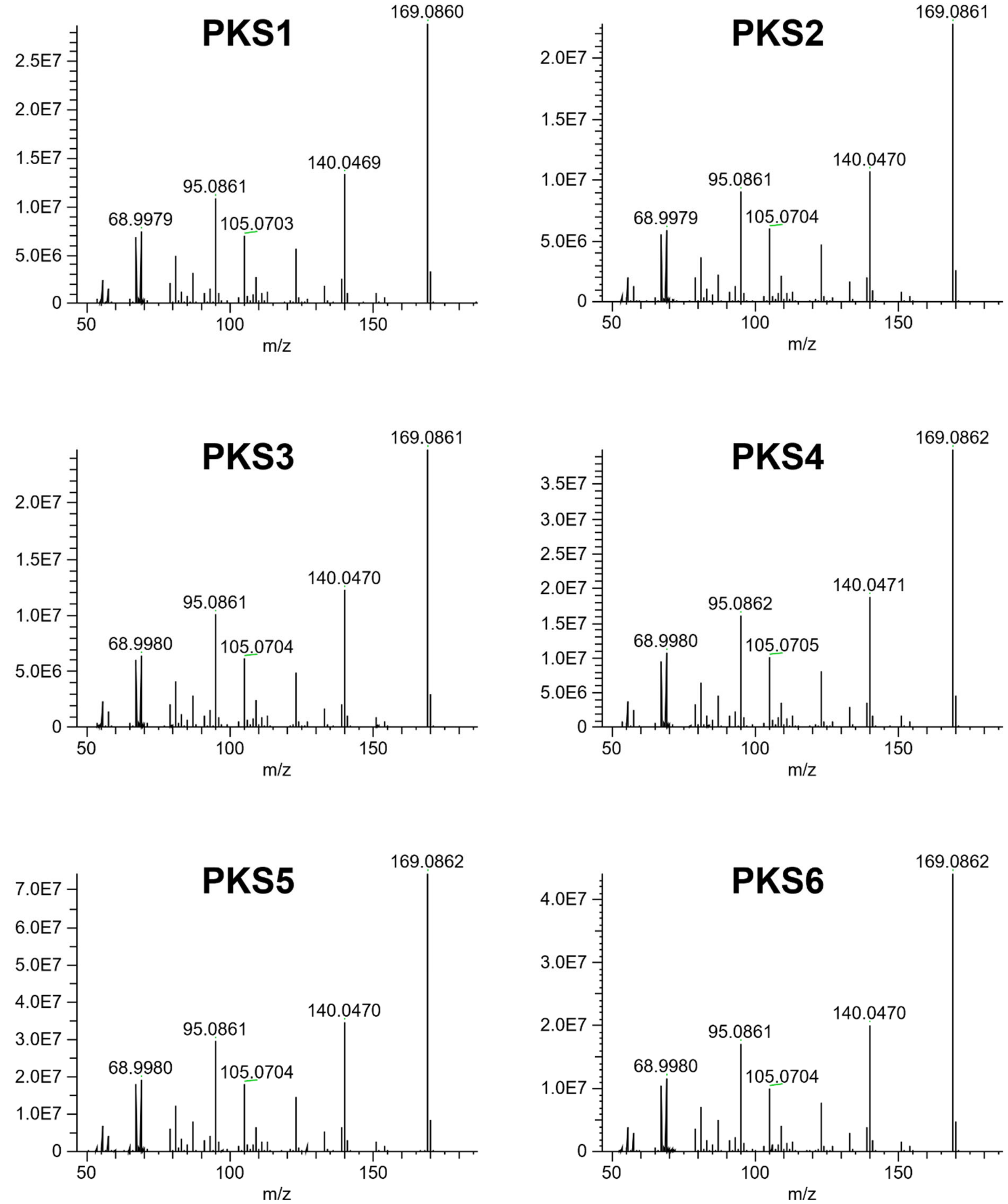

Supplementary Fig. 13. MS/MS analysis of *in vitro* products of the six PKS enzymes. The products of the six PKS enzymes were subjected to MS/MS fragmentation analysis. Mass fragments originating from $[M+H]^+ = 169.0859$. All data were derived from LC-MS analysis on a QExactive Hybrid Quadrupole-Orbitrap mass spectrometer using an RP-C_{18} column. Collision energy = 60 eV.

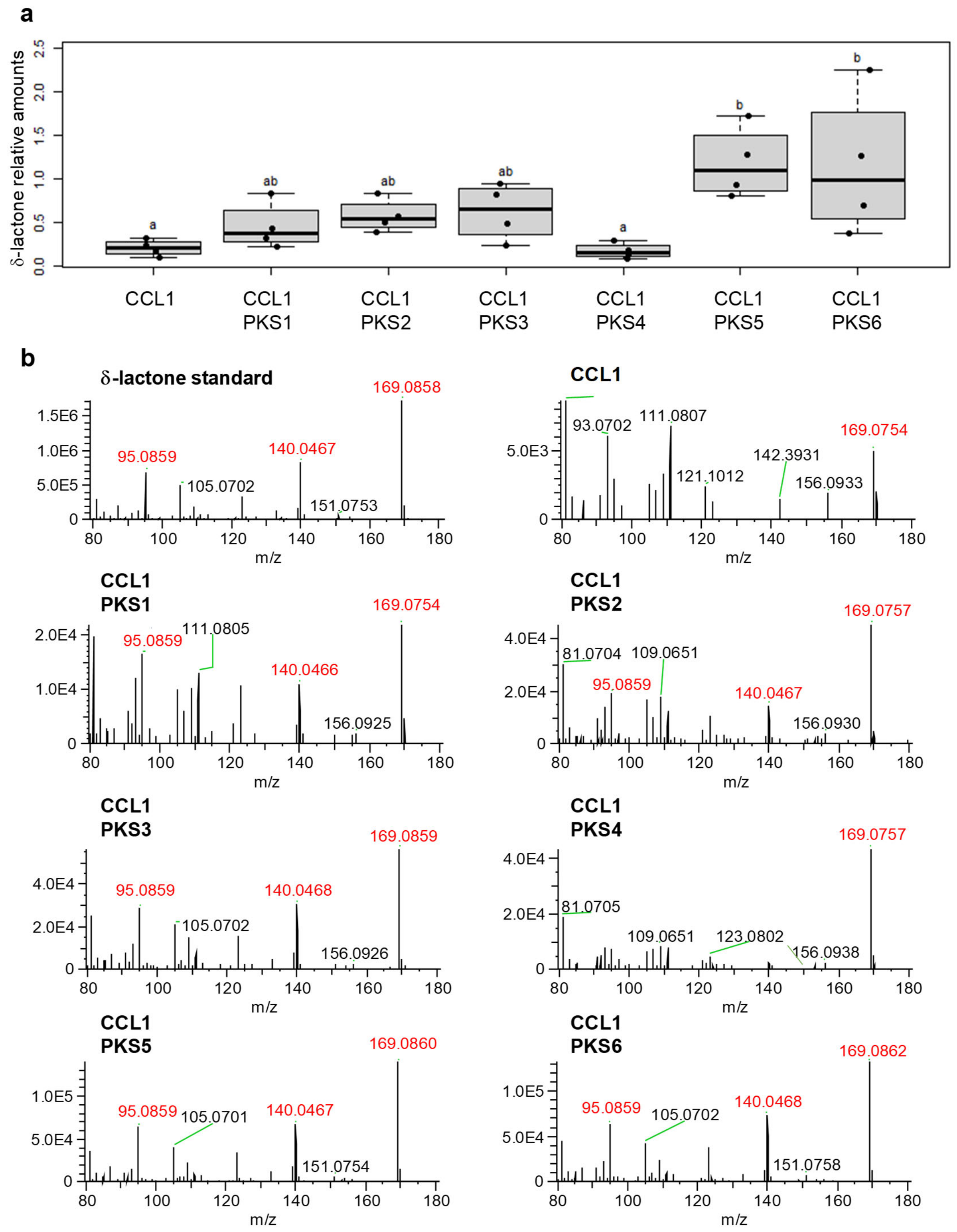

Supplementary Fig. 14. Detection and quantification of (*S*)-6-sec-butyl-4-hydroxy-2H-pyran-2-one (C_9-δ-lactone) (7) following *Agrobacterium*-mediated co-expression of CCL1 with each of the six *Q. saponaria* PKSs in leaves of *N. benthamiana.* **(a)** Quantitative analysis measuring relative amounts of C_9-δ-lactone individually produced by the six *Q. saponaria* PKS candidates. The boxplots show the distributions of the values for four biologically independent infiltrated leaf per treatment (represented by the dots), the centre line representing the median, the box showing the lower and upper quartile values and the whiskers representing the minimum and maximum data values. Letters represent significantly different data as determined by the two-sided post-hoc Tukey's HSD (p = 0.05) after ANOVA (Df = 6, p Value = 0.00538) using the multcompView package in R. **(b)** Spectra showing the fragmentation pattern of the C_9-δ-lactone for one replicate used in (**a**). The analyses were carried out using a QExactive Hybrid Quadrupole-Orbitrap mass spectrometer and an RP-C_{18} column.

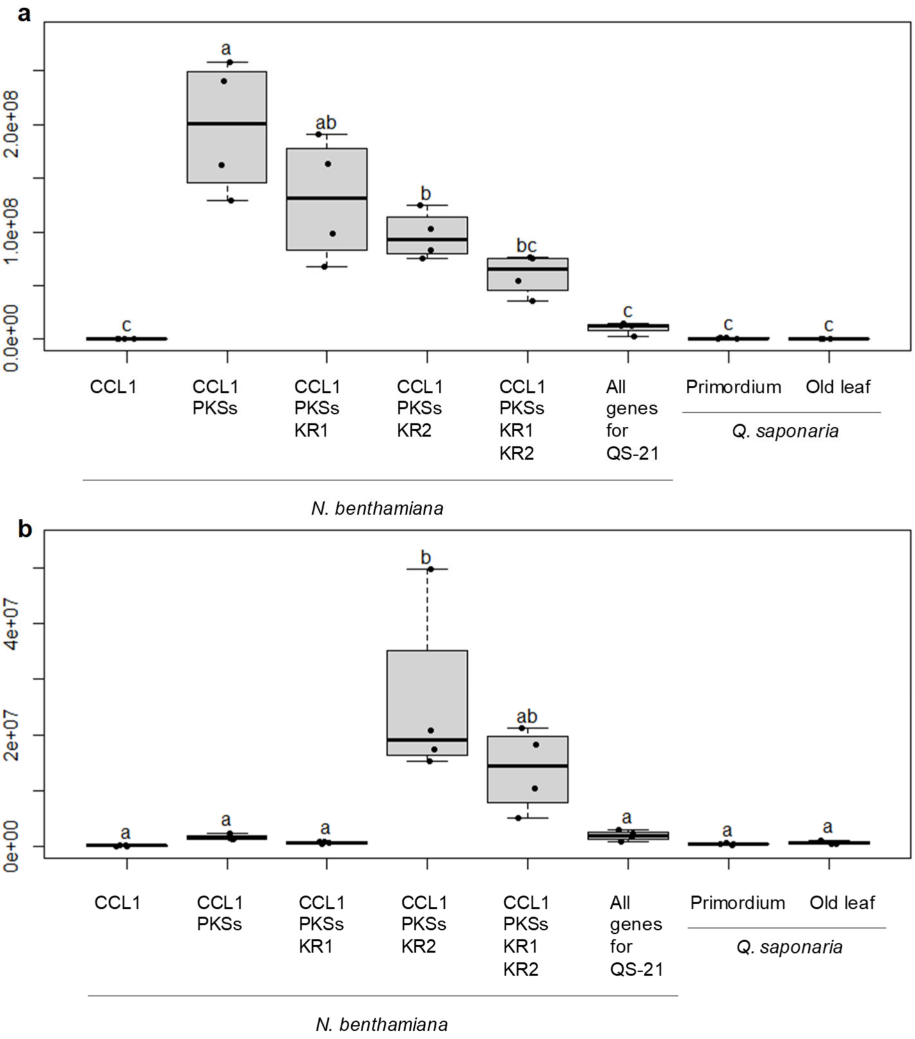

Supplementary Fig. 15. Formation of the C_9-δ-lactone and the monoreduced C_9-δ-lactone in *N. benthamiana* and *Q. saponaria*. (**a**) C_9-δ-lactone is formed in *N. benthamiana* leaves in the presence of the six PKS candidates. The addition of KR1 and/or KR2 lowers the amounts of C_9-δ-lactone suggesting the conversion of 6-methyl-3,5-dioxooctanoyl-CoA (**6**) into its mono- or di-reduced forms. When the gene set required to produce QS-21 is expressed in *N. benthamiana*, the amounts of C_9-δ-lactone are similar to the negative control suggesting efficient stabilisation or "pulling through" of (**6**), preventing the formation of C_9-δ-lactone. Similarly, C_9-δ-lactone was not detected in *Q. saponaria* primordia and old leaves suggesting that the presence of all enzymes protects against the formation of the C_9-δ-lactone. (**b**) The monoreduced-C_9-δ-lactone is formed in the presence of KR2. Co-expression of KR1 and KR2 results in a decrease in the monoreduced C_9-δ-lactone indicating that KR1 either catalyses the second keto-reduction or stabilizes (**6**), 5-hydroxy-6-methyl-3-oxooctanoyl-CoA and/or 3-hydroxy-6-methyl-5-oxooctanoyl-CoA, preventing the mono-reduced-C_9-δ-lactone formation. Similarly to the C_9-δ-lactone, the mono-reduced-C_9-δ-lactone did not significantly accumulate in the QS-21 producing *N. benthamiana* leaves and in *Q. saponaria.* Data were normalized on dry weight. The boxplots show the distributions of the values for four biologically independent infiltrated leaf per treatment (represented by the dots), the centre line representing the median, the box showing the lower and upper quartile values and the whiskers representing the minimum and maximum data values. Letters represent significantly different data as determined by the two-sided post-hoc Tukey's HSD ($p = 0.05$) after ANOVA (C_9-δ-lactone: Df = 7, p Value = $5.37E^{-09}$; monoreduced C_9-δ-lactone: Df = 7, p Value = $2.29E^{-05}$) using the multcompView package in R.

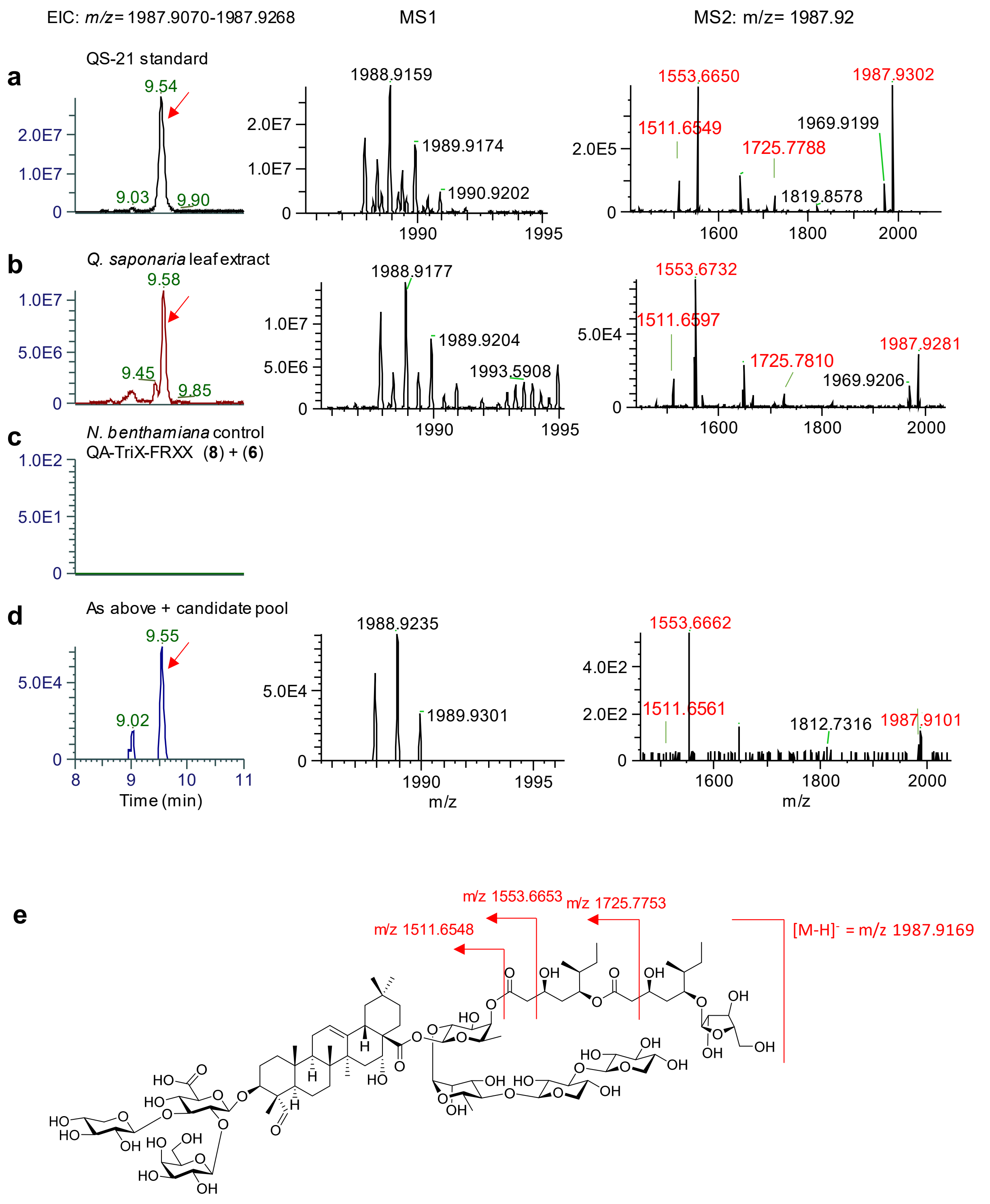

Supplementary Fig. 16. Co-expression of the genes for biosynthesis of QA-TriX-FRXX (8) and the acyl donor (6) with the pool of candidate genes for addition of the acyl moiety results in production of a compound identical to a standard of QS-21 (D-xyl) (2). (a-d) Extracted ion chromatograms (EIC), left; mass spectra for peaks indicated by arrows, right. (**a**) QS-21 standard (10 µM); (**b**) *Q. saponaria* leaf extract; (**c, d**) extracts of *N. benthamiana* leaves infiltrated with the gene set for production of QA-TriX-FRXX (**8**) without (**c**) and with (**d**) the pool of candidate acyl chain genes. (**e**) The structure of QS-21 (D-xyl) (**2**) with the characteristic mass fragments indicated.

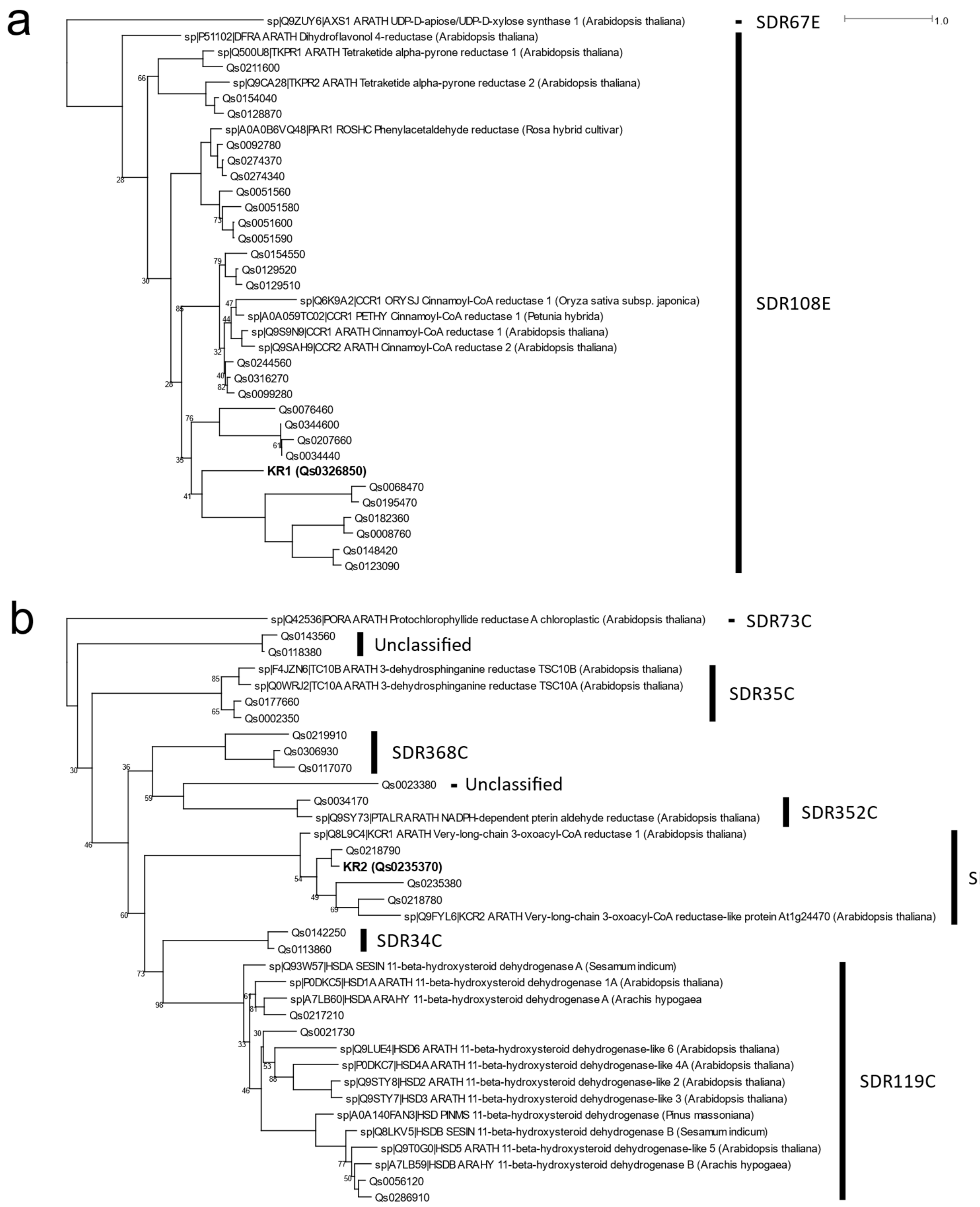

Supplementary Fig. 17. Phylogeny of *Q. saponaria* SDR enzymes. SDRs were mined from the *Q. saponaria* genome and reference sequences from SwissProt (https://www.uniprot.org/uniprot/?query=reviewed:yes) using Pfams PF00106, PF01073 and PF01370. SDR families were classified via pHMMs according to sdr-enzymes.org[32]. Sequences were aligned and phylogenies generated as described in the Materials and Methods. The scale bar indicates the number of amino acid substitutions per site. (**a**) A subset of SDRs containing the PF01370 domain, showing the placement of KR1 within the SDR108E family, with a representative of the SDR67E family as an outgroup. Members of the SDR108E family are known to catalyse the reduction of several phenolic compounds[48]. (**b**) A subset of SDRs containing the PF00106 domain, showing the placement of KR2 within the SDR12C family and phylogenetic relation to other PF00106 SDR families. The SDR12C family is also found in animals and its members are known to oxidise steroids[49]. A representative sequence of the SDR73C family is used as an outgroup.

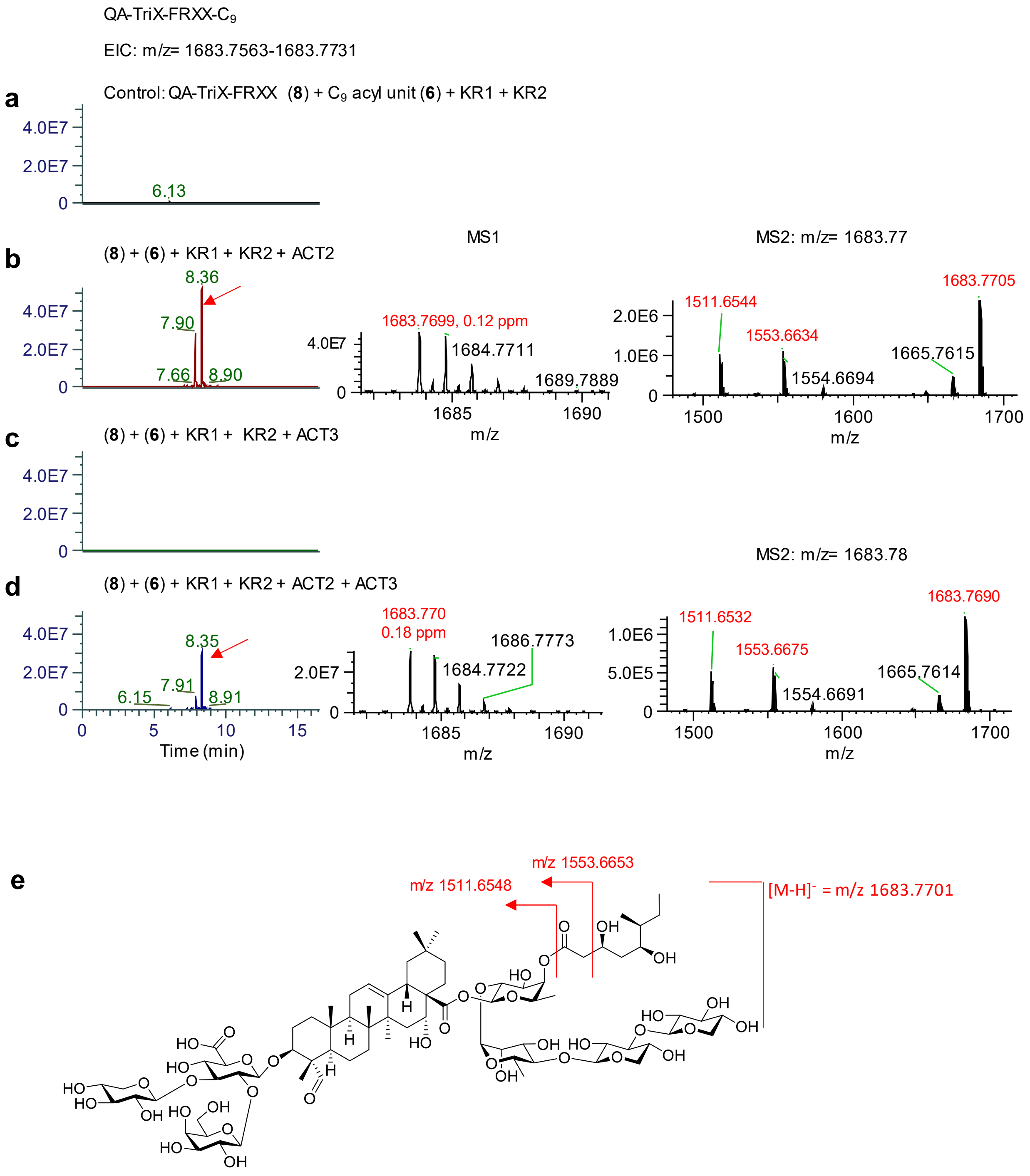

Supplementary Fig. 18. Functional analysis of the ACT candidates. (a) Control showing that without the ACT candidates, QA-TriX-FRXX-C_9 (**9**) is not made. (**b,c**) Addition of ACT2 (**b**) but not ACT3 (**c**) leads to formation of QA-TriX-FRXX-C_9 (**9**). (**d**) QA-TriX-FRXX-C_9 (**9**) is formed when both ACT2 and ACT3 are added. (e) Fragmentation pattern of QA-TriX-FRXX-C_9 (**9**). The chromatograms and mass spectra were generated using a QExactive Hybrid Quadrupole-Orbitrap mass spectrometer and an RP-C_{18} column.

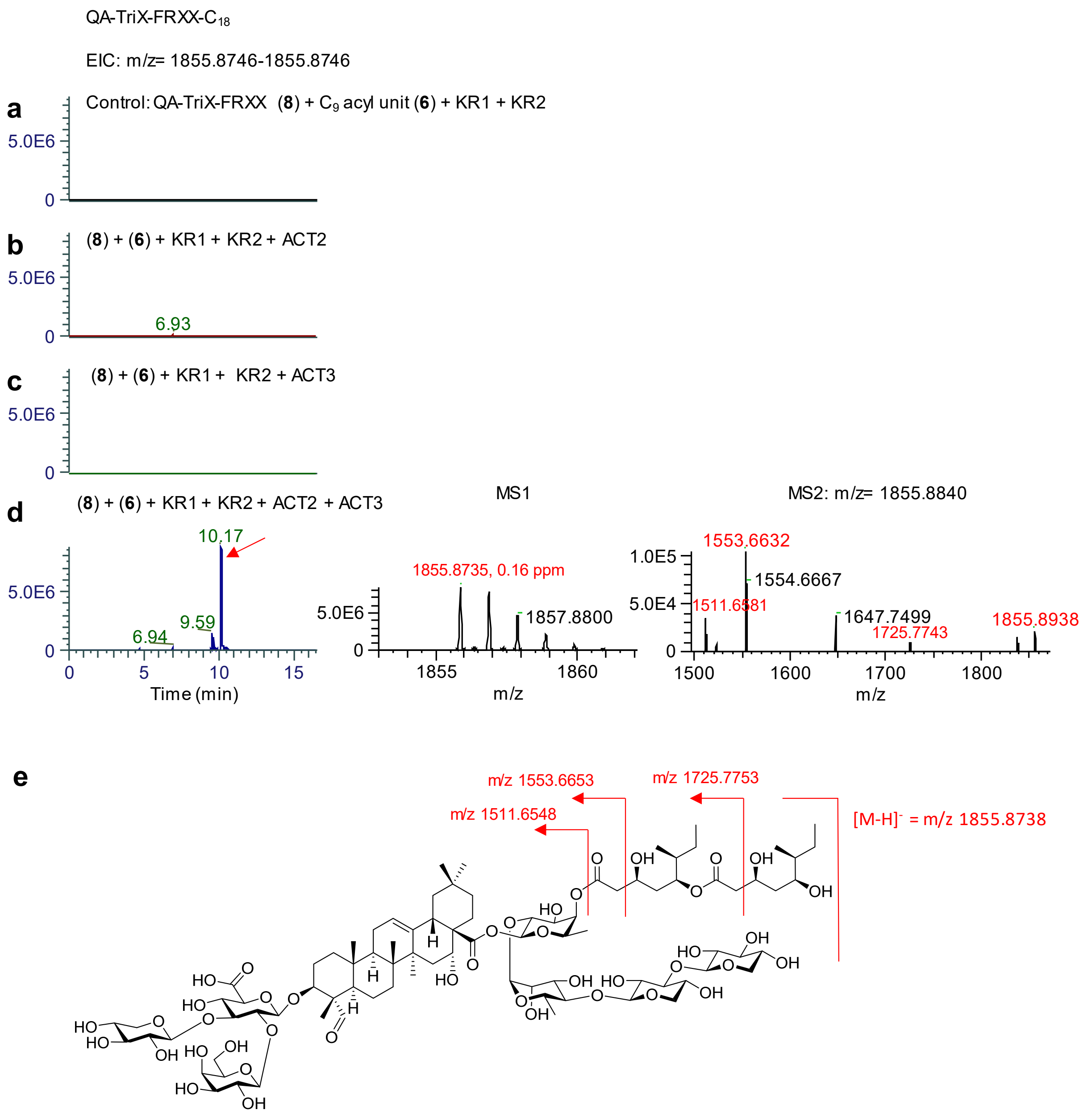

Supplementary Fig. 19. Further analysis of the ACT candidates – biosynthesis of QA-TriX-FRXX-C18 (10). (a-d) QA-TriX-FRXX-C18 (**10**) is only produced when both ACT2 and ACT3 are included together (**d**). Neither ACT2 or ACT3 are able to generate QA-TriX-FRXX-C18 (**10**) by themselves. Together with the results shown in Supplementary Fig. 21, these results suggest that ACT2 adds the first C9 acyl chain and ACT3 the second one. (**e**) Fragmentation pattern of QA-TriX-FRXX-C18 (**10**). The chromatograms and mass spectra were generated using a QExactive Hybrid Quadrupole-Orbitrap mass spectrometer and RP-C18 column.

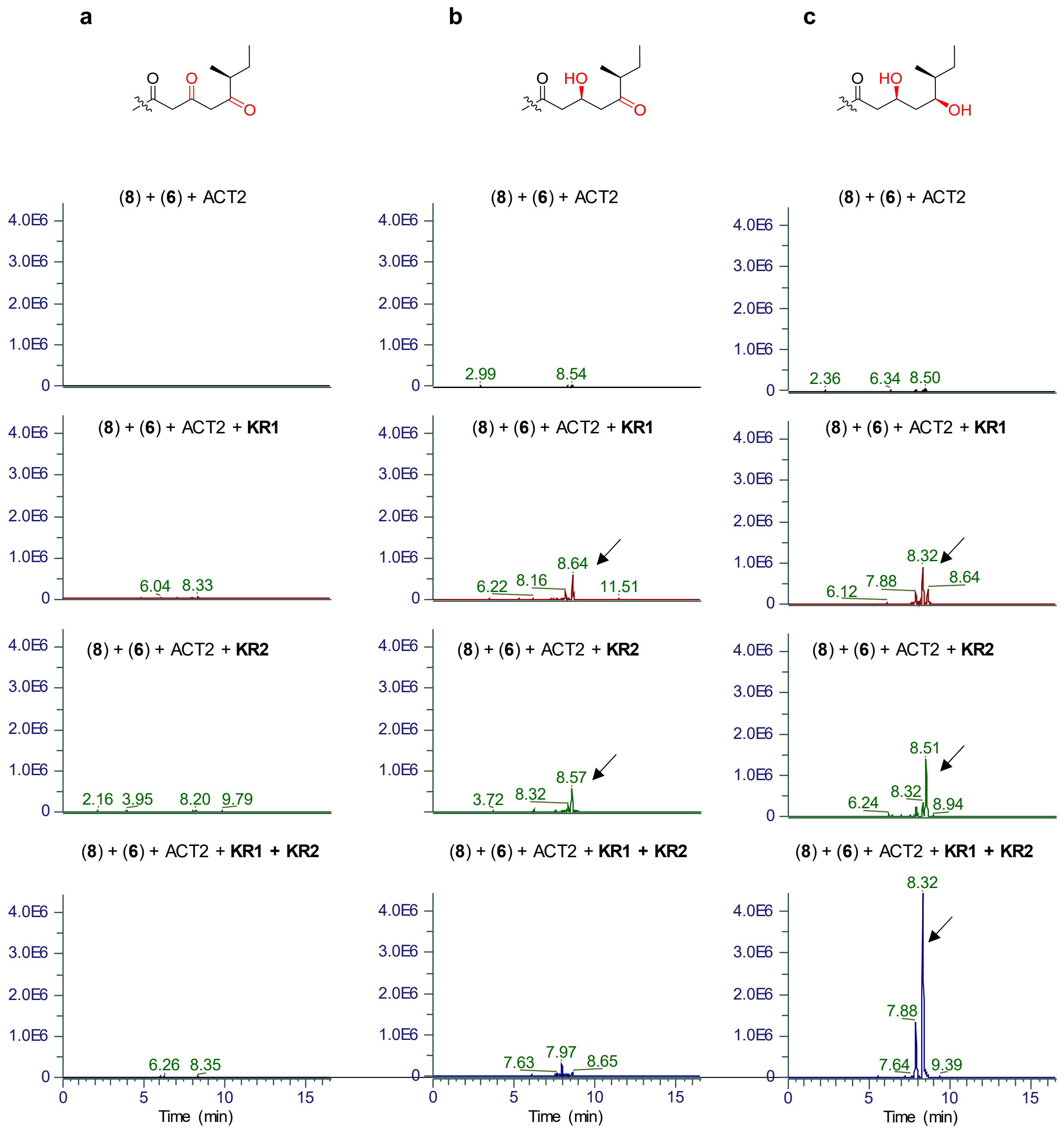

Supplementary Fig. 20. Screening for the reductase modifications on QA-TriX-FRXX-C9 (9). (a) The acyl chain attached to the triterpene scaffold but retaining the C3 and C5 ketones was not detected in *N. benthamiana* infiltrated with ACT2 with or without KR1 and KR2, suggesting that at least one reduction is required for the C9 acyl unit to be transferred to the triterpene scaffold. (**b**) Small amounts of QA-TriX-FRXX-C9 (**9**) retaining one of the acyl donor ketones were detected in the presence of either KR1 or KR2, suggesting that only one reduction is essential for transfer of the C9 acyl unit to the scaffold by ACT2. Note: the position of the alcohol could be at the C3 or C5 of the C9 acyl unit. The low levels of mono-reduced QA-TriX-FRXX-C9 may be due to preferential affinity of ACT2 for the fully reduced C9 acyl unit. (**c**) The levels of fully reduced QA-TriX-FRXX-C9 (**9**) are higher than those of mono-reduced QA-TriX-FRXX-C9, especially when the two KRs are co-infiltrated as the mono-reduced form was barely detectable for this combination, implying that KR1 and KR2 work synergistically. LC-MS extracted chromatograms (EIC) in negative mode are shown, with the screened masses of the negative ions formed by loss of hydrogen being *m/z* = 1679.7250-1679.7418 for QA-TriX-FRXX-C9 no reduction ($C_{78}H_{120}O_{39}$), *m/z* = 1681.7406-1681.7574 for QA-TriX-FRXX-C9 one reduction ($C_{78}H_{122}O_{39}$), *m/z* = 1683.7563-1683.7731 for QA-TriX-FRXX-C9 two reductions ($C_{78}H_{124}O_{39}$).

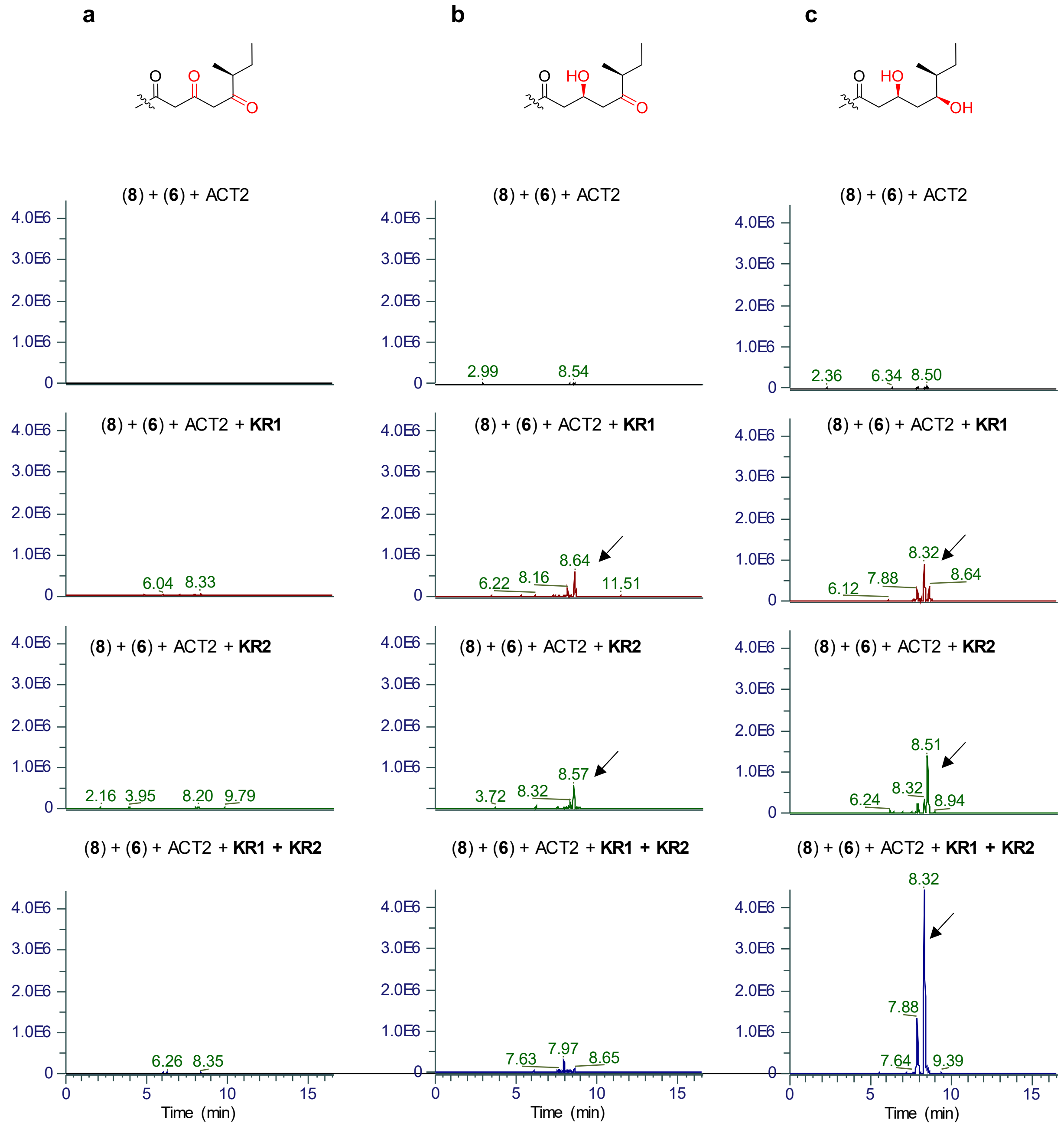

Supplementary Fig. 21. Screening for the reductase modifications on QA-TriX-FRXX-C_{18} (10). (a) Screening for QA-TriX-FRXX-C_{18} (**10**) retaining one of the ketones (note - any of the three positions highlighted in red may be the one retaining the ketone). Traces amounts were detected when the keto-reductases were infiltrated independently. A clear peak was detected when the KRs were co-infiltrated. This suggests again that both ketoreductases are essential. (**b**) The amount of fully reduced QA-TriX-FRXX-C_{18} (**10**) was >ten times higher than that of the doubly reduced QA-TriX-FRXX-C_{18}, indicating that the fully reduced C_{18} acyl chain is favoured. LC-MS extracted chromatograms (EIC) in negative mode are shown. The screened masses of the negative ions formed by loss of hydrogen were *m/z* = 1853.8497-1853.8683 for QA-TriX-FRXX-C_{18} with two reductions ($C_{87}H_{138}O_{42}$) and *m/z* = 1855.8653-1855.8839 for QA-TriX-FRXX-C_{18} with three reductions ($C_{87}H_{140}O_{42}$). No peaks were detected when screening for QA-TriX-FRXX-C_{18} lacking any reductions of the C_{18} acyl chain ($C_{87}H_{134}O_{42}$, *m/z* = 1849.8185-1849.8369), or with only one reduction ($C_{87}H_{136}O_{42}$. *m/z* = 1851.8340-1851.8526).

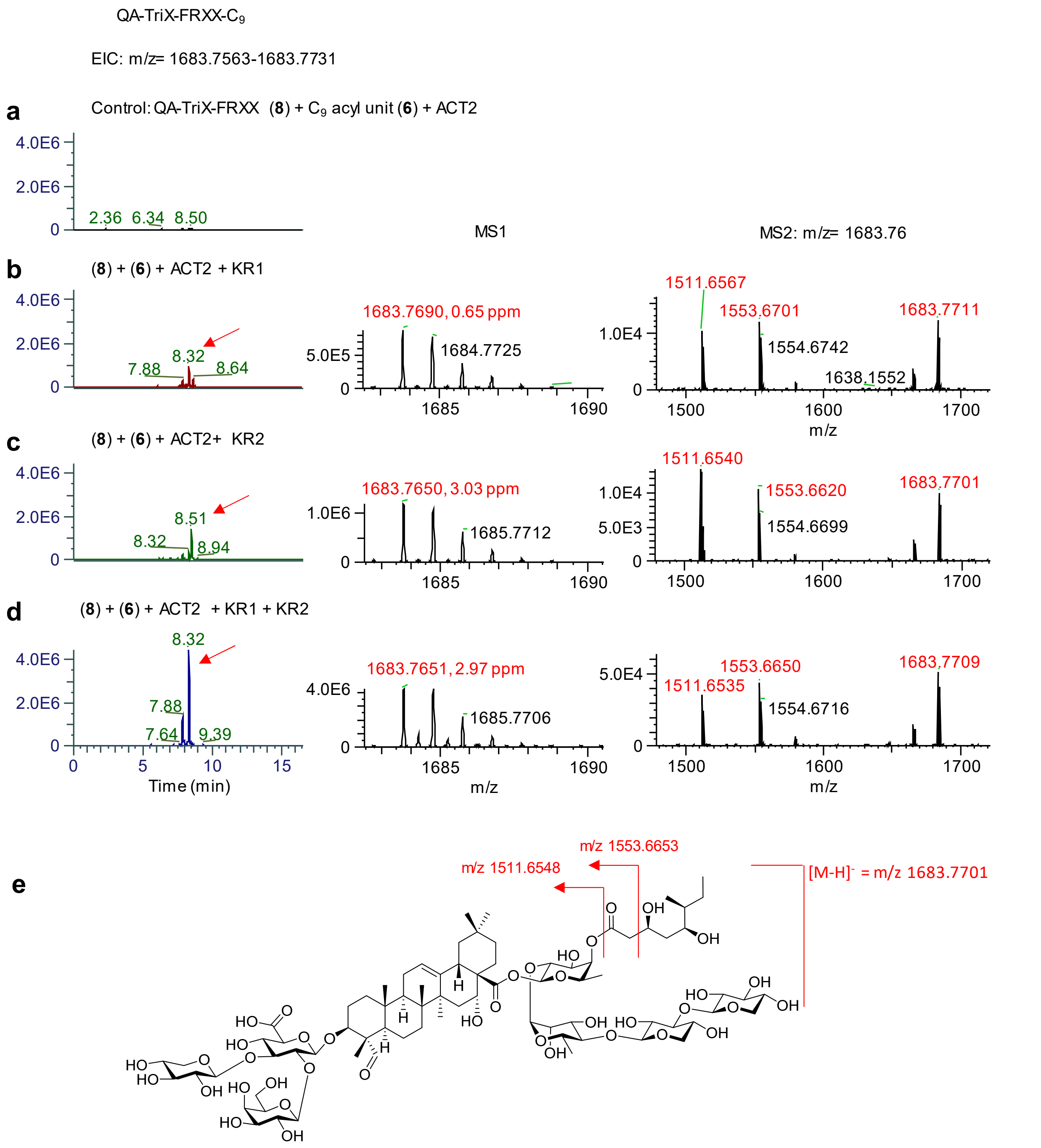

Supplementary Fig. 22. Functional analysis of the KR candidates (QA-TriX-FRXX-C_9). (a) Control showing that without the keto-reductase candidates, QA-TriX-FRXX-C_9 (**9**) was not made. (**b, c**) Addition of either KR1 (**b**) or KR2 (**c**) results in production of QA-TriX-FRXX-C_9 (**9**). (**d**) KR1 and KR2 act synergistically to give higher levels of QA-TriX-FRXX-C_9 (**9**). (**e**) Fragmentation pattern of QA-TriX-FRXX-C_9 (**9**). The chromatograms and mass spectra were generated using a QExactive Hybrid Quadrupole-Orbitrap mass spectrometer and an RP-C_{18} column.

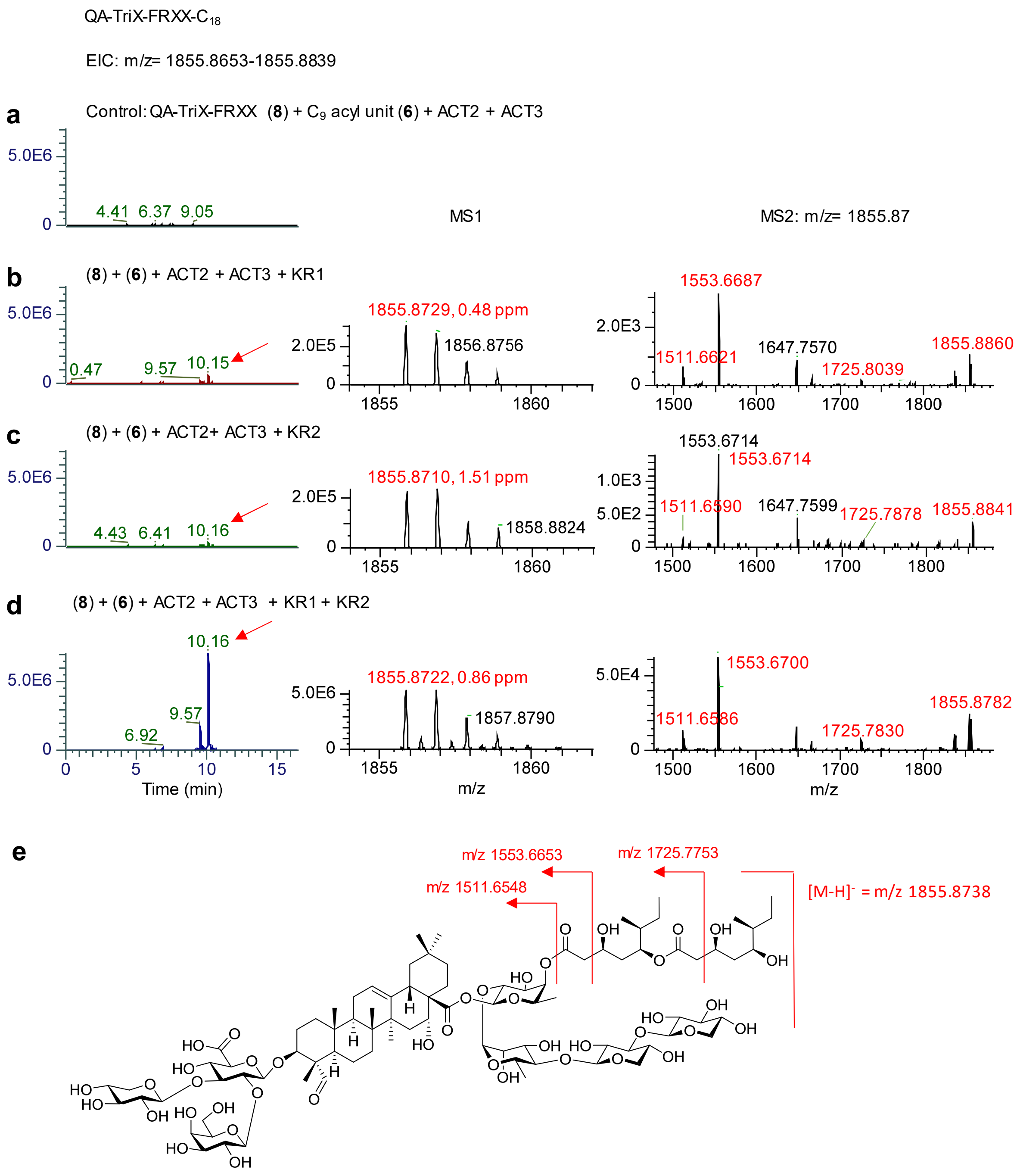

Supplementary Fig. 23. Functional analysis of the KR candidates (QA-TriX-FRXX-C18). (a) Control showing that without the keto-reductase candidates, QA-TriX-FRXX-C18 (**10**) was not made. (**b, c)** Addition of KR1 (**b**) or KR2 (**c**) leads to production of small amounts of QA-TriX-FRXX-C18 (**10**). (**d)** KR1 and KR2 act synergistically to give higher levels of QA-TriX-FRXX-C18 (**10**) than when either keto-reductase is included separately. **(e)** Fragmentation pattern of QA-TriX-FRXX-C18 (**10**). The chromatograms and mass spectra were generated using a QExactive Hybrid Quadrupole-Orbitrap mass spectrometer and an RP-C18 column.

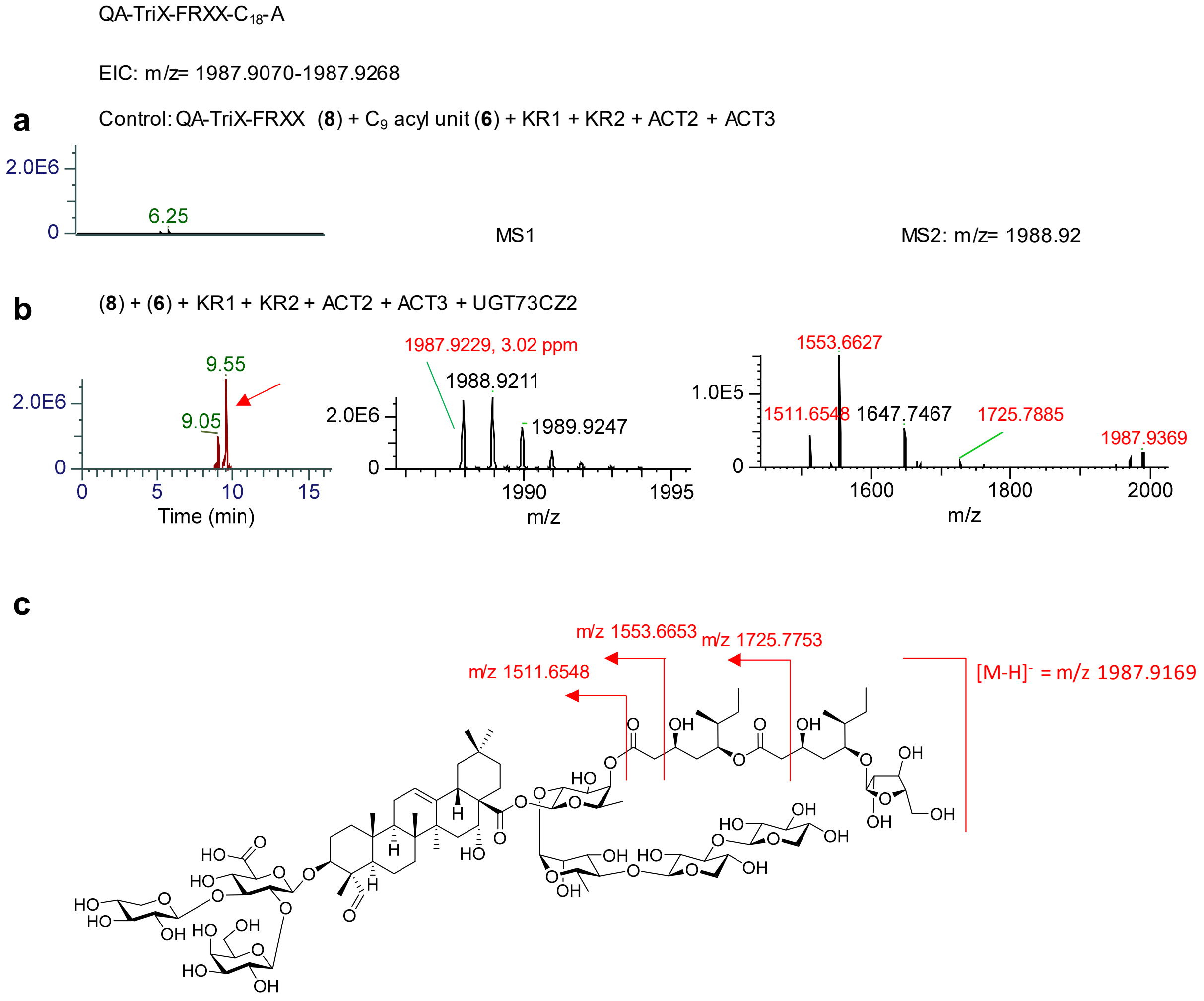

Supplementary Fig. 24. Functional analysis of the arabinofuranosyl transferase candidate UGT73CZ2. (**a**) Control showing that without UGT73CZ2, QA-TriX-FRXX-C18-A (**10**) is not made. (**b**) UGT73CZ2 is able to transfer an arabinofuranose (or a sugar of a similar mass) to QA-TriX-FRXX-C18 to putatively make QS-21(D-xyl form) (**2**). (**c**) Fragmentation pattern of (**2**). The chromatograms and mass spectra were generated using a QExactive Hybrid Quadrupole-Orbitrap mass spectrometer and an RP-C18 column.

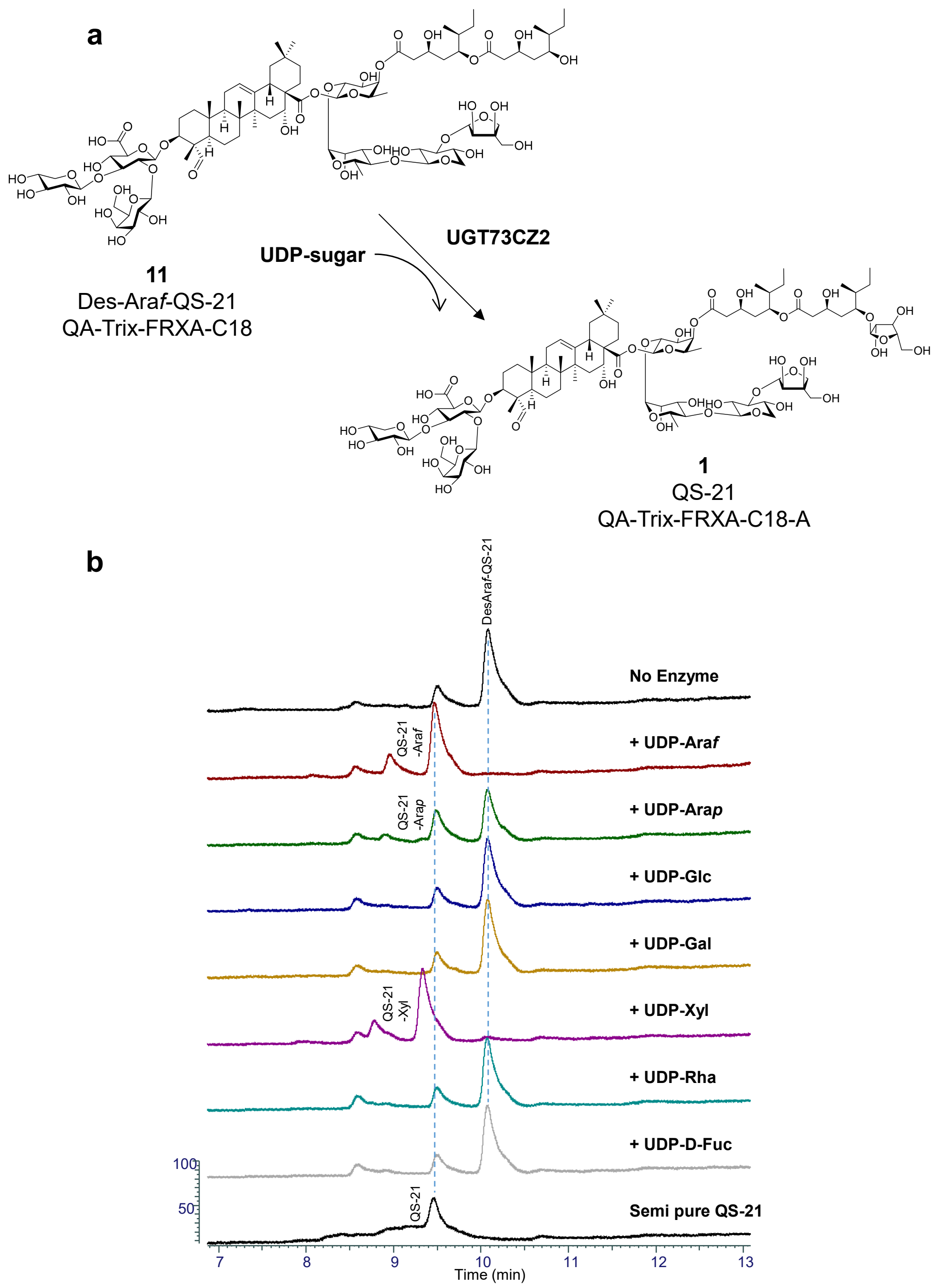

Supplementary Fig. 25. UDP sugar donor specificity of UGT73CZ2. (**a**) Conversion of des-Ara*f* QS-21 (QA-TriX-FRXA-C$_{18}$; (**11**)) to QS-21 (**2**). (**b**) Evaluation of the UDP sugar donor specificity of UGT73CZ2. UGT73CZ2 was expressed with a carboxy-terminal hexahistidine tag in *N. benthamiana* and purified for use in *in vitro* assays (see Materials and Methods). A starter molecule, 0.1 mM des-arabinosyl-QS-21 (Des-Araf-QS-21, QA-TriX-FRXA-C$_{18}$; **11**) was mixed with 0.5 mM of each UDP sugar in a final volume of 50 µL. Reactions were initiated by addition of 0.8 µg of purified UGT73CZ2 to the reaction mixtures and incubated at 25°C for 14 h. After quenching with methanol, the mixtures were analysed with a QExactive Hybrid Quadrupole-Orbitrap mass spectrometer equipped with a CAD and an RP-C$_{18}$ column. The negative control is at the top (No Enzyme), and a QS-21 standard is shown at the bottom. UGT73CZ2 is able to transfer L-arabinofuranose and also D-xylose to **11** (des-Araf-QS-21; QA-TriX-FRXA-C$_{18}$) with almost 100% efficiency, as shown by the absence of the substrate peaks. Charged Aerosol Detector (CAD) chromatograms are shown. QS-21-D-Xyl (QA-TriX-FRXA-C$_{18}$-X) eluted earlier than QS-21 (**1**).

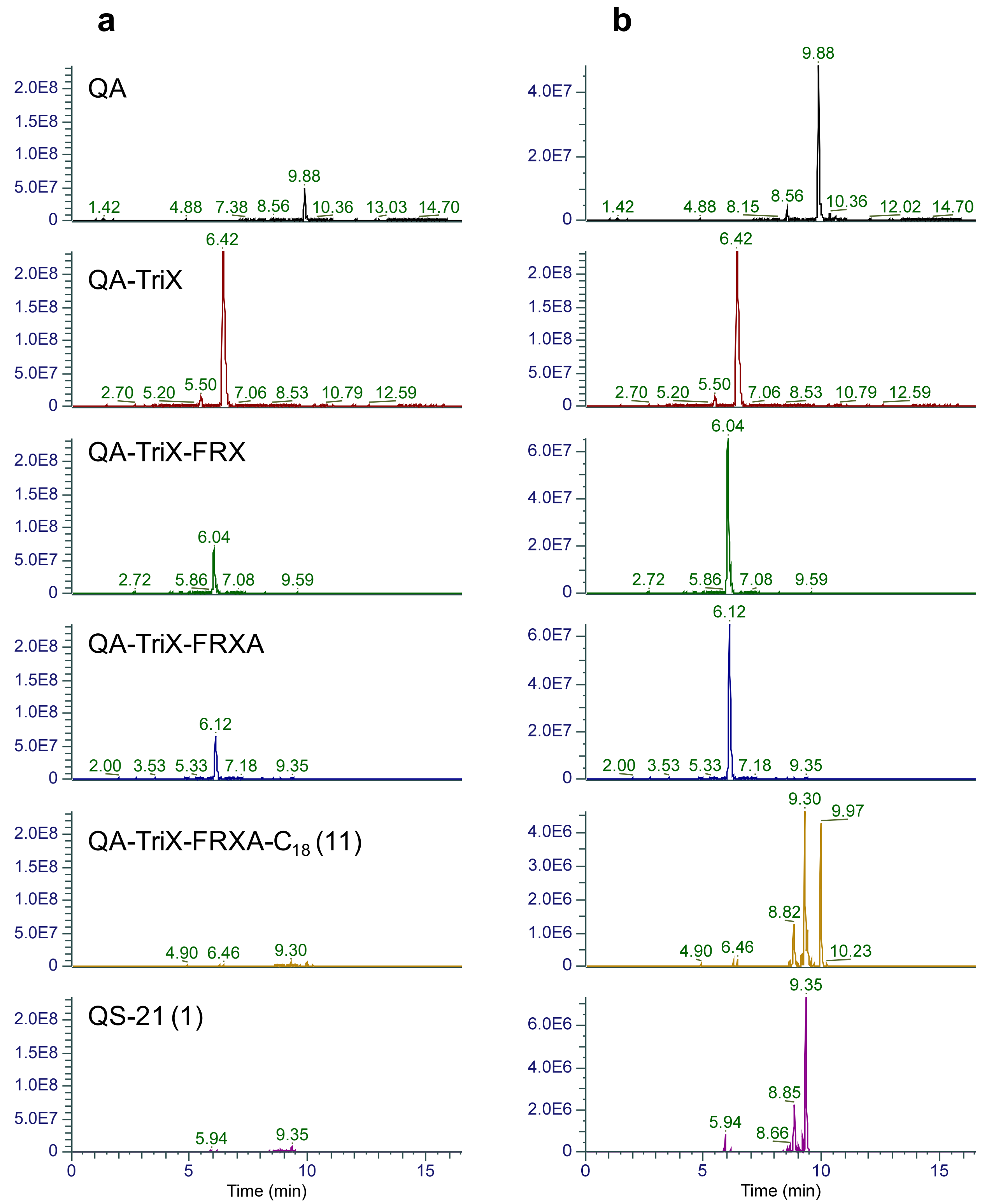

Supplementary Fig. 26. Detection of QS-21 and selected pathway intermediates in extracts from *N. benthamiana* leaves expressing QS-21 biosynthetic genes. (**a**) Chromatograms showing QS-21 (**1**) and five pathway intermediates with Y axes set at the level of the highest peak, that of QA-TriX. (**b**) As for (**a**) but with the Y axes adjusted to each peak. The screened masses of the negative ions formed by loss of hydrogen in negative mode are as follows: QA, m/z = 486.3351; QA-TriX, m/z = 955.4544; QA-TriX-FRX, m/z = 1379.6125; QA-TriX-FRXA, m/z = 1511.6548; QA-TriX-FRXA-C_{18} (**11**), m/z = 1855.8746; QS-21 (**1**), m/z = 1987.9169.

Score
0 1

Protein	Predicted Localization(s)	Cytoplasm	Nucleus	Extracellular	Cell membrane	Mitochondrion	Plastid	Endoplasmic reticulum	Lysosome/Vacuole	Golgi apparatus	Peroxisome
QsbAS1	Endoplasmic reticulum, Cytoplasm	0.490	0.180	0.049	0.383	0.330	0.083	0.782	0.461	0.247	0.054
CYP714E52	Endoplasmic reticulum	0.156	0.091	0.058	0.218	0.177	0.060	0.823	0.199	0.105	0.009
CYP716A224	Endoplasmic reticulum	0.139	0.073	0.052	0.158	0.089	0.073	0.940	0.335	0.176	0.033
CYP716A297	Endoplasmic reticulum	0.168	0.078	0.048	0.190	0.058	0.034	0.944	0.283	0.160	0.067
CSLM1	Endoplasmic reticulum	0.093	0.064	0.051	0.493	0.105	0.043	0.648	0.341	0.491	0.035
CSLM2	Endoplasmic reticulum, Cell membrane	0.087	0.060	0.072	0.565	0.089	0.024	0.701	0.379	0.565	0.017
UGT73B43	Cytoplasm	0.562	0.421	0.083	0.298	0.054	0.194	0.280	0.225	0.098	0.156
UGT73B44	Cytoplasm	0.572	0.398	0.075	0.301	0.054	0.262	0.313	0.219	0.092	0.109
UGT73CU3	Cytoplasm	0.720	0.386	0.098	0.174	0.107	0.156	0.306	0.168	0.123	0.079
UGT73CX1	Cytoplasm	0.586	0.458	0.050	0.263	0.075	0.163	0.288	0.184	0.086	0.037
UGT73CX2	Cytoplasm	0.547	0.362	0.067	0.293	0.075	0.282	0.310	0.181	0.068	0.055
UGT73CY2	Cytoplasm	0.525	0.406	0.056	0.289	0.079	0.358	0.316	0.267	0.096	0.214
UGT73CY3	Cytoplasm	0.515	0.400	0.055	0.256	0.067	0.346	0.350	0.210	0.083	0.156
UGT73CZ2	Cytoplasm	0.624	0.384	0.136	0.272	0.080	0.240	0.330	0.171	0.077	0.058
UGT74BX1	Cytoplasm	0.628	0.401	0.074	0.287	0.104	0.218	0.355	0.207	0.120	0.025
UGT91AP1	Cytoplasm	0.679	0.278	0.068	0.226	0.065	0.109	0.368	0.173	0.079	0.065
UGT91AQ1	Cytoplasm	0.671	0.325	0.072	0.187	0.056	0.129	0.372	0.235	0.074	0.073
UGT91AR1	Cytoplasm	0.637	0.375	0.058	0.185	0.052	0.107	0.333	0.210	0.059	0.112
Apiose/xylose synthase	Cytoplasm	0.688	0.284	0.055	0.213	0.148	0.065	0.266	0.118	0.153	0.044
QsFucSyn	Cytoplasm	0.742	0.465	0.261	0.124	0.100	0.276	0.174	0.188	0.054	0.041
QsTD	Plastid	0.111	0.112	0.013	0.050	0.121	0.970	0.019	0.066	0.065	0.039
CCL1	Cytoplasm	0.693	0.349	0.025	0.482	0.115	0.126	0.488	0.521	0.146	0.482
CCL2	Peroxisome	0.124	0.180	0.008	0.314	0.101	0.100	0.090	0.052	0.032	0.971
KR1	Cytoplasm	0.724	0.292	0.126	0.266	0.121	0.178	0.202	0.148	0.077	0.074
KR2	Endoplasmic reticulum	0.235	0.050	0.049	0.351	0.283	0.047	0.774	0.180	0.146	0.115
PKS1	Cytoplasm	0.687	0.275	0.044	0.384	0.269	0.088	0.210	0.141	0.154	0.167
PKS2	Cytoplasm	0.709	0.271	0.039	0.393	0.196	0.069	0.220	0.191	0.138	0.147
PKS4	Cytoplasm	0.639	0.317	0.027	0.396	0.141	0.089	0.178	0.244	0.089	0.104
PKS5	Cytoplasm	0.633	0.296	0.035	0.444	0.129	0.073	0.208	0.187	0.061	0.120
PKS3	Cytoplasm	0.659	0.268	0.047	0.418	0.276	0.096	0.199	0.134	0.144	0.126
PKS6	Cytoplasm	0.729	0.284	0.044	0.429	0.198	0.045	0.269	0.164	0.156	0.174
ACT1	Cytoplasm	0.536	0.470	0.056	0.182	0.060	0.219	0.337	0.091	0.052	0.039
ACT2	Cytoplasm	0.523	0.476	0.076	0.230	0.048	0.255	0.346	0.076	0.045	0.039
ACT3	Cytoplasm	0.487	0.495	0.066	0.242	0.060	0.153	0.237	0.105	0.082	0.122

Supplementary Fig. 27. Predicted subcellular localization of saponin biosynthetic genes from *Q. saponaria*. Biosynthetic genes elucidated in[7] and this publication were analysed using DeepLoc-2.0[50] (https://services.healthtech.dtu.dk/services/DeepLoc-2.0/). The localization with the highest score is outlined in bold, though note that DeepLoc-2.0 also incorporates signal peptide analysis to produce the final predicted localizations listed.

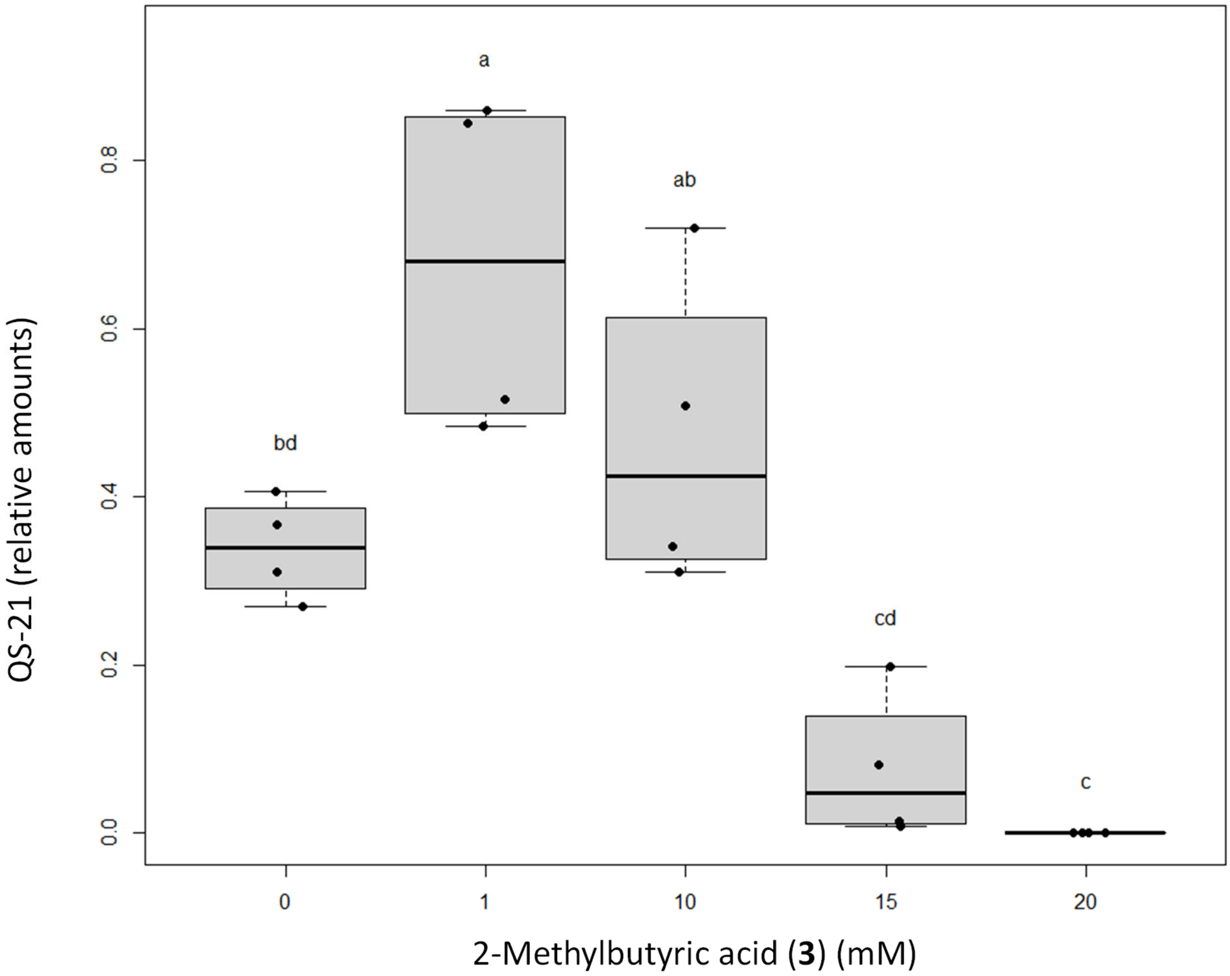

Supplementary Fig. 28. Effect of 2-methylbutyric acid supplementation on QS-21 yield. 2-MB was added at various concentrations to the infiltration buffer containing the Agrobacterium strains to determine whether providing one of the initial substrates of the C_9 unit would result into increased amounts of QS-21. Statistical analyses comprising ANOVA and Tukey tests were performed in R using the multcompView package. The boxplots show the distributions of the values for four biologically independent infiltrated leaf per treatment (represented by the dots), the centre line representing the median, the box showing the lower and upper quartile values and the whiskers representing the minimum and maximum data values. Letters represent significantly different data as determined by the two-sided post-hoc Tukey's HSD ($p = 0.05$) after ANOVA (Df = 4, p Value = $1.52E^{-05}$) using the multcompView package in R.

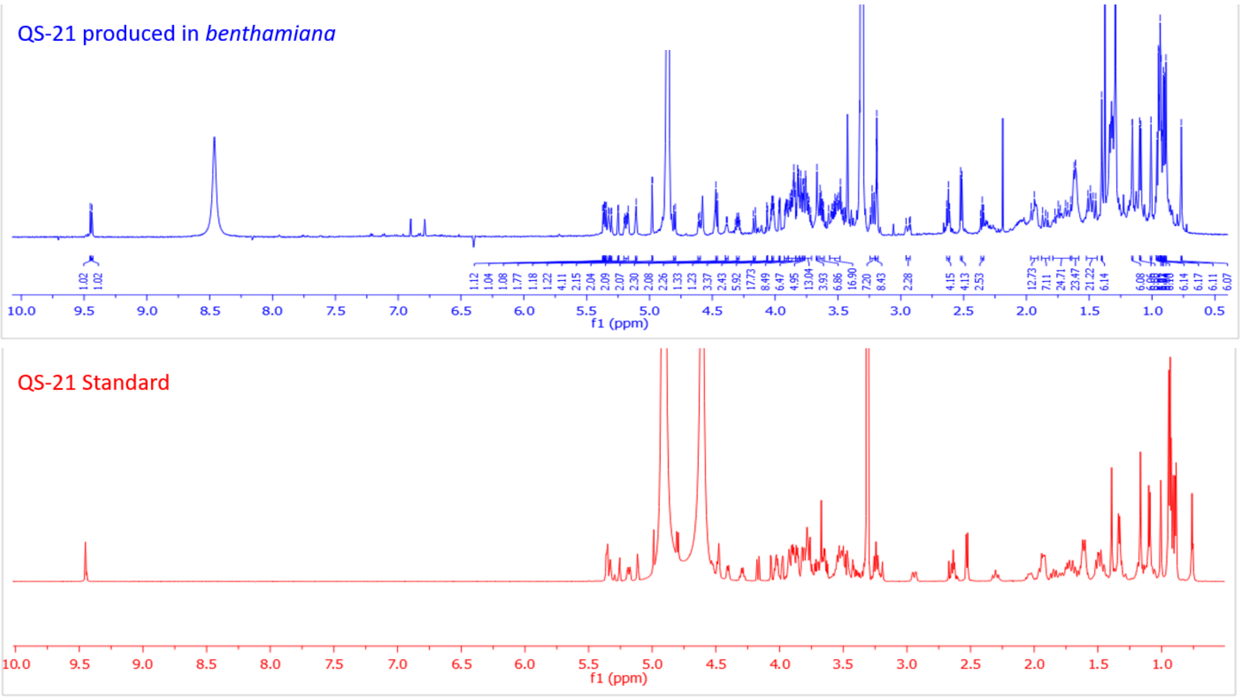

Supplementary Fig. 29. Full 1H NMR spectral comparison between the QS-21 preparation generated by large-scale agro-infiltration of N. benthamiana (blue) and a QS-21 standard (red) (recorded in MeOH-*d*4, 600 MHz).

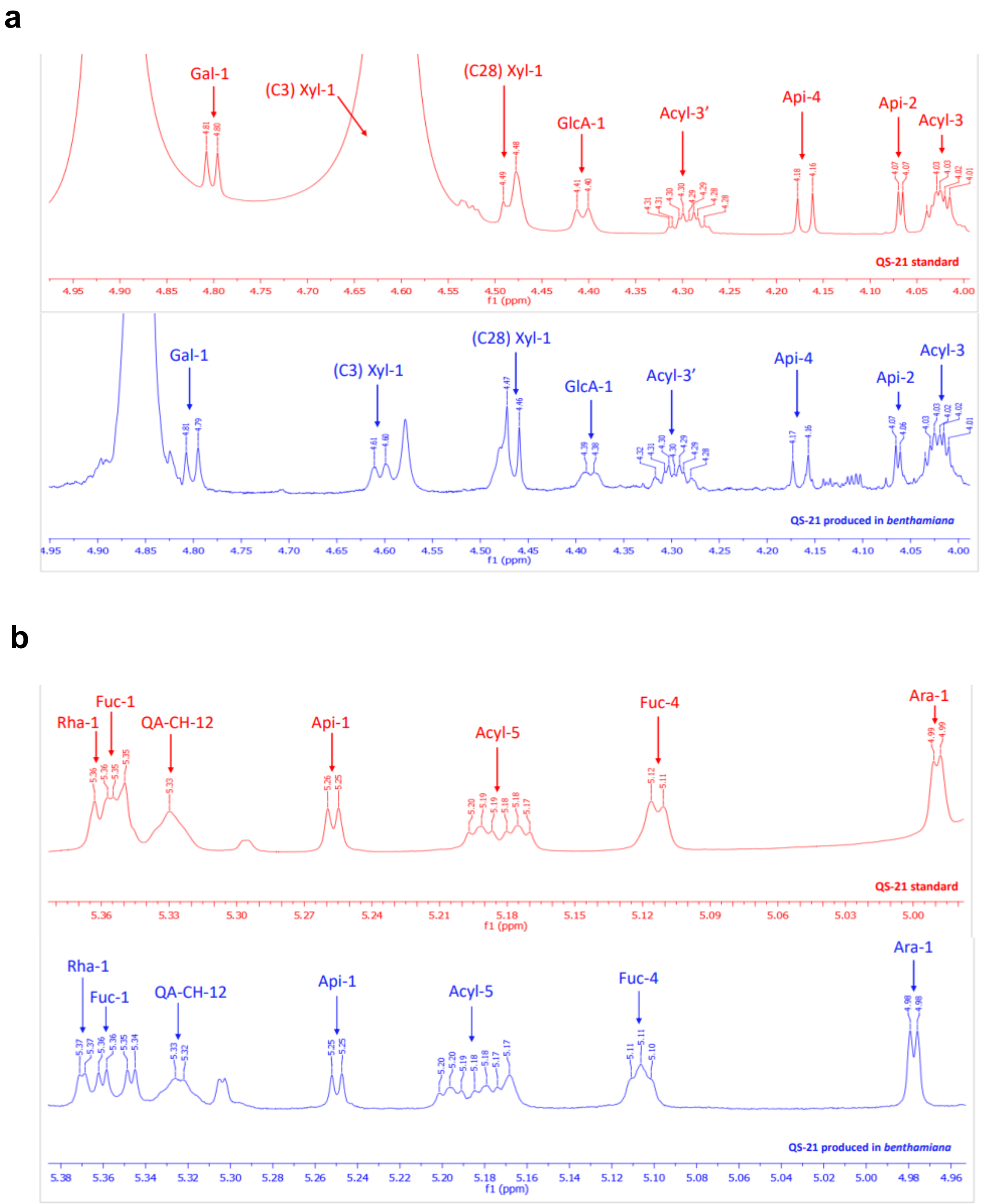

Supplementary Fig. 30. Expanded 1H-NMR spectral comparisons between a commercial QS-21 standard and the partially purified QS-21 produced in *N. benthamiana.* (**a**) 4.00-4.95 ppm; (**b**) 4.96-5.38 ppm, recorded in MeOH-d_4, 600 MHz.

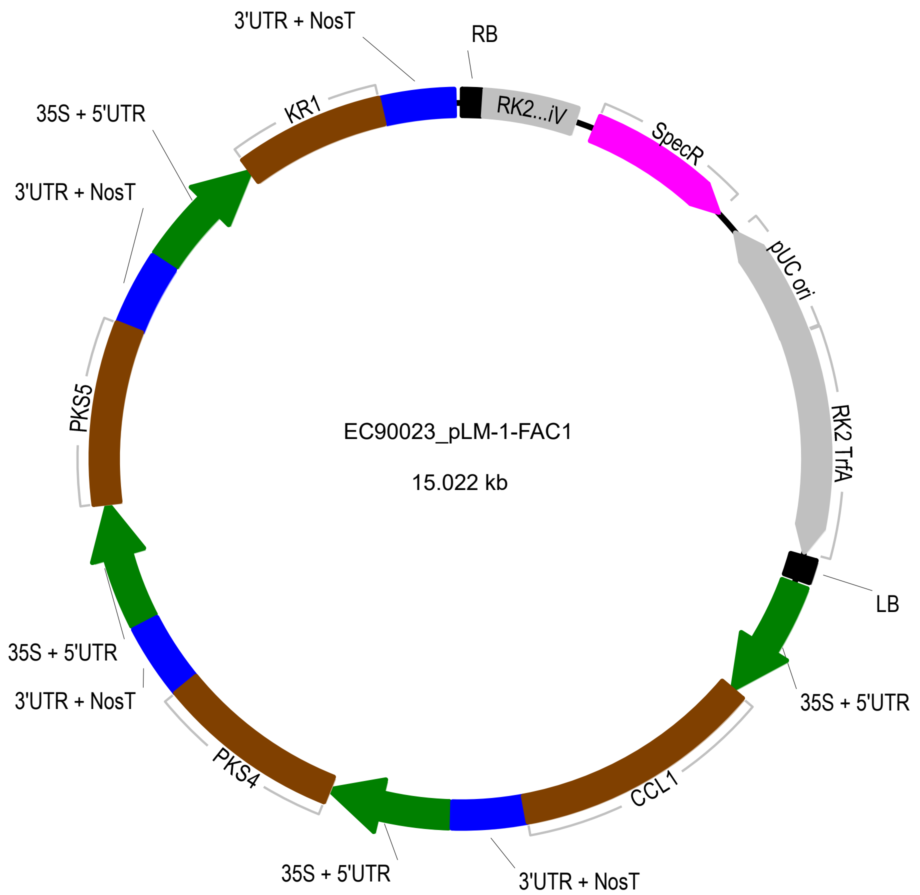

Supplementary Fig. 31. Plasmid map of Golden Gate vector EC90023_pLM-1-FAC1. This construct contains the four genes *CCL1, PKS4, PKS5* and *KR1*, flanked by an upstream module consisting of the cauliflower mosaic virus (CaMV) 35S promoter and modified cowpea mosaic virus (CPMV) 5’ UTR (35S + 5’UTR, green) and downstream by the CPMV 3’ UTR and noscaline synthase terminator (3’UTR + NosT, blue).

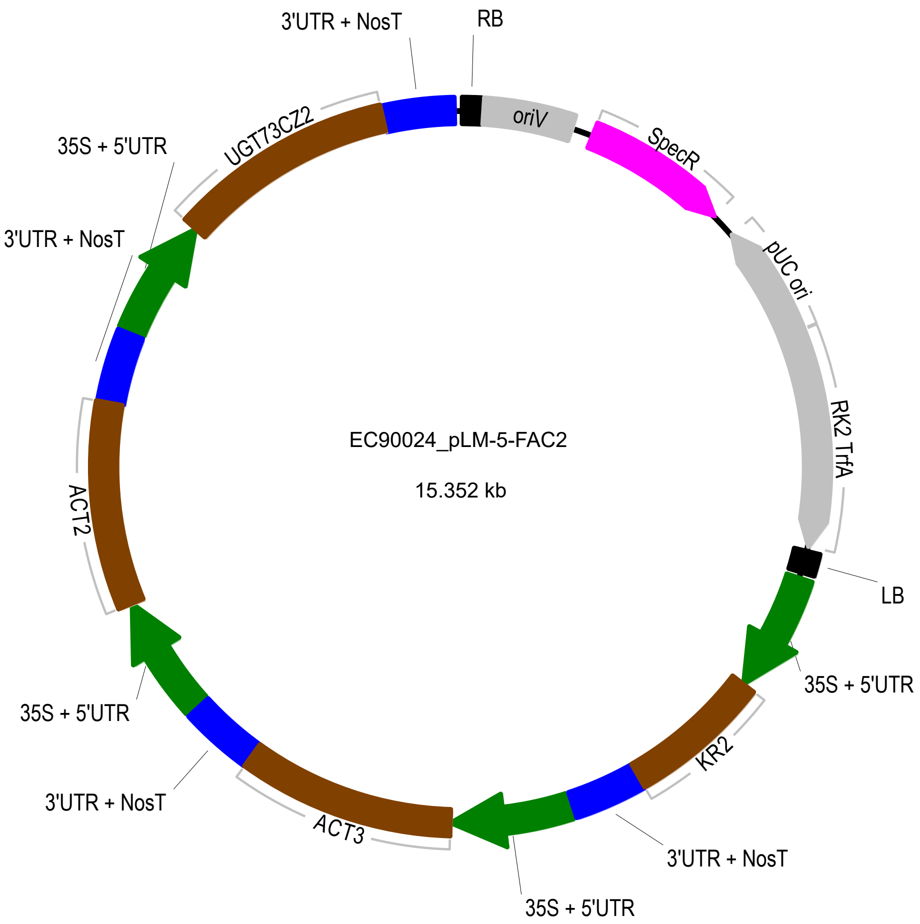

Supplementary Fig. 32. Plasmid map of Golden Gate vector EC90024_pLM-5-FAC2. This contains the four genes *KR2, ACT2, ACT3* and *UGT73CZ2* which are flanked upstream by a module consisting of the cauliflower mosaic virus (CaMV) 35S promoter and modified cowpea mosaic virus (CPMV) 5' UTR (35S + 5'UTR, green) and downstream by the CPMV 3' UTR and noscaline synthase terminator (3'UTR + NosT, blue).

Supplementary tables

Position	QS-21 standard	Product purified from *N. benthamiana*		Literature (26)	
	^{1}H-NMR	^{1}H-NMR	^{13}C-NMR	^{1}H-NMR	^{13}C-NMR
C-23	9.45, s	9.45, s	211.3	9.46	211.4
C_3-GlcA	4.41, d (7.1)	4.39, d (4.7)	104.4	4.45	104.1
C_3-Gal	4.80, d (7.2)	4.80, d (7.5)	103.6	4.81	103.3
C_3-Xyl	Overlapped with water residue	4.61, d (7.1)	104.6	4.59	104.6
C_{28}-Fuc-1	5.36. app d	5.36, d (2.6)	94.7	5.36	94.6
C_{28}-Fuc-4	5.11, d (3.2)	5.11, m	75.1	5.11	75.1
C_{28}-Rha-1	5.37, app d	5.37, d (1.6)	101.5	5.37	101.1
C_{28}-Xyl-1	4.48, d (8.1)	4.47, d (7.8)	107.2	4.47	107.1
C_{28}-Api-1	5.26, d (2.9)	5.25, d (2.8)	110.9	5.25	110.8
C_{28}-Api-2	4.07, d (2.9)	4.06, d (2.7)	77.5	4.06	77.5
C_{28}-Api-4-*a*	4.17, d (9.7)	4.17, d (9.7)	ND	4.17	74.7
Ara-1	4.99, d (1.8)	4.98, d (2)	108.6	4.98	108.3
Ara-2	3.98, dd (4.1, 1.9)	3.97, m	83.5	3.97	83.2
Ara-3	3.85, m	3.87, m	78.1	3.85	78.0
Ara-4	4.03, m	4.03	85.2	4.03	85.1
Ara-5	3.76/3.63	3.75/3.64, m	62.8	3.76/3.63	62.8
Acyl-2	2.64, t (6.9)	2.62, t (6.5)	43.4	2.62	43.4
Acyl-3	4.03, m	4.02, m	66.0	4.02	66.0
Acyl-4	1.85/1.68	1.85.1.68	39.5	1.84/1.67	39.6
Acyl-5	5.18, m	5.18, m	75.1	5.19	75.1
Acyl-6	1.61, m	1.61, m	40.1	1.61	40.0
FAcyl-8	0.93, m	0.94, m	12.2	0.93	12.1
Acyl-9	0.93, m	0.93, m	14.9	0.93	14.9
Acyl-1’	NR	-	173.6 HMBC	-	173.4
Acyl-2’	2.53, d (6.6)	2.52, d (6.6)	43.9	2.52	43.9
Acyl-3’	4.29, m	4.30, m	66.0	4.30	66.0
Acyl-4’	1.63/1.50, m	1.66/1.50	39.3	1.62/1.50	39.2
Acyl-5’	3.80, m	3.80, m	79.2 HMBC	3.80	79.0
Acyl-6’	1.61, m	1.61, m	40.0	1.61	40.0
Acyl-7’	1.61/1.12	1.62/1.12	25.4	1.61/1.12	25.4
Acyl-8’	0.93	0.94	12.2	0.93	12.1
Acyl-9’	0.89	0.89	14.9	0.88	14.9

Supplementary Table 1. 1H, 13C-NMR spectroscopic data comparison (key resonances, retrieved based on HSQC) for the QS-21 standard and the product produced in *N. benthamiana* with the data reported for QS-21 in the literature[27]. All spectra were recorded in MeOH-d_4 (600 MHz). ND: Not detected; NR: Not recorded

Compound	Molecular Formula	Exact Mass	Molecular Weight	Retention Time (min)	MS m/z Fragment 1	MS m/z Fragment 2	MS m/z Fragment 3
QA-TriX-FRXX-C_9	$C_{78}H_{124}O_{39}$	1684.7720	1685.8110	8.3	1511.6548	1553.6653	
QA-TriX-FRXX-C_{18}	$C_{87}H_{140}O_{42}$	1856.8819	1858.0350	10.2	1511.6548	1553.6653	1725.7753
QA-TriX-FRXX-C_{18}-A	$C_{92}H_{148}O_{46}$	1988.9242	1990.9242	9.5	1511.6548	1553.6653	1725.7753

Supplementary Table 2. LC/MS-MS information for the three saponins described in this work.

Profile name	Full name	Pfam ID
2-Hacid_dh_C	D-isomer specific 2-hydroxyacid dehydrogenase, NAD binding domain	PF02826
2-Hacid_dh	D-isomer specific 2-hydroxyacid dehydrogenase, catalytic domain	PF00389
2-oxoacid_dh	2-oxoacid dehydrogenases acyltransferase (catalytic domain)	PF00198
3Beta_HSD	3-beta hydroxysteroid dehydrogenase/isomerase family	PF01073
ACAS_N	Acetyl-coenzyme A synthetase N-terminus	PF16177
ACP_syn_III_C	3-Oxoacyl-[acyl-carrier-protein (ACP)] synthase III C terminal	PF08541
ACP_syn_III	3-Oxoacyl-[acyl-carrier-protein (ACP)] synthase III	PF08545
ADH_N	Alcohol dehydrogenase GroES-like domain	PF08240
ADH_zinc_N	Zinc-binding dehydrogenase	PF00107
Aldo_ket_red	Aldo/keto reductase family	PF00248
Aminotran_4	Amino-transferase class IV	PF01063
AMP-binding_C	AMP-binding enzyme C-terminal domain	PF13193
ATP-grasp_2	ATP-grasp domain	PF08442
ATP-synt_ab_Xtn	ATPsynthase alpha/beta subunit N-term extension	PF16886
Biotin_lipoyl	Biotin-requiring enzyme	PF00364
CDP-OH_P_transf	CDP-alcohol phosphatidyltransferase	PF01066
Citrate_bind	ATP citrate lyase citrate-binding	PF16114
Citrate_synt	Citrate synthase, C-terminal domain	PF00285
DHHC	DHHC palmitoyltransferase	PF01529
E3_binding	e3 binding domain	PF02817
ECH_1	Enoyl-CoA hydratase/isomerase	PF00378
ECH_2	Enoyl-CoA hydratase/isomerase	PF16113
FAE1_CUT1_RppA	FAE1/Type III polyketide synthase-like protein	PF08392
GDP_Man_Dehyd	GDP-mannose 4,6 dehydratase	PF16363
Glyco_hydro_1	Glycosyl hydrolase family 1	PF00232
HMG-CoA_red	Hydroxymethylglutaryl-coenzyme A reductase	PF00368
Ketoacyl-synt_C	Beta-ketoacyl synthase, C-terminal domain	PF02801
ketoacyl-synt	Beta-ketoacyl synthase, N-terminal domain	PF00109
KR	KR domain	PF08659
LCAT	Lecithin:cholesterol acyltransferase	PF02450
Ligase_CoA	CoA-ligase	PF00549
Lipase_GDSL	GDSL-like Lipase/Acylhydrolase	PF00657
LRR_8	Leucine rich repeat	PF13855
LRRNT_2	Leucine rich repeat N-terminal domain	PF08263
MDD_C	Mevalonate 5-diphosphate decarboxylase C-terminal domain	PF18376
NAD_binding_10	NAD(P)H-binding	PF13460
NAD_binding_2	NAD binding domain of 6-phosphogluconate dehydrogenase	PF03446
NAD_binding_4	Male sterility protein	PF07993
NmrA	NmrA-like family	PF05368
Nup188	Nucleoporin subcomplex protein binding to Pom34	PF10487
PALP	Pyridoxal-phosphate dependent enzyme	PF00291
Patatin	Patatin-like phospholipase	PF01734
PK_Tyr_Ser-Thr	Protein tyrosine and serine/threonine kinase	PF07714
Pkinase	Protein kinase domain	PF00069
Polysacc_synt_2	Polysaccharide biosynthesis protein	PF02719
RmlD_sub_bind	RmlD substrate binding domain	PF04321
Sterile	Male sterility protein	PF03015
Thiolase_C	Thiolase, C-terminal domain	PF02803
Thiolase_N	Thiolase, N-terminal domain	PF00108
Thr_dehydrat_C	C-terminal regulatory domain of Threonine dehydratase	PF00585

Supplementary Table 3. Additional pHHMs used in plantiSMASH analysis of *Q. saponaria* genome

Name	Sequence
Qs0095300_attB1F	GGGGACAAGTTTGTACAAAAAAGCAGGCTTAATGGATCAATTAAATCCAAAGCCTGTAAAC
Qs0095300_attB2R	GGGGACCACTTTGTACAAGAAAGCTGGGTATTACAACCGACTAGCTGTCAACG
Qs0012180_attB1F	GGGGACAAGTTTGTACAAAAAAGCAGGCTTAATGGAAGATCTTAAGCCAAGCCCAG
Qs0012180_attB2R	GGGGACCACTTTGTACAAGAAAGCTGGGTATCACATCCGACTCGATCTTAGTGGG
CCL1_attB1F	GGGGACAAGTTTGTACAAAAAAGCAGGCTTAATGGAAGATCTTAAACCAAGCTCTGC
CCL1_attB2R	GGGGACCACTTTGTACAAGAAAGCTGGGTACTATGTGTGGTTGGAGAGAGGC
CCL2_attB1F	GGGGACAAGTTTGTACAAAAAAGCAGGCTTAATGGAAAACCTTAAGCCAACCCC
CCL2_attB2R	GGGGACCACTTTGTACAAGAAAGCTGGGTATCACATCCGACTCGACCTTGG
Qs0229950_attB1F	GGGGACAAGTTTGTACAAAAAAGCAGGCTTAATGGAGCAACTCAACCCAAAACC
Qs0229950_attB2R	GGGGACCACTTTGTACAAGAAAGCTGGGTACTACAACCGACTATCTGTCAACGACC
Qs0295340_attB1F	GGGGACAAGTTTGTACAAAAAAGCAGGCTTAATGGAGCAACTCAACCCAAAACC
Qs0295340_attB2R	GGGGACCACTTTGTACAAGAAAGCTGGGTACTACAACCGACTATCTGTCAACGACC
PKS1_attB1F	GGGGACAAGTTTGTACAAAAAAGCAGGCTTAATGGTGACTGTGGAGGAAGTTCG
PKS1_attB2R	GGGGACCACTTTGTACAAGAAAGCTGGGTATTAAGCAGACACGCTATGAAGCAC
PKS2_attB1F	GGGGACAAGTTTGTACAAAAAAGCAGGCTTAATGGCATCCATCGAAGCAATCC
PKS2_attB2R	GGGGACCACTTTGTACAAGAAAGCTGGGTATTAATTGGTTTCAACCGGAACACTG
PKS3_attB1F	GGGGACAAGTTTGTACAAAAAAGCAGGCTTAATGGTGACTGTGGAGGAAGTTCG
PKS3_attB2R	GGGGACCACTTTGTACAAGAAAGCTGGGTATTAGGCAGCCAGACTATGAAGCAC
PKS4_attB1F	GGGGACAAGTTTGTACAAAAAAGCAGGCTTAATGGCTCCGGTGACCAACAC
PKS4_attB2R	GGGGACCACTTTGTACAAGAAAGCTGGGTATTACACAACTGGGACACTGCGC
PKS5_attB1F	GGGGACAAGTTTGTACAAAAAAGCAGGCTTAATGGGATCCTTGGATCAAGATTTTC
PKS5_attB2R	GGGGACCACTTTGTACAAGAAAGCTGGGTATTAATGGGCCTCATTTGTAGAAATGC
PKS6_attB1F	GGGGACAAGTTTGTACAAAAAAGCAGGCTTAATGGCATCCGTCGAAGCAATC
PKS6_attB2R	GGGGACCACTTTGTACAAGAAAGCTGGGTATCAATAACTTATCGGAACACTGTGCAAC
KR1_attB1F	GGGGACAAGTTTGTACAAAAAAGCAGGCTTAATGGCGGCGGCCGAAAGTGAAAG
KR1_attB2R	GGGGACCACTTTGTACAAGAAAGCTGGGTACTAGTTAGTTTCGAGAACTCCTGCCTTTCGATAGC
KR2_attB1F	GGGGACAAGTTTGTACAAAAAAGCAGGCTTAATGGAATGCTGTATAGTAGCTAGGCTAAAAACC
KR2_attB2R	GGGGACCACTTTGTACAAGAAAGCTGGGTATTATTCTTTCTTCCTTGCATCTTTAAGCTGCC
ACT2_attB1F	GGGGACAAGTTTGTACAAAAAAGCAGGCTTAATGATGGAGGTACATACCACATCGG
ACT2_attB2R	GGGGACCACTTTGTACAAGAAAGCTGGGTATCAAATGATAGTGGGATTAACCATGGCAG
ACT3_attB1F	GGGGACAAGTTTGTACAAAAAAGCAGGCTTAATGGCTGAAGCTTTGAAATTGAAAGTAGTTG
ACT3_attB2R	GGGGACCACTTTGTACAAGAAAGCTGGGTATTAGAGTATCTTCAATGTTTGATCAAATGTCTGAATG
UGT73CZ2_attB1F	GGGGACAAGTTTGTACAAAAAAGCAGGCTTAATGCCATTCATTCGTAATACTCC
UGT73CZ2_attB2R	GGGGACCACTTTGTACAAGAAAGCTGGGTATCATTCCTGCGTGCTAGTTG
QsTD_0222940_attB1F	GGGGACAAGTTTGTACAAAAAAGCAGGCTTAATGGAGACACTGTGTTACTCGCCATC
QsTD_0222940_attB2R	GGGGACCACTTTGTACAAGAAAGCTGGGTATTAATGCATCAAAAGCTGGAAATCTTCATC
QsTD_P540L_Q5F	TTCATCTTTCtGGAGAGGCCTG
QsTD_P540L_Q5R	ACGGCAAAGAACCTCATTC

Supplementary Table 4. Primer sequences used to clone the candidate genes for yeast and *N. benthamiana* expression. Gene specific sequences are shown in black, while the attB sites required for Gateway® cloning are shown in grey.

Analyte	MRM transitions	CV [V]	CE [V]
CoA-SH	768 → 428<br>768 → 261	70<br>70	30<br>30
2-Methylbutyryl-CoA	852 → 428<br>852 → 345	68<br>68	26<br>32
Isovaleryl-CoA	852 → 428<br>852 → 345	66<br>66	24<br>32
Isobutyryl-CoA	838 → 428<br>838 → 331	70<br>70	26<br>32
Malonyl-CoA	854 → 428<br>854 → 347	62<br>62	28<br>30
C_9-δ-lactone<br>Triketide pyrone	169 → 139<br>169 → 123	26<br>26	18<br>16

Supplementary Table 5. MRM transitions of acyl-CoA standards in ESI positive mode. Cone voltage (CV) and collision energy (CE) were individually tuned for each analyte using IntelliStart.

Analyte	MRM transitions	CV [V]	CE [V]
CoA-SH	766 → 408<br>766 → 159	40<br>40	32<br>56
2-Methylbutyryl-CoA	850 → 503<br>850 → 426	74<br>74	34<br>34
Isovaleryl-CoA	850 → 408<br>850 → 159	80<br>80	38<br>60
Isobutyryl-CoA	836 → 489<br>836 → 426	50<br>50	34<br>36
Malonyl-CoA	852 → 408<br>852 → 159	44<br>44	34<br>59
C_9-δ-lactone<br>Triketide pyrone	167 → 122<br>169 → 96	6<br>6	14<br>22

Supplementary Table 6. MRM transitions of acyl-CoA standards in ESI negative mode. Cone voltage (CV) and collision energy (CE) were individually tuned for each analyte using IntelliStart.

Gene name	Primer name	Primer sequence 5'-3'
PKS1	EAQ-QsPKS1_FW	attctgcccaaattcgATGgtgactgtggag
	EAQ-QsPKS1-His_RV	tgatgcataccggtcgTCAGTGGTGGTGGTGGTGGTG**CTCGAG**agcagacacgctatg
PKS3	EAQ-QsPKS3_FW	attctgcccaaattcgATGgtgactgtggag
	EAQ-QsPKS3-His_RV	tgatgcataccggtcgTCAGTGGTGGTGGTGGTGGTG**CTCGAG**ggcagccagactatg
PKS4	EAQ-QsPKS4_FW	attctgcccaaattcgATGgctccggtgacc
	EAQ-QsPKS4-His_RV	tgatgcataccggtcgTCAGTGGTGGTGGTGGTGGTG**CTCGAG**cacaactgggacact
PKS5	EAQ-QsPKS5_FW	attctgcccaaattcgATGggatccttggat
	EAQ-QsPKS5-His_RV	tgatgcataccggtcgTCAGTGGTGGTGGTGGTGGTG**CTCGAG**atgggcctcatttgt
PKS6	EAQ-QsPKS6_FW	attctgcccaaattcgATGgcatccgtcgaa
	EAQ-QsPKS6-His_RV	tgatgcataccggtcgTCAGTGGTGGTGGTGGTGGTG**CTCGAG**ataacttatcggaac
UGT73CZ2	EAQ-QsUGT73CZ2_FW	attctgcccaaattcgATGccattcattcgt
	EAQ-UGT73CZ2-His_RV	tgatgcataccggtcgTCAGTGGTGGTGGTGGTGGTGttcctgcgtgctagt

Supplementary Table 7. Primer sequences used to make His-tagged proteins for *in vitro* analysis. Uppercase: initiation codon, 6x histidine tag (underlined), XhoI site (bold), and termination codon.

**Amino acid**	**Parent mass**	**Daughter ions**	
Valine	288	170.58	145.16
Leucine	302	170.48	144.78
Isoleucine	302	170.58	145.16
Threonine	290	170.58	145.16

Supplementary Table 8. MRM transitions of amino acid standards in positive ESI mode

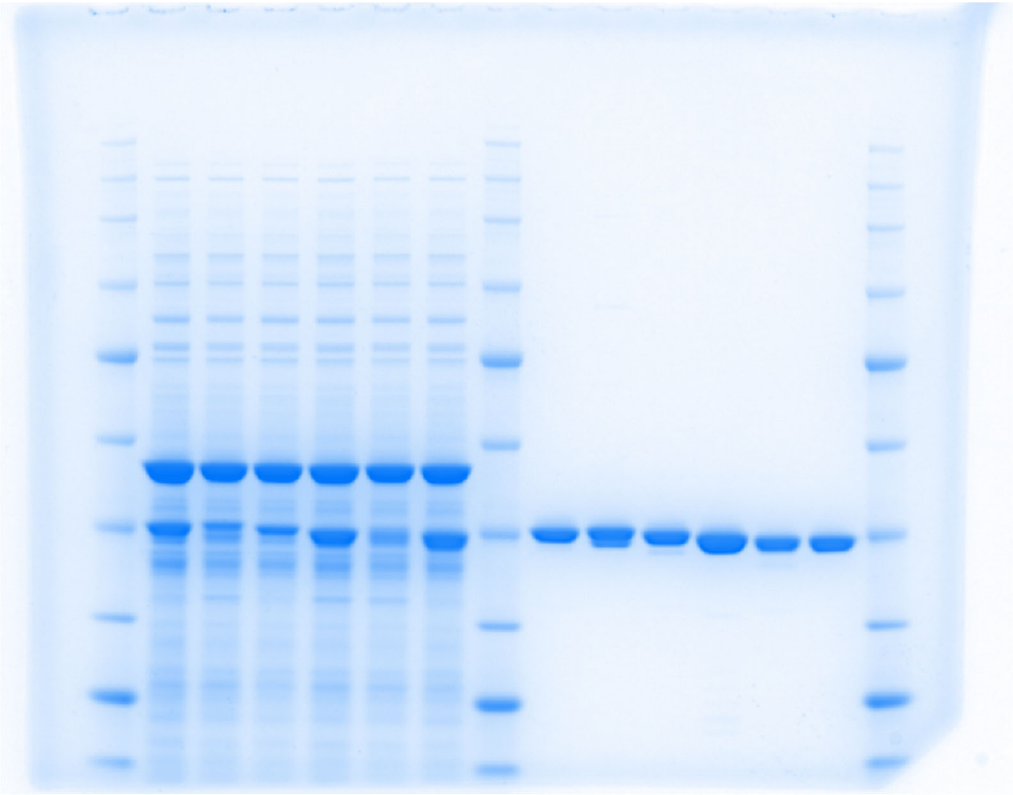

Source Data Supplementary Fig 7

Supplementary Information References

47 Shockey, J. M., Fulda, M. S. & Browse, J. Arabidopsis contains a large superfamily of acyl-activating enzymes. Phylogenetic and biochemical analysis reveals a new class of acyl-coenzyme a synthetases. *Plant Physiol.* **132**, 1065–1076 (2003).

48 Moummou, H., Kallberg, Y., Tonfack, L. B., Persson, B. & van der Rest, B. The plant short-chain dehydrogenase (SDR) superfamily: genome-wide inventory and diversification patterns. *BMC Plant Biol.* **12**, 219 (2012).

49 Kallberg, Y., Oppermann, U. & Persson, B. Classification of the short-chain dehydrogenase/reductase superfamily using hidden Markov models. *FEBS J.* **277**, 2375–2386 (2010).

50 Thumuluri, V., Almagro Armenteros, J. J., Johansen, A. R., Nielsen, H. & Winther, O. DeepLoc 2.0: multi-label subcellular localization prediction using protein language models. *Nucleic Acids Res.* **50**, W228–W234 (2022).
