# Supplementary material for: Complete biosynthesis of the potent vaccine adjuvant QS-21
Source: Nat Chem Biol. 2024 Jan 26;20(4):493–502. doi: 10.1038/s41589-023-01538-5 (PMC10972754; doi:10.1038/s41589-023-01538-5)
Supplement: Supplementary file 2 — Reporting Summary [file 41589_2023_1538_MOESM2_ESM.pdf]

nature portfolio

Corresponding author(s): Anne Osbourn

Last updated by author(s): 2023/11/28

Reporting Summary

Nature Portfolio wishes to improve the reproducibility of the work that we publish. This form provides structure for consistency and transparency in reporting. For further information on Nature Portfolio policies, see our Editorial Policies and the Editorial Policy Checklist.

Statistics

For all statistical analyses, confirm that the following items are present in the figure legend, table legend, main text, or Methods section.

n/a	Confirmed	
☐	☒	The exact sample size (*n*) for each experimental group/condition, given as a discrete number and unit of measurement
☐	☒	A statement on whether measurements were taken from distinct samples or whether the same sample was measured repeatedly
☐	☒	The statistical test(s) used AND whether they are one- or two-sided<br>*Only common tests should be described solely by name; describe more complex techniques in the Methods section.*
☒	☐	A description of all covariates tested
☒	☐	A description of any assumptions or corrections, such as tests of normality and adjustment for multiple comparisons
☐	☒	A full description of the statistical parameters including central tendency (e.g. means) or other basic estimates (e.g. regression coefficient) AND variation (e.g. standard deviation) or associated estimates of uncertainty (e.g. confidence intervals)
☐	☒	For null hypothesis testing, the test statistic (e.g. *F*, *t*, *r*) with confidence intervals, effect sizes, degrees of freedom and *P* value noted<br>*Give P values as exact values whenever suitable.*
☒	☐	For Bayesian analysis, information on the choice of priors and Markov chain Monte Carlo settings
☒	☐	For hierarchical and complex designs, identification of the appropriate level for tests and full reporting of outcomes
☒	☐	Estimates of effect sizes (e.g. Cohen's *d*, Pearson's *r*), indicating how they were calculated

Our web collection on statistics for biologists contains articles on many of the points above.

Software and code

Policy information about availability of computer code

Data collection	Masslynx 4.1 (which includes the IntelliStar function); Xcalibur 4.3; plantiSMASH 1.0
Data analysis	InterProScan 5.22; MAFFT 7.127; RaxML 2.1.9; Masslynx 4.1; FreeStyle 1.6; plantiSMASH 1.0; HMMER-3.1b2

For manuscripts utilizing custom algorithms or software that are central to the research but not yet described in published literature, software must be made available to editors and reviewers. We strongly encourage code deposition in a community repository (e.g. GitHub). See the Nature Portfolio guidelines for submitting code & software for further information.

Data

Policy information about availability of data

All manuscripts must include a data availability statement. This statement should provide the following information, where applicable:

- Accession codes, unique identifiers, or web links for publicly available datasets
- A description of any restrictions on data availability
- For clinical datasets or third party data, please ensure that the statement adheres to our policy

Transcriptome and genome sequence data for Q. saponaria was previously reported in Reed et al. (7) submitted under NCBI BioProject IDs PRJNA914309 (SRA accessions SRR22829626 - SRR22829649) and PRJNA914519. The sequences of the genes characterized in the current study have been deposited in GenBank as the following: CCL1 (Qs0229930), OQ241430; CCL2 (Qs0216480), OQ241421; PKS1 (Qs0007680), OQ241431; PKS2 (Qs0170050), OQ241424; PKS3 (Qs0181340), OQ241419; PKS4 (Qs0268330), OQ241427; PKS5 (Qs0268880), OQ241428; PKS6 (Qs0285490), OQ241422; KR1 (Qs0326850), OQ241429; KR2 (Qs0235370),

OQ241432; ACT2 (Qs0322030), OQ241420; ACT3 (Qs0264740), OQ241426; UGT73CZ2 (Qs0131010), OQ241425; Threonine deaminase (TD) (Qs0222940), OQ241423; Feedback insensitive threonine deaminase (TD-P540L) (Qs0222940_P540L), OQ241433.

The databases used in this study are SciFinder® Login (scifinder.cas.org), Reaxys.com, sdr.enzymes.org, InterPro-85.0 (https://www.ebi.ac.uk/interpro/); Pfam-33.1 (http://pfam.xfam.org/).

Human research participants

Policy information about studies involving human research participants and Sex and Gender in Research.

Reporting on sex and gender	n/a
Population characteristics	n/a
Recruitment	n/a
Ethics oversight	n/a

Note that full information on the approval of the study protocol must also be provided in the manuscript.

Field-specific reporting

Please select the one below that is the best fit for your research. If you are not sure, read the appropriate sections before making your selection.

☒ Life sciences ☐ Behavioural & social sciences ☐ Ecological, evolutionary & environmental sciences

For a reference copy of the document with all sections, see nature.com/documents/nr-reporting-summary-flat.pdf

Life sciences study design

All studies must disclose on these points even when the disclosure is negative.

Sample size	Sample sizes were as big as humanely manageable. Three or four replicates per sample is routine in biology.
Data exclusions	No data were excluded from the analyses
Replication	Details of biological/technical replicates are provided in the relevant figure legends, methods and supplementary information sections of the manuscript.
Randomization	Not applicable as samples were all under the same conditions.
Blinding	Not applicable as machines were used to generate data.

Reporting for specific materials, systems and methods

We require information from authors about some types of materials, experimental systems and methods used in many studies. Here, indicate whether each material, system or method listed is relevant to your study. If you are not sure if a list item applies to your research, read the appropriate section before selecting a response.

Materials & experimental systems

n/a	Involved in the study	
☒	☐	Antibodies
☒	☐	Eukaryotic cell lines
☒	☐	Palaeontology and archaeology
☒	☐	Animals and other organisms
☒	☐	Clinical data
☒	☐	Dual use research of concern

Methods

n/a	Involved in the study	
☒	☐	ChIP-seq
☒	☐	Flow cytometry
☒	☐	MRI-based neuroimaging
