# Supplementary material for: Complete biosynthesis of the potent vaccine adjuvant QS-21
Source: Nat Chem Biol. 2024 Jan 26;20(4):493–502. doi: 10.1038/s41589-023-01538-5 (PMC10972754; doi:10.1038/s41589-023-01538-5)

Supplementary Data 3

CAS SciFinder Search Output

CAS SciFinder^n

Task History

Initiating Search

July 7, 2022, 7:05PM

Substances:

Filtered By:

Stereochemistry: **Absolute Stereo Match**

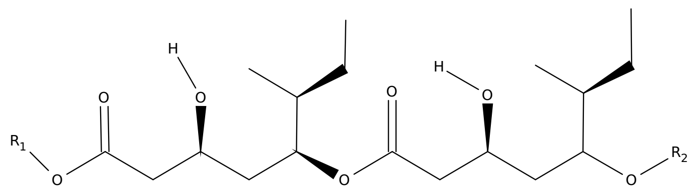

Structure Match: **Substructure**

R-Groups
R1: **A , Ak , Cb , Cy**
R2: **A , Ak , Cb , Cy**

Search Tasks

Task	Search Type	View
Exported: Returned Substance Results + Filters	Substances	View Results

Copyright © 2022 American Chemical Society (ACS). All Rights Reserved.
Internal use only. Redistribution is subject to the terms of your SciFinder^n License Agreement and CAS information Use Policies.

CAS SciFinderⁿ

Substances (60)

View in SciFinderⁿ

1

250643-56-2

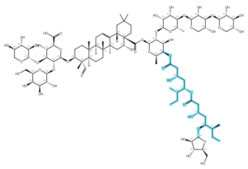

Absolute stereochemistry shown

$C_{92}H_{148}O_{46}$

(3β,4α,16α)-16-Hydroxy-23,28-dioxo-28-[[*O*-β-D-xylopyranosyl-(1→3)-*O*-β-D-xylopyranosyl-(1→4)-*O*-6-deoxy-α-L-mannopyranosyl-(1→2)-4-*O*-[(3*S*,5*S*,6*S*)-5-[[(3*S*,5*S*,6*S*)-5-(α-L-arabinofuranosyloxy)-3-hydroxy-6-methyl-1-oxooctyl]oxy]-3-hydroxy-6-methyl-1-oxooctyl]-6-deoxy-β-D-galactopyranosyl]oxy]olean-12-en-3-y...

35 References | 185 Reactions | 0 Suppliers

2

154335-27-0

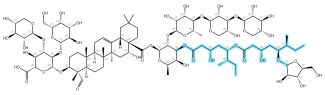

Absolute stereochemistry shown

$C_{92}H_{148}O_{46}$

(3β,4α,16α)-16-Hydroxy-23,28-dioxo-28-[[*O*-β-D-xylopyranosyl-(1→3)-*O*-β-D-xylopyranosyl-(1→4)-*O*-6-deoxy-α-L-mannopyranosyl-(1→2)-3-*O*-[(3*S*,5*S*,6*S*)-5-[[(3*S*,5*S*,6*S*)-5-(α-L-arabinofuranosyloxy)-3-hydroxy-6-methyl-1-oxooctyl]oxy]-3-hydroxy-6-methyl-1-oxooctyl]-6-deoxy-β-D-galactopyranosyl]oxy]olean-12-en-3-y...

24 References | 2 Reactions | 0 Suppliers

3

325966-83-4

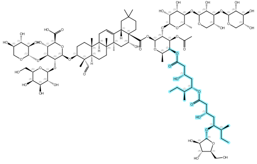

Absolute stereochemistry shown

$C_{94}H_{150}O_{47}$

(3β,4α,16α)-16-Hydroxy-23,28-dioxo-28-[[*O*-β-D-xylopyranosyl-(1→3)-*O*-β-D-xylopyranosyl-(1→4)-*O*-6-deoxy-α-L-mannopyranosyl-(1→2)-3-*O*-acetyl-4-*O*-[(3*S*,5*S*,6*S*)-5-[[(3*S*,5*S*,6*S*)-5-(α-L-arabinofuranosyloxy)-3-hydroxy-6-methyl-1-oxooctyl]oxy]-3-hydroxy-6-methyl-1-oxooctyl]-6-deoxy-β-D-galactopyranosyl]oxy]olea...

5 References | 0 Reactions | 0 Suppliers

4

325966-81-2

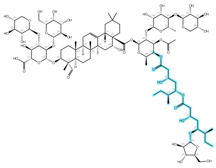

Absolute stereochemistry shown

$C_{89}H_{142}O_{43}$

(3β,4α,16α)-16-Hydroxy-23,28-dioxo-28-[[*O*-β-D-xylopyranosyl-(1→4)-*O*-6-deoxy-α-L-mannopyranosyl-(1→2)-3-*O*-acetyl-4-*O*-[(3*S*,5*S*,6*S*)-5-[[(3*S*,5*S*,6*S*)-5-(α-L-arabinofuranosyloxy)-3-hydroxy-6-methyl-1-oxooctyl]oxy]-3-hydroxy-6-methyl-1-oxooctyl]-6-deoxy-β-D-galactopyranosyl]oxy]olean-12-en-3-yl *O*-β-D-galacto...

5 References | 0 Reactions | 0 Suppliers

5

250643-54-0

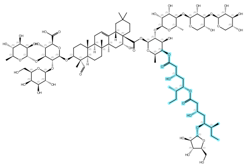

Absolute stereochemistry shown

$C_{93}H_{150}O_{46}$

(3β,4α,16α)-16-Hydroxy-23,28-dioxo-28-[[*O*-β-D-xylopyranosyl-(1→3)-*O*-β-D-xylopyranosyl-(1→4)-*O*-6-deoxy-α-L-mannopyranosyl-(1→2)-4-*O*-[(3*S*,5*S*,6*S*)-5-[[(3*S*,5*S*,6*S*)-5-(α-L-arabinofuranosyloxy)-3-hydroxy-6-methyl-1-oxooctyl]oxy]-3-hydroxy-6-methyl-1-oxooctyl]-6-deoxy-β-D-galactopyranosyl]oxy]olean-12-en-3-y...

5 References | 0 Reactions | 0 Suppliers

6

250643-52-8

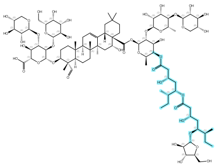

Absolute stereochemistry shown

$C_{87}H_{140}O_{42}$

(3β,4α,16α)-16-Hydroxy-23,28-dioxo-28-[[*O*-β-D-xylopyranosyl-(1→4)-*O*-6-deoxy-α-L-mannopyranosyl-(1→2)-4-*O*-[(3*S*,5*S*,6*S*)-5-[[(3*S*,5*S*,6*S*)-5-(α-L-arabinofuranosyloxy)-3-hydroxy-6-methyl-1-oxooctyl]oxy]-3-hydroxy-6-methyl-1-oxooctyl]-6-deoxy-β-D-galactopyranosyl]oxy]olean-12-en-3-yl *O*-β-D-galactopyranosyl-(...

5 References | 0 Reactions | 0 Suppliers

7

250643-50-6

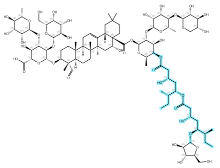

Absolute stereochemistry shown

$C_{88}H_{142}O_{42}$

(3β,4α,16α)-16-Hydroxy-23,28-dioxo-28-[[*O*-β-D-xylopyranosyl-(1→4)-*O*-6-deoxy-α-L-mannopyranosyl-(1→2)-4-*O*-[(3*S*,5*S*,6*S*)-5-[[(3*S*,5*S*,6*S*)-5-(α-L-arabinofuranosyloxy)-3-hydroxy-6-methyl-1-oxooctyl]oxy]-3-hydroxy-6-methyl-1-oxooctyl]-6-deoxy-β-D-galactopyranosyl]oxy]olean-12-en-3-yl *O*-6-deoxy-α-L-mannopyran...

5 References | 0 Reactions | 0 Suppliers

8

836632-62-3

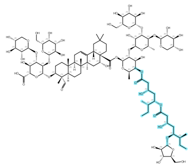

Absolute stereochemistry shown

$C_{93}H_{150}O_{47}$

(3β,4α,16α)-28-[[*O*-β-D-Glucopyranosyl-(1→3)-*O*-[β-D-xylopyranosyl-(1→4)]-*O*-6-deoxy-α-L-mannopyranosyl-(1→2)-4-*O*-[(3*S*,5*S*,6*S*)-5-[[(3*S*,5*S*,6*S*)-5-(α-L-arabinofuranosyloxy)-3-hydroxy-6-methyl-1-oxooctyl]oxy]-3-hydroxy-6-methyl-1-oxooctyl]-6-deoxy-β-D-galactopyranosyl]oxy]-16-hydroxy-23,28-dioxoolean-12-en-...

4 References | 0 Reactions | 0 Suppliers

9

836632-61-2

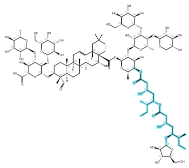

Absolute stereochemistry shown

$C_{94}H_{152}O_{47}$

(3β,4α,16α)-28-[[*O*-β-D-Glucopyranosyl-(1→3)-*O*-[β-D-xylopyranosyl-(1→4)]-*O*-6-deoxy-α-L-mannopyranosyl-(1→2)-4-*O*-[(3*S*,5*S*,6*S*)-5-[[(3*S*,5*S*,6*S*)-5-(α-L-arabinofuranosyloxy)-3-hydroxy-6-methyl-1-oxooctyl]oxy]-3-hydroxy-6-methyl-1-oxooctyl]-6-deoxy-β-D-galactopyranosyl]oxy]-16-hydroxy-23,28-dioxoolean-12-en-...

4 References | 0 Reactions | 0 Suppliers

10

325966-82-3

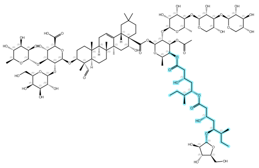

Absolute stereochemistry shown

$C_{95}H_{152}O_{47}$

(3β,4α,16α)-16-Hydroxy-23,28-dioxo-28-[[*O*-β-D-xylopyranosyl-(1→3)-*O*-β-D-xylopyranosyl-(1→4)-*O*-6-deoxy-α-L-mannopyranosyl-(1→2)-3-*O*-acetyl-4-*O*-[(3*S*,5*S*,6*S*)-5-[[(3*S*,5*S*,6*S*)-5-(α-L-arabinofuranosyloxy)-3-hydroxy-6-methyl-1-oxooctyl]oxy]-3-hydroxy-6-methyl-1-oxooctyl]-6-deoxy-β-D-galactopyranosyl]oxy]olea...

4 References | 0 Reactions | 0 Suppliers

11

325966-80-1

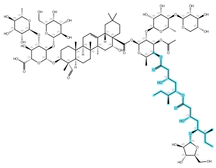

Absolute stereochemistry shown

$C_{90}H_{144}O_{43}$

(3β,4α,16α)-16-Hydroxy-23,28-dioxo-28-[[*O*-β-D-xylopyranosyl-(1→4)-*O*-6-deoxy-α-L-mannopyranosyl-(1→2)-3-*O*-acetyl-4-*O*-[(3*S*,5*S*,6*S*)-5-[[(3*S*,5*S*,6*S*)-5-(α-L-arabinofuranosyloxy)-3-hydroxy-6-methyl-1-oxooctyl]oxy]-3-hydroxy-6-methyl-1-oxooctyl]-6-deoxy-β-D-galactopyranosyl]oxy]olean-12-en-3-yl *O*-6-deoxy-α-L...

4 References | 0 Reactions | 0 Suppliers

12

250643-59-5

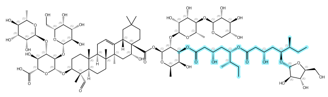

Absolute stereochemistry shown

$C_{88}H_{142}O_{42}$

(3β,4α,16α)-16-Hydroxy-23,28-dioxo-28-[[*O*-β-D-xylopyranosyl-(1→4)-*O*-6-deoxy-α-L-mannopyranosyl-(1→2)-3-*O*-[(3*S*,5*S*,6*S*)-5-[[(3*S*,5*S*,6*S*)-5-(α-L-arabinofuranosyloxy)-3-hydroxy-6-methyl-1-oxooctyl]oxy]-3-hydroxy-6-methyl-1-oxooctyl]-6-deoxy-β-D-galactopyranosyl]oxy]olean-12-en-3-yl *O*-6-deoxy-α-L-mannopyran...

4 References | 0 Reactions | 0 Suppliers

13

836632-64-5

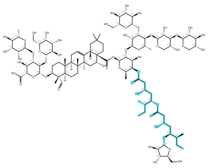

Absolute stereochemistry shown

$C_{99}H_{160}O_{51}$

(3β,4α,16α)-16-Hydroxy-23,28-dioxo-28-[[*O*-β-D-xylopyranosyl-(1→3)-*O*-β-D-xylopyranosyl-(1→4)-*O*-[β-D-glucopyranosyl-(1→3)]-*O*-6-deoxy-α-L-mannopyranosyl-(1→2)-4-*O*-[(3*S*,5*S*,6*S*)-5-[[(3*S*,5*S*,6*S*)-5-(α-L-arabinofuranosyloxy)-3-hydroxy-6-methyl-1-oxooctyl]oxy]-3-hydroxy-6-methyl-1-oxooctyl]-6-deoxy-β-D-galacto...

3 References | 0 Reactions | 0 Suppliers

14

836632-60-1

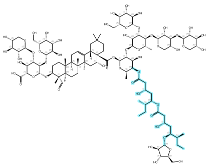

Absolute stereochemistry shown

$C_{98}H_{158}O_{51}$

(3β,4α,16α)-16-Hydroxy-23,28-dioxo-28-[[*O*-β-D-xylopyranosyl-(1→3)-*O*-β-D-xylopyranosyl-(1→4)-*O*-[β-D-glucopyranosyl-(1→3)]-*O*-6-deoxy-α-L-mannopyranosyl-(1→2)-4-*O*-[(3*S*,5*S*,6*S*)-5-[[(3*S*,5*S*,6*S*)-5-(α-L-arabinofuranosyloxy)-3-hydroxy-6-methyl-1-oxooctyl]oxy]-3-hydroxy-6-methyl-1-oxooctyl]-6-deoxy-β-D-galacto...

3 References | 0 Reactions | 0 Suppliers

15

263259-79-6

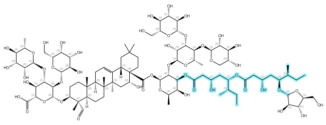

Absolute stereochemistry shown

$C_{94}H_{152}O_{47}$

(3β,4α,16α)-28-[[*O*-β-D-Glucopyranosyl-(1→3)-*O*-[β-D-xylopyranosyl-(1→4)]-*O*-6-deoxy-α-L-mannopyranosyl-(1→2)-3-*O*-[(3*S*,5*S*,6*S*)-5-[[(3*S*,5*S*,6*S*)-5-(α-L-arabinofuranosyloxy)-3-hydroxy-6-methyl-1-oxooctyl]oxy]-3-hydroxy-6-methyl-1-oxooctyl]-6-deoxy-β-D-galactopyranosyl]oxy]-16-hydroxy-23,28-dioxoolean-12-en-...

3 References | 0 Reactions | 0 Suppliers

16

250643-61-9

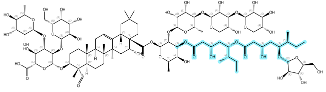

Absolute stereochemistry shown

$C_{93}H_{150}O_{46}$

(3β,4α,16α)-16-Hydroxy-23,28-dioxo-28-[[*O*-β-D-xylopyranosyl-(1→3)-*O*-β-D-xylopyranosyl-(1→4)-*O*-6-deoxy-α-L-mannopyranosyl-(1→2)-3-*O*-[(3*S*,5*S*,6*S*)-5-[[(3*S*,5*S*,6*S*)-5-(α-L-arabinofuranosyloxy)-3-hydroxy-6-methyl-1-oxooctyl]oxy]-3-hydroxy-6-methyl-1-oxooctyl]-6-deoxy-β-D-galactopyranosyl]oxy]olean-12-en-3-y...

3 References | 0 Reactions | 0 Suppliers

17

250643-60-8

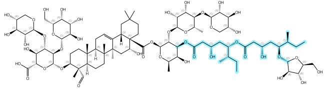

Absolute stereochemistry shown

$C_{87}H_{140}O_{42}$

(3β,4α,16α)-16-Hydroxy-23,28-dioxo-28-[[*O*-β-D-xylopyranosyl-(1→4)-*O*-6-deoxy-α-L-mannopyranosyl-(1→2)-3-*O*-[(3*S*,5*S*,6*S*)-5-[[(3*S*,5*S*,6*S*)-5-(α-L-arabinofuranosyloxy)-3-hydroxy-6-methyl-1-oxooctyl]oxy]-3-hydroxy-6-methyl-1-oxooctyl]-6-deoxy-β-D-galactopyranosyl]oxy]olean-12-en-3-yl *O*-β-D-galactopyranosyl-(...

3 References | 0 Reactions | 0 Suppliers

18

2345644-24-6

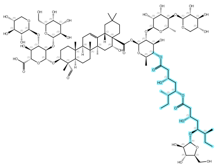

Absolute stereochemistry shown

$C_{87}H_{140}O_{42}$

β-D-Glucopyranosiduronic acid, (3β,4α,16α)-16-hydroxy-23,28-dioxo-28-[[*O*-β-D-xylopyranosyl-(1→4)-*O*-6-deoxy-α-L-mannopyranosyl-(1→2)-4-*O*-[(3*S*,5*S*,6*S*)-5-[[(3*S*,5*S*,6*S*)-5-(α-L-arabinofuranosyloxy)-3-hydroxy-6-methyl-1-oxooctyl]oxy]-3-hydroxy-6-methyl-1-oxooctyl]-6-deoxy-β-D-glucopyranosyl]oxy]olean-12-en-...

2 References | 0 Reactions | 0 Suppliers

19

2345644-22-4

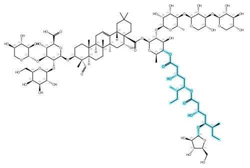

Absolute stereochemistry shown

$C_{92}H_{148}O_{46}$

β-D-Glucopyranosiduronic acid, (3β,4α, 16α)-16-hydroxy-23,28-dioxo-28-[[*O*-β-D-xylopyranosyl-(1→3)-*O*-β-D-xylopyranosyl-(1→4)-*O*-6-deoxy-α-L-mannopyranosyl-(1→2)-4-*O*-[(3*S*,5*S*,6*R*)-5-[[(3*S*,5*S*,6*R*)-5-(α-L-arabinofuranosyloxy)-3-hydroxy-6-methyl-1-oxooctyl]oxy]-3-hydroxy-6-methyl-1-oxooctyl]-6-deoxy-β-D-gluco...

2 References | 0 Reactions | 0 Suppliers

20

836632-91-8

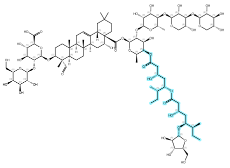

Absolute stereochemistry shown

$C_{87}H_{140}O_{42}$

(3β,4α,16α)-16-Hydroxy-23,28-dioxo-28-[[*O*-β-D-xylopyranosyl-(1→3)-*O*-β-D-xylopyranosyl-(1→4)-*O*-6-deoxy-α-L-mannopyranosyl-(1→2)-4-*O*-[(3*S*,5*S*,6*S*)-5-[[(3*S*,5*S*,6*S*)-5-(α-L-arabinofuranosyloxy)-3-hydroxy-6-methyl-1-oxooctyl]oxy]-3-hydroxy-6-methyl-1-oxooctyl]-6-deoxy-β-D-galactopyranosyl]oxy]olean-12-en-3-y...

2 References | 0 Reactions | 0 Suppliers

21

836632-65-6

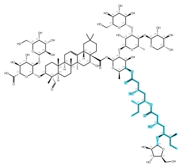

Absolute stereochemistry shown

$C_{88}H_{142}O_{43}$

(3β,4α,16α)-28-[[*O*-β-D-Glucopyranosyl-(1→3)-*O*-[β-D-xylopyranosyl-(1→4)]-*O*-6-deoxy-α-L-mannopyranosyl-(1→2)-4-*O*-[(3*S*,5*S*,6*S*)-5-[[(3*S*,5*S*,6*S*)-5-(α-L-arabinofuranosyloxy)-3-hydroxy-6-methyl-1-oxooctyl]oxy]-3-hydroxy-6-methyl-1-oxooctyl]-6-deoxy-β-D-galactopyranosyl]oxy]-16-hydroxy-23,28-dioxoolean-12-en-...

2 References | 0 Reactions | 0 Suppliers

22

263259-80-9

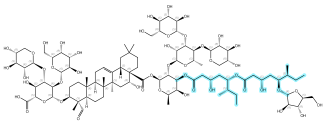

Absolute stereochemistry shown

$C_{93}H_{150}O_{47}$

(3β,4α,16α)-28-[[*O*-β-D-Glucopyranosyl-(1→3)-*O*-[β-D-xylopyranosyl-(1→4)]-*O*-6-deoxy-α-L-mannopyranosyl-(1→2)-3-*O*-[(3*S*,5*S*,6*S*)-5-[[(3*S*,5*S*,6*S*)-5-(α-L-arabinofuranosyloxy)-3-hydroxy-6-methyl-1-oxooctyl]oxy]-3-hydroxy-6-methyl-1-oxooctyl]-6-deoxy-β-D-galactopyranosyl]oxy]-16-hydroxy-23,28-dioxoolean-12-en-...

2 References | 0 Reactions | 0 Suppliers

23

2756968-82-6

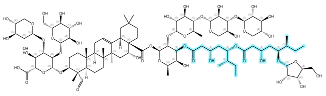

Absolute stereochemistry shown

$C_{92}H_{148}O_{46}$

β-D-Glucopyranosiduronic acid, (3β,4α, 16α)-16-hydroxy-23,28-dioxo-28-[[*O*-β-D-xylopyranosyl-(1→3)-*O*-β-L-xylopyranosyl-(1→4)-*O*-6-deoxy-α-D-glucopyranosyl-(1→2)-6-deoxy-3-*O*-[(3*S*,5*S*,6*S*)-3-hydroxy-5-[[(3*S*,5*S*,6*S*)-3-hydroxy-6-methyl-1-oxo-5-(α-L-ribofuranosyloxy)heptyl]oxy]-6-methyl-1-oxoheptyl]-β-D-galact...

1 Reference | 0 Reactions | 0 Suppliers

24

2756968-80-4

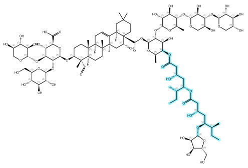

Absolute stereochemistry shown

$C_{92}H_{148}O_{46}$

β-D-Glucopyranosiduronic acid, (3β,4α, 16α)-16-hydroxy-23,28-dioxo-28-[[*O*-β-D-xylopyranosyl-(1→3)-*O*-β-L-xylopyranosyl-(1→4)-*O*-6-deoxy-α-D-glucopyranosyl-(1→2)-6-deoxy-4-*O*-[(3*S*,5*S*,6*S*)-3-hydroxy-5-[[(3*S*,5*S*,6*S*)-3-hydroxy-6-methyl-1-oxo-5-(α-L-ribofuranosyloxy)heptyl]oxy]-6-methyl-1-oxoheptyl]-β-D-galact...

1 Reference | 0 Reactions | 0 Suppliers

25

2587448-50-6

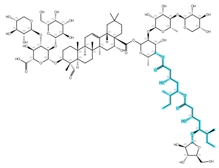

Absolute stereochemistry shown

$C_{87}H_{140}O_{42}$

β-D-Glucopyranosiduronic acid, (3β,4α, 16α)-16-hydroxy-23,28-dioxo-28-[[*O*-β-D-xylopyranosyl-(1→4)-*O*-6-deoxy-α-L-mannopyranosyl-(1→2)-4-*O*-[(3*S*,5*S*,6*S*)-5-[[(3*S*,5*S*,6*S*)-5-(α-L-arabinofuranosyloxy)-3-hydroxy-6-methyl-1-oxooctyl]oxy]-3-hydroxy-6-methyl-1-oxooctyl]-6-deoxy-L-galactopyranosyl]oxy]olean-12-en-...

1 Reference | 0 Reactions | 0 Suppliers

26

1114960-22-3

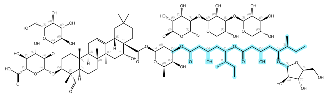

Absolute stereochemistry shown

$C_{87}H_{140}O_{42}$

(3β,4α,16α)-16-Hydroxy-23,28-dioxo-28-[[*O*-β-D-xylopyranosyl-(1→3)-*O*-β-D-xylopyranosyl-(1→4)-*O*-6-deoxy-α-L-mannopyranosyl-(1→2)-3-*O*-[(3*S*,5*S*,6*S*)-5-[[(3*S*,5*S*,6*S*)-5-(α-L-arabinofuranosyloxy)-3-hydroxy-6-methyl-1-oxooctyl]oxy]-3-hydroxy-6-methyl-1-oxooctyl]-6-deoxy-β-D-galactopyranosyl]oxy]olean-12-en-3-y...

1 Reference | 0 Reactions | 0 Suppliers

27

1114960-20-1

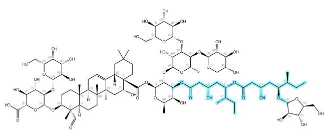

Absolute stereochemistry shown

$C_{88}H_{142}O_{43}$

(3β,4α,16α)-28-[[*O*-β-D-Glucopyranosyl-(1→3)-*O*-[β-D-xylopyranosyl-(1→4)]-*O*-6-deoxy-α-L-mannopyranosyl-(1→2)-3-*O*-[(3*S*,5*S*,6*S*)-5-[[(3*S*,5*S*,6*S*)-5-(α-L-arabinofuranosyloxy)-3-hydroxy-6-methyl-1-oxooctyl]oxy]-3-hydroxy-6-methyl-1-oxooctyl]-6-deoxy-β-D-galactopyranosyl]oxy]-16-hydroxy-23,28-dioxoolean-12-en-...

1 Reference | 0 Reactions | 0 Suppliers

28

1114960-19-8

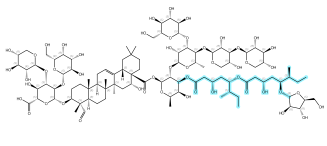

Absolute stereochemistry shown

$C_{98}H_{158}O_{51}$

(3β,4α,16α)-16-Hydroxy-23,28-dioxo-28-[[*O*-β-D-xylopyranosyl-(1→3)-*O*-β-D-xylopyranosyl-(1→4)-*O*-[β-D-glucopyranosyl-(1→3)]-*O*-6-deoxy-α-L-mannopyranosyl-(1→2)-3-*O*-[(3*S*,5*S*,6*S*)-5-[[(3*S*,5*S*,6*S*)-5-(α-L-arabinofuranosyloxy)-3-hydroxy-6-methyl-1-oxooctyl]oxy]-3-hydroxy-6-methyl-1-oxooctyl]-6-deoxy-β-D-galacto...

1 Reference | 0 Reactions | 0 Suppliers

29

1114960-18-7

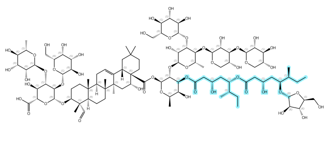

Absolute stereochemistry shown

$C_{99}H_{160}O_{51}$

(3β,4α,16α)-16-Hydroxy-23,28-dioxo-28-[[*O*-β-D-xylopyranosyl-(1→3)-*O*-β-D-xylopyranosyl-(1→4)-*O*-[β-D-glucopyranosyl-(1→3)]-*O*-6-deoxy-α-L-mannopyranosyl-(1→2)-3-*O*-[(3*S*,5*S*,6*S*)-5-[[(3*S*,5*S*,6*S*)-5-(α-L-arabinofuranosyloxy)-3-hydroxy-6-methyl-1-oxooctyl]oxy]-3-hydroxy-6-methyl-1-oxooctyl]-6-deoxy-β-D-galacto...

1 Reference | 0 Reactions | 0 Suppliers

30

837374-95-5

325966-83-4

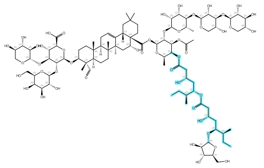

Absolute stereochemistry shown

6014-42-2

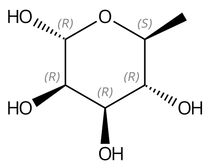

Absolute stereochemistry shown

$C_{100}H_{160}O_{51}$

β-D-Glucopyranosiduronic acid, (3β,4α, 16α)-16-hydroxy-23,28-dioxo-28-[[*O*-β-D-xylopyranosyl-(1→3)-*O*-β-D-xylopyranosyl-(1→4)-*O*-6-deoxy-α-L-mannopyranosyl-(1→2)-3-*O*-acetyl-4-*O*-[(3*S*,5*S*,6*S*)-5-[[(3*S*,5*S*, 6*S*)-5-(α-L-arabinofuranosyloxy)-3-hydroxy-6-methyl-1-oxooctyl]oxy]-3-hydroxy-6-methyl-1-oxooctyl]-6-deox...

1 Reference | 0 Reactions | 0 Suppliers

31

837374-93-3

325966-82-3

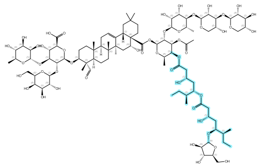

Absolute stereochemistry shown

6014-42-2

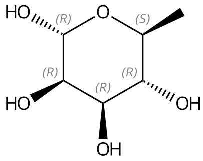

Absolute stereochemistry shown

$C_{101}H_{162}O_{51}$

β-D-Glucopyranosiduronic acid, (3β,4α, 16α)-16-hydroxy-23,28-dioxo-28-[[*O*-β-D-xylopyranosyl-(1→3)-*O*-β-D-xylopyranosyl-(1→4)-*O*-6-deoxy-α-L-mannopyranosyl-(1→2)-3-*O*-acetyl-4-*O*-[(3*S*,5*S*,6*S*)-5-[[(3*S*,5*S*,6*S*)-5-(α-L-arabinofuranosyloxy)-3-hydroxy-6-methyl-1-oxooctyl]oxy]-3-hydroxy-6-methyl-1-oxooctyl]-6-deox...

1 Reference | 0 Reactions | 0 Suppliers

32

836632-94-1

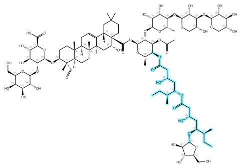

Absolute stereochemistry shown

$C_{89}H_{142}O_{43}$

β-D-Glucopyranosiduronic acid, (3β,4α, 16α)-16-hydroxy-23,28-dioxo-28-[[*O*-β-D-xylopyranosyl-(1→3)-*O*-β-D-xylopyranosyl-(1→4)-*O*-6-deoxy-α-L-mannopyranosyl-(1→2)-3-*O*-acetyl-4-*O*-[(3*S*,5*S*,6*S*)-5-[[(3*S*,5*S*,6*S*)-5-(α-L-arabinofuranosyloxy)-3-hydroxy-6-methyl-1-oxooctyl]oxy]-3-hydroxy-6-methyl-1-oxooctyl]-6-deox...

1 Reference | 0 Reactions | 0 Suppliers

33

836632-88-3

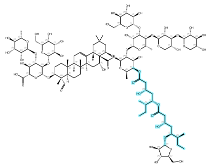

Absolute stereochemistry shown

$C_{99}H_{160}O_{52}$

β-D-Glucopyranosiduronic acid, (3β,4α, 16α,22β)-16,22-dihydroxy-23,28-dioxo-28-[[*O*-β-D-xylopyranosyl-(1→3)-*O*-β-D-xylopyranosyl-(1→4)-*O*-[β-D-glucopyranosyl-(1→3)]-*O*-6-deoxy-α-L-mannopyranosyl-(1→2)-4-*O*-[(3*S*,5*S*,6*S*)-5-[[(3*S*,5*S*,6*S*)-5-(α-L-arabinofuranosyloxy)-3-hydroxy-6-methyl-1-oxooctyl]oxy]-3-hydroxy-...

1 Reference | 0 Reactions | 0 Suppliers

34

836632-86-1

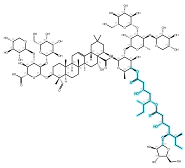

Absolute stereochemistry shown

$C_{93}H_{150}O_{48}$

β-D-Glucopyranosiduronic acid, (3β,4α, 16α,22β)-28-[[*O*-β-D-glucopyranosyl-(1→3)-*O*-[β-D-xylopyranosyl-(1→4)]-*O*-6-deoxy-α-L-mannopyranosyl-(1→2)-4-*O*-[(3*S*,5*S*,6*S*)-5-[[(3*S*,5*S*,6*S*)-5-(α-L-arabinofuranosyloxy)-3-hydroxy-6-methyl-1-oxooctyl]oxy]-3-hydroxy-6-methyl-1-oxooctyl]-6-deoxy-β-D-galactopyranosyl]oxy]...

1 Reference | 0 Reactions | 0 Suppliers

35

836632-84-9

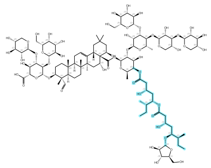

Absolute stereochemistry shown

$C_{98}H_{158}O_{52}$

β-D-Glucopyranosiduronic acid, (3β,4α, 16α,22β)-16,22-dihydroxy-23,28-dioxo-28-[[*O*-β-D-xylopyranosyl-(1→3)-*O*-β-D-xylopyranosyl-(1→4)-*O*-[β-D-glucopyranosyl-(1→3)]-*O*-6-deoxy-α-L-mannopyranosyl-(1→2)-4-*O*-[(3*S*,5*S*,6*S*)-5-[[(3*S*,5*S*,6*S*)-5-(α-L-arabinofuranosyloxy)-3-hydroxy-6-methyl-1-oxooctyl]oxy]-3-hydroxy-...

1 Reference | 0 Reactions | 0 Suppliers

36

2446587-94-4

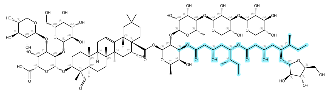

Absolute stereochemistry shown

$C_{92}H_{148}O_{46}$

β-D-Glucopyranosiduronic acid, (3β,4α, 16α)-16-hydroxy-23,28-dioxo-28-[[*O*-β-D-xylopyranosyl-(1→3)-*O*-β-D-xylopyranosyl-(1→4)-*O*-6-deoxy-α-L-mannopyranosyl-(1→2)-3-*O*-[(3*S*,5*S*,6*S*)-5-[[(3*S*,5*S*,6*S*)-5-(α-L-arabinofuranosyloxy)-3-hydroxy-6-methyl-1-oxooctyl]oxy]-3-hydroxy-6-methyl-1-oxooctyl]-6-deoxy-β-D-galac...

0 References | 0 Reactions | 0 Suppliers

37

2446826-41-9

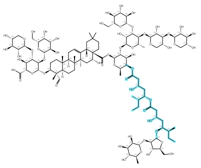

Absolute stereochemistry shown

$C_{104}H_{168}O_{55}$

β-D-Glucopyranosiduronic acid, (3β,4α,16α)-16-hydroxy-23,28-dioxo-28-[*O*-β-D-xylopyranosyl-(1→3)-*O*-β-D-xylopyranosyl-(1→4)-*O*-[β-D-glucopyranosyl-(1→3)]-*O*-6-deoxy-α-L-mannopyranosyl-(1→2)-4-*O*-[(3*S*,5*S*,6*S*)-5-[[(3*S*,5*S*,6*S*)-5-[[2-*O*-(6-deoxy-α-L-mannopyranosyl)-α-L-arabinofuranosyl]oxy]-3-hydroxy-6-methyl-1...

13 References | 1 Reaction | 0 Suppliers

38

2377518-27-7

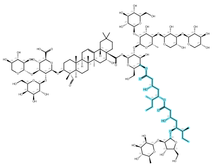

Absolute stereochemistry shown

$C_{104}H_{168}O_{56}$

β-D-Glucopyranosiduronic acid, (3β,4α,6α)-16-hydroxy-23,28-dioxo-28-[[*O*-β-D-xylopyranosyl-(1→3)-*O*-β-D-xylopyranosyl-(1→4)-*O*-[β-D-glcopyranosyl-(1→2)]-*O*-6-deoxy-α-L-mannopyranosyl-(1→2)-4-*O*-[(3*S*,5*S*,6*S*)-5-[[(3*S*,5*S*,6*S*)-5-[[2-*O*-(6-deoxy-α-L-mannopyranosyl)-α-L-arabinofuranosyl]oxy]-3-hydroxy-6-methyl-1-...

1 Reference | 0 Reactions | 0 Suppliers

39

2446587-95-5

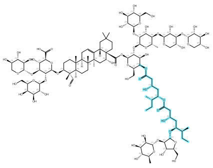

Absolute stereochemistry shown

$C_{104}H_{168}O_{56}$

β-D-Glucopyranosiduronic acid, (3β,4α,6α)-16-hydroxy-23,28-dioxo-28-[[*O*-α-D-xylopyranosyl-(1→3)-*O*-β-D-xylopyranosyl-(1→4)-*O*-[β-D-glcopyranosyl-(1→2)]-*O*-6-deoxy-α-L-mannopyranosyl-(1→2)-4-*O*-[(3*S*,5*S*,6*S*)-5-[[(3*S*,5*S*,6*S*)-5-[[2-*O*-(6-deoxy-α-L-mannopyranosyl)-α-L-arabinofuranosyl]oxy]-3-hydroxy-6-methyl-1-...

0 References | 0 Reactions | 0 Suppliers

40

141256-04-4

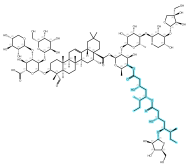

Absolute stereochemistry shown

$C_{92}H_{148}O_{46}$

Stimulon

1,172 References | 301 Reactions | 11 Suppliers

41

836632-59-8

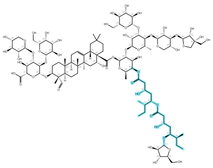

Absolute stereochemistry shown

$C_{98}H_{158}O_{51}$

QS 18

64 References | 1 Reaction | 0 Suppliers

42

250643-64-2

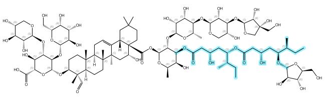

Absolute stereochemistry shown

$C_{92}H_{148}O_{46}$

(3β,4α,16α)-28-[[*O*-D-Apio-β-D-furanosyl-(1→3)-*O*-β-D-xylopyranosyl-(1→4)-*O*-6-deoxy-α-L-mannopyranosyl-(1→2)-3-*O*-[(3*S*,5*S*,6*S*)-5-[[(3*S*,5*S*,6*S*)-5-(α-L-arabinofuranosyloxy)-3-hydroxy-6-methyl-1-oxooctyl]oxy]-3-hydroxy-6-methyl-1-oxooctyl]-6-deoxy-β-D-galactopyranosyl]oxy]-16-hydroxy-23,28-dioxoolean-12-en-...

14 References | 1 Reaction | 0 Suppliers

43

2448141-14-6

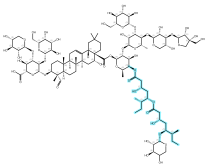

Absolute stereochemistry shown

$C_{98}H_{158}O_{51}$

β-D-Glucopyranosiduronic acid, (3β,4α, 16α)-28-[[*O*-D-apio-β-D-furanosyl-(1→3)-β-D-xylopyranosyl-(1→4)-*O*-[β-D-glucopyranosyl-(1→3)]-*O*-6-deoxy-α-L-mannopyranosyl-(1→2)-6-deoxy-4-*O*-[(3*S*,5*S*,6*S*)-3-hydroxy-5-[[(3*S*,5*S*,6*S*)-3-hydroxy-6-methyl-1-oxo-5-[(4ξ)-β-D-*threo*-pentopyranosyloxy]octyl]oxy]-5-methyl-1-oxo...

12 References | 1 Reaction | 0 Suppliers

44

154335-25-8

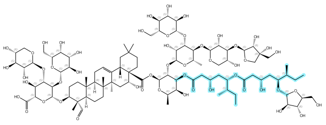

Absolute stereochemistry shown

$C_{98}H_{158}O_{51}$

(3β,4α,16α)-28-[[*O*-D-Apio-β-D-furanosyl-(1→3)-*O*-β-D-xylopyranosyl-(1→4)-*O*-[β-D-glucopyranosyl-(1→3)]-*O*-6-deoxy-α-L-mannopyranosyl-(1→2)-3-*O*-[(3*S*,5*S*,6*S*)-5-[[(3*S*,5*S*,6*S*)-5-(α-L-arabinofuranosyloxy)-3-hydroxy-6-methyl-1-oxooctyl]oxy]-3-hydroxy-6-methyl-1-oxooctyl]-6-deoxy-β-D-galactopyranosyl]oxy]-16-hy...

10 References | 0 Reactions | 0 Suppliers

45

250643-57-3

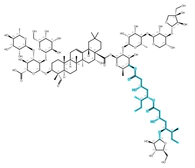

Absolute stereochemistry shown

$C_{93}H_{150}O_{46}$

(3β,4α,16α)-28-[[*O*-D-Apio-β-D-furanosyl-(1→3)-*O*-β-D-xylopyranosyl-(1→4)-*O*-6-deoxy-α-L-mannopyranosyl-(1→2)-4-*O*-[(3*S*,5*S*,6*S*)-5-[[(3*S*,5*S*,6*S*)-5-(α-L-arabinofuranosyloxy)-3-hydroxy-6-methyl-1-oxooctyl]oxy]-3-hydroxy-6-methyl-1-oxooctyl]-6-deoxy-β-D-galactopyranosyl]oxy]-16-hydroxy-23,28-dioxoolean-12-en-...

6 References | 0 Reactions | 0 Suppliers

46

836632-63-4

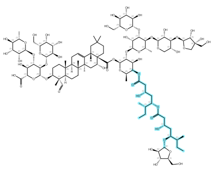

Absolute stereochemistry shown

$C_{99}H_{160}O_{51}$

(3β,4α,16α)-28-[[*O*-D-Apio-β-D-furanosyl-(1→3)-*O*-β-D-xylopyranosyl-(1→4)-*O*-[β-D-glucopyranosyl-(1→3)]-*O*-6-deoxy-α-L-mannopyranosyl-(1→2)-4-*O*-[(3*S*,5*S*,6*S*)-5-[[(3*S*,5*S*,6*S*)-5-(α-L-arabinofuranosyloxy)-3-hydroxy-6-methyl-1-oxooctyl]oxy]-3-hydroxy-6-methyl-1-oxooctyl]-6-deoxy-β-D-galactopyranosyl]oxy]-16-hy...

5 References | 0 Reactions | 0 Suppliers

47

325966-85-6

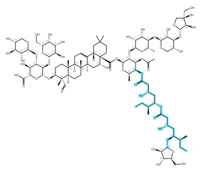

Absolute stereochemistry shown

$C_{94}H_{150}O_{47}$

(3β,4α,16α)-28-[[*O*-D-Apio-β-D-furanosyl-(1→3)-*O*-β-D-xylopyranosyl-(1→4)-*O*-6-deoxy-α-L-mannopyranosyl-(1→2)-3-*O*-acetyl-4-*O*-[(3*S*,5*S*,6*S*)-5-[[(3*S*,5*S*,6*S*)-5-(α-L-arabinofuranosyloxy)-3-hydroxy-6-methyl-1-oxooctyl]oxy]-3-hydroxy-6-methyl-1-oxooctyl]-6-deoxy-β-D-galactopyranosyl]oxy]-16-hydroxy-23,28-dioxoo...

5 References | 0 Reactions | 0 Suppliers

48

325966-84-5

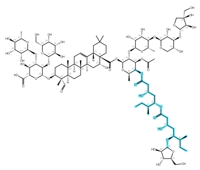

Absolute stereochemistry shown

$C_{95}H_{152}O_{47}$

(3β,4α,16α)-28-[[*O*-D-Apio-β-D-furanosyl-(1→3)-*O*-β-D-xylopyranosyl-(1→4)-*O*-6-deoxy-α-L-mannopyranosyl-(1→2)-3-*O*-acetyl-4-*O*-[(3*S*,5*S*,6*S*)-5-[[(3*S*,5*S*,6*S*)-5-(α-L-arabinofuranosyloxy)-3-hydroxy-6-methyl-1-oxooctyl]oxy]-3-hydroxy-6-methyl-1-oxooctyl]-6-deoxy-β-D-galactopyranosyl]oxy]-16-hydroxy-23,28-dioxoo...

4 References | 0 Reactions | 0 Suppliers

49

2711015-46-0

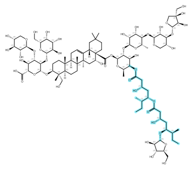

Absolute stereochemistry shown

$C_{92}H_{150}O_{46}$

β-D-Glucopyranosiduronic acid, (3β,4α,16α)-28-[(*O*-D-apio-β-D-furanosyl-(1→3)-*O*-β-D-xylopyranosyl-(1→4)-*O*-6-deoxy-α-L-mannopyranosyl-(1→2)-4-*O*-[(3*S*,5*S*,6*S*)-5-[[(3*S*,5*S*,6*S*)-5-(α-L-arabinofuranosyloxy)-3-hydroxy-6-methyl-1-oxooctyl]oxy]-3-hydroxy-6-methyl-1-oxooctyl]-6-deoxy-β-D-galactopyranosyl)oxy]-16,...

3 References | 5 Reactions | 0 Suppliers

50

250643-63-1

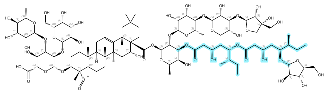

Absolute stereochemistry shown

$C_{93}H_{150}O_{46}$

(3β,4α,16α)-28-[[*O*-D-Apio-β-D-furanosyl-(1→3)-*O*-β-D-xylopyranosyl-(1→4)-*O*-6-deoxy-α-L-mannopyranosyl-(1→2)-3-*O*-[(3*S*,5*S*,6*S*)-5-[[(3*S*,5*S*,6*S*)-5-(α-L-arabinofuranosyloxy)-3-hydroxy-6-methyl-1-oxooctyl]oxy]-3-hydroxy-6-methyl-1-oxooctyl]-6-deoxy-β-D-galactopyranosyl]oxy]-16-hydroxy-23,28-dioxoolean-12-en-...

3 References | 0 Reactions | 0 Suppliers

51

2345644-31-5

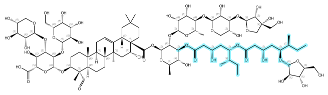

Absolute stereochemistry shown

$C_{92}H_{148}O_{46}$

β-D-Glucopyranosiduronic acid, (3β,4α,16α)-28-[[*O*-D-apio-β-D-furanosyl-(1→3)-*O*-β-D-xylopyranosyl-(1→4)-*O*-6-deoxy-α-L-mannopyranosyl-(1→2)-3-*O*-[(3*S*,5*S*,6*R*)-5-[[(3*S*,5*S*,6*R*)-5-(α-L-arabinofuranosyloxy)-3-hydroxy-6-methyl-1-oxooctyl]oxy]-3-hydroxy-6-methyl-1-oxooctyl]-6-deoxy-β-D-glucopyranosyl]oxy]-16-hy...

2 References | 0 Reactions | 0 Suppliers

52

2345644-26-8

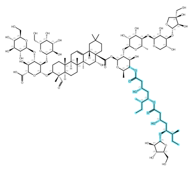

Absolute stereochemistry shown

$C_{93}H_{150}O_{47}$

β-D-Glucopyranosiduronic acid, (3β,4α,16α)-28-[[*O*-D-apio-β-D-furanosyl-(1→3)-*O*-β-D-xylopyranosyl-(1→4)-*O*-6-deoxy-α-L-mannopyranosyl-(1→2)-4-*O*-[(3*S*,5*S*,6*R*)-5-[[(3*S*,5*S*,6*R*)-5-(α-L-arabinofuranosyloxy)-3-hydroxy-6-methyl-1-oxooctyl]oxy]-3-hydroxy-6-methyl-1-oxooctyl]-6-deoxy-β-D-glucopyranosyl]oxy]-16-hy...

2 References | 0 Reactions | 0 Suppliers

53

836632-66-7

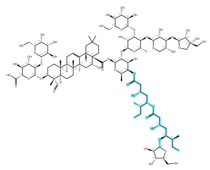

Absolute stereochemistry shown

$C_{93}H_{150}O_{47}$

(3β,4α,16α)-28-[[*O*-D-Apio-β-D-furanosyl-(1→3)-*O*-β-D-xylopyranosyl-(1→4)-*O*-[β-D-glucopyranosyl-(1→3)]-*O*-6-deoxy-α-L-mannopyranosyl-(1→2)-4-*O*-[(3*S*,5*S*,6*S*)-5-[[(3*S*,5*S*,6*S*)-5-(α-L-arabinofuranosyloxy)-3-hydroxy-6-methyl-1-oxooctyl]oxy]-3-hydroxy-6-methyl-1-oxooctyl]-6-deoxy-β-D-galactopyranosyl]oxy]-16-hy...

2 References | 0 Reactions | 0 Suppliers

54

263259-81-0

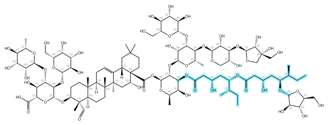

Absolute stereochemistry shown

$C_{99}H_{160}O_{51}$

(3β,4α,16α)-28-[[*O*-D-Apio-β-D-furanosyl-(1→3)-*O*-β-D-xylopyranosyl-(1→4)-*O*-[β-D-glucopyranosyl-(1→3)]-*O*-6-deoxy-α-L-mannopyranosyl-(1→2)-3-*O*-[(3*S*,5*S*,6*S*)-5-[[(3*S*,5*S*,6*S*)-5-(α-L-arabinofuranosyloxy)-3-hydroxy-6-methyl-1-oxooctyl]oxy]-3-hydroxy-6-methyl-1-oxooctyl]-6-deoxy-β-D-galactopyranosyl]oxy]-16-hy...

2 References | 0 Reactions | 0 Suppliers

55

2756968-81-5

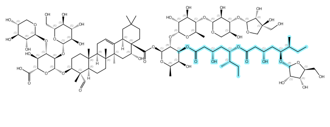

Absolute stereochemistry shown

$C_{92}H_{148}O_{46}$

β-D-Glucopyranosiduronic acid, (3β,4α, 16α)-28-[[*O*-6-*O*-D-apio-β-L-furanosyl-(1→3)-*O*-α-L-xylopyranosyl-(1→4)-*O*-6-deoxy-α-D-glucopyranosyl-(1→2)-6-deoxy-3-*O*-[(3*S*,5*S*,6*S*)-3-hydroxy-5-[[(3*S*,5*S*,6*S*)-3-hydroxy-6-methyl-1-oxo-5-(α-L-ribofuranosyloxy)heptyl]oxy]-6-methyl-1-oxoheptyl]-β-D-galactopyranosyl]oxy]-...

1 Reference | 0 Reactions | 0 Suppliers

56

2344757-97-5

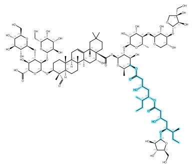

Absolute stereochemistry shown

$C_{93}H_{150}O_{47}$

β-D-Glucopyranosiduronic acid, (3β,4α, 16α)-28-[*O*-D-apio-β-D-furanosyl-(1→3)-*O*-β-D-xylopyranosyl-(1→4)-*O*-6-deoxy-α-L-mannopyranosyl-(1→2)-4-*O*-[(3*S*,5*S*,6*S*)-5-[[(3*S*,5*S*,6*S*)-5-(α-L-arabinofuranosyloxy)-3-hydroxy-6-methyl-1-oxooctyl]-3-hydroxy-6-methyl-1-oxooctyl]-6-deoxy-β-D-galactopyranosyl]oxy]-16-hydro...

1 Reference | 0 Reactions | 0 Suppliers

57

1114960-21-2

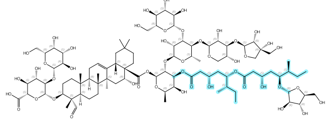

Absolute stereochemistry shown

$C_{93}H_{150}O_{47}$

(3β,4α,16α)-28-[[*O*-D-Apio-β-D-furanosyl-(1→3)-*O*-β-D-xylopyranosyl-(1→4)-*O*-[β-D-glucopyranosyl-(1→3)]-*O*-6-deoxy-α-L-mannopyranosyl-(1→2)-3-*O*-[(3*S*,5*S*,6*S*)-5-[[(3*S*,5*S*,6*S*)-5-(α-L-arabinofuranosyloxy)-3-hydroxy-6-methyl-1-oxooctyl]oxy]-3-hydroxy-6-methyl-1-oxooctyl]-6-deoxy-β-D-galactopyranosyl]oxy]-16-hy...

1 Reference | 0 Reactions | 0 Suppliers

58

837374-97-7

836632-59-8

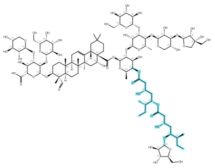

Absolute stereochemistry shown

6014-42-2

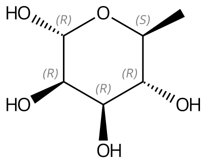

Absolute stereochemistry shown

$C_{104}H_{168}O_{55}$

β-D-Glucopyranosiduronic acid, (3β,4α, 16α)-28-[[*O*-D-apio-β-D-furanosyl-(1→3)-*O*-β-D-xylopyranosyl-(1→4)-*O*-[β-D-glucopyranosyl-(1→3)]-*O*-6-deoxy-α-L-mannopyranosyl-(1→2)-4-*O*-[(3*S*,5*S*,6*S*)-5-[[(3*S*,5*S*,6*S*)-5-(α-L-arabinofuranosyloxy)-3-hydroxy-6-methyl-1-oxooctyl]oxy]-3-hydroxy-6-methyl-1-oxooctyl]-6-deoxy-...

1 Reference | 0 Reactions | 0 Suppliers

59

837374-96-6

325966-85-6

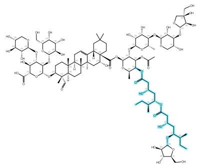

Absolute stereochemistry shown

6014-42-2

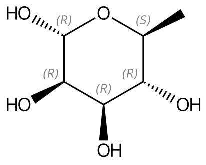

Absolute stereochemistry shown

$C_{100}H_{160}O_{51}$

β-D-Glucopyranosiduronic acid, (3β,4α, 16α)-28-[[*O*-D-apio-β-D-furanosyl-(1→3)-*O*-β-D-xylopyranosyl-(1→4)-*O*-6-deoxy-α-L-mannopyranosyl-(1→2)-3-*O*-acetyl-4-*O*-[(3*S*,5*S*,6*S*)-5-[[(3*S*,5*S*,6*S*)-5-(α-L-arabinofuranosyloxy)-3-hydroxy-6-methyl-1-oxooctyl]oxy]-3-hydroxy-6-methyl-1-oxooctyl]-6-deoxy-β-D-galactopyranos...

1 Reference | 0 Reactions | 0 Suppliers

60

837374-94-4

325966-84-5

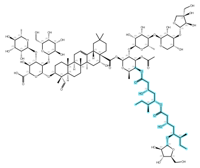

Absolute stereochemistry shown

6014-42-2

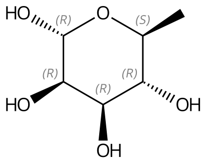

Absolute stereochemistry shown

$C_{101}H_{162}O_{51}$

β-D-Glucopyranosiduronic acid, (3β,4α, 16α)-28-[[*O*-D-apio-β-D-furanosyl-(1→3)-*O*-β-D-xylopyranosyl-(1→4)-*O*-6-deoxy-α-L-mannopyranosyl-(1→2)-3-*O*-acetyl-4-*O*-[(3*S*,5*S*,6*S*)-5-[[(3*S*,5*S*,6*S*)-5-(α-L-arabinofuranosyloxy)-3-hydroxy-6-methyl-1-oxooctyl]oxy]-3-hydroxy-6-methyl-1-oxooctyl]-6-deoxy-β-D-galactopyranos...

1 Reference | 0 Reactions | 0 Suppliers

Copyright © 2022 American Chemical Society (ACS). All Rights Reserved.
Internal use only. Redistribution is subject to the terms of your SciFinder^{n} License Agreement and CAS information Use Policies.

Supplementary data 5

Confirmation of the structure of des-arabinosyl QS-21 (D -apiose) (11) (used in UGT73CZ2 assays)

No.	δ_C, Type	δ_H mult, (*J* in Hz)	No.	δ_C, Type	δ_H mult, (*J* in Hz)
**1**	39.4, CH_2	1.70/1.12, m	**Gal-2**	73.3, CH	3.48, m
**2**	25.9, CH_2	1.92/1.78, m	**Gal-3**	75.4, CH	3.47, m
**3**	86.3, CH	3.91, m	**Gal-4**	70.9, CH	3.83, m
**4**	56.6, Cq	-	**Gal-5**	76.9, CH	3.51, m
**5**	49.1, CH	1.33, m	**Gal-6**	62.4, CH_2	3.80/3.71, m
**6**			**C_3-Xyl-1**	105.0, CH	4.60, d (7.2)
**7**	33.7, CH_2, HMBC	Not observed in HSQC	**C_3-Xyl-2**	75.4, CH	3.23, m
**8**	41.2, Cq	-	**C_3-Xyl-3**	78.2, CH	3.33, overlapped
**9**	48.1, CH	1.74, m	**C_3-Xyl-4**	71.0, CH	3.53, m
**10**	37.2, Cq	-	**C_3-Xyl-5**	67.0, CH_2	3.92/3.24, m
**11**	24.6, CH_2	1.92/1.92, m	**Fuc-1**	95.0, CH	5.35, m overlapped
**12**	123.4, CH	5.34, m	**Fuc-2**	75.2, CH	3.77, m
**13**	144.7, Cq	-	**Fuc-3**	74.6, CH	3.88, m
**14**	43.0, Cq	-	**Fuc-4**	75.4, CH	5.11, d (4.2), m
**15**	36.8, CH_2	1.60/1.45, m	**Fuc-5**	71.0, CH	3.85, m
**16**	74.6, CH	4.48, br d (7.7)	**Fuc-6**	16.7, CH_3	1.09, d (6.4)
**17**	50.3, Cq	-	**C_{28}-Rha-1**	101.6, CH	5.36, m overlapped
**18**	42.2, CH	2.94, dd (15.3, 4.1)	**C_{28}-Rha-2**	72.0, CH	3.93, m
**19**	48.1, CH_2	2.30/1.05, m	**C_{28}-Rha-3**	72.1, CH	3.80, m
**20**	31.4, Cq	-	**C_{28}-Rha-4**	85.0, CH	3.53, m
**21**	36.7, CH_2	1.94/1.18, m	**C_{28}-Rha-5**	69.0, CH	3.77, m
**22**	Not observed in HSQC		**C_{28}-Rha-6**	18.5, CH_3	1.33, d (6.1)
**23**	211.6, CH	9.46, s	**C_{28}-Xyl-1**	107.4, CH	4.48, br d (7.7)
**24**	11.1, CH_3	1.18, s	**C_{28}-Xyl-2**	75.6, CH	3.31, m
**25**	16.6, CH_3	1.01, s	**C_{28}-Xyl-3**	85.7, CH	3.43, m
**26**	17.9, CH_3	0.76, s	**C_{28}-Xyl-4**	69.7, CH	3.51, m
**27**	27.3, CH_3	1.39, s	**C_{28}-Xyl-5**	66.8, CH_2	(3.89/3.24), m
**28**	177.4, Cq	-	**Api-1**	111.0, CH	5.26, d (2.9)
**29**	33.6, CH_3	0.88, s	**Api-2**	78.0, CH	4.07, d (3)
**30**	25.0, CH_3	0.94, s	**Api--3**	80.8, Cq	-
**GlcA-1**	104.6, CH	4.48, br d (7.7)	**Api-4**	75.1, CH_2	4.17, d (9.7)/3.83, m
**GlcA-2**	78.5, CH	3.67, m	**Api-5**	65.4, CH_2	3.68, m
**GlcA-3**	Not observed in HSQC				
**GlcA-4**	71.5, CH	3.55, m			
**GlcA-5**	76.6, CH	3.80, m			
**GlcA-6**	Not observed in HSQC				
**Gal-1**	103.8, CH	4.82, d (7.1)			

Continuo

No.	δ_C, Type	δ_H mult, (*J* in Hz)
**Fa-1**	173.4, Cq	-
**Fa-2**	43.6, CH_2	2.64, dd (8.6, 6.5)
**Fa-3**	66.3, CH	4.02, m
**Fa-4**	39.7, CH_2	1.68/1.84, m
**Fa-5**	75.6, CH	5.19, m
**Fa-6**	40.3, CH	1.61, m
**Fa-7**	26.6, CH_2	1.51/1.15, m
**Fa-8**	12.4, CH_3	0.92, m
**Fa-9**	14.9, CH_3	0.94, br d (1.7)
**Fa-10**	174.0, Cq	-
**Fa-11**	44.2, CH_2	2.53, dd (14.2, 6.5)
**Fa-12**	66.9, CH	4.26, ddd (13.0, 8.4, 4.8)
**Fa-13**	Not observed in HSQC	
**Fa-14**	72.1, CH	3.76, m
**Fa-15**	42.3, CH	1.35, m
**Fa-16**	26.9, CH_2	1.56/1.11, m
**Fa-17**	12.4, CH_3	0.94, m
**Fa-18**	14.4, CH_3	0.88, overlapped

Table A: ^{1}H, ^{13}C NMR spectral data for **11** in MeOH-d4/D2O (9/1), (400, 100 MHz)

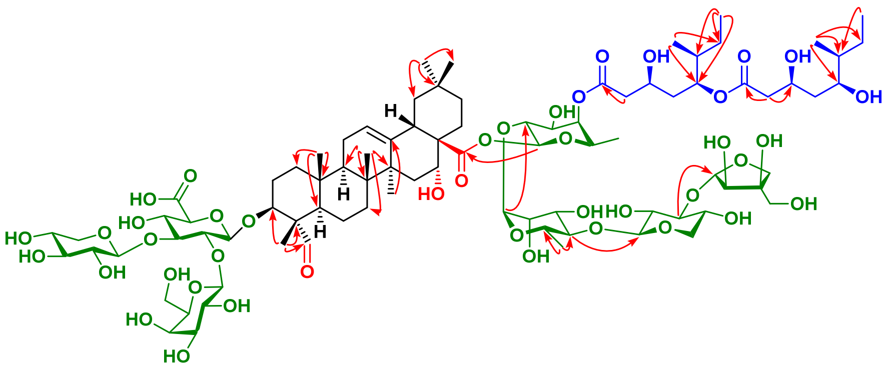

Scheme 1: Key HMBC (H→C)

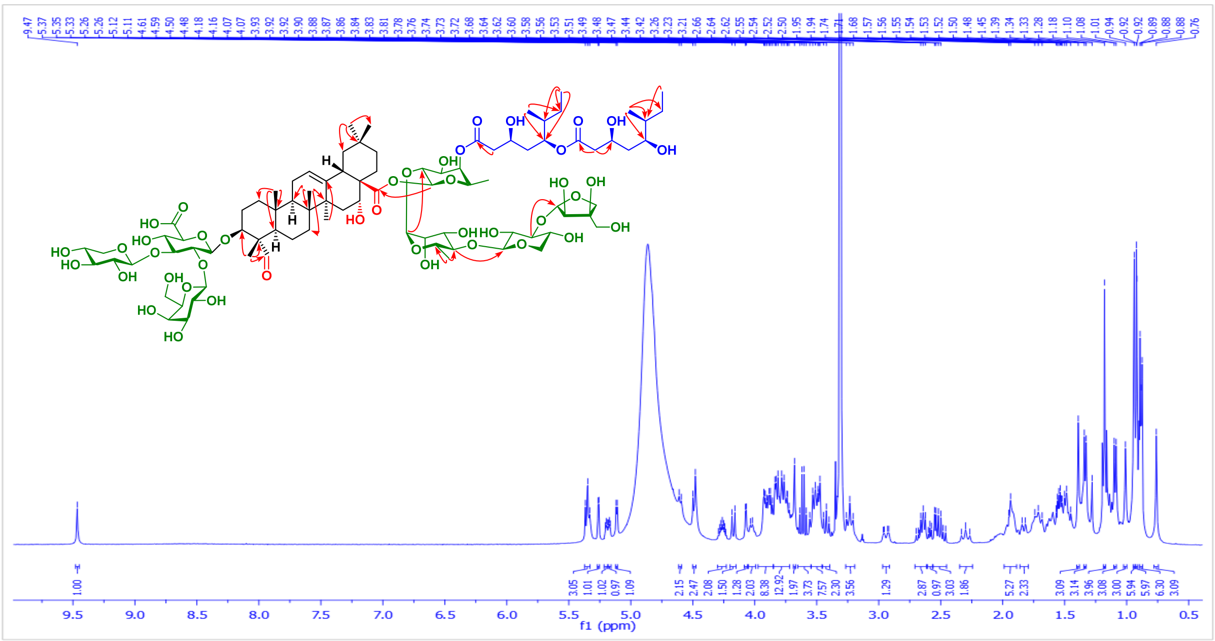

Figure A: ^{1}HNMR in MeOH-d_4/D_2O, 9/1 (400 MHz)

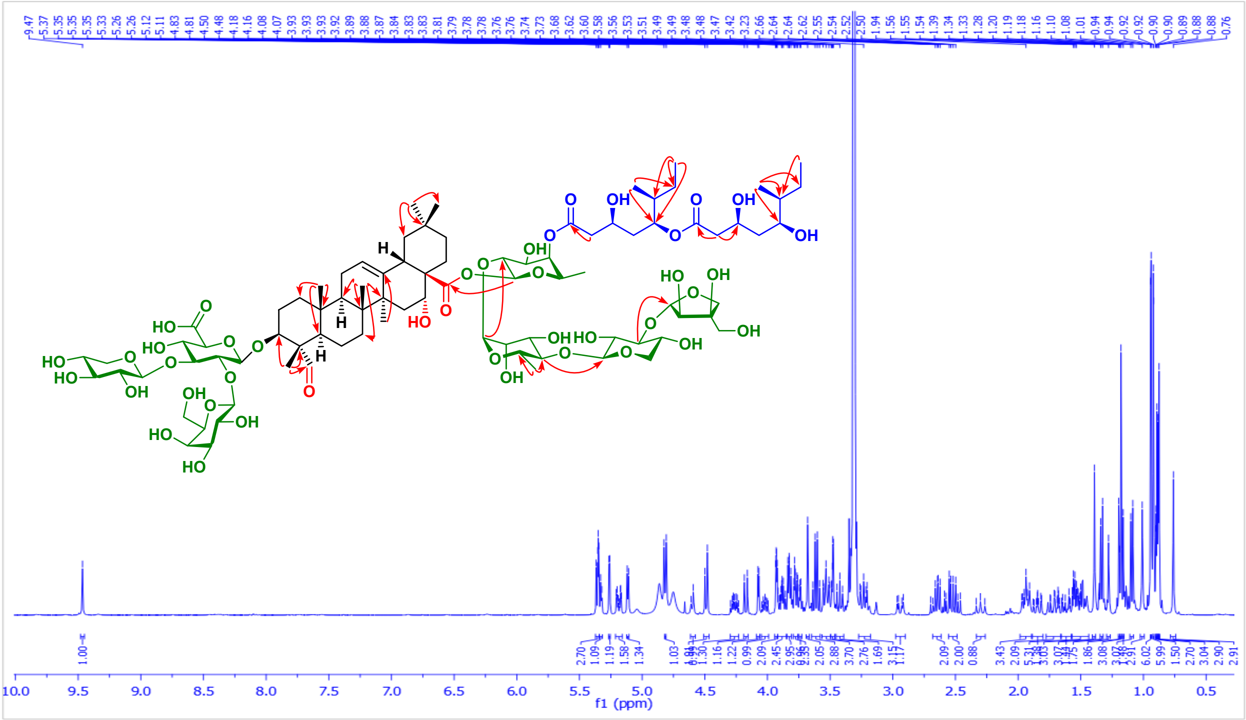

Figure B: ^{1}HNMR (water residue removed by GSD, mesternova) in MeOH-d_4/D_2O, 9/1 (400 MHz)

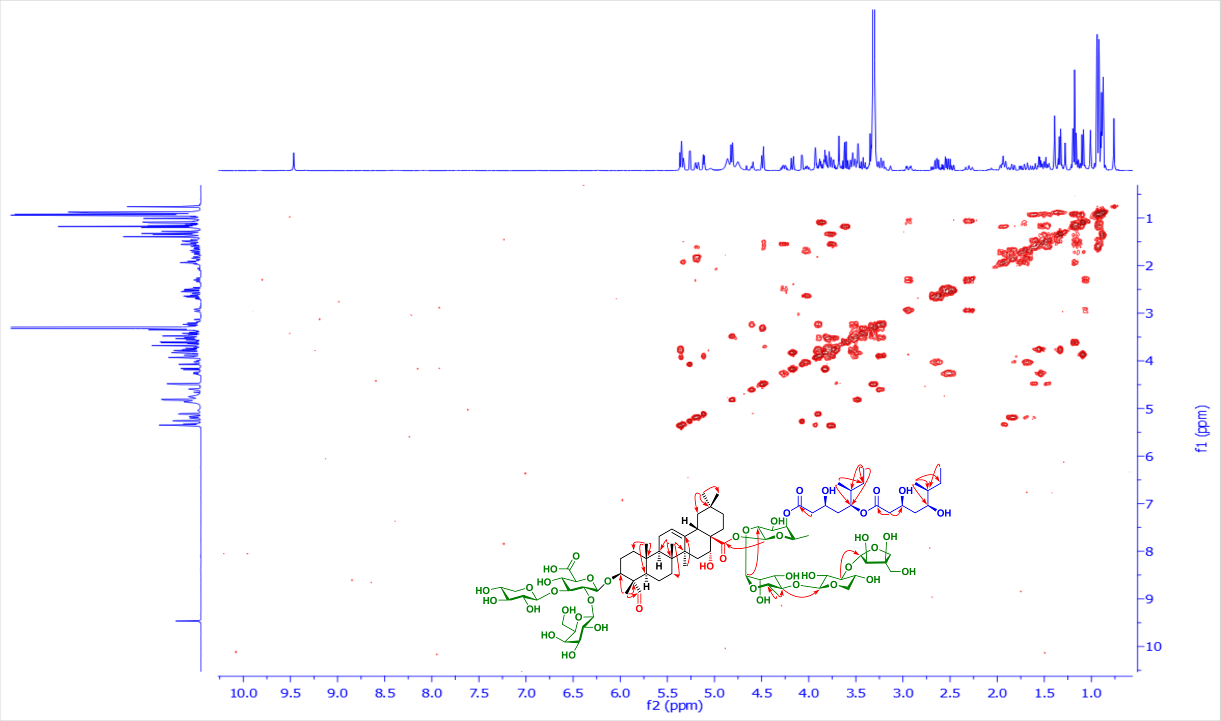

Figure C: ^{1}H-^{1}H COSY in MeOH-d_4/D_2O, 9/1 (400 MHz)

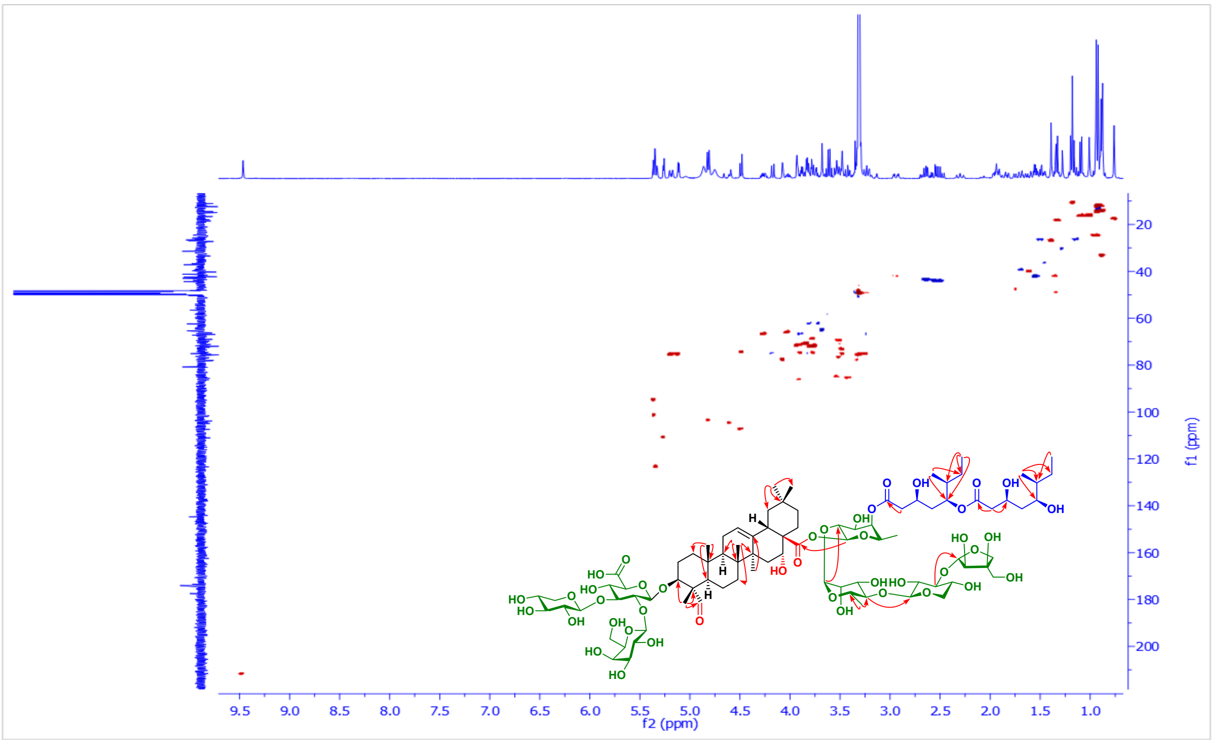

Figure D: ^{1}H-^{13}C HSQC in MeOH-d_4/D_2O, 9/1 (400, 100 MHz)

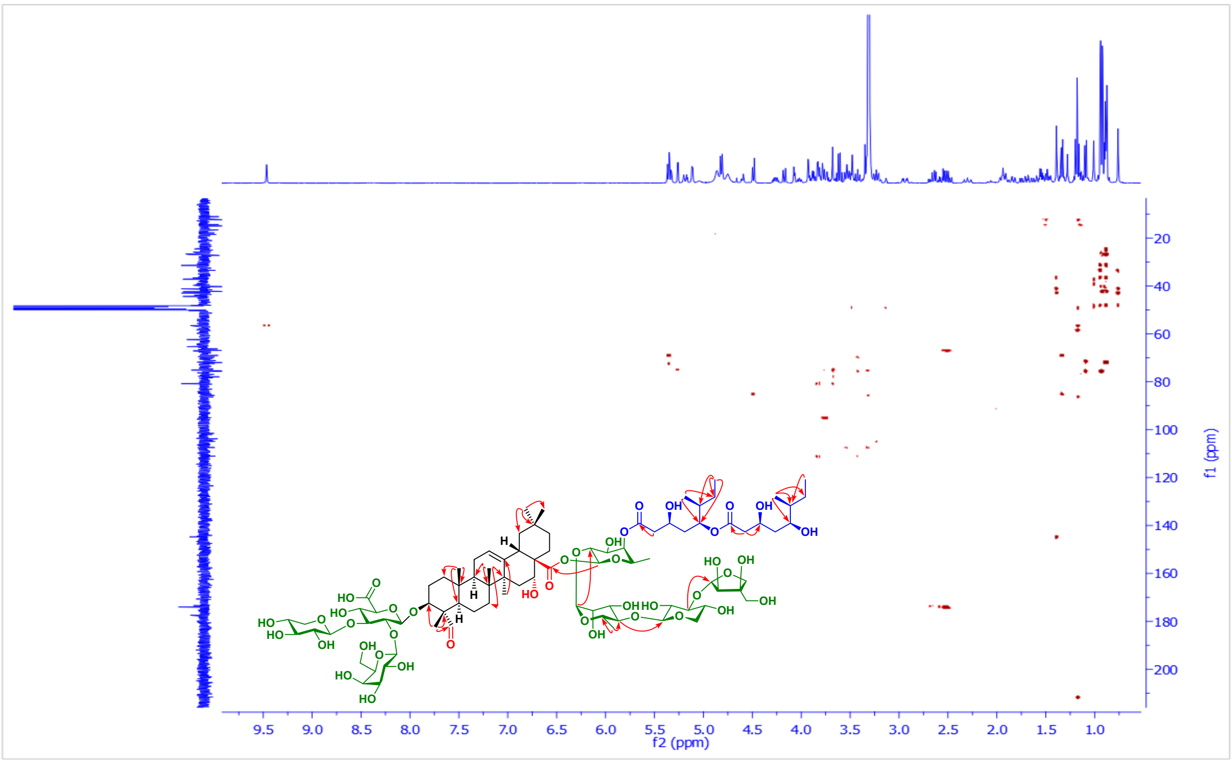

Figure E: ^{1}H-^{13}C HMBC in MeOH-d_4/D_2O, 9/1 (400, 100 MHz)

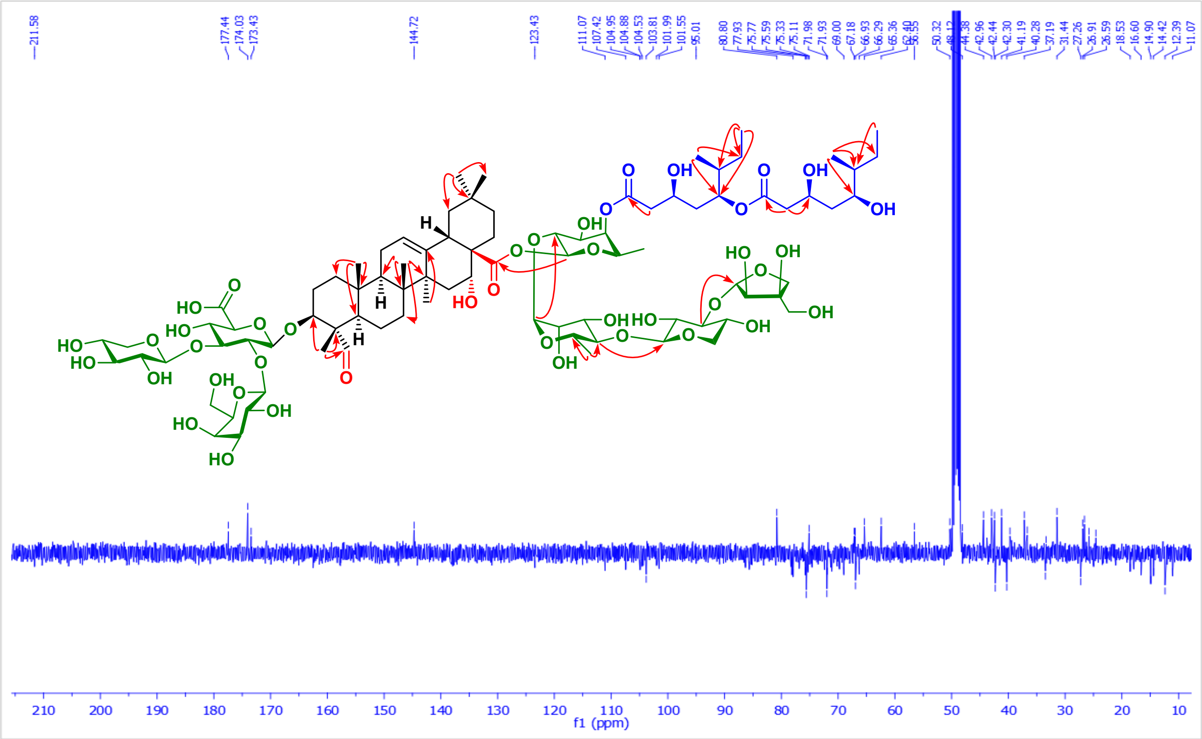

Figure F: DEPTQ in MeOH-d_4/D_2O, 9/1 (100 MHz)

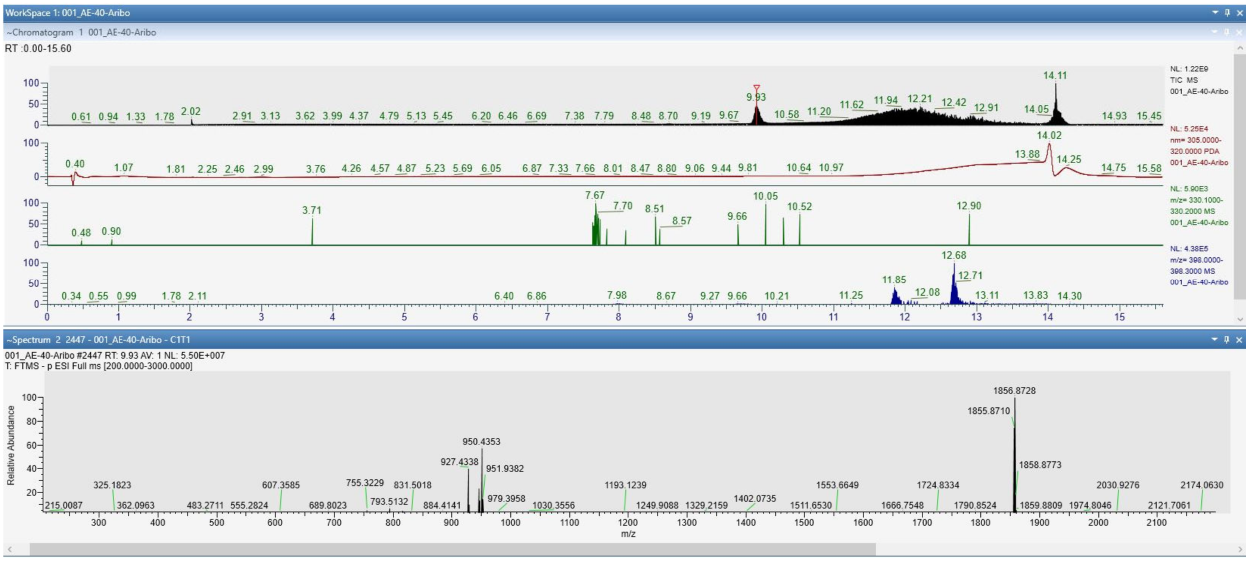

Figure G: LC-HRESI-MS

Supplementary Data 6

Spectral Confirmation of QS-21/Des-apiosyl QS-21 produced and purified from *Nicotiana benthamiana*

QS-21

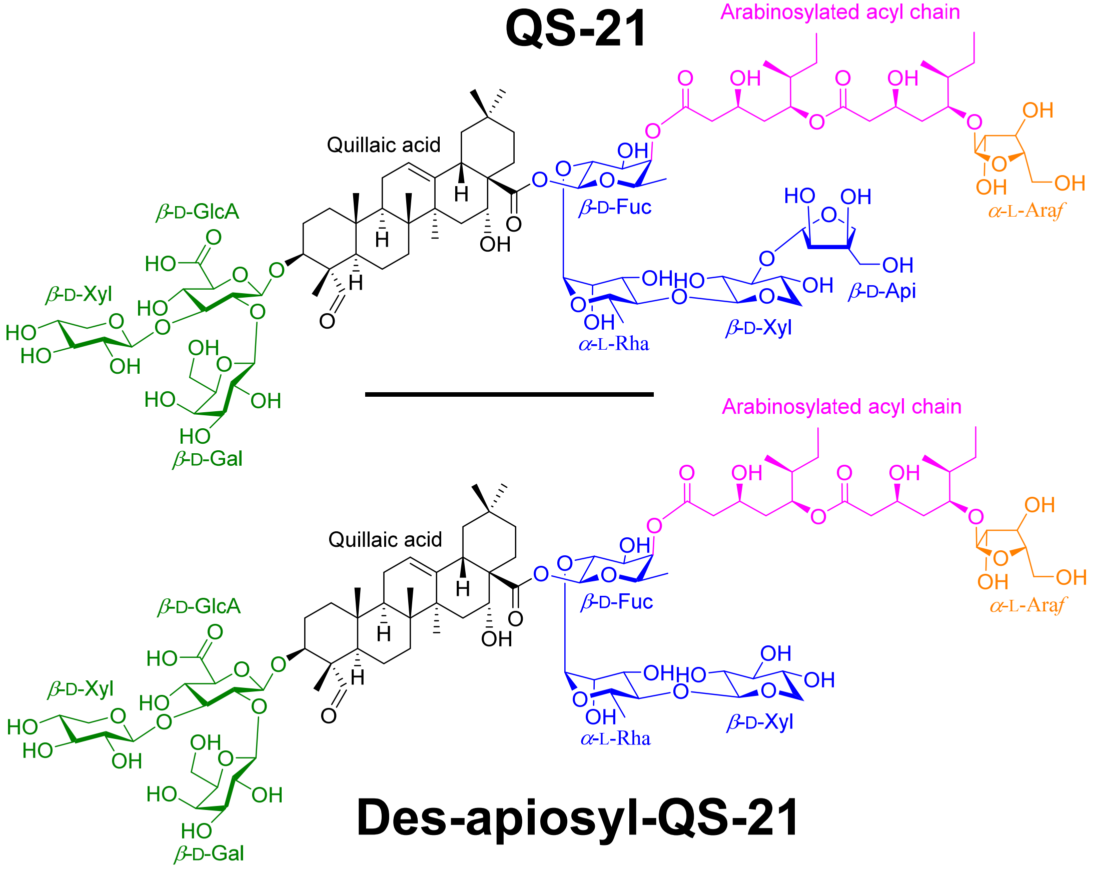

Des-apiosyl-QS-21

Table of Contents

Scheme 1: Key ^{1}H-NMR resonances recorded (compared with standard) for semi-purified QS-21 produced in *benthamiana*.. 3

Scheme 2: Key HMBC (H→C, red arrows) reported for semi-purified QS-21 produced in *benthamiana* 4

Figure S1: ^{1}H-NMR spectrum of semi-purified QS-21 (**1**) produced in *benthamiana*, recorded in MeOH-d_4, 600 MHz 5

Figure S2: Expanded ^{1}H-NMR spectrum (4.80-9.50 ppm) of semi-purified QS-21 (**1**) produced in *benthamiana*, recorded in MeOH-d_4, 600 MHz 6

Figure S3: Expanded ^{1}H-NMR spectrum (3.00-4.80 ppm) of semi-purified QS-21 (**1**) produced in *benthamiana*, recorded in MeOH-d_4, 600 MHz 7

Figure S4: Expanded ^{1}H-NMR spectrum (3.00-4.80 ppm) of semi-purified QS-21 (**1**) produced in *benthamiana*, recorded in MeOH-d_4, 600 MHz 8

Figure S5: Expanded ^{1}H-NMR spectrum (0.5-3.00 ppm) of semi-purified QS-21 (**1**) produced in *benthamiana*, recorded in MeOH-d_4, 600 MHz 9

Figure S6: Comparison between ^{1}H-NMR spectrum of **semi-purified QS-21 produced in *benthamiana***, and **QS-21 standard**, both recorded in MeOH-d_4, 600 MHz 10

Figure S7: Expanded ^{1}HNMR spectral comparison between **QS-21 standard** and **semi-QS-21 produced in *benth*** (**4.00-4.95 ppm**), recored in MeOH-d_4, 600 MHz 11

Figure S8: Expanded ^{1}HNMR spectral comparison between **QS-21 standard** and **semi-QS-21 produced in *benth*** (4.96-5.38 ppm), recored in MeOH-d_4, 600 MHz. 12

Figure S9: ^{1}H-^{1}H COSY spectrum of semi-purified QS-21 (**1**) produced in *benthamiana*, recorded in MeOH-d_4, 600 MHz... 13

Figure S10: ^{1}H-^{13}C HSQC spectrum of semi-purified QS-21 (**1**) produced in *benthamiana*, recorded in MeOH-d_4, 600/150 MHz... 14

Figure S11: Expanded ^{1}H-^{13}C HSQC spectrum of the anomeric region (4.20-5.50/60-130 ppm) of semi-purified QS-21 (**1**) produced in *benthamiana*, recorded in MeOH-d_4, 600/150 MHz 15

Figure S12: ^{1}H-^{13}C HMBC spectrum of semi-purified QS-21 (**1**) produced in *benthamiana*, recorded in MeOH-d_4, 600/150 MHz 16

Figure S13: Expanded key ^{1}H-^{13}C HMBC spectrum (4.96-5.00/76-81.5 ppm) of semi-purified QS-21 (**1**) produced in *benthamiana*, recorded in MeOH-d_4, 600/150 MHz 17

Figure S14: LC-HR-ESI(-ve)-MS of non-separable mixture (1:1) of QS-21/Des-apiosyl-QS-21 18

Scheme 1: Key ^{1}H-NMR resonances recorded (compared with standard) for semi-purified QS-21 produced in *benthamiana*

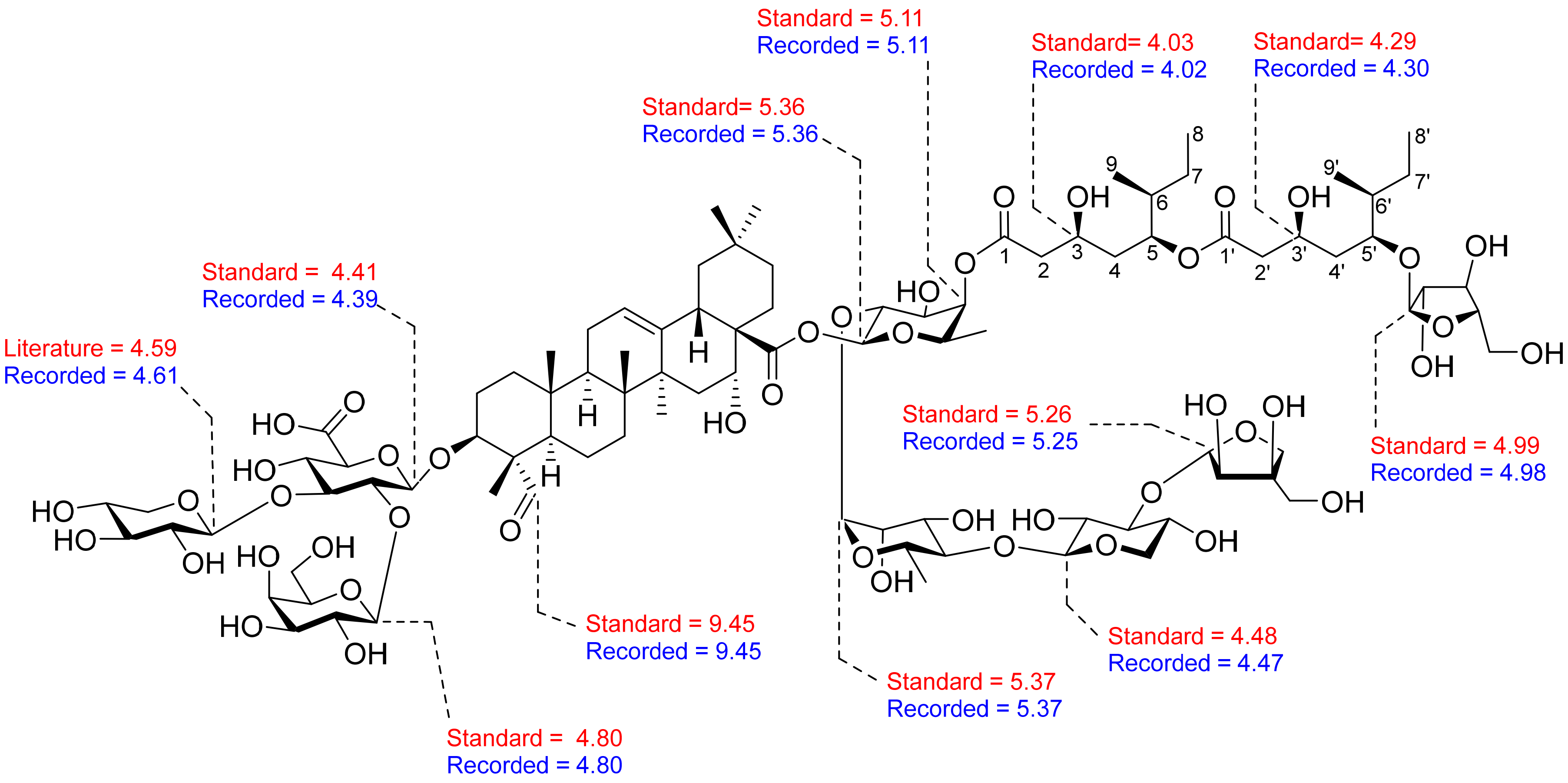

Scheme 2: Key HMBC (H→C, red arrows) reported for semi-purified QS-21 produced in *benthamiana*

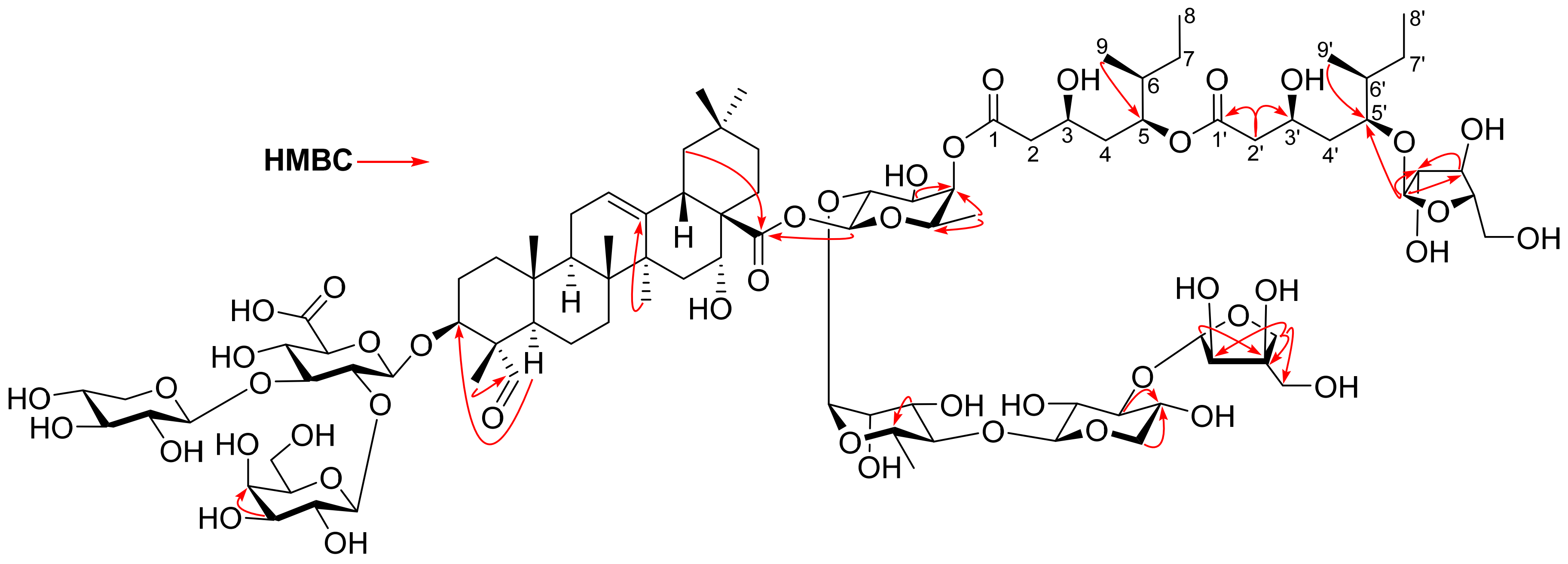

Figure S1: ^{1}H-NMR spectrum of semi-purified QS-21 (**1**) produced in *benthamiana*, recorded in MeOH-d_4, 600 MHz

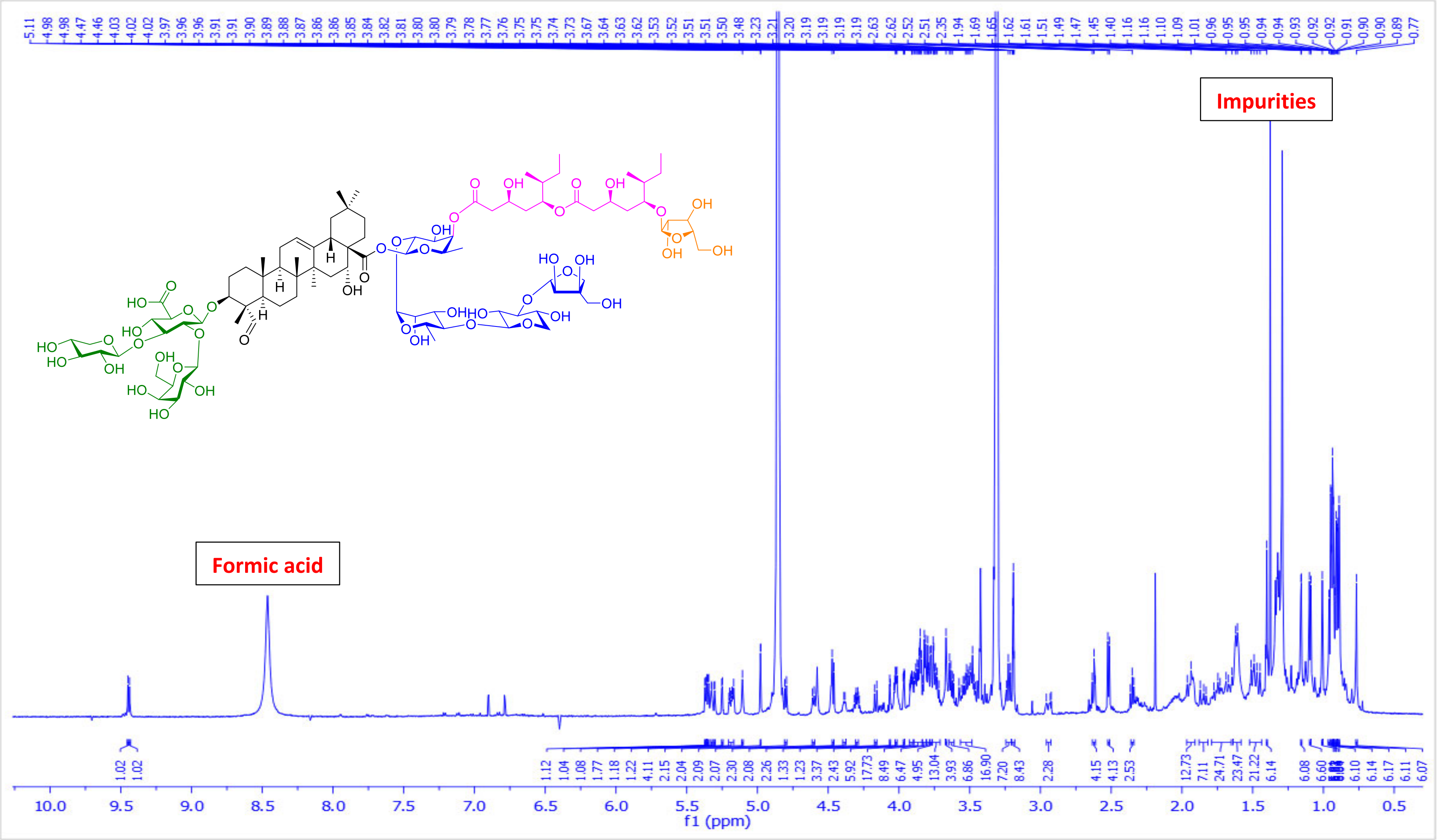

Figure S2: Expanded ^{1}H-NMR spectrum (4.80-9.50 ppm) of semi-purified QS-21 (**1**) produced in *benthamiana*, recorded in MeOH-d_4, 600 MHz

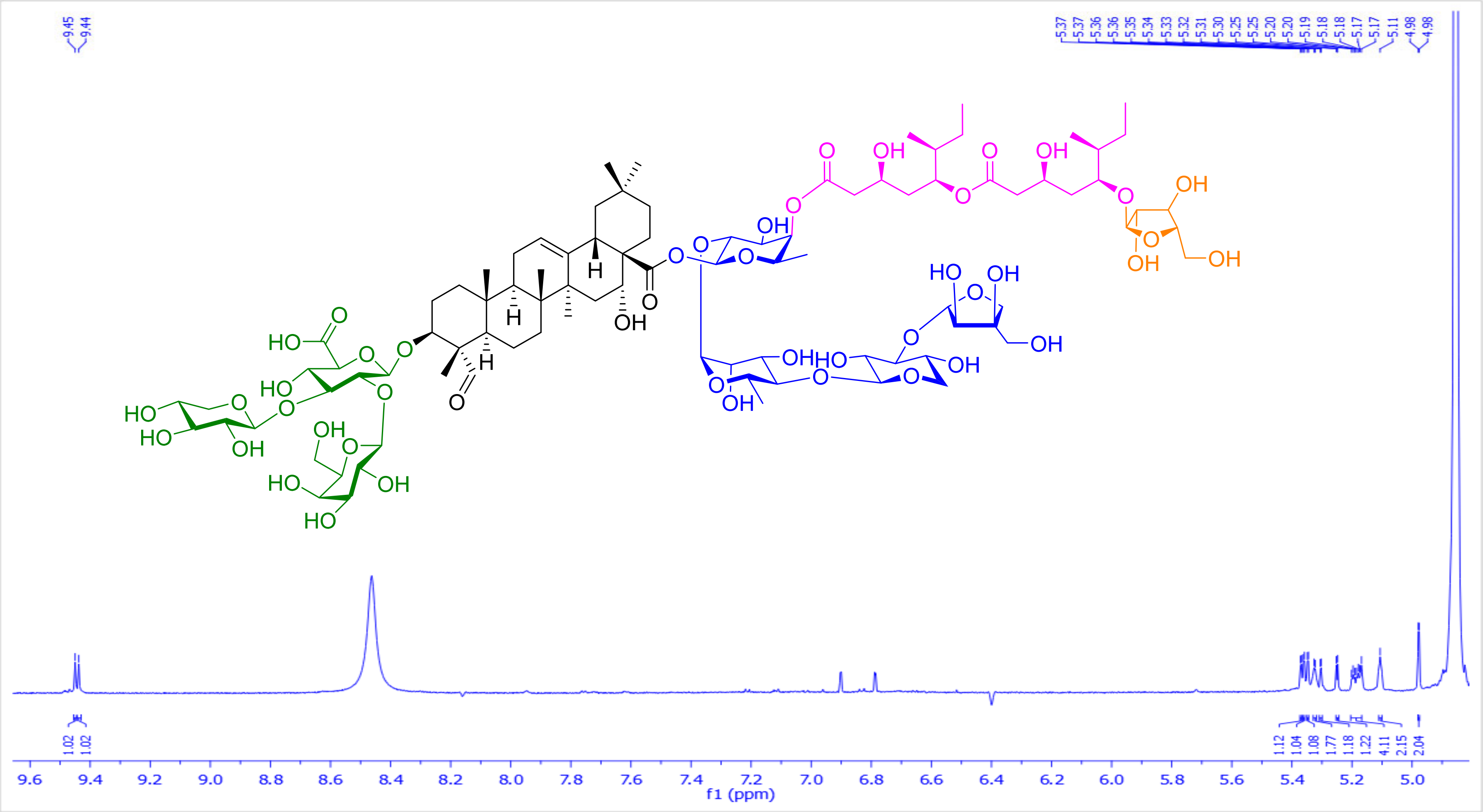

Figure S3: Expanded ^{1}H-NMR spectrum (3.00-4.80 ppm) of semi-purified QS-21 (**1**) produced in *benthamiana*, recorded in MeOH-d_4, 600 MHz

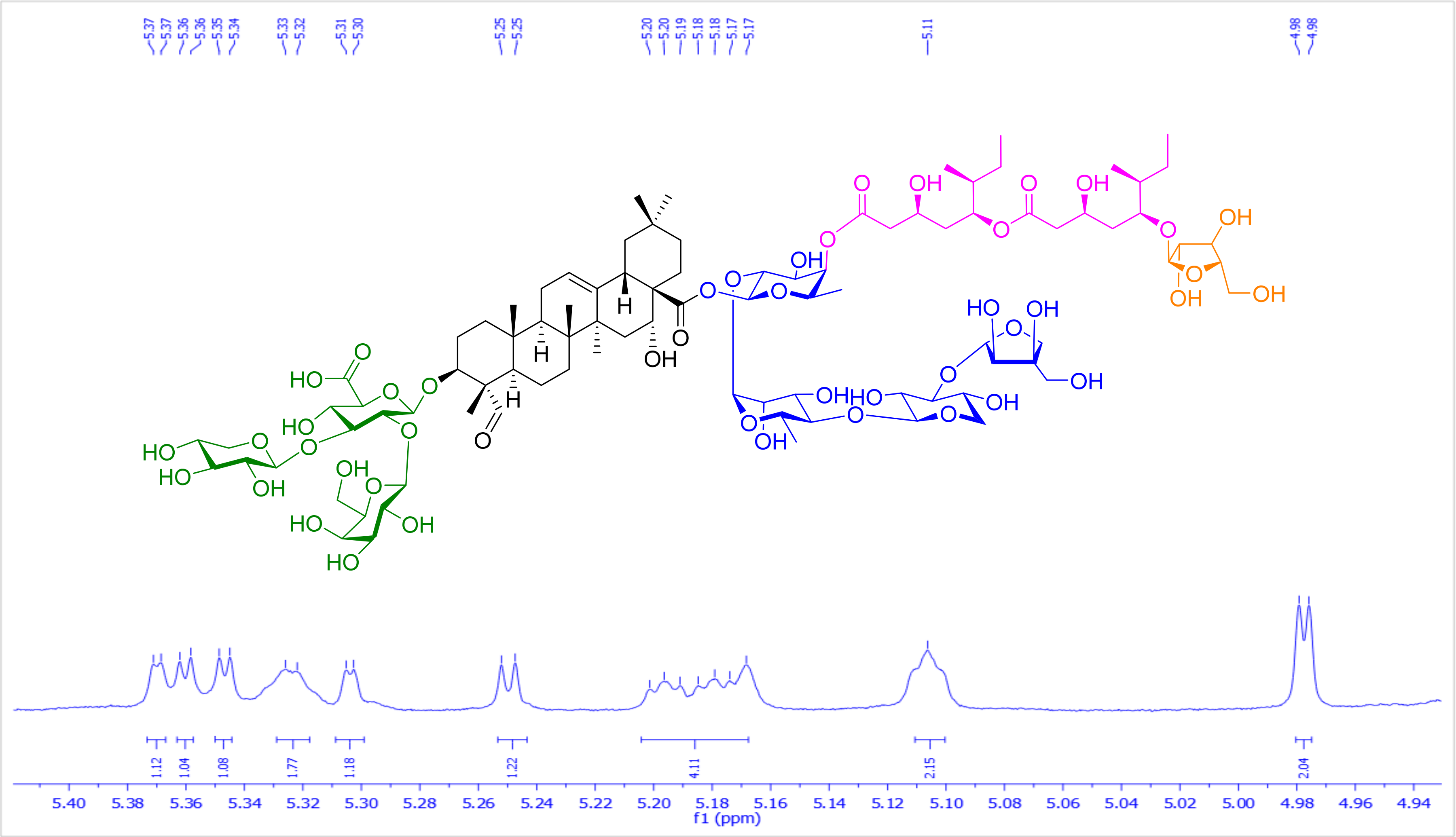

Figure S4: Expanded ^{1}H-NMR spectrum (3.00-4.80 ppm) of semi-purified QS-21 (**1**) produced in *benthamiana*, recorded in MeOH-d_4, 600 MHz

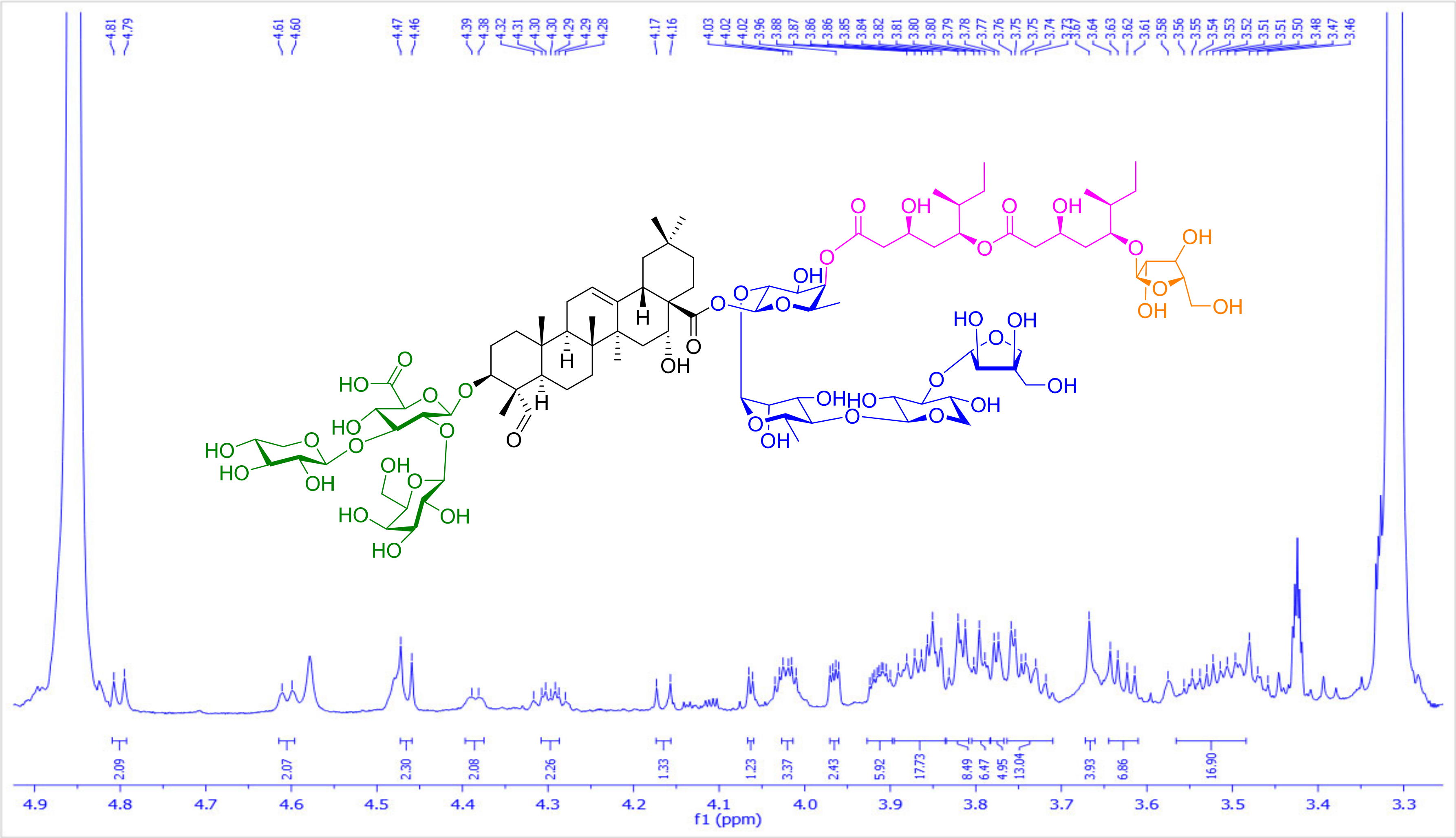

Figure S5: Expanded ^{1}H-NMR spectrum (0.5-3.00 ppm) of semi-purified QS-21 (**1**) produced in *benthamiana*, recorded in MeOH-d_4, 600 MHz

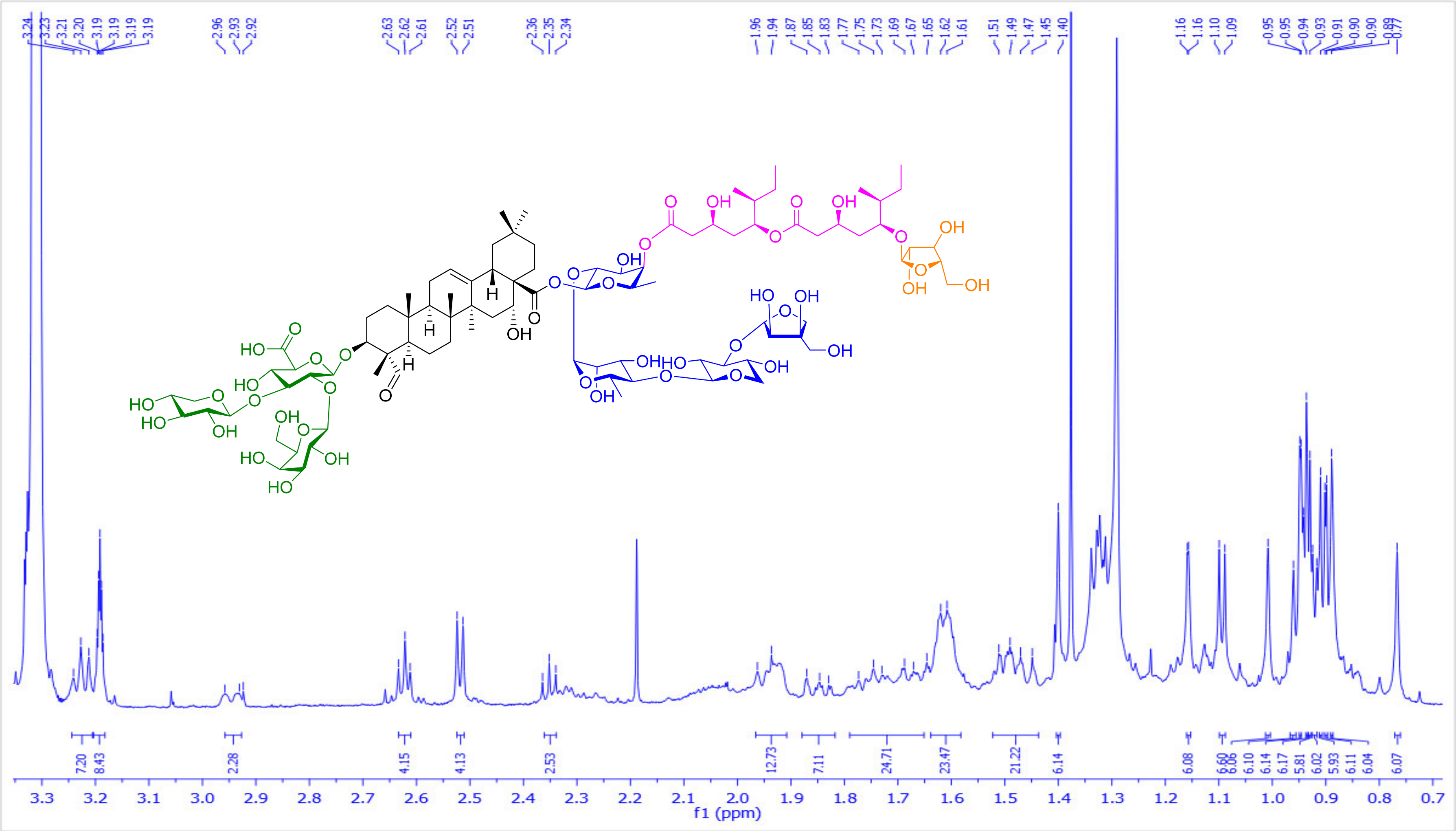

Figure S6: Comparison between ^{1}H-NMR spectrum of **semi-purified QS-21 produced in *benthamiana***, and **QS-21 standard**, both recorded in MeOH-d_4, 600 MHz

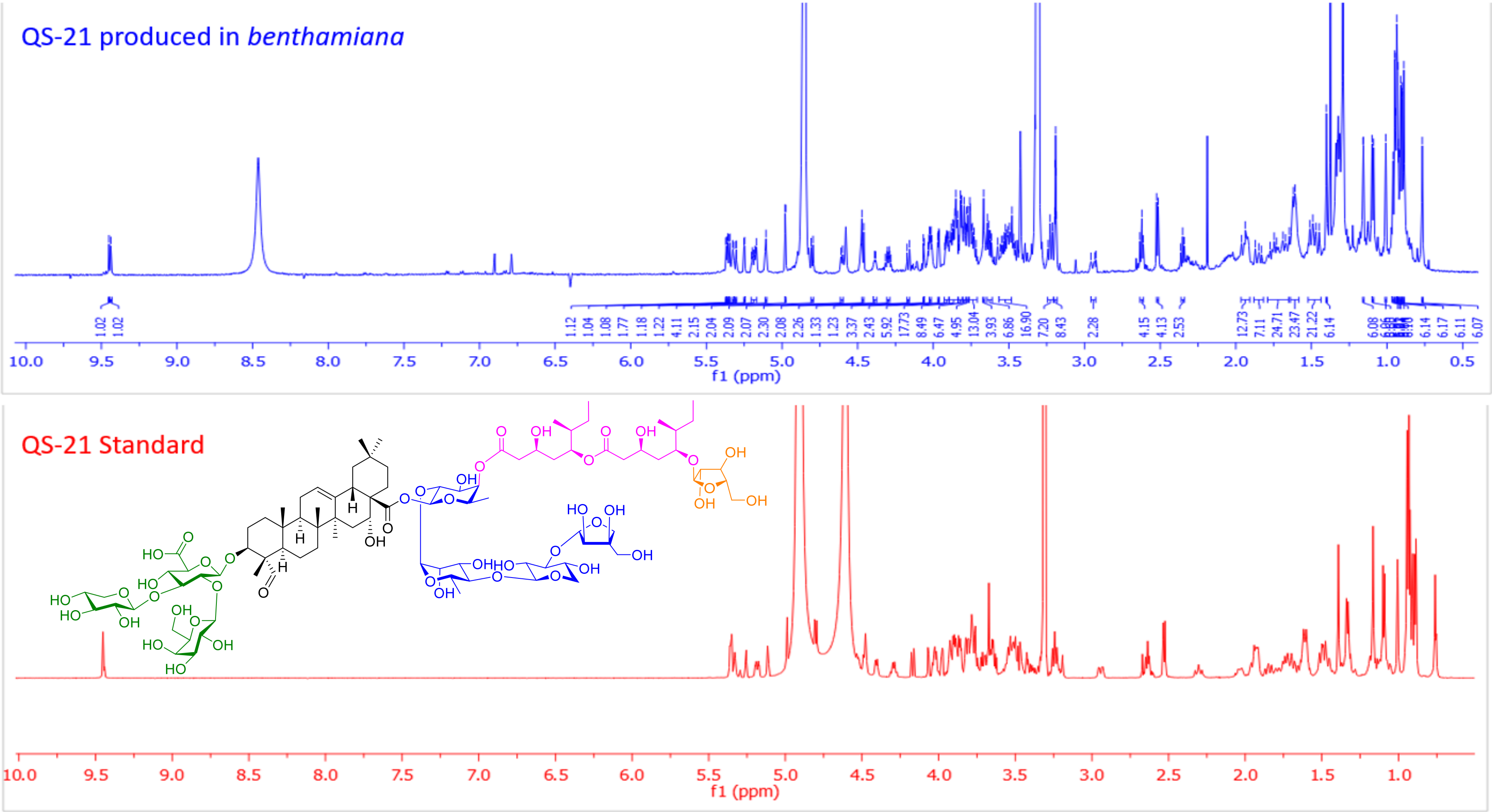

Figure S7: Expanded ^{1}HNMR spectral comparison between **QS-21 standard** and **semi-QS-21 produced in *benth*** (**4.00-4.95 ppm**), recored in MeOH-d_4, 600 MHz

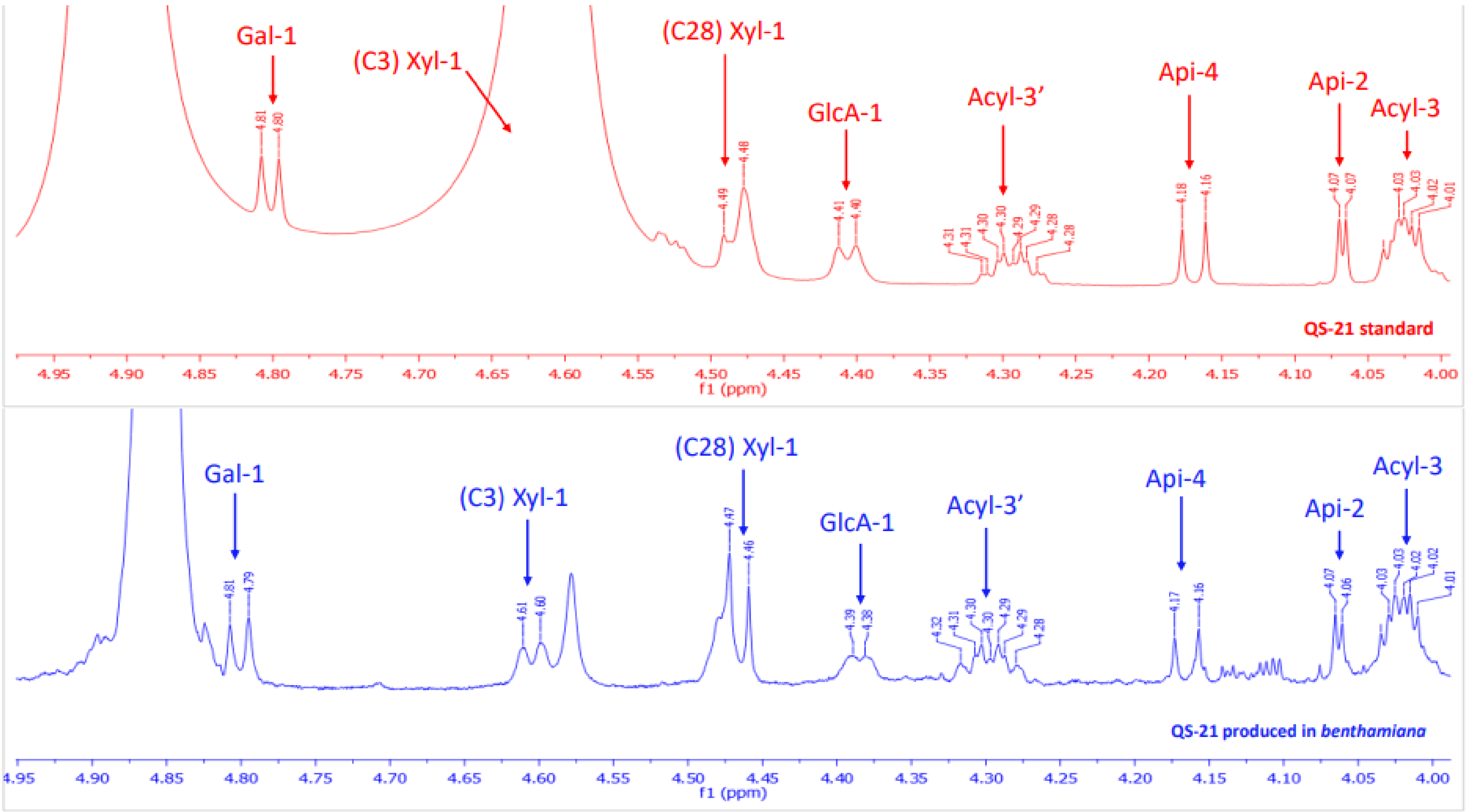

Figure S8: Expanded ^{1}HNMR spectral comparison between **QS-21 standard** and **semi-QS-21 produced in *benth*** (4.96-5.38 ppm), recored in MeOH-d_4, 600 MHz.

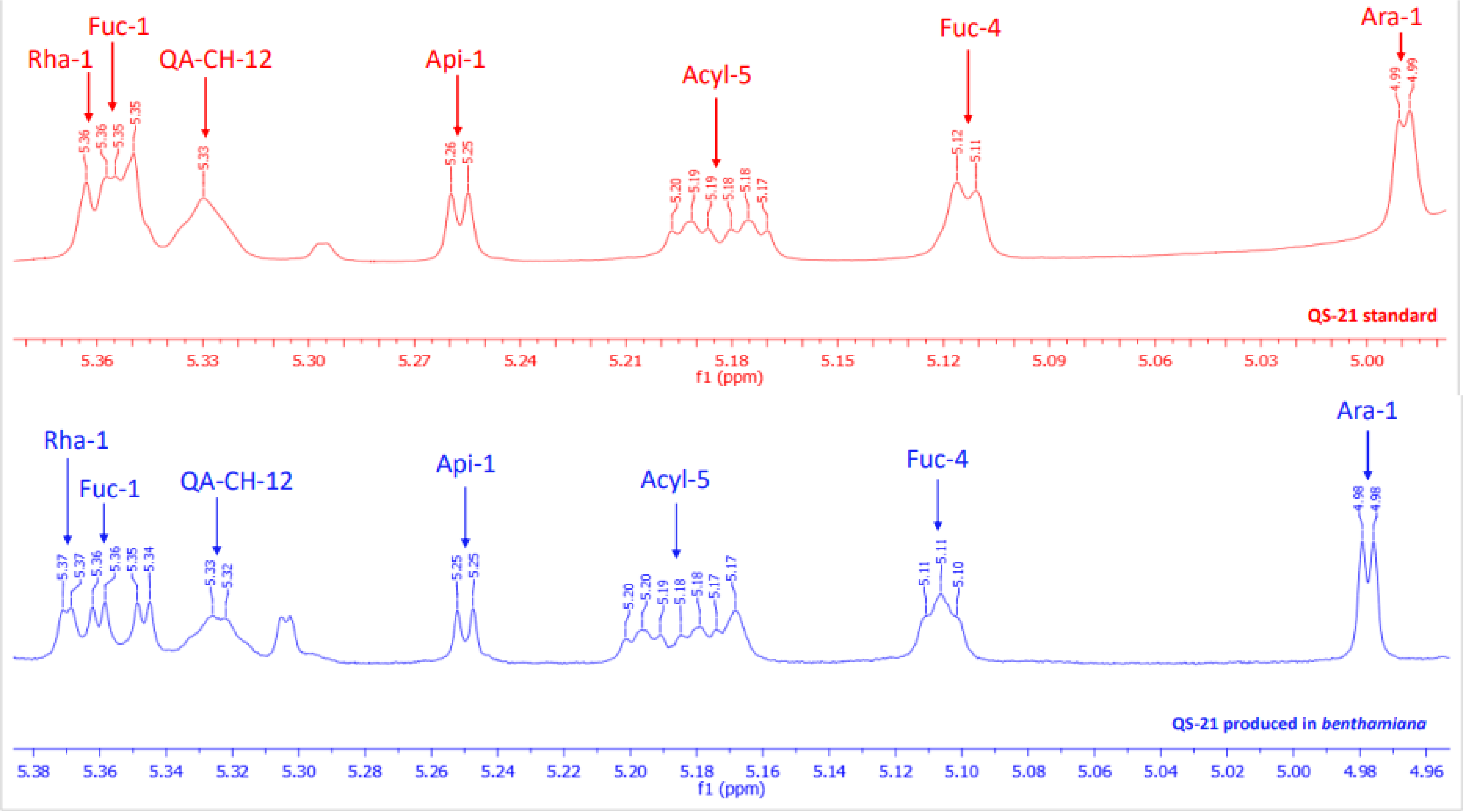

Figure S9: ^{1}H-^{1}H COSY spectrum of semi-purified QS-21 (**1**) produced in *benthamiana*, recorded in MeOH-d_4, 600 MHz

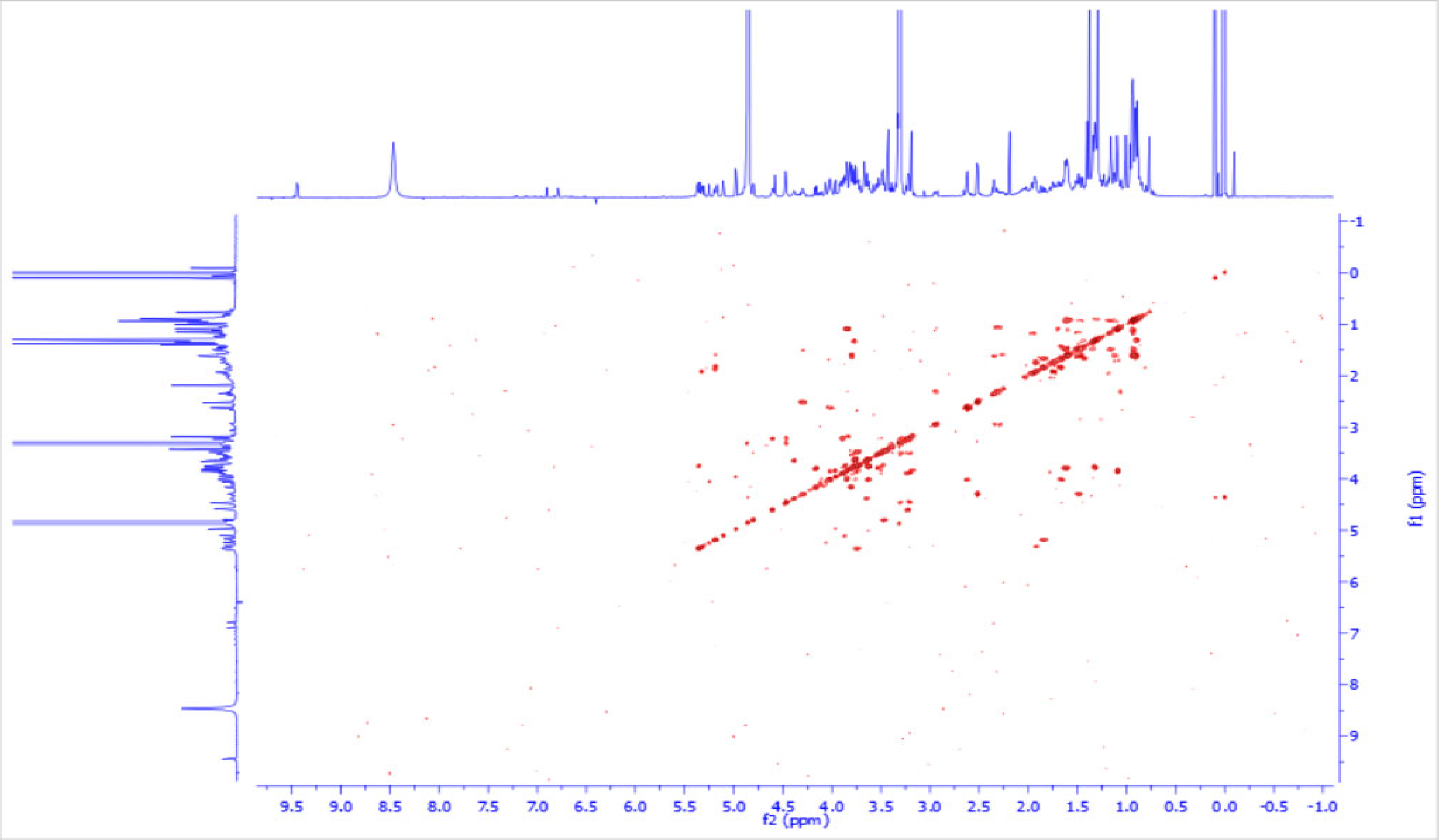

Figure S10: ^{1}H-^{13}C HSQC spectrum of semi-purified QS-21 (**1**) produced in *benthamiana*, recorded in MeOH-d_4, 600/150 MHz

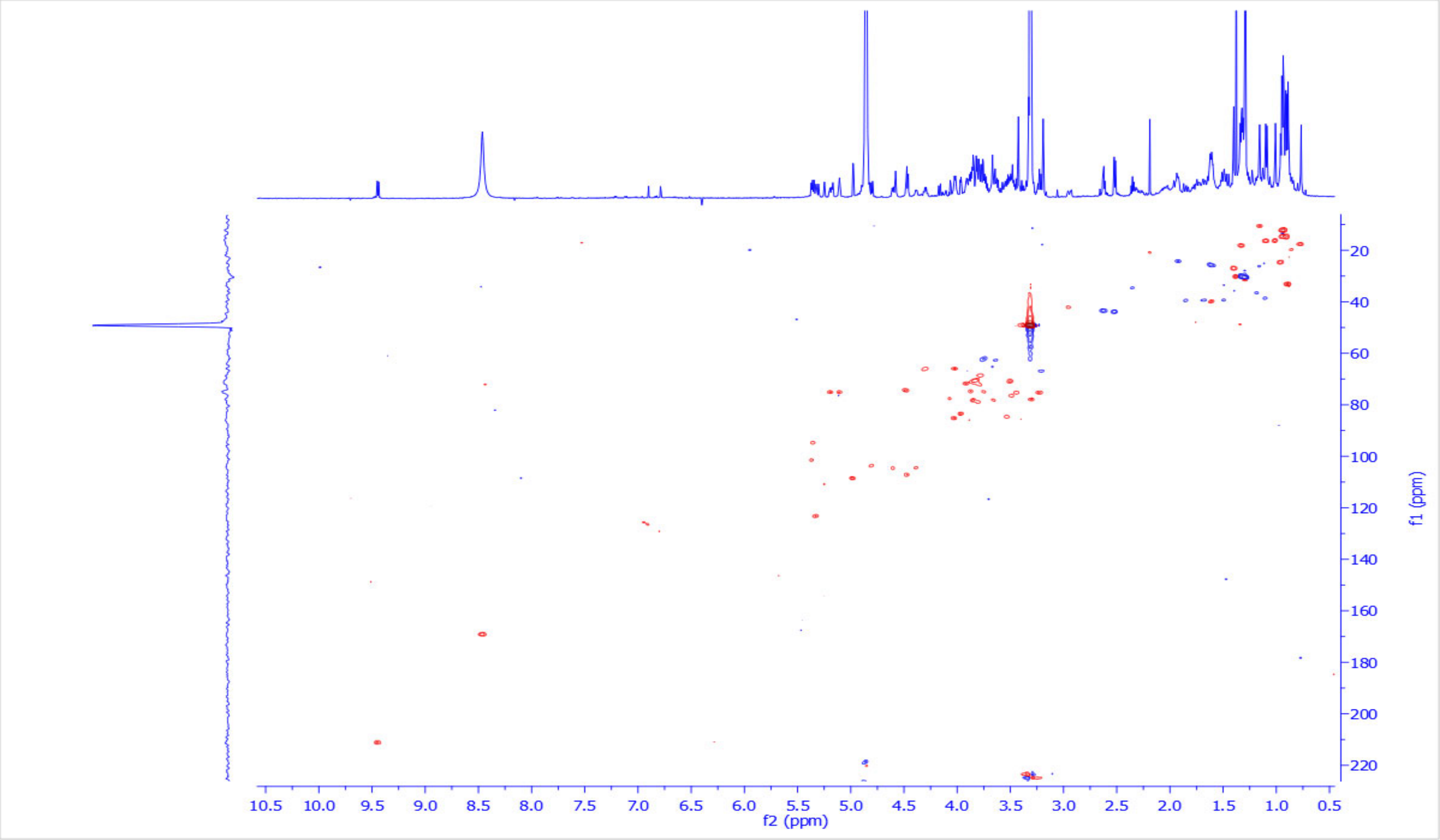

Figure S11: Expanded ^{1}H-^{13}C HSQC spectrum of the anomeric region (4.20-5.50/60-130 ppm) of semi-purified QS-21 (**1**) produced in *benthamiana*, recorded in MeOH-d_4, 600/150 MHz

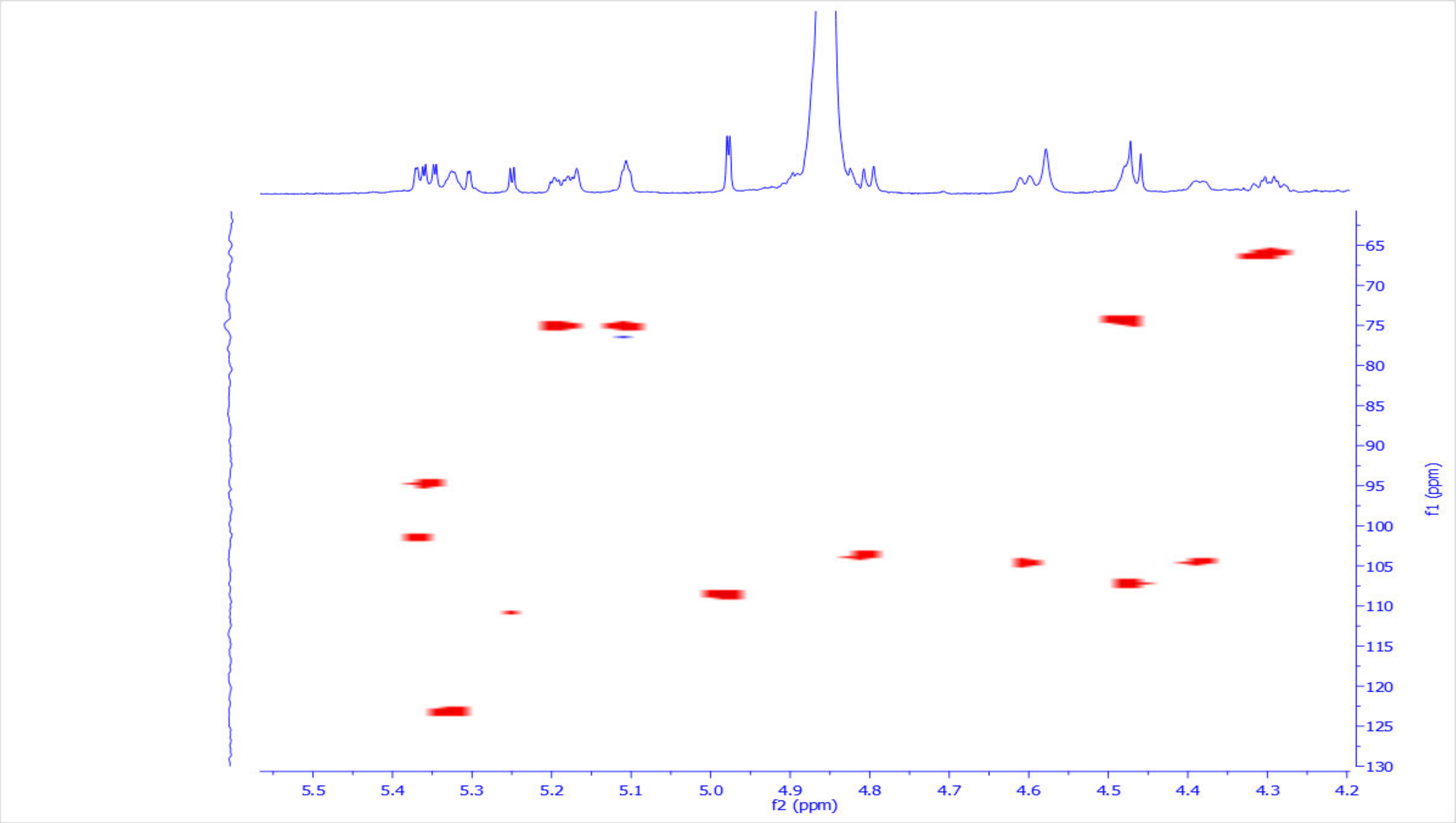

Figure S12: ^{1}H-^{13}C HMBC spectrum of semi-purified QS-21 (**1**) produced in *benthamiana*, recorded in MeOH-d_4, 600/150 MHz

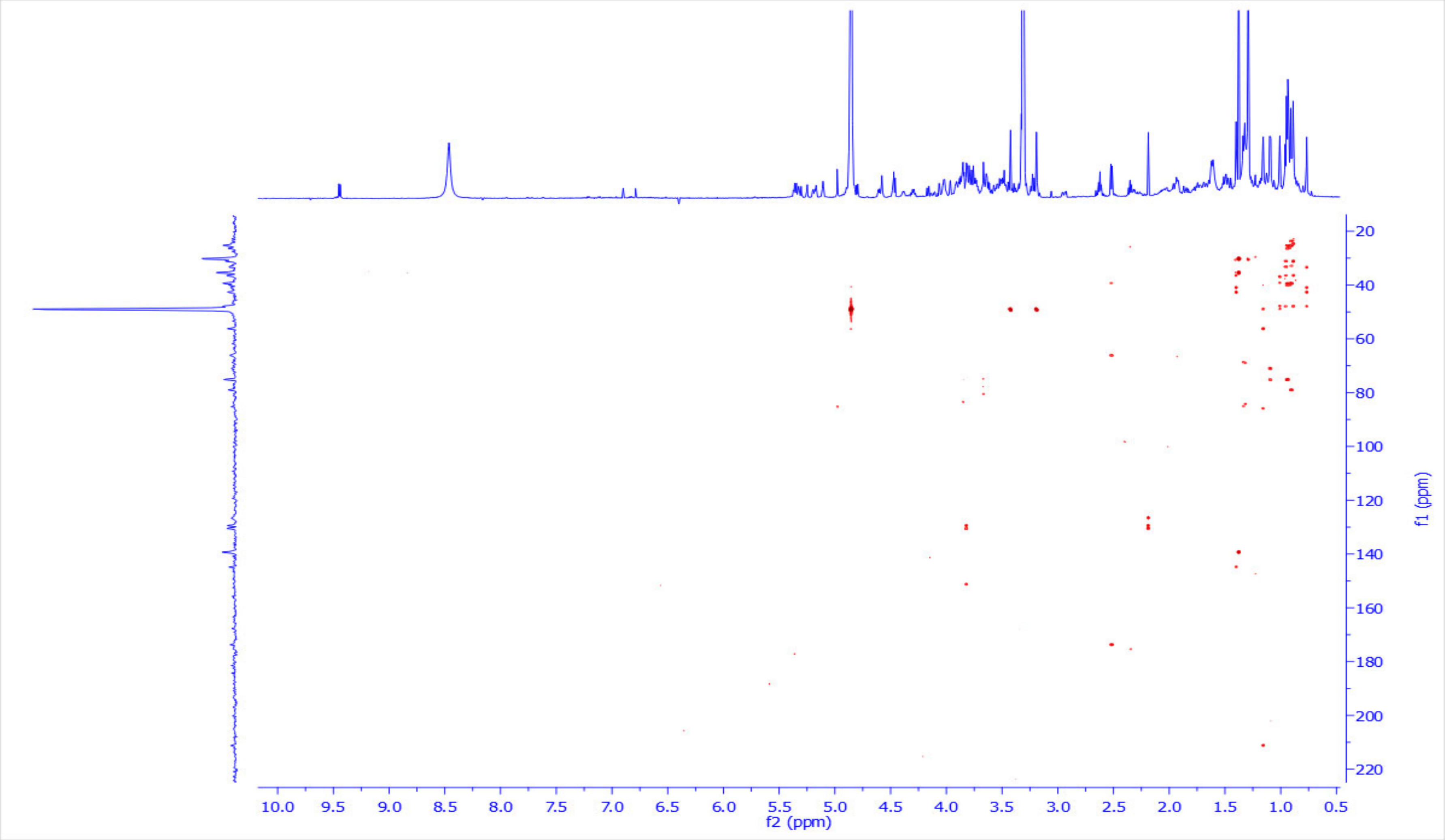

Figure S13: Expanded key ^{1}H-^{13}C HMBC spectrum (4.96-5.00/76-81.5 ppm) of semi-purified QS-21 (**1**) produced in *benthamiana*, recorded in MeOH-d_4, 600/150 MHz

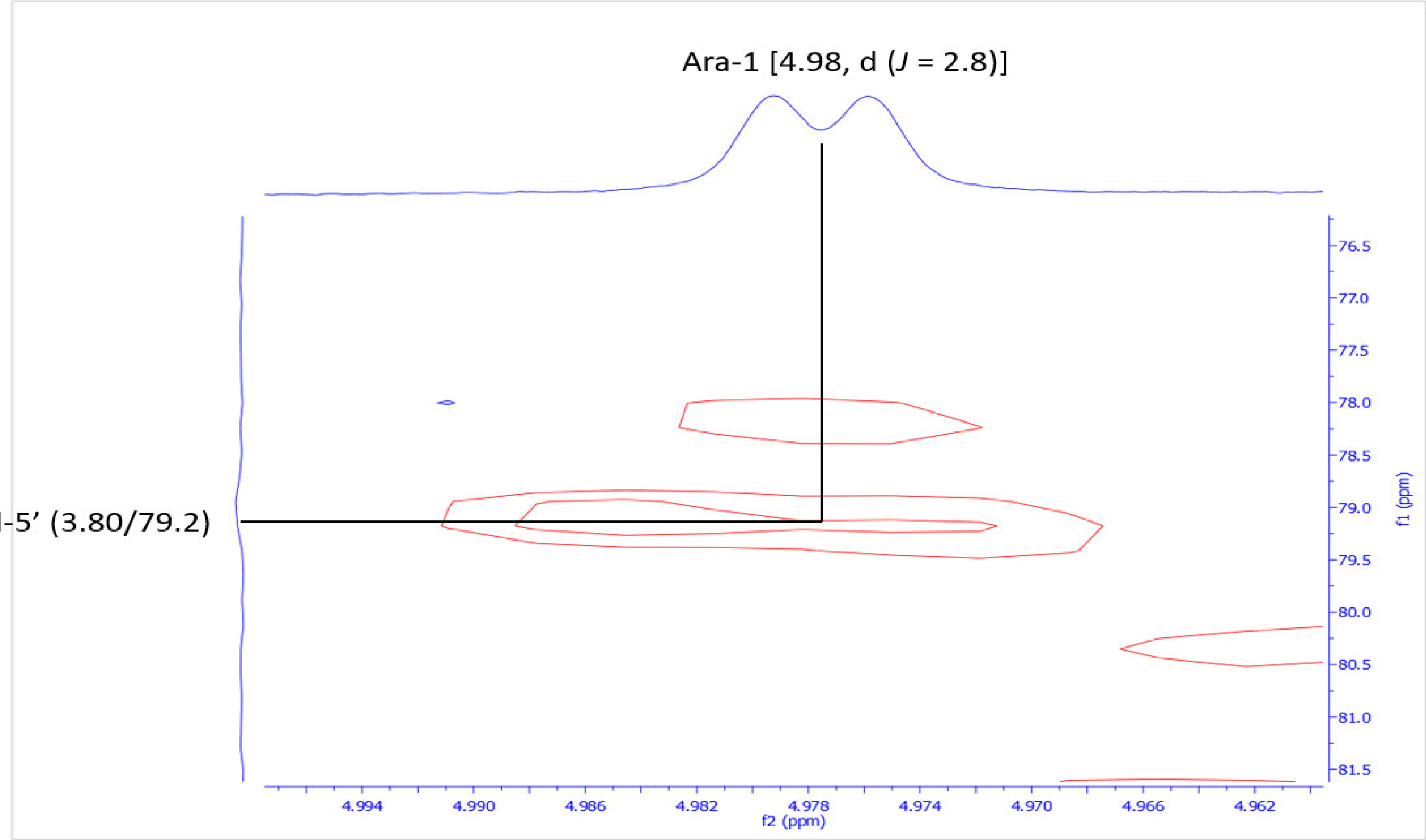

Figure S14: LC-HR-ESI(-ve)-MS of non-separable mixture (1:1) of QS-21/Des-apiosyl-QS-21

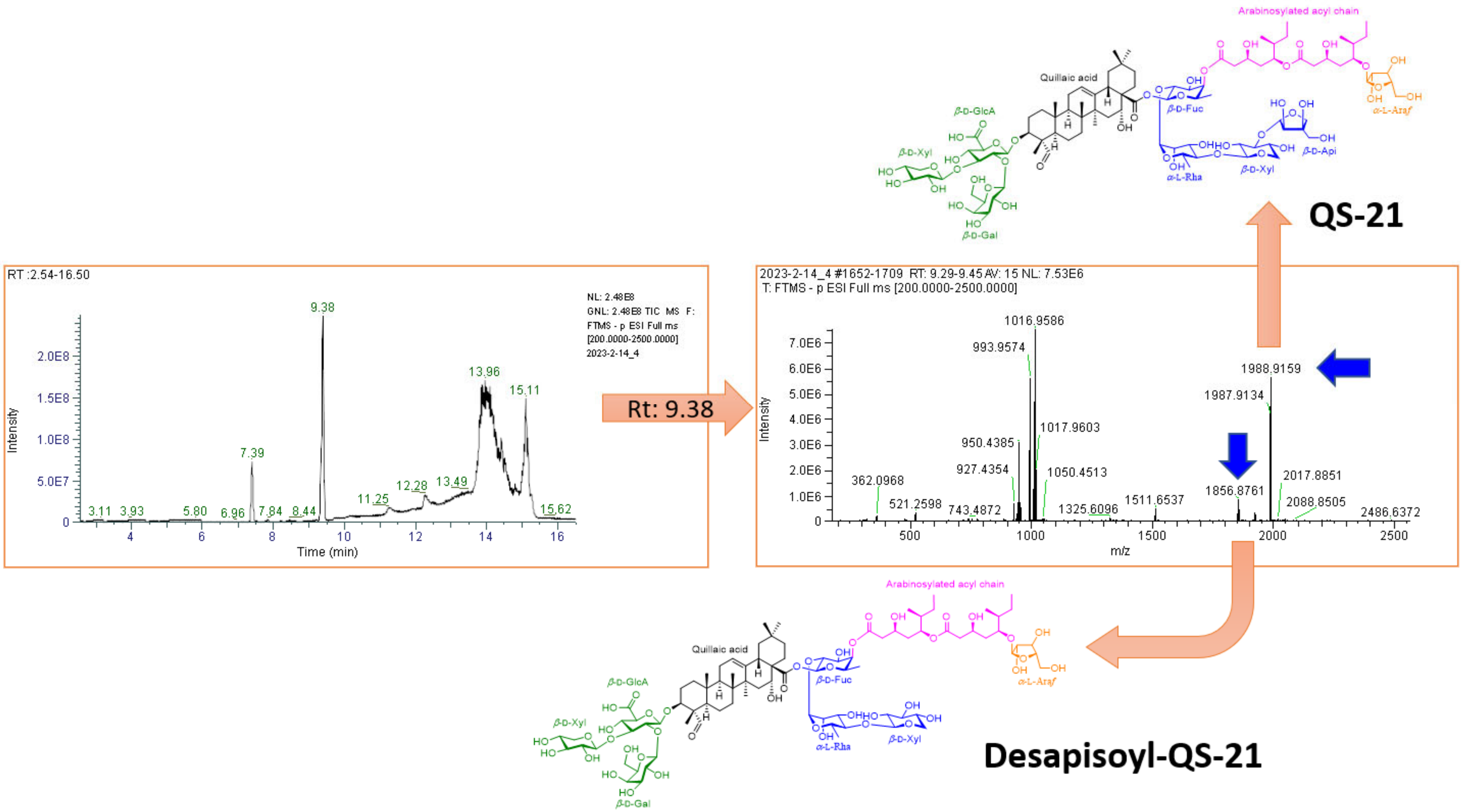

Supplement: Supplementary file 4 — Supplementary Data 3. CAS SciFinder search output. Supplementary Data 5. Confirmation of the structure of des-arabinosyl QS-21 (d-apiose; 11; used in UGT73CZ2 assays). Supplementary Data 6. Spectral confirmation of QS-21/des-apiosyl-QS-21 produced and purified from N. benthamiana. [file 41589_2023_1538_MOESM4_ESM.pdf]
